# Proangiogenesis effects of compound danshen dripping pills in zebrafish

**DOI:** 10.1186/s12906-022-03589-y

**Published:** 2022-04-22

**Authors:** Yang-Xi Hu, Hong-Min You, Chang-Zhen Ren, Bo-Wen Hu, Lu-Jun Zhang, Yan-Da Zhang, Zhi-Qing He, Ru Ding, Zhi-Fu Guo, Chun Liang

**Affiliations:** 1 Department of Cardiology, Changzheng Hospital, Naval Medical University, Shanghai, 200003 China; 2 Department of Cardiology, Changhai Hospital, Naval Medical University, Shanghai, 200433 China

**Keywords:** Angiogenesis, Wound healing, Traditional Chinese medicine, Myocardial infarction, Cardiovascular, Zebrafish

## Abstract

**Background:**

The compound Danshen Dripping Pill (CDDP), which is a mixture of extracts from *Radix Salviae* and *Panax notoginseng*, is a patented traditional Chinese medicine that is widely used in multiple countries for relieving coronary heart disease (CHD), but its pharmacological mechanism has not been fully elucidated. In this study, we screened the key pharmacological pathways and targets of CDDP that act on CHD using a network pharmacology-based strategy, and the angiogenic activity of CDDP was directly visually investigated in zebrafish embryos in vivo.

**Methods:**

The potential therapeutic targets and pathways were predicted through a bioinformatics analysis. The proangiogenic effects of CDDP were examined using vascular sprouting assays on subintestinal vessels (SIVs) and optic arteries (OAs) as well as injury assays on intersegmental vessels (ISVs). Pharmacological experiments were applied to confirm the pathway involved.

**Results:**

Sixty-five potential therapeutic targets of CDDP on CHD were identified and enriched in the *PI3K/AKT* and *VEGF/VEGFR* pathways*.* An in vivo study revealed that CDDP promoted angiogenesis in SIVs and OAs in a dose-dependent manner and relieved the impairments in ISVs induced by lenvatinib, a VEGF receptor kinase inhibitor (VRI). In addition, *Vegfaa* and *Kdrl* expression were significantly upregulated after CDDP treatment. Furthermore, the proangiogenic effect of CDDP could be abolished by *PI3K/AKT* pathway inhibitors.

**Conclusions:**

CDDP has a proangiogenic effect, the mechanism of which involves the *VEGF/VEGFR* and *PI3K/AKT* signaling pathways. These results suggest a new insight into the cardiovascular protective effect of CDDP.

**Supplementary Information:**

The online version contains supplementary material available at 10.1186/s12906-022-03589-y.

## Introduction

Current data from the World Health Organization show that cardiovascular diseases (CVDs) are still the leading cause of death among noncommunicable diseases and account for approximately one-third of all deaths worldwide [1]. Among CVDs, coronary heart disease (CHD) may cause angina pectoris (AP), which signals the risk of myocardial infarction and sudden death and is also recognized as a predictor of many other major adverse cardiac events. However, therapies for CHD and other ischemic CVDs are limited.

Angiogenesis is a process by which new blood vessels sprout from pre-existing blood vessels and is affected by *VEGF/VEGFR* signaling [2]. Endothelial cells (ECs), with physiological functions such as proliferation, migration, and tube formation play a key role in the initiation of angiogenesis [3]. Drugs targeting both proangiogenic and antiangiogenic processes have been developed to help maintain the normal physiological condition of ECs [4]. In recent decades, to suppress the nutrient supply of tumors, antiangiogenic drugs have been widely applied in cancer treatment [2], and proangiogenic therapy is important for treating and preventing the serious consequences of CHD [5]. Enhancing angiogenesis facilitates the delivery of oxygen and nutrients to the ischemic site of cardiomyocytes, which benefits the process of myocardial healing.

Compound Danshen Dripping Pill (CDDP), a patented traditional Chinese medicine (TCM), is widely used for relieving AP, ischemic heart diseases and coronary arteriosclerosis in China. It was first launched in 1995 by the China Food and Drug Administration (CFDA) and was approved by the US Food and Drug Administration (FDA) in 2016 to enter phase III (NCT01659580) clinical trials [6]. Currently, CDDP is marketed as a prescription or nonprescription drug for the prevention and treatment of ischemic CVDs in Singapore, Canada, South Korea, Mongolia, and Vietnam. However, the mechanism of CDDP in the treatment of CHD has not been fully elucidated. According to previous reports, CDDP is a mixture of extracts from Radix Salviae (Danshen), Panax notoginseng (Sanqi), and other herbs [7]. These active components of CDDP have been reported to improve microcirculation in ischemic/reperfusion injury and other CVDs [8], and we recently discovered that pretreatment with CDDP prevents lipid infusion-induced cardiac microvascular disorder (CMD) in a mouse model [9]. However, whether angiogenesis is involved in the cardiovascular protective effect of CDDP remains unknown.

The zebrafish has become an ideal animal model to study angiogenesis, in part because its responses to many pro- or antiangiogenic drugs are comparable to those observed in mammalian systems [10–12]. Remarkably, the formation of the cardiovascular system is highly typical in developing zebrafish larvae, and the subintestinal vessel plexus (SIVs), intersegmental vessels (ISVs) and optic arteries (OAs) can be visualized microscopically [13, 14]. Furthermore, the zebrafish embryo assay allows the identification of side effects, including toxicity and developmental delay [15]. In this study, we screened the key pharmacological pathways and targets of CDDP that act on CHD using a network pharmacology-based strategy. The angiogenic activity of CDDP was then directly visually investigated in zebrafish embryos in vivo. Our results provide novel evidence that CDDP exhibits proangiogenic activity by inducing the formation of new blood vessels and stimulating endothelial cell proliferation; hence, CDDP could also serve as a remedy targeting *VEGF/VEGFR* and *PI3K/AKT* signaling for ischemic CVDs.

## Materials and methods

### Network pharmacology-based screening

The main active ingredients of CDDP, Radix Salviae and Panax notoginseng, were recognized based on traditional medicine guidelines and the Chinese Clinical Trial Registry up to April 30, 2020 (all components are listed in Supplemental Table 1). The constituents were screened using the Traditional Chinese Medicine Systems Pharmacology Database and Analysis Platform (TCMSP) (https://old.tcmsp-e.com/tcmsp.php) with the filters OB (oral bioavailability) ≥ 30% and DL (drug-likeness) ≥ 0.18. Target prediction of the active compounds was then performed using TCMSP and confirmed through the DrugBank database (https://go.drugbank.com/). For CHD-related target screening, we searched the keyword ‘coronary heart disease’ in the GeneCards database (https://www.genecards.org/). The target genes obtained were then pooled using R software, and the genes in common were regarded as hub genes to be further screened. The protein–protein interactions of these hub genes were constructed using the STRING database (http://string-db.org/) with a minimum required interaction score of 0.950. The Gene Ontology (GO) and Kyoto Encyclopedia of Genes and Genomes (KEGG) [16] pathway enrichment analyses were performed and visualized using R software.

### Chemicals and reagents

CDDP extracts were purchased from Tasly Pharmaceutical Group Co. Ltd. (Tianjin, China, 27 mg/package, batch number 20210103) and dissolved using dimethyl sulfoxide (DMSO) (Sigma–Aldrich, Munich, Germany) to make different concentrations. In previous research, the chemical profile of CDDP had been fully investigated using HPLC [17]. The final DMSO concentration was ≤ 0.5% weight/volume (w/v) in all treatment groups. The VEGF receptor kinase inhibitor (VRI) lenvatinib, PI3K inhibitor LY294002 (LY) and AKT inhibitor VIII (AKTi) were obtained from Beyotime Technology (Shanghai, China).

### Zebrafish maintenance and feeding

Transgenic zebrafish embryos, *Tg(fli1: EGFP)*, were obtained from the Laboratory of the Department of Cardiology, Shanghai Changhai Hospital, maintained at 28 °C on a 14/10-h light/dark cycle and staged as previously described [18]. To prevent pigmentation during embryonic development, 0.003% 2-phenylthiourea (PTU, Sigma–Aldrich, CA, USA) was added to the culture water. The development age of the embryos corresponded to hours post-fertilization (hpf) and was staged according to Kimmel et al. [19]. The embryos applied in the experiments were all normally developed and reached the indicated stages. Approximately one hundred embryos were collected in a 6 cm dish and treated with Danieu’s solution with 0.04% tricaine (Sigma–Aldrich, CA, USA) for immobilization before the corresponding assays.

### Vascular sprouting assay

Twenty-four hpf WT zebrafish were used to perform toxicity screening of CDDP. After being dechorionated with 0.5 mg/mL Pronase E (Roche, IN, USA), 24 hpf Tg(fli1:EGFP) embryos were incubated with CDDP at safe concentrations (0.3, 0.6, 0.9 and 1.2 mg/mL) for 48 h. DMSO (≤ 0.5%) served as the vehicle control and was equivalent to no treatment. For each concentration, three replicates of fifteen embryos were set up and exposed to 1 mL of test medium. The entire screening system was performed in a 24-well tissue culture plate. At 72 hpf, the embryos were examined using a fluorescence microscope (Leica, Wetzlar, Germany) and each group tested was scored for mortality and morphological defects. The numbers of sprouting vessels in SIVs and abnormally developed vessels in OAs were photographed and counted for quantitative analysis. For the ISV injury and restoration assay, twenty-four hpf zebrafish embryos were treated with VRI (100 nM) for 24 h and then incubated with or without various concentrations of CDDP for another 24 h. LY and AKTi were added to the system 30 min before CDDP when used. The intact and deficient vessels of intersegmental vessels (ISVs) were calculated for each embryo. The ISV index was calculated as: (the number of intact vessels × 1) + (the number of defective vessels × 0.5).

### Total RNA extraction and real-time quantitative PCR (rt-qPCR)

Total RNA was isolated from CDDP-treated embryos using TRIzol reagent (Thermo Fisher, MA, USA) and reverse transcribed using a FastQuant RT Kit (Tiangen, Beijing, China). Quantitative RT–PCR assays were performed using SYBR Green Real-time PCR Master Mix (Toyobo, Osaka, Japan). The primers used for mRNA detection are listed in Supplementary Table 2 and were provided by Biosune Ltd., Shanghai, China.

### Statistical analysis

All experiments were conducted more than three times. The data are presented as the mean ± SEM and were analyzed using GraphPad Prism 7.0 software. Normality was evaluated using the Shapiro–Wilk test. The Mann–Whitney test or Kruskal–Wallis test followed by Dunn's multiple comparisons test was then performed for the data that did not fit a normal distribution. Whereas for the data that followed a normal distribution, Levene's test of homogeneity of variance was further performed. When the data exhibited homogeneity of variance, a one-way ANOVA was applied. A *P* < 0.05 was considered statistically significant.

## Results

### Screening of potential target genes and pathways of CDDP

**Fig. 1:**
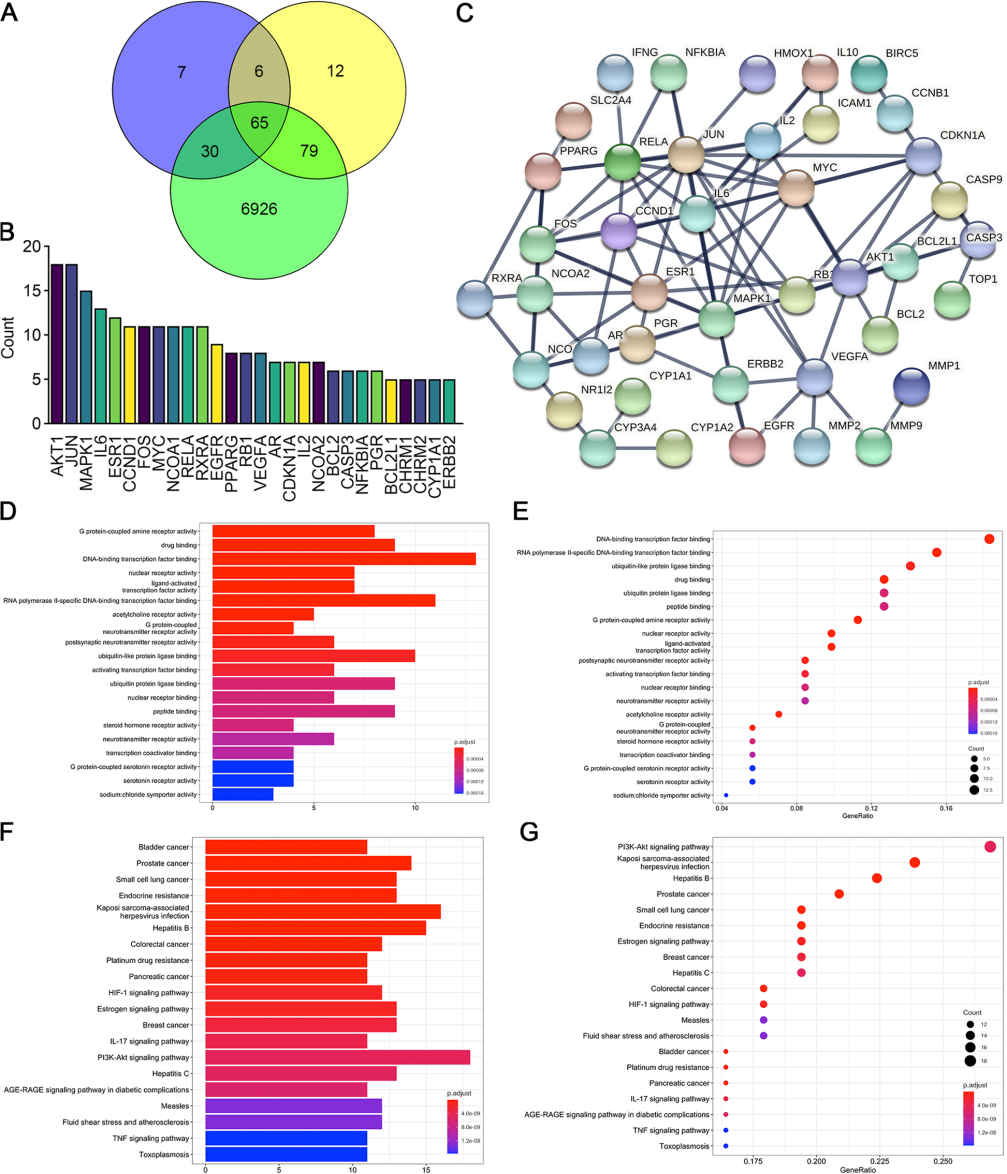
Screening of potential target genes andpathways of CDDP. **A** We obtained 7,100 coronary heart disease-related genes from GeneCards, 108 target genes of *Panax notoginseng* and 162 target genes of *Radix Salviae* from the TCMSP database. The 65 intersecting genes were considered important targets and were further studied. **B** Among the top 28 potential target genes predicted to be regulated by CDDP, *AKT1* and *JUN* ranked highest. **C** Protein–protein interactions (PPIs) of the 65 targets (interaction score ≥ 0.950). The separated proteins were predicted to be less related and then deleted. **D, E** GO enrichment analysis showing the top 20 molecular biological functions of the target genes. This result demonstrated that DNA-binding transcription factor binding was the most common gene function. The abscissa represents the ratio of related genes, and the ordinate lists the functions corresponding to proteins. The bubble size represents the number of genes, whereas the color represents the *P* value. **F, G** KEGG pathway enrichment analysis showing the 20 most likely pathways involved; the *PI3K-AKT* signaling pathway ranked first

The target genes of the two ingredients of CDDP, *Radix Salviae* and *Panax notoginseng*, were screened using the TCMSP database. A total of 65 compounds were identified in *Radix Salviae* (Supplementary Table 3), and 8 were identified in *Panax notoginseng* (Supplementary Table 4). We then obtained known targets of these ingredients, which are listed in Supplementary Table 5 and Supplementary Table 6, from the TCMSP databases. Furthermore, 7,100 CHD-related genes were obtained from GeneCards databases (Supplementary Table 7). A Venn plot revealed that 65 CHD-related genes were also targeted by CDDP (Fig. 1A, Supplementary Table 8). The count of the ingredients predicted to regulate each of the target genes showed that *AKT1* and *JUN* involved the most constituents of CDDP (Fig. 1B). The protein–protein interaction (PPI) network of these 65 target genes was constructed, and nodes with high confidence were identified by limiting their interaction scores to more than 0.950 and excluding the separated proteins (Fig. 1C). We noticed that VEGFA and AKT1 were both predicted to be hub proteins in the PPI network. GO analysis of the overlapping targets showed that DNA-binding transcription factor binding, RNA polymerase II-specific DNA-binding transcription factor binding, and ubiquitin-like protein ligase binding were the top three enriched functions (Fig. 1D). In addition, 13 proteins were associated with DNA-binding transcription factor binding activity with a gene ratio of 0.231 (Fig. 1E). Thus, CDDP compounds might potentially target these proteins to regulate kinase activity, in particular the DNA-binding transcription factor binding process. As shown by the KEGG analysis, the top three significantly enriched pathways among the 65 overlapping genes were *PI3K/AKT* (18 proteins), Kaposi sarcoma-associated herpesvirus infection related (16 proteins), and Hepatitis B related signaling pathways (15 proteins), (Fig. 1F). The *PI3K/AKT* pathway ranked first with 18 proteins (Fig. 1G) and has been reported to play an important role in angiogenesis [20]. Moreover, VEGFA, one of the most powerful activators of *VEGF/VEGFR* signaling, was also identified as a hub protein in the PPI network above (Fig. 1C). We surmised that both the *VEGF/VEGFR* and *PI3K/AKT* pathways were targeted by CDDP in treating CHD.

### The proangiogenic effect of CDDP on SIVs

**Fig. 2:**
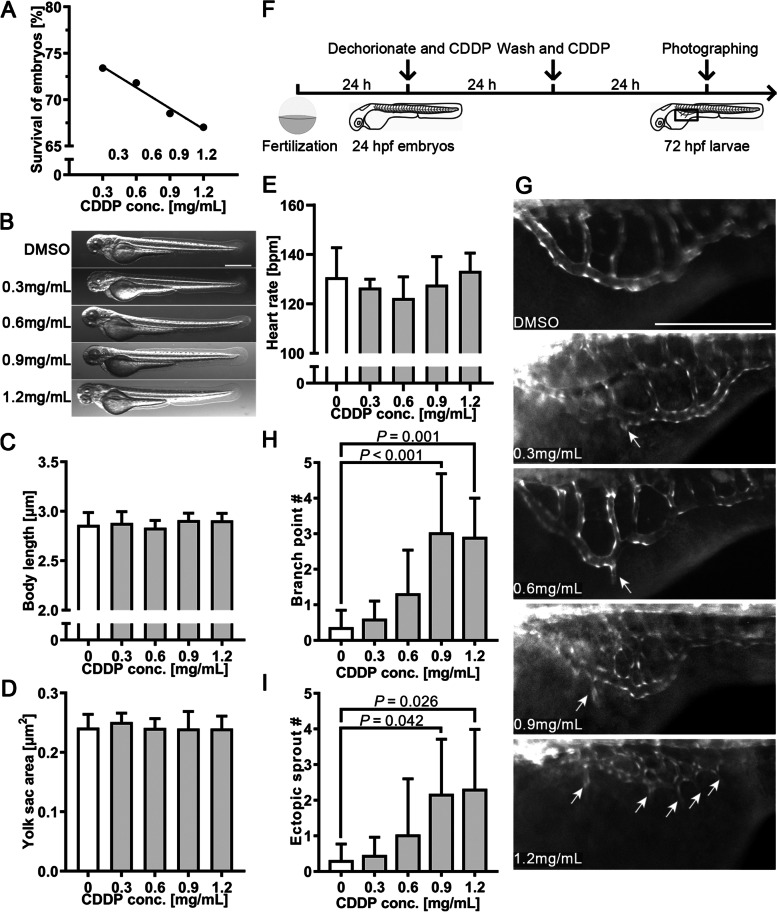
The proangiogenic effect of CDDP on subintestinal vessels (SIVs). **A** Twenty-four hpf embryos were treated with various concentrations of CDDP (0.3, 0.6, 0.9 and 1.2 mg/mL) for 48 h. The survival rate was recorded. **B** The toxicity screening of CDDP did not significantly affect the early-stage appearance of embryos. Scale bar, 50 μm. **C** Body length was not affected by CDDP treatment, indicating that embryo development was normal. **D** The yolk sac area was also unchanged, which meant that the nutritional status was maintained at a similar level. **E** Heart rates of the treatment groups were not notably different from those of the control group. **F** Schematic representation of the experimental design for **G-, I**. Twenty-four hpf transgenic zebrafish *Tg(fli1: EGFP)* embryos were cotreated with CDDP (0.3, 0.6, 0.9 and 1.2 mg/mL) for 24 h. After washing with culture water, the embryos were treated with the same concentration of CDDP for another 24 h. The box in 72 hpf larvae indicates the position photographed and displayed in **G**. **G** Representative pictures of each group; arrows indicate branch points and sprouting vessels of SIVs induced by CDDP in zebrafish. Scale bar, 50 μm. **H**, **I** The branch point and sprouting vessel numbers of SIVs were recorded in each zebrafish. Fifteen embryos were included in each group, and the experiment was replicated four times. Data are represented as the mean ± SEM

We employed the transgenic zebrafish line *Tg(fli1: EGFP)*, which specifically expresses enhanced green fluorescence protein (EGFP) in vascular endothelial cells, to evaluate the proangiogenic effect of CDDP. The toxicity of CDDP in zebrafish embryos was evaluated as follows. Twenty-four hours post-fertilization (24 hpf), embryos were treated with various concentrations of CDDP for 48 h because angiogenic vessel development begins at approximately 20 hpf and is completed at 72 hpf [21]. Figure 2A demonstrates that the survival rate decreased with CDDP concentration. The concentration of CDDP causing death to 50% of the tested embryos, or the median lethal concentration (LC50), was calculated to be 5.081 mg/mL. Figure 2B shows that up to 1.2 mg/mL, no significant morphological change in larvae exposed to the tested concentrations of CDDP could be found. Their body length (Fig. 2C) and yolk sac area (Fig. 2D), the indicators reflecting development and nutritional status, respectively, did not change significantly compared with those of the control group. Moreover, the observation of heart rate also showed insignificant differences (Fig. 2E). These results revealed that CDDP did not affect embryonic development during CDDP treatment.

Twenty-four hpf embryos were treated with CDDP as illustrated in Fig. 2F. As the results indicated in Fig. 2G, CDDP increased the branch point number and promoted ectopic vascular sprouting from SIVs (arrows). The count of SIV branch points (Fig. 2H) and ectopic sprouts (Fig. 2I) showed a significant difference between the control group and the CDDP treatment group at 0.9 and 1.2 mg/mL. These results revealed that CDDP enhanced the growth of SIVs in zebrafish.

### CDDP induced endothelial layer thickening and ectopic growth of OAs

**Fig. 3:**
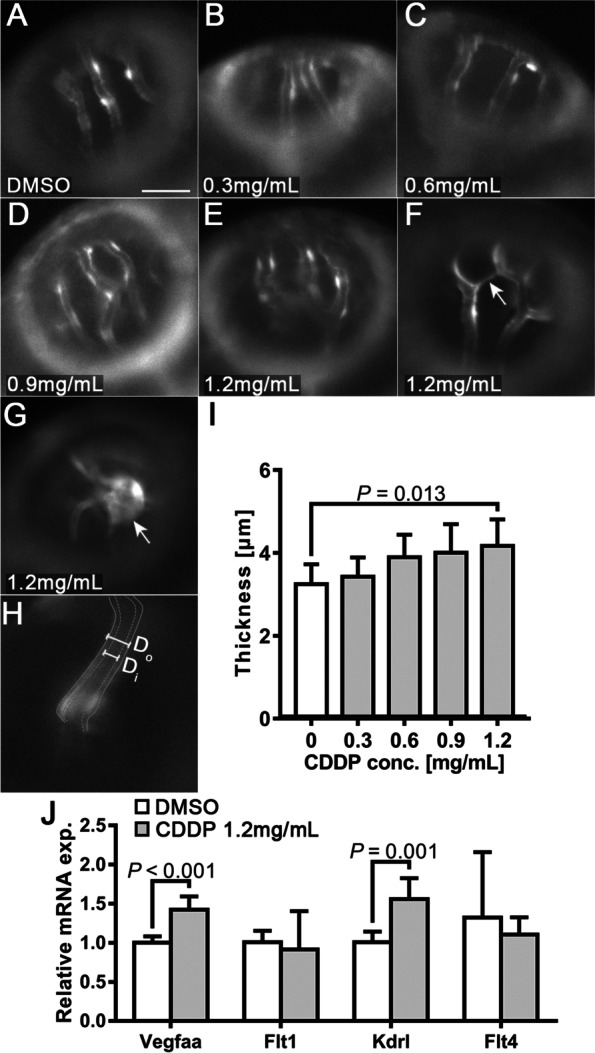
Endothelial layer thickening and ectopic growth of optical vessels in the CDDP treatment group. **A-E** Different treatments with CDDP led to thickening of the endothelial layer in the optical arteries of *Tg(fli1:EGFP)* larvae. Scale bar, 50 μm. In the 1.2 mg/mL CDDP treatment group, ectopic sprouts **F** and tumor-like growth **G** could be observed. **H** Endothelial layer thickness (T) was calculated by subtracting inner diameter (Di) from outer diameter (Do) using the formula *T* = (*Do*—*Di*)/2. **I** Changes in the thickness of the endothelial layer were quantified and are presented as the mean ± SEM. CDDP promoted thickening of the endothelial layer, although only the 1.2 mg/mL group showed statistical significance. *N* = 15 for each group, and the experiment was replicated 4 times. **J** Twenty-four hpf *Tg(fli1: EGFP)* embryos treated with or without CDDP (1.2 mg/mL) for 48 h. The mRNA extracted from 20 embryos from the same group was pooled, and the expressions of *VEGF* receptors (*Flt1*, *Kdrl* and *Flt4*) and *VEGF* (*Vegfaa*) were analyzed by RT–qPCR. Both *VEGF* receptor 2 (*Vegfaa*) and *Kdrl* were significantly upregulated in the CDDP treatment group. The relative expression level of each gene was normalized to the fold change compared with the control group. *N *= 6, and the data are presented as the mean ± SEM

We also observed thickening of the walls of OAs (Fig. 3A-E) and noticed more ectopic growth of OAs in the 1.2 mg/mL CDDP treatment group (indicated by arrows in Fig. 3F-G). The thickness of the endothelial layer of OAs (T) was determined by subtracting the inner diameter (Di) from the outer diameter (Do) using the formula T = (Do—Di)/2 (Fig. 3H). Quantitative analysis confirmed the thickening of OAs in the group treated with 1.2 mg/mL CDDP (Fig. 3I). We further detected changes in the mRNA expression of *Vegfaa* (an isoform of *VEGFA* in zebrafish) and its receptors, Flt1, Kdrl and Flt4, in the whole tissue homogenate of CDDP-treated embryos. Figure 3J demonstrates that the expression levels of both *Vegfaa* and *Kdrl* mRNA were upregulated in the 1.2 mg/mL CDDP treatment group.

### Analysis of target genes involved in FGF, MAPK/ERK, and SREBP 1 and 2 signaling pathways in CDDP-treated embryos

**Fig. 4:**
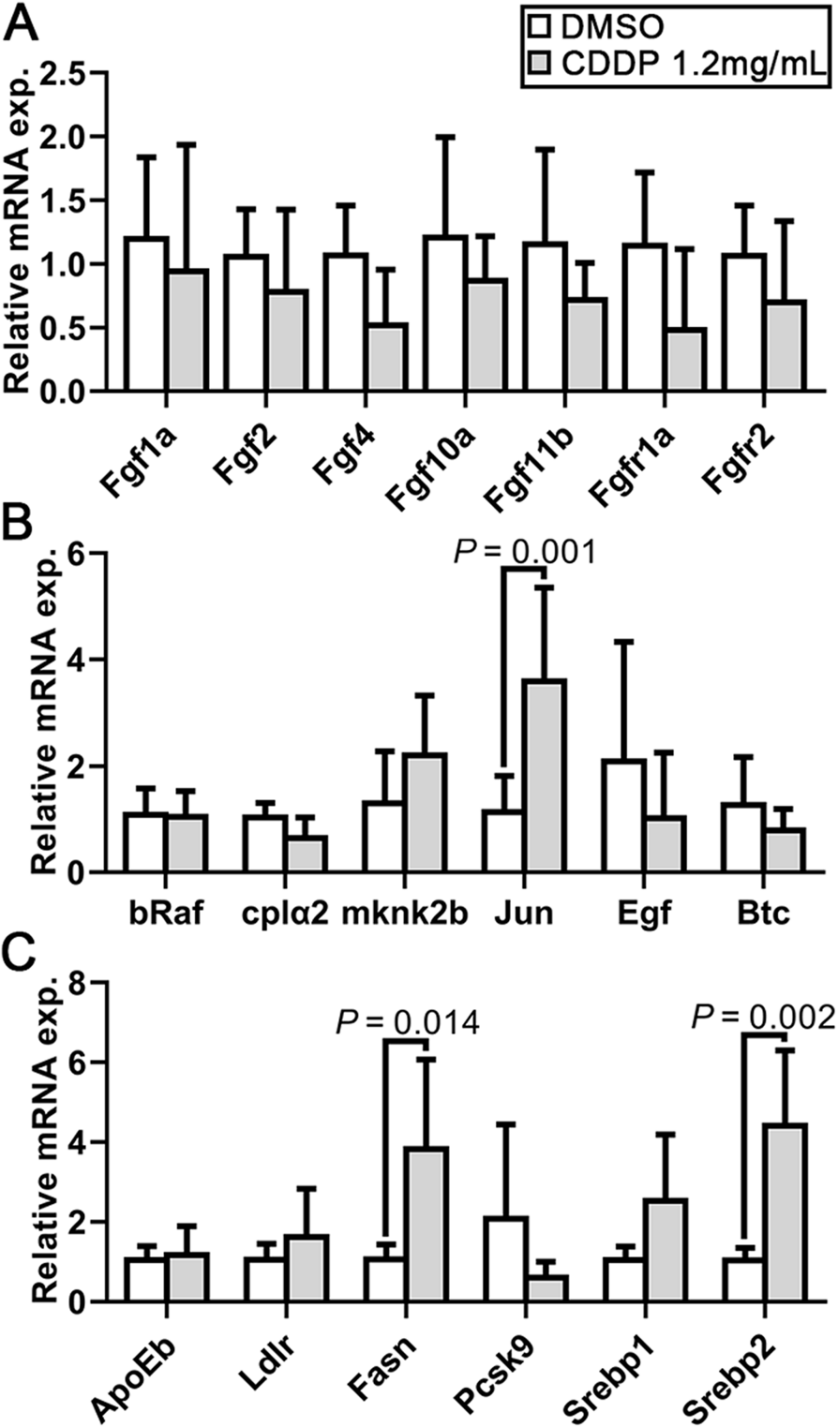
Analysis of *FGF*, *MAPK/ERK*, *SREBP 1* and *2* signaling pathways in CDDP-treated embryos. Expression levels were quantified using RT–qPCR and normalized against β-actin. Data are represented by relative fold changes ± SEM with respect to control embryos (arbitrarily set to 1). **A** Relative mRNA expression levels of *Fgf1a*, *Fgf2*, *Fgf4*, *Fgf10a* and *Fgf11b*, as well as the receptors *Fgfr1a* and *Fgfr2*. None were statistically changed. **B** The relative mRNA expression of *bRaf*, *cplα2*, *mknk2b*, *JUN*, *Egf* and *Btc* was also detected. Only *JUN* expression was significantly upregulated. **C** Relative mRNA expression levels of the *SREBP 1* and *2* signaling pathways showed that *Srebp2* might be activated and that as a downstream factor, *Fasn* expression was elevated. *N* = 6, and the data are presented as the mean ± SEM

Other pathways involved in angiogenesis were also detected. Previous studies have revealed the important role of the *FGF* signaling pathway in regulating angiogenesis in zebrafish [22]. However, examination of the expression of the key genes showed that the 1.2 mg/mL CDDP treatment exhibited no obvious regulation of the *FGF* pathway (Fig. 4A). However, both bRaf and MAP kinase interacting serine/threonine kinase 2 (Mknk2) play a role in regulating the *MAPK/ERK* signaling pathway, which might next regulate angiogenesis. Figure 4B shows no discernible difference in the relative mRNA expression levels of the genes involved in this pathway in CDDP-exposed zebrafish embryos, except *JUN*, a crucial target gene of the *MAPK/ERK* signaling pathway. The upregulation of *JUN* was consistent with the previous prediction in Fig. 1. Due to the reported effect of *Radix Salviae* on lipid metabolism, two key signaling pathways, *SREBP 1* and *2*, were further detected. The results showed that the *SREBP 2*, but not the *SREBP 1*, pathway was significantly activated. As a target gene of the *SREBP 2* pathway, *Fasn* was also upregulated (Fig. 4C).

### Proangiogenesis effect of CDDP in VRI-induced ISV deficiency in zebrafish

**Fig. 5:**
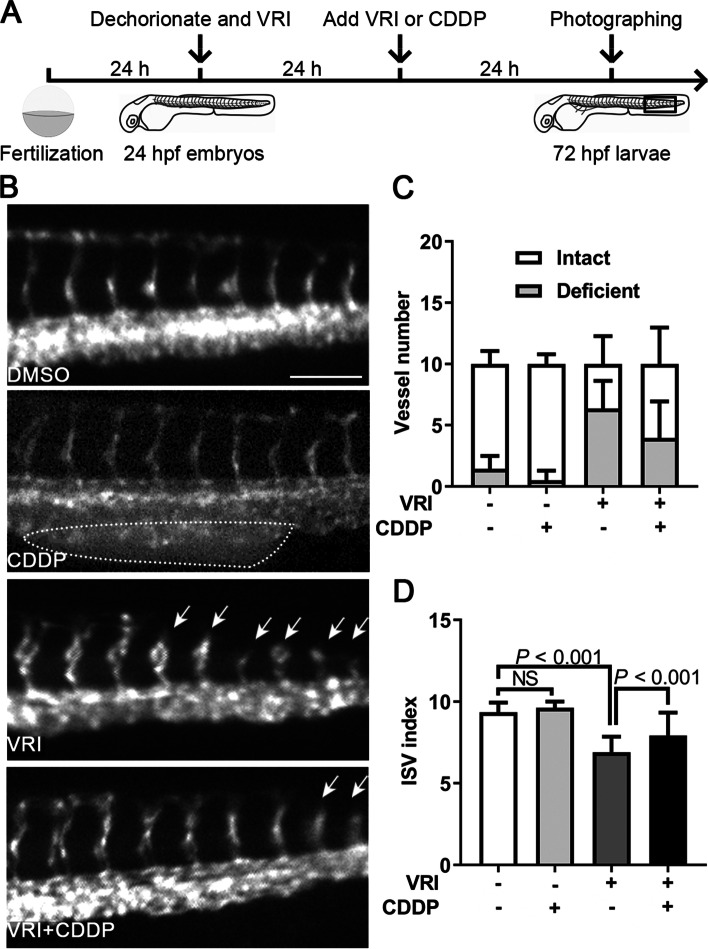
Pro-angiogenesis effect of CDDP in VRI-induced ISV deficiency in zebrafish.** A** Schematic representation of the experimental design. Twenty-four hpf *Tg(fli1:EGFP)* zebrafish embryos were pretreated with VRI (100 nM) for 24 h and then treated with 1.2 mg/mL CDDP or fresh VRI for another 24 h. Ten consecutive ISVs in the framed area were photographed, observed in each embryo, and then displayed in B. Fifteen embryos were included in each group, and the experiment was replicated three times. **B** Embryos treated with CDDP alone showed obvious enlarged posterior blood islands (PBIs, indicated by the dotted circle), whereas VRI treatment inhibited the development of ISVs, which could be restored by CDDP treatment. Arrows indicate deficient ISVs. Scale bar, 100 μm. **C** The numbers of intact and deficient vessels were calculated. **D** The ISV index was used to quantify the growth of ISVs, which was calculated using the formula *ISV index* = *(intact vessel number* × 1) + *(defective vessel number* × *0.5*). The results showed that there was no obvious defect in the development of ISVs in the CDDP and DMSO treatment groups, but the ISVs of the VRI-treated embryos were significantly shorter, and the VRI + CDDP treatment reversed the phenotype. *N* = 15, and the data are presented as the mean ± SEM

Lenvatinib is a specific VEGF receptor inhibitor (VRI) and can be used to establish a defective angiogenesis model in zebrafish [20]. In the present study, 24 hpf embryos were pretreated with VRI (100 nM) for 24 h and then treated with 1.2 mg/mL CDDP or fresh VRI for another 24 h (Fig. 5A). We found that CDDP treatment caused obvious enlargement of the posterior blood island (PBI), and that ISV growth was inhibited by VRI with decreased intact vessels and increased defective vessels (Fig. 5B-C). However, CDDP recovered the VRI-impaired ISV growth in zebrafish (Fig. 5B-C). The ISV index was used to quantify the growth of ISVs, which was calculated using the formula ISV index = (intact vessel number × 1) + (defective vessel number × 0.5). Consistent with the results above, VRI treatment significantly reduced the ISV index, whereas CDDP restored it (Fig. 5D).

### PI3K and AKT inhibitors reduced the proangiogenic effect of CDDP in zebrafish.

**Fig. 6:**
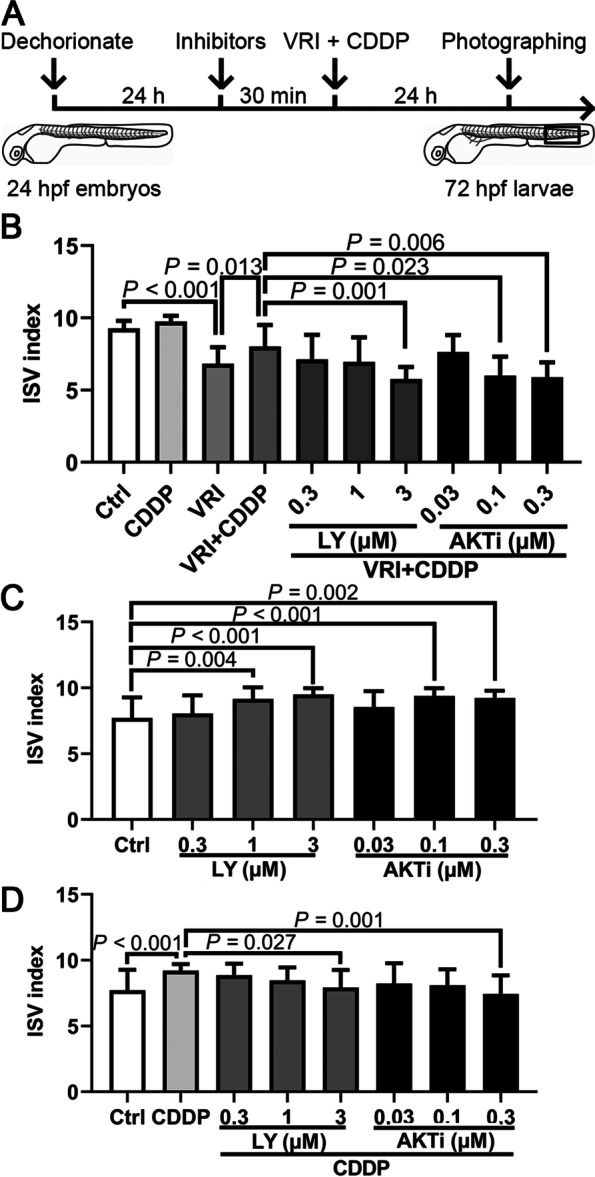
The effect of the *VEGF/PI3K/AKT* signaling pathway on the proangiogenic effect of CDDP. A Schematic representation of the experimental design. Twenty-four hpf *Tg(fli1:EGFP)* embryos were pretreated with various concentrations of the *PI3K* inhibitor LY294002 (LY, 0.3–3 µM) and the *AKT* inhibitor VIII (AKTi, 0.03–0.3 µM) for 30 min and were then cotreated with VRI (100 nM) and CDDP (1.2 mg/mL) for 24 h. Ten consecutive ISVs in the framed area were observed in each embryo. Fifteen embryos were analyzed in each group, and the experiment was replicated four times. **B** CDDP relieved the abnormal development of ISVs, which could be inhibited by 3 µM LY, 0.1 or 0.3 µM AKTi. **C** Twenty-four hpf embryos were treated with various concentrations of LY (0.3–3 µM) and AKTi (0.03–0.3 µM) for 24 h. Both inhibitors could promote angiogenesis of ISVs at specific concentrations. **D** The angiogenic efficacy of CDDP could be weakened by LY or AKTi at specific concentrations. The ISV index of each zebrafish embryo was calculated as (the number of intact vessels × 1) + (the number of defective vessels × 0.5). Data are presented as the mean ± SEM

It has been reported that the VEGF receptor activates the *PI3K/AKT* signaling pathway and promotes angiogenesis [20]. Our screening results above also suggested that the *VEGF/PI3K/AKT* signaling pathway probably affected angiogenesis, so we speculated that CDDP also promotes the angiogenesis process by activating the *VEGF/PI3K/AKT* signaling pathway. Based on this inference, because CDDP reversed the vascular development disorder caused by VRI, when the activation of the *PI3K/AKT* pathway was inhibited, the protective effect of CDDP on vascular development might also be blocked. Hence, the PI3K inhibitor LY (0.3–3 µM) and AKT inhibitor AKTi (0.03–0.3 µM) were employed to evaluate this prediction in vivo (Fig. 6A). Consistent with previous reports, we found that PI3K and AKT inhibitors reduced the proangiogenic effect of CDDP (1.2 mg/mL) in a VRI-induced angiogenesis deficiency model in a dose-dependent manner (Fig. 6B). Interestingly, the growth of ISVs in embryos treated with PI3K and AKT inhibitors but without CDDP or VRI was slightly stimulated (Fig. 6C). Moreover, PI3K and AKT inhibitors hindered the proangiogenic effect of CDDP even without VRI pretreatment (Fig. 6D).

## Discussion

As a novel field that integrates bioinformatics with pharmacology, biochemistry, and genomics [23], network pharmacology is an extraordinary tool to visualize and analyze complex interactive data on herbs, compounds, targets, and diseases based on computer modeling analysis and target prediction methods [24] and is particularly suitable for characterizing TCM formulations. For example, CDDP is a TCM formula with complex components. There may be a variety of synergistic effects among its active ingredients, which play a role in the treatment of CHD. Therefore, it is impractical to extract and study the effective compounds it contains one by one. In this study, we took advantage of network pharmacology and predicted the compounds contained in the two main components of CDDP, *Radix Salviae* and *Panax notoginseng*, and their regulatory targets. We compared these target genes with the reported genes related to CHD and screened 65 potential targets of CDDP in the treatment of CHD (Fig. 1A). Further studies showed that CDDP might indeed promote angiogenesis because some key genes and signaling pathways in the process of angiogenesis were predicted to be targets of CDDP, such as the *VEGFA* and *PI3K/AKT* pathways (Fig. 1). VEGFA binds to the VEGF receptor (VEGFR), particularly VEGFR2 (Kdrl), and then mediates downstream pathways to regulate angiogenesis. The *PI3K/AKT* signaling pathway was also suggested to regulate endothelial cell proliferation, migration, and invasion, therefore promoting angiogenesis [20].

We then employed the transgenic zebrafish line *Tg(fli1:EGFP)*, which expresses enhanced fluorescent protein in vascular endothelial cells, to test whether CDDP actually has proangiogenic activity in vivo and in the involved pathways. This animal model, with the advantages of high genetic similarity to humans and low cost, was convenient for vascular system observation under a fluorescence microscope. We found that CDDP did not affect the survival rate or early global development below a concentration of 1.2 mg/mL (Fig. 2A-E), which indicates low lethal toxicity. When focused on the vascular system, we noticed that CDDP significantly promoted SIV branching and sprouting (Fig. 2G-I). Simultaneously, OA demonstrated a thickened endothelial layer (Fig. 3A-E) and ectopic development (Fig. 3F-G) after CDDP treatment. The RT–qPCR assays revealed that the *VEGF/VEGFR* signaling pathway was affected (Fig. 3J). These results indicate that CDDP promotes SIV and OA growth in zebrafish.

Several reported key genes and pathways affecting angiogenesis such as the *FGF* and *MAPK/ERK* pathways were detected (Fig. 4A-B). However, their expression was largely unchanged after CDDP treatment, except *JUN*, which was also predicted to be activated by CDDP, as shown in Fig. 1. This might be another target gene of CDDP in CHD treatment and is worthy of further study. Notably, the lipid-metabolism-related *SREBP2* pathway was upregulated (Fig. 4C). This indicated that CDDP might have a lipid-lowering effect, and this might be another potential mechanism in CHD treatment. Further confirmation was achieved through inhibitor-induced ISV injury experiments. The enlarged PBI that we observed in CDDP-treated embryos suggested that CDDP may also promote the differentiation and proliferation of the hematopoietic endothelium. Therefore, CDDP might also promote early hematopoiesis in zebrafish embryos, which suggests that it could also be a potential drug for the treatment of blood system diseases, which is worthy of further exploration. Our results showed that VRI significantly impaired ISV sprouting, whereas CDDP could rescue the phenotype (Fig. 5). We have proven through in vivo experiments and *VEGF/VEGFR* pathway gene expression analysis that CDDP activates this pathway to promote angiogenesis. As a well-known and widely studied pathway involved in angiogenesis across a variety of vertebrates, *VEGF/VEGFR* signaling was reported to regulate various downstream targets. Fan *et al.* reported that Dalbergia odorifera extract has a proangiogenic effect, and its mechanism could be *VEGF/VEGFR* mRNA regulation and *PI3K/MAPK* signaling pathway activation [20]. Because the* PI3K/AKT* signaling pathway was also predicted by our network pharmacological study to be a potential key target pathway of CDDP in the treatment of CHD, we further tested whether the *PI3K/AKT* signaling pathway played a role downstream of the CDDP promotion of the angiogenesis process. Consistent with our speculation, when cotreated with LY or AKTi, the restoration of ISV growth induced by CDDP was clearly inhibited (Fig. 6B), which proved that *PI3K/AKT* might be the potential pathway of CDDP’s proangiogenic effect. In brief, we hypothesize that CDDP activates PI3K, a downstream molecule of the *VEGF/VEGFR* pathway, by increasing the expression of *VEGFA* and *Kdrl*, thereby increasing the phosphorylation of AKT1 and exerting transcriptional regulation to promote vascular endothelial proliferation and angiogenesis (Supplemental Fig. S1). Interestingly, when treated with LY or AKTi alone, ISV growth was slightly enhanced (Fig. 6C). This might be because angiogenesis is regulated by multiple signaling pathways. When *PI3K/AKT* alone was inhibited, some other pathways may be regulated in vivo to some extent, maintaining a physiological balance of angiogenesis. Direct treatment with CDDP also promoted ISV development, which could be diminished by LY or AKTi cotreatment (Fig. 6D). These results further proved that CDDP might be used to treat CHD through *VEGF/VEGFR* and *PI3K/AKT* pathway-mediated angiogenesis.

However, our study still has many limitations. For example, fold changes of the genes and pathways mentioned in this manuscript was not further detected on the protein level due to the lack of specific antibodies against zebrafish. Hence, our results could provide a reference for TCM researchers and clinicians but still need further verification

## Conclusions

In conclusion, our study demonstrates that CDDP, a patented TCM formula, promotes angiogenic effects in zebrafish embryos through the activation of the *VEGF/VEGFR* and *PI3K/AKT * signaling pathways. Our results provide new evidence for the application of CDDP in the treatment of CHD.

## Supplementary Information

**Additional file 1. Fig. S1**. CDDP promotes sprouting angiogenesis. Schematic diagram of CDDP promoting angiogenesis by activating the VEGF/VEGFR and PI3K/AKT signaling pathways.

**Additional file 2. Table 1.** Composition of CDDP. The main 2 components ofCDDP are listed in the table. 

**Additional file 3. Table 2. **Primers usedfor RT–qPCR in this study. The primers used in this study are listed in thetable.

**Additional file 4.** **Table 3. **Pharmacokinetic parameters of theingredients reported in *Radix Salviae*. The 65 ingredients and their pharmacokinetic parameters in *RadixSalviae* were obtained from the online database TCMSP.

**Additional file 5. Table 4. **Pharmacokinetic parameters of theingredients reported for *Panax notoginseng*. The 8 ingredients and their pharmacokinetic parameters in *Panaxnotoginseng* were obtained from the online database TCMSP.

**Additional file 6. Table 5. **Genes related to active ingredients of* Radix Salviae*. The screening of ingredient-related genes of *Radix Salviae* wasperformed using TCMSP and confirmed through the DrugBank database 

**Additional file 7.** **Table 6. **Genes related to the active ingredients of* Panax notoginseng*. The screening of ingredient-related genes of *Panax notoginseng*was performed using TCMSP and confirmed through the DrugBank database.

**Additional file 8.** **Table 7.** Key genes related to coronary heart disease.The 7,100 coronary heart disease-related genes reported were screened outthrough the GeneCards database

**Additional file 9.** **Table 8. **Screenedtarget genes of CDDP for CHD treatment. In total, 65 potential target genes of CDDP forCHD treatment were identified.

## Data Availability

The datasets used and/or analyzed during the current study are available from the corresponding author on reasonable request.

## Abbreviations

AKT: Protein kinase B
AKT1: V-AKT murine thymoma viral oncogene homologue 1
AKTi: AKT inhibitor VIII
ANOVA: Analysis of variance
AP: Angina pectoris
bRaf: B-Raf proto-oncogene, serine/threonine kinase
CDDP: Compound Danshen Dripping Pill
CFDA: China Food and Drug Administration
CHD: Coronary heart disease
CMD: Cardiac microvascular disorder
CVD: Cardiovascular disease
Di: Inner diameter
DL: Drug-likeness
DMSO: Dimethyl sulphoxide
Do: Outer diameter
EC: Endothelial cell
EGFP: Enhanced green fluorescent protein
ERK: Extracellular regulated protein kinase
Fasn: Fatty acid synthase
FDA: US Food and Drug Administration
FGF: Fibroblast growth factor
Flt1: Fms related receptor tyrosine kinase 1
Flt4: Fms-related tyrosine kinase 4
GO: Gene ontology
hpf: Hours post fertilization
ISV: Intersegmental vessel
UN: Jun proto-oncogene
AP-1: Transcription factor subunit
KEGG: Kyoto Encyclopedia of Genes and Genomes
Kdrl: Vascular endothelial growth factor receptor kdr-like
LC50: The median lethal concentration;
LY: LY294002
MAPK: Mitogen-activated protein kinase
Mknk2: MAP kinase interacting serine/threonine kinase 2
OA: Optic artery
OB: Oral bioavailability
PI3K: Phosphatidylinositol 3-kinase
PPI: Protein–protein interaction
PTU: 2-Phenylthiourea;
rt-qPCR: Real-time quantitative PCR
SEM: Standard error of mean
SIV: Sub-intestinal vessel plexus
SREBP: Sterol regulatory element binding protein
T: Thickness of endothelial layer
TCM: Traditional Chinese medicine
TCMSP: Traditional Chinese Medicine Systems Pharmacology Database and Analysis Platform
VEGF: Vascular endothelial growth factor
VEGFA: Vascular endothelial growth factor A
VEGFR: Vascular endothelial growth factor receptor
VRI: VEGF receptor kinase inhibitor Lenvatinib
WT: Wild-type; w/v, weight/volume
