# Supplementary material for: Proangiogenesis effects of compound danshen dripping pills in zebrafish
Source: BMC Complement Med Ther. 2022 Apr 22;22:112. doi: 10.1186/s12906-022-03589-y (PMC9034551; doi:10.1186/s12906-022-03589-y)
Supplement: Supplementary file 1 — Additional file 1. Fig. S1. CDDP promotes sprouting angiogenesis. Schematic diagram of CDDP promoting angiogenesis by activating the VEGF/VEGFR and PI3K/AKT signaling pathways. [file 12906_2022_3589_MOESM1_ESM.docx]

**
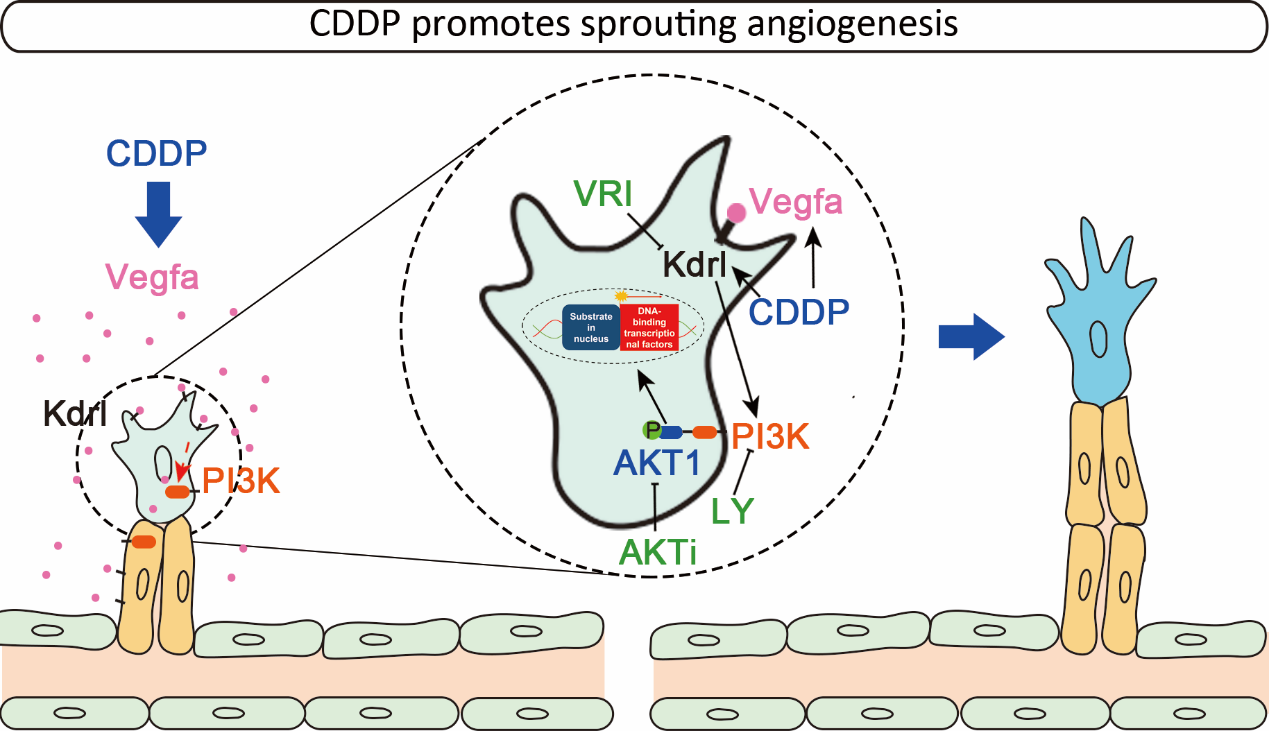
**

**Supplementary Figure S1. CDDP promotes sprouting angiogenesis.** Schematic diagram of CDDP promoting angiogenesis by activating *VEGF/VEGFR* and *PI3K/AKT* signaling pathways. CDDP increases the expression of VEGFA and Kdrl, activating PI3K, a downstream molecule of the *VEGF/VEGFR* pathway, thereby increases the phosphorylation of AKT1, exerting transcriptional regulation to promote vascular endothelial proliferation and angiogenesis.
