# Supplementary material for: Proangiogenesis effects of compound danshen dripping pills in zebrafish
Source: BMC Complement Med Ther. 2022 Apr 22;22:112. doi: 10.1186/s12906-022-03589-y (PMC9034551; doi:10.1186/s12906-022-03589-y)
Supplement: Supplementary file 2 — Additional file 2. Table 1. Composition of CDDP. The main 2 components ofCDDP are listed in the table. [file 12906_2022_3589_MOESM2_ESM.docx]

**Supplementary Table 1.** Composition of CDDP.

| **Chinese name** | **Latin name** | **English name** | **Weight (g)** |
| --- | --- | --- | --- |
| Danshen | *Salvia miltiorrhiza* Bunge | *Radix Salviae* | 10 |
| Sanqi | *Panax Notoginseng* (Burk.) F. H. Chen Ex C. Chow | *Panax Notoginseng* | 10 |
