# Supplementary material for: Proangiogenesis effects of compound danshen dripping pills in zebrafish
Source: BMC Complement Med Ther. 2022 Apr 22;22:112. doi: 10.1186/s12906-022-03589-y (PMC9034551; doi:10.1186/s12906-022-03589-y)
Supplement: Supplementary file 3 — Additional file 3. Table 2. Primers usedfor RT–qPCR in this study. The primers used in this study are listed in thetable. [file 12906_2022_3589_MOESM3_ESM.docx]

**Supplementary Table 2.** Primers used for RT–qPCR in this study.

| **Gene symbol** | **Forward primer (5’-3’)** | **Reverse primer (5’-3’)** |
| --- | --- | --- |
| *Vegfaa* | cagctgtcaagagtgcctacatac | catcagggtactcctgctgaatttc |
| *Flt1* | aactcacagaccagtgaacaagatc | gccctgtaacgtgtgcactaaa |
| *Kdrl* | gaccataaaacaagtgaggcagaag | ctcctggtttgacagagcgata |
| *Flt4* | aggacgatggcttctcacaa | accatcccactgtctgtctg |
| *Fgf1a* | aggagtggtggtgatcgaag | ccccgtttcattttcccgtt |
| *Fgf2* | ggaggaaaaccactacaacac | acctgtcgtggaagaaagaaaatgg |
| *Fgf4* | ccggcgtacacaacgaaaac | gaactgctcagatccgtaaagc |
| *Fgfr1a* | gaatacgagcttccccagga | gtggcatccgatttgagcat |
| *Fgfr2* | agaagaggacgctggtgaaa | tcttagccgacgtcctcatc |
| *Fgfr10a* | cgatccgtacagtacactcgaaa | agcttgcagtcaatgccgaa |
| *Fgfrl1b* | ccggacgggtcatatctcaa | ctgggatgccaatgatcagc |
| *Braf* | ttacagctcccactccacag | atgatgtgcaggtggtggta |
| *Cplα2* | gcctccccttactccacttt | agctgtctgttaggtgggtc |
| *Mknk2b* | atggcccacagagacttgaa | cacaggtcacagcgcttatc |
| *Jun* | gcgtgccgttaaaggatgat | gtcgcgtccctgttttactc |
| *Egf* | ggcaaggcaaagaactggaa | tctacaaggctcctctccct |
| *Btc* | ctatgcaaacactcccaggc | tgctcctctatgaaacggca |
| *Apoeb* | tggaccgtttctggcagtat | gggtcatctgggtttggaga |
| *Ldlr* | aattcacctgtaccgcctga | cacccacaaccaaacagagg |
| *Fasn* | gctgacgttcttatggctgg | tctgttcctgttcgatggct |
| *Pcsk9* | gggtactgtgtctggagctt | actgtgatcacctctggctc |
| *Srebp1* | ttgtggatgaggactggacc | gctcttgtctcctctgggtt |
| *Srebp2* | ctgacgaactgcagcatctc | atgctgtgagtgaacgagga |
| *Hmgcr* | tgccccaaactagatgagca | attgaggcctgtcagctctt |
| *β-actin* | cgagcaggagatgggaacc | caacggaaacgctcattgc |
| *Dusp6* | tctatctcgagggtggcttca | actcgatgtccgaggagtca |
| *Pgfa* | agtgaagccccagagatgac | aaatcttcatgtcgagcggc |
| *Pgfb* | ctattcgggcggactactgt | cccaaacctcctgaaacagc |
