# Supplementary material for: Proangiogenesis effects of compound danshen dripping pills in zebrafish
Source: BMC Complement Med Ther. 2022 Apr 22;22:112. doi: 10.1186/s12906-022-03589-y (PMC9034551; doi:10.1186/s12906-022-03589-y)
Supplement: Supplementary file 4 — Additional file 4. Table 3. Pharmacokinetic parameters of theingredients reported in Radix Salviae. The 65 ingredients and their pharmacokinetic parameters in RadixSalviae were obtained from the online database TCMSP. [file 12906_2022_3589_MOESM4_ESM.docx]

**Supplementary Table 3.** Pharmacokinetic parameters of the ingredients reported in *Radix Salviae*.

| **Mol ID** | **Molecule name** | **MW** | **OB (%)** | **DL** | **BBB** | **HL** |
| --- | --- | --- | --- | --- | --- | --- |
| MOL001601 | 1,2,5,6-tetrahydrotanshinone | 280.34 | 38.75 | 0.36 | 0.39 | 18.05 |
| MOL001942 | isoimperatorin | 270.30 | 45.46 | 0.23 | 0.66 | -1.44 |
| MOL002651 | Dehydrotanshinone II A | 292.35 | 43.76 | 0.40 | 0.52 | 23.71 |
| MOL007041 | 2-isopropyl-8-methylphenanthrene-3,4-dione | 264.34 | 40.86 | 0.23 | 0.81 | 14.89 |
| MOL007049 | 4-methylenemiltirone | 266.36 | 34.35 | 0.23 | 0.87 | 14.60 |
| MOL007058 | formyltanshinone | 290.28 | 73.44 | 0.42 | -0.28 | 24.12 |
| MOL007061 | Methylenetanshinquinone | 278.32 | 37.07 | 0.36 | 0.46 | 24.33 |
| MOL007079 | tanshinaldehyde | 308.35 | 52.47 | 0.45 | -0.07 | 23.49 |
| MOL007085 | Salvilenone | 292.40 | 30.38 | 0.38 | 1.07 | 20.81 |
| MOL007088 | cryptotanshinone | 296.39 | 52.34 | 0.40 | 0.51 | 17.30 |
| MOL007094 | danshenspiroketallactone | 282.36 | 50.43 | 0.31 | 0.51 | 15.19 |
| MOL007100 | dihydrotanshinlactone | 266.31 | 38.68 | 0.32 | 0.81 | 5.42 |
| MOL007101 | dihydrotanshinoneⅠ | 278.32 | 45.04 | 0.36 | 0.43 | 18.32 |
| MOL007105 | epidanshenspiroketallactone | 284.38 | 68.27 | 0.31 | 0.61 | 1.77 |
| MOL007108 | isocryptotanshi-none | 296.39 | 54.98 | 0.39 | 0.34 | 31.92 |
| MOL007111 | Isotanshinone II | 294.37 | 49.92 | 0.40 | 0.45 | 24.73 |
| MOL007122 | Miltirone | 282.41 | 38.76 | 0.25 | 0.87 | 14.82 |
| MOL007127 | 1-methyl-8,9-dihydro-7H-naphtho[5,6-g]benzofuran-6,10,11-trione | 280.29 | 34.72 | 0.37 | -0.27 | 37.89 |
| MOL007154 | tanshinone iia | 294.37 | 49.89 | 0.40 | 0.70 | 23.56 |
| MOL001659 | Poriferasterol | 412.77 | 43.83 | 0.76 | 1.03 | 5.34 |
| MOL001771 | poriferast-5-en-3beta-ol | 414.79 | 36.91 | 0.75 | 1.14 | 5.07 |
| MOL002222 | sugiol | 300.48 | 36.11 | 0.28 | 0.70 | 14.62 |
| MOL006824 | α-amyrin | 426.80 | 39.51 | 0.76 | 1.20 | 3.06 |
| MOL007045 | 3α-hydroxytanshinoneⅡa | 310.37 | 44.93 | 0.44 | 0.22 | 23.78 |
| MOL007059 | 3-beta-Hydroxymethyllenetanshiquinone | 294.32 | 32.16 | 0.41 | -0.48 | 22.51 |
| MOL007063 | przewalskin a | 398.49 | 37.11 | 0.65 | -0.69 | 1.63 |
| MOL007064 | przewalskin b | 330.46 | 110.32 | 0.44 | 0.22 | 2.17 |
| MOL007068 | Przewaquinone B | 292.30 | 62.24 | 0.41 | -0.45 | 24.94 |
| MOL007069 | przewaquinone c | 296.34 | 55.74 | 0.40 | -0.30 | 23.70 |
| MOL007081 | Danshenol B | 354.48 | 57.95 | 0.56 | 0.11 | 4.28 |
| MOL007082 | Danshenol A | 336.41 | 56.97 | 0.52 | -0.01 | 5.15 |
| MOL007093 | dan-shexinkum d | 336.41 | 38.88 | 0.55 | -0.15 | 30.00 |
| MOL007098 | deoxyneocryptotanshinone | 298.41 | 49.40 | 0.29 | 0.24 | 27.17 |
| MOL007107 | C09092 | 286.50 | 36.07 | 0.25 | 1.54 | -0.16 |
| MOL007115 | manool | 304.57 | 45.04 | 0.20 | 1.16 | 5.81 |
| MOL007118 | microstegiol | 298.46 | 39.61 | 0.28 | 0.99 | 4.52 |
| MOL007119 | miltionone Ⅰ | 312.39 | 49.68 | 0.32 | -0.11 | 41.49 |
| MOL007120 | miltionone Ⅱ | 312.39 | 71.03 | 0.44 | 0.03 | 2.91 |
| MOL007121 | miltipolone | 300.43 | 36.56 | 0.37 | 0.17 | 1.70 |
| MOL007123 | miltirone Ⅱ | 272.32 | 44.95 | 0.24 | -0.25 | 2.24 |
| MOL007124 | neocryptotanshinone ii | 270.35 | 39.46 | 0.23 | 0.16 | 26.98 |
| MOL007143 | salvilenone Ⅰ | 270.40 | 32.43 | 0.23 | 0.77 | 1.00 |
| MOL007145 | salviolone | 268.38 | 31.72 | 0.24 | 0.72 | 0.33 |
| MOL007149 | NSC 122421 | 300.48 | 34.49 | 0.28 | 0.63 | 14.56 |
| MOL007155 | (6S)-6-(hydroxymethyl)-1,6-dimethyl-8,9-dihydro-7H-naphtho[8,7-g]benzofuran-10,11-dione | 310.37 | 65.26 | 0.45 | -0.31 | 23.48 |
| MOL007036 | 5,6-dihydroxy-7-isopropyl-1,1-dimethyl-2,3-dihydrophenanthren-4-one | 298.41 | 33.77 | 0.29 | 0.80 | 14.91 |
| MOL007050 | 2-(4-hydroxy-3-methoxyphenyl)-5-(3-hydroxypropyl)-7-methoxy-3-benzofurancarboxaldehyde | 356.40 | 62.78 | 0.40 | -0.73 | 7.89 |
| MOL007070 | (6S,7R)-6,7-dihydroxy-1,6-dimethyl-8,9-dihydro-7H-naphtho[8,7-g]benzofuran-10,11-dione | 312.34 | 41.31 | 0.45 | -0.68 | 22.54 |
| MOL007071 | przewaquinone f | 312.34 | 40.31 | 0.46 | -0.90 | 22.45 |
| MOL007077 | sclareol | 308.56 | 43.67 | 0.21 | 0.51 | 4.71 |
| MOL007125 | neocryptotanshinone | 314.41 | 52.49 | 0.32 | -0.13 | 14.46 |
| MOL007150 | (6S)-6-hydroxy-1-methyl-6-methylol-8,9-dihydro-7H-naphtho[8,7-g]benzofuran-10,11-quinone | 312.34 | 75.39 | 0.46 | -0.74 | 23.45 |
| MOL007151 | Tanshindiol B | 312.34 | 42.67 | 0.45 | -0.63 | 22.25 |
| MOL007152 | Przewaquinone E | 312.34 | 42.85 | 0.45 | -0.65 | 22.44 |
| MOL007156 | tanshinone Ⅵ | 296.34 | 45.64 | 0.30 | -0.28 | 15.21 |
| MOL000006 | luteolin | 286.25 | 36.16 | 0.25 | -0.84 | 15.94 |
| MOL007048 | (E)-3-[2-(3,4-dihydroxyphenyl)-7-hydroxy-benzofuran-4-yl]acrylic acid | 312.29 | 48.24 | 0.31 | -0.89 | 8.87 |
| MOL007130 | prolithospermic acid | 314.31 | 64.37 | 0.31 | -0.75 | 8.82 |
| MOL007141 | salvianolic acid g | 340.30 | 45.56 | 0.61 | -0.97 | 2.40 |
| MOL007051 | 6-o-syringyl-8-o-acetyl shanzhiside methyl ester | 628.64 | 46.69 | 0.71 | -2.08 | 9.94 |
| MOL007132 | (2R)-3-(3,4-dihydroxyphenyl)-2-[(Z)-3-(3,4-dihydroxyphenyl)acryloyl]oxy-propionic acid | 360.34 | 109.38 | 0.35 | -1.02 | 2.01 |
| MOL007140 | (Z)-3-[2-[(E)-2-(3,4-dihydroxyphenyl)vinyl]-3,4-dihydroxy-phenyl]acrylic acid | 314.31 | 88.54 | 0.26 | -0.77 | 4.31 |
| MOL002776 | Baicalin | 446.39 | 40.12 | 0.75 | -1.74 | 17.36 |
| MOL000569 | digallate | 322.24 | 61.85 | 0.26 | -1.52 | 5.29 |
| MOL007142 | salvianolic acid j | 538.49 | 43.38 | 0.72 | -2.14 | 5.77 |

Abbreviations: MW, relative molecular mass; OB, oral bioavailability; DL, drug-likeness; BBB, blood–brain barrier; HL, half-life.
