# Supplementary material for: Proangiogenesis effects of compound danshen dripping pills in zebrafish
Source: BMC Complement Med Ther. 2022 Apr 22;22:112. doi: 10.1186/s12906-022-03589-y (PMC9034551; doi:10.1186/s12906-022-03589-y)
Supplement: Supplementary file 5 — Additional file 5. Table 4. Pharmacokinetic parameters of theingredients reported for Panax notoginseng. The 8 ingredients and their pharmacokinetic parameters in Panaxnotoginseng were obtained from the online database TCMSP. [file 12906_2022_3589_MOESM5_ESM.docx]

**Supplementary Table 4.** Pharmacokinetic parameters of the ingredients reported for *Panax notoginseng*.

| **Mol ID** | **Molecule name** | **MW** | **OB (%)** | **DL** | **BBB** | **HL** |
| --- | --- | --- | --- | --- | --- | --- |
| MOL001494 | Mandenol | 308.56 | 42.00 | 0.19 | 1.14 | 5.39 |
| MOL001792 | [DFV](https://tcmspw.com/molecule.php?qn=1792) | 256.27 | 32.76 | 0.18 | -0.29 | 17.89 |
| MOL002879 | [Diop](https://tcmspw.com/molecule.php?qn=2879) | 390.62 | 43.59 | 0.39 | 0.26 | 3.60 |
| MOL000358 | [beta-sitosterol](https://tcmspw.com/molecule.php?qn=358) | 414.79 | 36.91 | 0.75 | 0.99 | 5.36 |
| MOL000449 | [Stigmasterol](https://tcmspw.com/molecule.php?qn=449) | 412.77 | 43.83 | 0.76 | 1.00 | 5.57 |
| MOL005344 | [ginsenoside rh2](https://tcmspw.com/molecule.php?qn=5344) | 622.98 | 36.32 | 0.56 | -1.38 | 11.08 |
| MOL007475 | [ginsenoside f2](https://tcmspw.com/molecule.php?qn=7475) | 785.14 | 36.43 | 0.25 | -3.03 | 13.11 |
| MOL000098 | quercetin | 302.25 | 46.43 | 0.28 | -0.77 | 14.40 |

Abbreviations: MW, relative molecular mass; OB, oral bioavailability; DL, drug-likeness; BBB, blood–brain barrier; HL, half-life.
