# Supplementary material for: Proangiogenesis effects of compound danshen dripping pills in zebrafish
Source: BMC Complement Med Ther. 2022 Apr 22;22:112. doi: 10.1186/s12906-022-03589-y (PMC9034551; doi:10.1186/s12906-022-03589-y)
Supplement: Supplementary file 6 — Additional file 6. Table 5. Genes related to active ingredients of Radix Salviae. The screening of ingredient-related genes of Radix Salviae wasperformed using TCMSP and confirmed through the DrugBank database [file 12906_2022_3589_MOESM6_ESM.docx]

**Supplementary Table 5.** Genes related to active ingredients of *Radix Salviae*.

| **Id** | **Ingredients** | **Target genes** |
| --- | --- | --- |
| MOL001601 | 1,2,5,6-tetrahydrotanshinone | PTGS1; CHRM3; CHRM1; SCN5A; CHRM5; PTGS2; HTR3A; CHRM4; RXRA; OPRD1; ADRA1A; CHRM2; ADRA1B; SLC6A3; ADRB2; ADRA1D; OPRM1; GABRA1; NCOA2; NCOA1; SLC6A4 |
| MOL001659 | Poriferasterol | PGR; NR3C2 |
| MOL001771 | Poriferast-5-en-3beta-ol | PGR; NCOA2 |
| MOL001942 | Isoimperatorin | PTGS2 |
| MOL002222 | Sugiol | CHRM3; CHRM1; SCN5A; CHRM5; PTGS2; CHRM4; OPRD1; ACHE; ADRA1A; CHRM2; ADRA1B; ADRB2; ADRA1D; DRD2; OPRM1 |
| MOL002651 | Dehydrotanshinone II A | CHRM3; CHRM1; ESR1; AR; SCN5A; PPARG; CHRM5; PTGS2; CHRM4; OPRD1; ACHE; ADRA1A; ADRB2; OPRM1; GABRA1; NCOA1 |
| MOL000569 | Digallate | PTGS2; AKR1B1 |
| MOL000006 | Luteolin | PTGS1; AR; PTGS2; PRSS1; NCOA2; RELA; EGFR; AKT1; VEGFA; CCND1; BCL2L1; CDKN1A; CASP9; MMP2; MMP9; MAPK1; IL10; RB1; TNFSF15; JUN; IL6; CASP3; TP63; NFKBIA; TOP1; MDM2; APP; MMP1; PCNA; ERBB2; PPARG; HMOX1; CASP7; ICAM1; MCL1; BIRC5; IL2; CCNB1; TYR; IFNG; IL4; TOP2A; GSTP1; SLC2A4; INSR; CD40LG; PTGES; NUF2; ADCY2; MET |
| MOL007036 | 5,6-dihydroxy-7-isopropyl-1,1-dimethyl-2,3-dihydrophenanthren-4-one | PTGS1; CHRM3; CHRM1; SCN5A; PTGS2; RXRA; ACHE; ADRA1A; ADRA1B; ADRB2; OPRM1; NCOA2; NCOA1 |
| MOL007041 | 2-isopropyl-8-methylphenanthrene-3,4-dione | PTGS1; CHRM3; CHRM1; ESR1; AR; SCN5A; PPARG; CHRM5; PTGS2; HTR3A; CHRM4; RXRA; ADRA1A; CHRM2; ADRA1B; SLC6A3; ADRB2; ADRA1D; SLC6A4; OPRM1; GABRA1; CCNA2; NCOA2 |
| MOL007045 | 3α-hydroxytanshinoneⅡa | CHRM1; SCN5A; CHRM5; PTGS2; OPRD1; ACHE; ADRB2; OPRM1; PRSS1; NCOA1 |
| MOL007048 | (E)-3-[2-(3,4-dihydroxyphenyl)-7-hydroxy-benzofuran-4-yl]acrylic acid | PTGS2 |
| MOL007049 | 4-methylenemiltirone | PTGS1; CHRM3; CHRM1; ESR1; AR; SCN5A; PPARG; CHRM5; PTGS2; ADRA2A; ADRA2C; CHRM4; RXRA; OPRD1; ADRA1A; CHRM2; ADRA1B; SLC6A3; ADRB2; ADRA1D; SLC6A4; DRD2; OPRM1; GABRA1; NCOA2; NCOA1 |
| MOL007050 | 2-(4-hydroxy-3-methoxyphenyl)-5-(3-hydroxypropyl)-7-methoxy-3-benzofurancarboxaldehyde | NOS2; ESR1; AR; PPARG; ESR2; MAPK14; GSK3B; CCNA2 |
| MOL007058 | Formyltanshinone | AR; PTGS2; RXRA; NCOA1 |
| MOL007059 | 3-beta-Hydroxymethyllenetanshiquinone | CHRM1; PTGS2; RXRA; OPRD1; ACHE; ADRA1A; ADRB2; OPRM1; PRSS1; NCOA1 |
| MOL007061 | Methylenetanshinquinone | CHRM3; CHRM1; SCN5A; CHRM5; PTGS2; RXRA; OPRD1; ACHE; ADRA1A; CHRM2; ADRB2; SLC6A4; OPRM1; GABRA1; PRSS1; NCOA1 |
| MOL007063 | Przewalskin a | NR3C2; NR3C1 |
| MOL007064 | Przewalskin b | PTGS2; PGR; NR3C2; NR3C1; NCOA2; NCOA1 |
| MOL007068 | Przewaquinone B | PTGS2; RXRA; PRSS1; NCOA1 |
| MOL007069 | Przewaquinone c | PTGS1; CHRM3; CHRM1; SCN5A; CHRM5; PTGS2; CHRM4; OPRD1; ACHE; ADRA1A; CHRM2; ADRB2; OPRM1; GABRA1; NCOA1 |
| MOL007070 | (6S,7R)-6,7-dihydroxy-1,6-dimethyl-8,9-dihydro-7H-naphtho[8,7-g]benzofuran-10,11-dione | PTGS2; ACHE; PRSS1; NCOA1 |
| MOL007071 | Przewaquinone f | PTGS2; PRSS1; NCOA1 |
| MOL007077 | Sclareol | PTGS2 |
| MOL007079 | Tanshinaldehyde | CHRM1; PTGS2; OPRD1; ACHE; ADRB2; OPRM1; PRSS1; NCOA1 |
| MOL007081 | Danshenol B | PTGS2; PGR; OPRM1; NR3C1; NCOA1 |
| MOL007082 | Danshenol A | PTGS1; KCNH2; SCN5A; PTGS2; RXRA; NCOA1 |
| MOL007085 | Salvilenone | PTGS1; ESR1; AR; CHRM5; PTGS2; HTR3A; ESR2 |
| MOL007088 | Cryptotanshinone | PTGS1; CHRM3; CHRM1; SCN5A; CHRM5; PTGS2; CHRM4; OPRD1; ADRA1A; CHRM2; ADRA1B; ADRB2; ADRA1D; OPRM1; NCOA2; NCOA1; PGR; GABRA1; RELA; STAT3; CCND1; BCL2L1; TNFSF15; APP; EDN3; BIRC5 |
| MOL007093 | Dan-shexinkum d | NOS2; PTGS1; KCNH2; CHRM1; ESR1; AR; SCN5A; PPARG; PTGS2; RXRA; ACHE; ADRA1B; ADRB2; ESR2; GSK3B; CHEK1; PRSS1; CCNA2; NCOA2; NCOA1 |
| MOL007094 | Danshenspiroketallactone | PTGS1; CHRM3; CHRM1; ESR1; SCN5A; CHRM5; PTGS2; CHRM4; RXRA; ACHE; ADRA1A; CHRM2; ADRA1B; ADRB2; ADRA1D; CHRNA2; SLC6A4; OPRM1; GABRA1 |
| MOL007098 | Deoxyneocryptotanshinone | PTGS1; CHRM3; CHRM1; ESR1; AR; SCN5A; CHRM5; PTGS2; CHRM4; RXRA; OPRD1; ADRA1A; CHRM2; ADRA1B; ADRB2; ADRA1D; OPRM1; GSK3B; NCOA2; NCOA1 |
| MOL007100 | Dihydrotanshinlactone | NOS2; PTGS1; CHRM3; CHRM1; ESR1; AR; SCN5A; PPARG; CHRM5; PTGS2; HTR3A; RXRA; ACHE; ADRA1A; ADRA1B; SLC6A3; ADRB2; ADRA1D; SLC6A4; OPRM1; GABRA1; GSK3B; PRSS1; CCNA2 |
| MOL007101 | DihydrotanshinoneⅠ | PTGS1; SCN5A; PTGS2; HTR3A; RXRA; ADRA1A; ADRA1B; ADRB2; GABRA1; NCOA2; NCOA1 |
| MOL007105 | Epidanshenspiroketallactone | PTGS1; CHRM3; CHRM1; ESR1; SCN5A; CHRM5; PTGS2; CHRM4; RXRA; OPRD1; ADRA1A; CHRM2; ADRA1B; ADRB2; ADRA1D; SLC6A4; OPRM1; GABRA1 |
| MOL007107 | C09092 | CHRM3; CHRM1; SCN5A; ACHE; ADRA1A; CHRM2; ADRA1B; ADRB2; ADRA1D; OPRM1 |
| MOL007108 | Isocryptotanshi-none | NOS2; PTGS1; CHRM3; CHRM1; ESR1; AR; SCN5A; CHRM5; PTGS2; CHRM4; RXRA; OPRD1; ACHE; ADRA1A; CHRM2; ADRA1B; ADRB2; ADRA1D; DRD2; OPRM1; GABRA1; PRSS1; NCOA2; NCOA1 |
| MOL007111 | Isotanshinone II | NOS2; CHRM3; CHRM1; ESR1; AR; SCN5A; CHRM5; PTGS2; RXRA; OPRD1; ACHE; ADRA1A; CHRM2; ADRB2; OPRM1; ESR2; GABRA1; GSK3B; CHEK1; CCNA2 |
| MOL007115 | Manool | NCOA2 |
| MOL007119 | Miltionone Ⅰ | PTGS1; CHRM3; CHRM1; ESR1; AR; SCN5A; PTGS2; RXRA; OPRD1; ADRA1A; CHRM2; ADRA1B; ADRB2; OPRM1; NR3C1; GSK3B; CCNA2; NCOA2; NCOA1 |
| MOL007120 | Miltionone Ⅱ | PTGS2; ACHE; PGR; NR3C1; NCOA2; NCOA1 |
| MOL007121 | Miltipolone | ESR1; ACHE |
| MOL007122 | Miltirone | PTGS1; CHRM3; CHRM1; ESR1; AR; DRD5; SCN5A; CHRM5; PTGS2; ADRA2C; CHRM4; RXRA; OPRD1; ADRA1A; CHRM2; ADRA1B; SLC6A3; ADRB2; ADRA1D; OPRM1; NCOA2 |
| MOL007124 | Neocryptotanshinone ii | PTGS1; CHRM3; CHRM1; ESR1; AR; SCN5A; PTGS2; CHRM4; RXRA; OPRD1; ADRA1A; CHRM2; ADRA1B; SLC6A3; ADRB2; ADRA1D; SLC6A4; OPRM1; GABRA1; GSK3B; CCNA2 |
| MOL007125 | Neocryptotanshinone | PTGS1; CHRM3; CHRM1; SCN5A; PPARG; PTGS2; ADRA1B; ADRB2; ADRA1D; OPRM1; NCOA2; NCOA1 |
| MOL007127 | 1-methyl-8,9-dihydro-7H-naphtho[5,6-g]benzofuran-6,10,11-trione | PTGS1; CHRM3; SCN5A; CHRM5; PTGS2; RXRA; ACHE; ADRA1A; ADRB2; OPRM1; GABRA1; NCOA1 |
| MOL007130 | Prolithospermic acid | NOS2; PTGS1; ESR1; AR; PTGS2; PRSS1 |
| MOL007132 | (2R)-3-(3,4-dihydroxyphenyl)-2-[(Z)-3-(3,4-dihydroxyphenyl)acryloyl]oxy-propionic acid | ESR1; AR; PPARG; PTGS2; PRSS1; CCNA2 |
| MOL007141 | Salvianolic acid g | PTGS2 |
| MOL007142 | Salvianolic acid j | F7; PRSS1 |
| MOL007143 | Salvilenone Ⅰ | PTGS2; RXRA; ACHE; PGR; NR3C1; NCOA2; NCOA1 |
| MOL007145 | Salviolone | PTGS1; CHRM3; CHRM1; DRD5; SCN5A; CHRM5; PTGS2; ADRA2A; HTR3A; CHRM4; OPRD1; ACHE; SLC6A2; ADRA1A; CHRM2; ADRA2B; ADRA1B; SLC6A3; ADRB2; CHRNA2; SLC6A4; DRD2; OPRM1; GABRA1; GABRG3; GABRE |
| MOL007150 | (6S)-6-hydroxy-1-methyl-6-methylol-8,9-dihydro-7H-naphtho[8,7-g]benzofuran-10,11-quinone | PTGS2; ACHE; PRSS1; NCOA1 |
| MOL007151 | Tanshindiol B | PTGS2; ACHE; NCOA1 |
| MOL007152 | Przewaquinone E | PTGS2; ACHE; NCOA1 |
| MOL007154 | Tanshinone iia | CHRM3; CHRM1; SCN5A; CHRM5; PTGS2; CHRM4; OPRD1; ACHE; ADRA1A; CHRM2; ADRB2; OPRM1; NCOA1; RXRA; RELA; BCL2; FOS; CDKN1A; MMP9; JUN; AHSA1; CASP3; TP63; NFKBIA; FASN; EDNRA; EDN3; CYP3A4; CYP1A2; MYC; CYP1A1; NR1I2; NPM1; ECE1; PARP4; CALCR; ITGB3 |
| MOL007155 | (6S)-6-(hydroxymethyl)-1,6-dimethyl-8,9-dihydro-7H-naphtho[8,7-g]benzofuran-10,11-dione | CHRM1; SCN5A; PTGS2; OPRD1; ACHE; ADRA1A; ADRB2; OPRM1; PRSS1; NCOA1 |
| MOL007156 | Tanshinone Ⅵ | PTGS1; ESR1; AR; SCN5A; PPARG; PTGS2; NCOA2; NCOA1 |

These genes were downloaded from TCMSP database and conﬁrmed by Drugbank database. All the genes’ names were standardized through UNiProtKB database with *Homo sapiens*.
