# Supplementary material for: Proangiogenesis effects of compound danshen dripping pills in zebrafish
Source: BMC Complement Med Ther. 2022 Apr 22;22:112. doi: 10.1186/s12906-022-03589-y (PMC9034551; doi:10.1186/s12906-022-03589-y)
Supplement: Supplementary file 7 — Additional file 7. Table 6. Genes related to the active ingredients of Panax notoginseng. The screening of ingredient-related genes of Panax notoginsengwas performed using TCMSP and confirmed through the DrugBank database. [file 12906_2022_3589_MOESM7_ESM.docx]

**Supplementary Table 6.** Genes related to the active ingredients of *Panax notoginseng*.

| **Id** | **Ingredients** | **Target genes** |
| --- | --- | --- |
| MOL001494 | Mandenol | PTGS1; PTGS2; NCOA2 |
| MOL001792 | DFV | PTGS1; ESR1; PTGS2; RXRA; ADRB2; MAOB; SLC6A4; PKIA |
| MOL002879 | Diop | SCN5A; ADRB2; CHRM3 |
| MOL000358 | Beta-sitosterol | PGR; NCOA2; PTGS1; PTGS2; KCNH2; CHRM3; CHRM1; SCN5A; CHRM4; ADRA1A; CHRM2; ADRA1B; ADRB2; CHRNA2; SLC6A4; OPRM1; GABRA1; BCL2; BAX; CASP9; JUN; CASP3; CASP8; PRKCA; PON1; MAP2 |
| MOL000449 | Stigmasterol | PGR; NR3C2; NCOA2; ADH1C; RXRA; NCOA1; PTGS1; PTGS2; ADRA2A; SLC6A2; SLC6A3; ADRB2; AKR1B1; PLAU; LTA4H; MAOB; MAOA; CTRB1; CHRM3; CHRM1; ADRB1; SCN5A; ADRA1A; CHRM2; ADRA1B; GABRA1 |
| MOL005344 | Ginsenoside rh2 | BAX; TNFSF15; CASP3; PTGS2; NFKBIA; IL1B; CASP1; IFNG; ADCYAP1; PSMG1; MAP2K4; SLC2A4 |
| MOL000098 | Quercetin | PTGS1; AR; PPARG; PTGS2; NCOA2; AKR1B1; PRSS1; KCNH2; SCN5A; ADRB2; MMP3; F7; RXRA; ACHE; GABRA1; MAOB; RELA; EGFR; AKT1; VEGFA; CCND1; BCL2; BCL2L1; FOS; CDKN1A; EIF6; BAX; CASP9; PLAU; MMP2; MMP9; MAPK1; IL10; EGF; RB1; TNFSF15; JUN; IL6; AHSA1; CASP3; TP63; ELK1; NFKBIA; POR; ODC1; CASP8; TOP1; RAF1; SOD1; PRKCA; MMP1; HIF1A; STAT1; RUNX1T1; ERBB2; PPARG; ACACA; HMOX1; CYP3A4; CYP1A2; CAV1; MYC; F3; GJA1; CYP1A1; ICAM1; IL1B; CCL2; SELE; VCAM1; PTGER3; CXCL8; PRKCB; BIRC5; DUOX2; NOS3; HSPB1; SULT1E1; MGAM; IL2; NR1I2; CYP1B1; CCNB1; PLAT; THBD; SERPINE1; COL1A1; IFNG; ALOX5; IL1A; MPO; TOP2A; NCF1; ABCG2; HAS2; GSTP1; NFE2L2; NQO1; PARP1; AHR; PSMD3; SLC2A4; COL3A1; CXCL11; CXCL2; DCAF5; NR1I3; CHEK2; INSR; CLDN4; PPARA; PPARD; HSF1; CRP; CXCL10; CHUK; SPP1; RUNX2; RASSF1; E2F1; E2F2; ACPP; CTSD; IGFBP3; IGF2; CD40LG; IRF1; ERBB3; PON1; DIO1; PCOLCE; NPEPPS; HK2; RASA1; GSTM1; GSTM2 |

These genes were downloaded from TCMSP database and conﬁrmed by Drugbank database. All the genes’ names were standardized through UNiProtKB database with *Homo sapiens*.
