# Supplementary material for: Proangiogenesis effects of compound danshen dripping pills in zebrafish
Source: BMC Complement Med Ther. 2022 Apr 22;22:112. doi: 10.1186/s12906-022-03589-y (PMC9034551; doi:10.1186/s12906-022-03589-y)
Supplement: Supplementary file 8 — Additional file 8. Table 7. Key genes related to coronary heart disease.The 7,100 coronary heart disease-related genes reported were screened outthrough the GeneCards database [file 12906_2022_3589_MOESM8_ESM.docx]

**Supplementary Table 7.** Key genes related to coronary heart disease.

| **Gene Symbol** | **Description** | **Category** | **GC Id** | **Relevance score** |
| --- | --- | --- | --- | --- |
| IL6 | Interleukin 6 | Protein Coding | GC07P022765 | 121.98 |
| ACE | Angiotensin I Converting Enzyme | Protein Coding | GC17P063477 | 115.92 |
| APOE | Apolipoprotein E | Protein Coding | GC19P044906 | 113.29 |
| TNF | Tumor Necrosis Factor | Protein Coding | GC06P047305 | 110.97 |
| ALB | Albumin | Protein Coding | GC04P073397 | 97.27 |
| SCN5A | Sodium Voltage-Gated Channel Alpha Subunit 5 | Protein Coding | GC03M038549 | 96.34 |
| APOB | Apolipoprotein B | Protein Coding | GC02M020956 | 95.68 |
| NOS3 | Nitric Oxide Synthase 3 | Protein Coding | GC07P150990 | 95.07 |
| LMNA | Lamin A/C | Protein Coding | GC01P156082 | 93.55 |
| IL10 | Interleukin 10 | Protein Coding | GC01M206767 | 93.36 |
| IL1B | Interleukin 1 Beta | Protein Coding | GC02M112829 | 93.33 |
| APOA1 | Apolipoprotein A1 | Protein Coding | GC11M116835 | 91.05 |
| ABCA1 | ATP Binding Cassette Subfamily A Member 1 | Protein Coding | GC09M104781 | 90.82 |
| INS | Insulin | Protein Coding | GC11M002159 | 88.08 |
| GATA4 | GATA Binding Protein 4 | Protein Coding | GC08P011676 | 85.98 |
| VWF | Von Willebrand Factor | Protein Coding | GC12M005917 | 83.67 |
| PRKAG2 | Protein Kinase AMP-Activated Non-Catalytic Subunit Gamma 2 | Protein Coding | GC07M151556 | 83.31 |
| VEGFA | Vascular Endothelial Growth Factor A | Protein Coding | GC06P043770 | 83.1 |
| EDN1 | Endothelin 1 | Protein Coding | GC06P012290 | 82.89 |
| ELN | Elastin | Protein Coding | GC07P074027 | 82.51 |
| NKX2-5 | NK2 Homeobox 5 | Protein Coding | GC05M173232 | 81.65 |
| TGFB1 | Transforming Growth Factor Beta 1 | Protein Coding | GC19M041301 | 80.64 |
| LDLR | Low Density Lipoprotein Receptor | Protein Coding | GC19P011061 | 80.43 |
| CD40LG | CD40 Ligand | Protein Coding | GC0XP136649 | 80.05 |
| F2 | Coagulation Factor II, Thrombin | Protein Coding | GC11P046720 | 79.22 |
| LPL | Lipoprotein Lipase | Protein Coding | GC08P019901 | 78.89 |
| GJA1 | Gap Junction Protein Alpha 1 | Protein Coding | GC06P121436 | 77.36 |
| PON1 | Paraoxonase 1 | Protein Coding | GC07M095297 | 77.27 |
| MYH6 | Myosin Heavy Chain 6 | Protein Coding | GC14M023380 | 77.14 |
| MTHFR | Methylenetetrahydrofolate Reductase | Protein Coding | GC01M011785 | 77.04 |
| TNNI3 | Troponin I3, Cardiac Type | Protein Coding | GC19M055151 | 76.62 |
| PPARG | Peroxisome Proliferator Activated Receptor Gamma | Protein Coding | GC03P012287 | 76.21 |
| NPPA | Natriuretic Peptide A | Protein Coding | GC01M011846 | 76.16 |
| TNNT2 | Troponin T2, Cardiac Type | Protein Coding | GC01M201359 | 75.62 |
| SERPINE1 | Serpin Family E Member 1 | Protein Coding | GC07P101127 | 75.57 |
| CCL2 | C-C Motif Chemokine Ligand 2 | Protein Coding | GC17P034255 | 73.1 |
| TLR4 | Toll Like Receptor 4 | Protein Coding | GC09P117704 | 72.68 |
| TP53 | Tumor Protein P53 | Protein Coding | GC17M007661 | 72.34 |
| MYH7 | Myosin Heavy Chain 7 | Protein Coding | GC14M023412 | 72.01 |
| AGTR1 | Angiotensin II Receptor Type 1 | Protein Coding | GC03P148697 | 71.73 |
| NOTCH1 | Notch Receptor 1 | Protein Coding | GC09M136602 | 71.63 |
| LPA | Lipoprotein(A) | Protein Coding | GC06M160531 | 71.29 |
| CRP | C-Reactive Protein | Protein Coding | GC01M159716 | 70.63 |
| TBX5 | T-Box Transcription Factor 5 | Protein Coding | GC12M114353 | 70.07 |
| TTN | Titin | Protein Coding | GC02M178525 | 69.91 |
| IFNG | Interferon Gamma | Protein Coding | GC12M068064 | 69.9 |
| REN | Renin | Protein Coding | GC01M204154 | 68.66 |
| ICAM1 | Intercellular Adhesion Molecule 1 | Protein Coding | GC19P010270 | 68.11 |
| MMP3 | Matrix Metallopeptidase 3 | Protein Coding | GC11M102835 | 67.2 |
| FBN1 | Fibrillin 1 | Protein Coding | GC15M048408 | 67.07 |
| GATA6 | GATA Binding Protein 6 | Protein Coding | GC18P022169 | 66.68 |
| RYR2 | Ryanodine Receptor 2 | Protein Coding | GC01P237042 | 66.42 |
| CETP | Cholesteryl Ester Transfer Protein | Protein Coding | GC16P056961 | 65.89 |
| KCNQ1 | Potassium Voltage-Gated Channel Subfamily Q Member 1 | Protein Coding | GC11P002444 | 65.49 |
| JAG1 | Jagged Canonical Notch Ligand 1 | Protein Coding | GC20M010637 | 65.43 |
| IGF1 | Insulin Like Growth Factor 1 | Protein Coding | GC12M102395 | 65.17 |
| CDKN2A | Cyclin Dependent Kinase Inhibitor 2A | Protein Coding | GC09M021967 | 63.36 |
| KCNJ5 | Potassium Inwardly Rectifying Channel Subfamily J Member 5 | Protein Coding | GC11P128891 | 62.17 |
| FN1 | Fibronectin 1 | Protein Coding | GC02M215360 | 61.9 |
| KCNH2 | Potassium Voltage-Gated Channel Subfamily H Member 2 | Protein Coding | GC07M150944 | 61.89 |
| RYR1 | Ryanodine Receptor 1 | Protein Coding | GC19P038528 | 61.86 |
| LEP | Leptin | Protein Coding | GC07P128241 | 61.7 |
| PSEN1 | Presenilin 1 | Protein Coding | GC14P073136 | 61.36 |
| AGT | Angiotensinogen | Protein Coding | GC01M230702 | 61.11 |
| MPO | Myeloperoxidase | Protein Coding | GC17M058269 | 60.93 |
| IL1RN | Interleukin 1 Receptor Antagonist | Protein Coding | GC02P115307 | 60.91 |
| LIPC | Lipase C, Hepatic Type | Protein Coding | GC15P058410 | 60.54 |
| NPPB | Natriuretic Peptide B | Protein Coding | GC01M011858 | 60.21 |
| APOC3 | Apolipoprotein C3 | Protein Coding | GC11P116829 | 60.15 |
| TTR | Transthyretin | Protein Coding | GC18P031557 | 60.08 |
| SERPINC1 | Serpin Family C Member 1 | Protein Coding | GC01M174153 | 59.88 |
| CAT | Catalase | Protein Coding | GC11P034460 | 59.85 |
| ACTA2 | Actin Alpha 2, Smooth Muscle | Protein Coding | GC10M088935 | 59.57 |
| MYBPC3 | Myosin Binding Protein C3 | Protein Coding | GC11M061124 | 59.52 |
| KNG1 | Kininogen 1 | Protein Coding | GC03P186717 | 59.4 |
| MMP2 | Matrix Metallopeptidase 2 | Protein Coding | GC16P055390 | 59.31 |
| CACNA1C | Calcium Voltage-Gated Channel Subunit Alpha1 C | Protein Coding | GC12P001970 | 58.88 |
| ADRB1 | Adrenoceptor Beta 1 | Protein Coding | GC10P114044 | 58.83 |
| COG2 | Component Of Oligomeric Golgi Complex 2 | Protein Coding | GC01P230642 | 58.61 |
| PCSK9 | Proprotein Convertase Subtilisin/Kexin Type 9 | Protein Coding | GC01P055039 | 58.39 |
| TBX1 | T-Box Transcription Factor 1 | Protein Coding | GC22P019747 | 58.39 |
| GJA5 | Gap Junction Protein Alpha 5 | Protein Coding | GC01M147756 | 58.29 |
| MIR21 | MicroRNA 21 | RNA Gene | GC17P059841 | 58.27 |
| SOD1 | Superoxide Dismutase 1 | Protein Coding | GC21P031659 | 57.21 |
| EPO | Erythropoietin | Protein Coding | GC07P100720 | 56.54 |
| HMOX1 | Heme Oxygenase 1 | Protein Coding | GC22P035380 | 56.5 |
| PTPN11 | Protein Tyrosine Phosphatase Non-Receptor Type 11 | Protein Coding | GC12P112418 | 56.34 |
| AKT1 | AKT Serine/Threonine Kinase 1 | Protein Coding | GC14M104769 | 55.98 |
| TLR2 | Toll Like Receptor 2 | Protein Coding | GC04P153684 | 55.92 |
| HFE | Homeostatic Iron Regulator | Protein Coding | GC06P026087 | 55.91 |
| STAT3 | Signal Transducer And Activator Of Transcription 3 | Protein Coding | GC17M042313 | 55.78 |
| DSP | Desmoplakin | Protein Coding | GC06P007541 | 55.55 |
| TNFRSF1A | TNF Receptor Superfamily Member 1A | Protein Coding | GC12M006328 | 55.45 |
| DMD | Dystrophin | Protein Coding | GC0XM031047 | 55.16 |
| ESR1 | Estrogen Receptor 1 | Protein Coding | GC06P151656 | 55.14 |
| GLA | Galactosidase Alpha | Protein Coding | GC0XM101393 | 54.97 |
| HLA-DRB1 | Major Histocompatibility Complex, Class II, DR Beta 1 | Protein Coding | GC06M032578 | 54.91 |
| THBD | Thrombomodulin | Protein Coding | GC20M023026 | 54.9 |
| MIR126 | MicroRNA 126 | RNA Gene | GC09P136670 | 54.85 |
| TGFB2 | Transforming Growth Factor Beta 2 | Protein Coding | GC01P218345 | 54.59 |
| ADRB2 | Adrenoceptor Beta 2 | Protein Coding | GC05P148825 | 54.47 |
| APOA5 | Apolipoprotein A5 | Protein Coding | GC11M116789 | 54.29 |
| CTNNB1 | Catenin Beta 1 | Protein Coding | GC03P041236 | 53.96 |
| TGFB3 | Transforming Growth Factor Beta 3 | Protein Coding | GC14M075958 | 53.95 |
| LCAT | Lecithin-Cholesterol Acyltransferase | Protein Coding | GC16M067939 | 53.95 |
| MMP1 | Matrix Metallopeptidase 1 | Protein Coding | GC11M102810 | 53.82 |
| CD36 | CD36 Molecule | Protein Coding | GC07P080369 | 53.24 |
| CST3 | Cystatin C | Protein Coding | GC20M023608 | 53.1 |
| HRAS | HRas Proto-Oncogene, GTPase | Protein Coding | GC11M000635 | 52.89 |
| COMT | Catechol-O-Methyltransferase | Protein Coding | GC22P019941 | 52.75 |
| SMAD4 | SMAD Family Member 4 | Protein Coding | GC18P051028 | 52.74 |
| ENG | Endoglin | Protein Coding | GC09M127815 | 52.65 |
| CD40 | CD40 Molecule | Protein Coding | GC20P046118 | 52.45 |
| MIR146A | MicroRNA 146a | RNA Gene | GC05P160485 | 52.1 |
| SPP1 | Secreted Phosphoprotein 1 | Protein Coding | GC04P087975 | 52.08 |
| ENPP1 | Ectonucleotide Pyrophosphatase/Phosphodiesterase 1 | Protein Coding | GC06P131808 | 51.77 |
| APP | Amyloid Beta Precursor Protein | Protein Coding | GC21M025880 | 51.43 |
| GAA | Glucosidase Alpha, Acid | Protein Coding | GC17P080101 | 51.33 |
| PON2 | Paraoxonase 2 | Protein Coding | GC07M095404 | 51 |
| NOS2 | Nitric Oxide Synthase 2 | Protein Coding | GC17M027756 | 50.6 |
| TNFRSF11B | TNF Receptor Superfamily Member 11b | Protein Coding | GC08M118923 | 50.29 |
| PLA2G7 | Phospholipase A2 Group VII | Protein Coding | GC06M046704 | 50.24 |
| CTLA4 | Cytotoxic T-Lymphocyte Associated Protein 4 | Protein Coding | GC02P203867 | 50 |
| MYH11 | Myosin Heavy Chain 11 | Protein Coding | GC16M015704 | 49.46 |
| RETN | Resistin | Protein Coding | GC19P007669 | 49.4 |
| MIR17 | MicroRNA 17 | RNA Gene | GC13P091350 | 49.29 |
| HSPD1 | Heat Shock Protein Family D (Hsp60) Member 1 | Protein Coding | GC02M197486 | 49.17 |
| CAV1 | Caveolin 1 | Protein Coding | GC07P116524 | 49.09 |
| TNFRSF1B | TNF Receptor Superfamily Member 1B | Protein Coding | GC01P012167 | 49.01 |
| MMP9 | Matrix Metallopeptidase 9 | Protein Coding | GC20P046008 | 48.97 |
| TBX20 | T-Box Transcription Factor 20 | Protein Coding | GC07M035237 | 48.94 |
| CXCR4 | C-X-C Motif Chemokine Receptor 4 | Protein Coding | GC02M136114 | 48.88 |
| ZIC3 | Zic Family Member 3 | Protein Coding | GC0XP137566 | 48.73 |
| CXCL12 | C-X-C Motif Chemokine Ligand 12 | Protein Coding | GC10M044294 | 48.64 |
| SMAD3 | SMAD Family Member 3 | Protein Coding | GC15P067063 | 48.49 |
| FAS | Fas Cell Surface Death Receptor | Protein Coding | GC10P088969 | 48.48 |
| MIR145 | MicroRNA 145 | RNA Gene | GC05P149430 | 48.45 |
| TGFBR2 | Transforming Growth Factor Beta Receptor 2 | Protein Coding | GC03P030623 | 48.34 |
| MIR155 | MicroRNA 155 | RNA Gene | GC21P025573 | 48.23 |
| ACTC1 | Actin Alpha Cardiac Muscle 1 | Protein Coding | GC15M034788 | 48.23 |
| KCNJ2 | Potassium Inwardly Rectifying Channel Subfamily J Member 2 | Protein Coding | GC17P070168 | 48.23 |
| CFTR | CF Transmembrane Conductance Regulator | Protein Coding | GC07P117287 | 48.19 |
| PLG | Plasminogen | Protein Coding | GC06P160702 | 48.05 |
| CXCL8 | C-X-C Motif Chemokine Ligand 8 | Protein Coding | GC04P073740 | 47.97 |
| TGFBR1 | Transforming Growth Factor Beta Receptor 1 | Protein Coding | GC09P099104 | 47.95 |
| ANK2 | Ankyrin 2 | Protein Coding | GC04P112706 | 47.93 |
| DES | Desmin | Protein Coding | GC02P219418 | 47.93 |
| SOD2 | Superoxide Dismutase 2 | Protein Coding | GC06M159669 | 47.9 |
| MAPK1 | Mitogen-Activated Protein Kinase 1 | Protein Coding | GC22M021754 | 47.76 |
| HCN4 | Hyperpolarization Activated Cyclic Nucleotide Gated Potassium Channel 4 | Protein Coding | GC15M073319 | 47.72 |
| CDKN2B | Cyclin Dependent Kinase Inhibitor 2B | Protein Coding | GC09M022002 | 47.61 |
| TRPM4 | Transient Receptor Potential Cation Channel Subfamily M Member 4 | Protein Coding | GC19P049157 | 47.53 |
| FLNA | Filamin A | Protein Coding | GC0XM154348 | 47.36 |
| MEF2A | Myocyte Enhancer Factor 2A | Protein Coding | GC15P099565 | 47.3 |
| LOX | Lysyl Oxidase | Protein Coding | GC05M122063 | 47.23 |
| PRODH | Proline Dehydrogenase 1 | Protein Coding | GC22M018912 | 47.1 |
| KCNE2 | Potassium Voltage-Gated Channel Subfamily E Regulatory Subunit 2 | Protein Coding | GC21P034364 | 47.09 |
| CCR5 | C-C Motif Chemokine Receptor 5 | Protein Coding | GC03P046383 | 47.03 |
| F7 | Coagulation Factor VII | Protein Coding | GC13P113105 | 47.01 |
| ADIPOQ | Adiponectin, C1Q And Collagen Domain Containing | Protein Coding | GC03P186842 | 46.88 |
| F8 | Coagulation Factor VIII | Protein Coding | GC0XM154835 | 46.87 |
| MGP | Matrix Gla Protein | Protein Coding | GC12M014881 | 46.81 |
| HTR2A | 5-Hydroxytryptamine Receptor 2A | Protein Coding | GC13M046831 | 46.72 |
| PKP2 | Plakophilin 2 | Protein Coding | GC12M032790 | 46.39 |
| EDNRA | Endothelin Receptor Type A | Protein Coding | GC04P147480 | 46.38 |
| F5 | Coagulation Factor V | Protein Coding | GC01M169511 | 46.33 |
| NPC1 | NPC Intracellular Cholesterol Transporter 1 | Protein Coding | GC18M023506 | 46.25 |
| H19 | H19 Imprinted Maternally Expressed Transcript | RNA Gene | GC11M001995 | 46.21 |
| CYBA | Cytochrome B-245 Alpha Chain | Protein Coding | GC16M088643 | 46.18 |
| ELANE | Elastase, Neutrophil Expressed | Protein Coding | GC19P000854 | 46.18 |
| ABCG5 | ATP Binding Cassette Subfamily G Member 5 | Protein Coding | GC02M043806 | 46.14 |
| NR3C2 | Nuclear Receptor Subfamily 3 Group C Member 2 | Protein Coding | GC04M148078 | 46.03 |
| BMPR2 | Bone Morphogenetic Protein Receptor Type 2 | Protein Coding | GC02P202376 | 45.91 |
| LDB3 | LIM Domain Binding 3 | Protein Coding | GC10P086666 | 45.77 |
| JUP | Junction Plakoglobin | Protein Coding | GC17M041754 | 45.73 |
| SMPD1 | Sphingomyelin Phosphodiesterase 1 | Protein Coding | GC11P006390 | 45.55 |
| PRKG1 | Protein Kinase CGMP-Dependent 1 | Protein Coding | GC10P050991 | 45.34 |
| TMEM43 | Transmembrane Protein 43 | Protein Coding | GC03P014124 | 45.06 |
| ITGB3 | Integrin Subunit Beta 3 | Protein Coding | GC17P047254 | 45.04 |
| EDNRB | Endothelin Receptor Type B | Protein Coding | GC13M077895 | 44.83 |
| EGFR | Epidermal Growth Factor Receptor | Protein Coding | GC07P055019 | 44.82 |
| SLC6A4 | Solute Carrier Family 6 Member 4 | Protein Coding | GC17M030194 | 44.78 |
| ABCG8 | ATP Binding Cassette Subfamily G Member 8 | Protein Coding | GC02P043828 | 44.62 |
| SOS1 | SOS Ras/Rac Guanine Nucleotide Exchange Factor 1 | Protein Coding | GC02M038981 | 44.59 |
| MIR223 | MicroRNA 223 | RNA Gene | GC0XP066018 | 44.58 |
| LAMP2 | Lysosomal Associated Membrane Protein 2 | Protein Coding | GC0XM120426 | 44.45 |
| POMC | Proopiomelanocortin | Protein Coding | GC02M025160 | 44.34 |
| DSG2 | Desmoglein 2 | Protein Coding | GC18P031498 | 44.31 |
| SELP | Selectin P | Protein Coding | GC01M169558 | 43.91 |
| HLA-DQB1 | Major Histocompatibility Complex, Class II, DQ Beta 1 | Protein Coding | GC06M032804 | 43.77 |
| ABCC9 | ATP Binding Cassette Subfamily C Member 9 | Protein Coding | GC12M021797 | 43.72 |
| KCNE1 | Potassium Voltage-Gated Channel Subfamily E Regulatory Subunit 1 | Protein Coding | GC21M034446 | 43.64 |
| GHRL | Ghrelin And Obestatin Prepropeptide | Protein Coding | GC03M010285 | 43.53 |
| GATA5 | GATA Binding Protein 5 | Protein Coding | GC20M062464 | 43.48 |
| APOA2 | Apolipoprotein A2 | Protein Coding | GC01M161222 | 43.38 |
| COL3A1 | Collagen Type III Alpha 1 Chain | Protein Coding | GC02P188974 | 43.24 |
| CFH | Complement Factor H | Protein Coding | GC01P196621 | 43.08 |
| FOXP3 | Forkhead Box P3 | Protein Coding | GC0XM049250 | 42.76 |
| CRELD1 | Cysteine Rich With EGF Like Domains 1 | Protein Coding | GC03P009960 | 42.7 |
| BMP4 | Bone Morphogenetic Protein 4 | Protein Coding | GC14M053949 | 42.48 |
| HAND2 | Heart And Neural Crest Derivatives Expressed 2 | Protein Coding | GC04M173524 | 42.36 |
| MTR | 5-Methyltetrahydrofolate-Homocysteine Methyltransferase | Protein Coding | GC01P236795 | 42.2 |
| DSC2 | Desmocollin 2 | Protein Coding | GC18M031058 | 42.13 |
| TAZ | Tafazzin | Protein Coding | GC0XP154411 | 42.12 |
| NAGLU | N-Acetyl-Alpha-Glucosaminidase | Protein Coding | GC17P042535 | 42.12 |
| CYP11B2 | Cytochrome P450 Family 11 Subfamily B Member 2 | Protein Coding | GC08M142910 | 42.03 |
| IL18 | Interleukin 18 | Protein Coding | GC11M112143 | 42.02 |
| LRP6 | LDL Receptor Related Protein 6 | Protein Coding | GC12M013893 | 41.92 |
| EGF | Epidermal Growth Factor | Protein Coding | GC04P109912 | 41.92 |
| PSEN2 | Presenilin 2 | Protein Coding | GC01P226870 | 41.84 |
| FOS | Fos Proto-Oncogene, AP-1 Transcription Factor Subunit | Protein Coding | GC14P075278 | 41.74 |
| EMD | Emerin | Protein Coding | GC0XP154379 | 41.62 |
| SLC17A5 | Solute Carrier Family 17 Member 5 | Protein Coding | GC06M073593 | 41.6 |
| TPM1 | Tropomyosin 1 | Protein Coding | GC15P073930 | 41.57 |
| GSR | Glutathione-Disulfide Reductase | Protein Coding | GC08M030678 | 41.56 |
| MT-CO1 | Mitochondrially Encoded Cytochrome C Oxidase I | Protein Coding | GCMTP005906 | 41.54 |
| PITX2 | Paired Like Homeodomain 2 | Protein Coding | GC04M110617 | 41.52 |
| MME | Membrane Metalloendopeptidase | Protein Coding | GC03P155024 | 41.48 |
| CCN2 | Cellular Communication Network Factor 2 | Protein Coding | GC06M131948 | 41.38 |
| OLR1 | Oxidized Low Density Lipoprotein Receptor 1 | Protein Coding | GC12M013864 | 41.32 |
| FCGR2A | Fc Fragment Of IgG Receptor IIa | Protein Coding | GC01P161505 | 41.22 |
| RAF1 | Raf-1 Proto-Oncogene, Serine/Threonine Kinase | Protein Coding | GC03M012583 | 41.18 |
| C3 | Complement C3 | Protein Coding | GC19M006677 | 41.1 |
| FGF23 | Fibroblast Growth Factor 23 | Protein Coding | GC12M004368 | 41.01 |
| MBL2 | Mannose Binding Lectin 2 | Protein Coding | GC10M052760 | 40.87 |
| IL13 | Interleukin 13 | Protein Coding | GC05P132656 | 40.85 |
| PLAT | Plasminogen Activator, Tissue Type | Protein Coding | GC08M042174 | 40.82 |
| PKD2 | Polycystin 2, Transient Receptor Potential Cation Channel | Protein Coding | GC04P088007 | 40.6 |
| ATP2A2 | ATPase Sarcoplasmic/Endoplasmic Reticulum Ca2+ Transporting 2 | Protein Coding | GC12P110280 | 40.55 |
| BSCL2 | BSCL2 Lipid Droplet Biogenesis Associated, Seipin | Protein Coding | GC11M063438 | 40.45 |
| B2M | Beta-2-Microglobulin | Protein Coding | GC15P044711 | 40.19 |
| CACNB2 | Calcium Voltage-Gated Channel Auxiliary Subunit Beta 2 | Protein Coding | GC10P018165 | 40.19 |
| CSRP3 | Cysteine And Glycine Rich Protein 3 | Protein Coding | GC11M019160 | 40.13 |
| SELE | Selectin E | Protein Coding | GC01M169722 | 40.1 |
| TBX2 | T-Box Transcription Factor 2 | Protein Coding | GC17P061399 | 39.84 |
| STAT1 | Signal Transducer And Activator Of Transcription 1 | Protein Coding | GC02M190908 | 39.83 |
| CRYAB | Crystallin Alpha B | Protein Coding | GC11M111908 | 39.81 |
| IRS1 | Insulin Receptor Substrate 1 | Protein Coding | GC02M226731 | 39.74 |
| F3 | Coagulation Factor III, Tissue Factor | Protein Coding | GC01M094530 | 39.7 |
| MYLK | Myosin Light Chain Kinase | Protein Coding | GC03M123610 | 39.69 |
| XDH | Xanthine Dehydrogenase | Protein Coding | GC02M031294 | 39.67 |
| AVP | Arginine Vasopressin | Protein Coding | GC20M003082 | 39.66 |
| FLT1 | Fms Related Receptor Tyrosine Kinase 1 | Protein Coding | GC13M028300 | 39.64 |
| ADAMTS13 | ADAM Metallopeptidase With Thrombospondin Type 1 Motif 13 | Protein Coding | GC09P133414 | 39.55 |
| GP1BA | Glycoprotein Ib Platelet Subunit Alpha | Protein Coding | GC17P004932 | 39.54 |
| GNAS | GNAS Complex Locus | Protein Coding | GC20P058839 | 39.37 |
| TEK | TEK Receptor Tyrosine Kinase | Protein Coding | GC09P027109 | 39.35 |
| MIR150 | MicroRNA 150 | RNA Gene | GC19M049500 | 39.33 |
| JAK2 | Janus Kinase 2 | Protein Coding | GC09P004985 | 39.28 |
| VCAM1 | Vascular Cell Adhesion Molecule 1 | Protein Coding | GC01P100719 | 39.27 |
| LTA | Lymphotoxin Alpha | Protein Coding | GC06P047303 | 39.22 |
| HADHA | Hydroxyacyl-CoA Dehydrogenase Trifunctional Multienzyme Complex Subunit Alpha | Protein Coding | GC02M026190 | 39.21 |
| INSR | Insulin Receptor | Protein Coding | GC19M007112 | 39.13 |
| CCL3 | C-C Motif Chemokine Ligand 3 | Protein Coding | GC17M036088 | 39.13 |
| FXN | Frataxin | Protein Coding | GC09P069035 | 39.02 |
| COL1A1 | Collagen Type I Alpha 1 Chain | Protein Coding | GC17M050183 | 38.95 |
| CHD7 | Chromodomain Helicase DNA Binding Protein 7 | Protein Coding | GC08P060678 | 38.93 |
| MIR221 | MicroRNA 221 | RNA Gene | GC0XM045746 | 38.82 |
| BBS2 | Bardet-Biedl Syndrome 2 | Protein Coding | GC16M056467 | 38.77 |
| PTPRC | Protein Tyrosine Phosphatase Receptor Type C | Protein Coding | GC01P198607 | 38.76 |
| PRDM16 | PR/SET Domain 16 | Protein Coding | GC01P003068 | 38.72 |
| MEF2C | Myocyte Enhancer Factor 2C | Protein Coding | GC05M088718 | 38.62 |
| ITGA2B | Integrin Subunit Alpha 2b | Protein Coding | GC17M044388 | 38.61 |
| MYL2 | Myosin Light Chain 2 | Protein Coding | GC12M110910 | 38.58 |
| ITGAM | Integrin Subunit Alpha M | Protein Coding | GC16P031550 | 38.51 |
| NR2F2 | Nuclear Receptor Subfamily 2 Group F Member 2 | Protein Coding | GC15P096325 | 38.46 |
| CFC1 | Cripto, FRL-1, Cryptic Family 1 | Protein Coding | GC02M130592 | 38.36 |
| IDUA | Alpha-L-Iduronidase | Protein Coding | GC04P000986 | 38.33 |
| MAP2K2 | Mitogen-Activated Protein Kinase Kinase 2 | Protein Coding | GC19M004090 | 38.31 |
| ABCC6 | ATP Binding Cassette Subfamily C Member 6 | Protein Coding | GC16M016148 | 38.24 |
| ECE1 | Endothelin Converting Enzyme 1 | Protein Coding | GC01M021217 | 38.17 |
| HGF | Hepatocyte Growth Factor | Protein Coding | GC07M081699 | 37.89 |
| TBX4 | T-Box Transcription Factor 4 | Protein Coding | GC17P061451 | 37.76 |
| NODAL | Nodal Growth Differentiation Factor | Protein Coding | GC10M070431 | 37.59 |
| CYP2C9 | Cytochrome P450 Family 2 Subfamily C Member 9 | Protein Coding | GC10P094938 | 37.46 |
| FLNC | Filamin C | Protein Coding | GC07P128830 | 37.43 |
| ACTB | Actin Beta | Protein Coding | GC07M005527 | 37.3 |
| PTH | Parathyroid Hormone | Protein Coding | GC11M013492 | 37.26 |
| MYD88 | MYD88 Innate Immune Signal Transduction Adaptor | Protein Coding | GC03P038179 | 37.25 |
| HMGCR | 3-Hydroxy-3-Methylglutaryl-CoA Reductase | Protein Coding | GC05P075336 | 37.22 |
| ABCA4 | ATP Binding Cassette Subfamily A Member 4 | Protein Coding | GC01M093992 | 37.11 |
| TLR3 | Toll Like Receptor 3 | Protein Coding | GC04P186059 | 37.1 |
| KCNJ8 | Potassium Inwardly Rectifying Channel Subfamily J Member 8 | Protein Coding | GC12M021764 | 37.09 |
| IL4 | Interleukin 4 | Protein Coding | GC05P132673 | 37.05 |
| CITED2 | Cbp/P300 Interacting Transactivator With Glu/Asp Rich Carboxy-Terminal Domain 2 | Protein Coding | GC06M139371 | 37 |
| NR3C1 | Nuclear Receptor Subfamily 3 Group C Member 1 | Protein Coding | GC05M143277 | 36.93 |
| MIR29B1 | MicroRNA 29b-1 | RNA Gene | GC07M130877 | 36.88 |
| MYPN | Myopalladin | Protein Coding | GC10P068106 | 36.87 |
| ERCC6 | ERCC Excision Repair 6, Chromatin Remodeling Factor | Protein Coding | GC10M049454 | 36.85 |
| AGTR2 | Angiotensin II Receptor Type 2 | Protein Coding | GC0XP116170 | 36.85 |
| CDKN2B-AS1 | CDKN2B Antisense RNA 1 | RNA Gene | GC09P021994 | 36.77 |
| MIR208A | MicroRNA 208a | RNA Gene | GC14M023388 | 36.64 |
| CPT2 | Carnitine Palmitoyltransferase 2 | Protein Coding | GC01P053196 | 36.6 |
| TBX18 | T-Box Transcription Factor 18 | Protein Coding | GC06M084666 | 36.59 |
| VCL | Vinculin | Protein Coding | GC10P073995 | 36.59 |
| SRC | SRC Proto-Oncogene, Non-Receptor Tyrosine Kinase | Protein Coding | GC20P037344 | 36.47 |
| LRP1 | LDL Receptor Related Protein 1 | Protein Coding | GC12P057128 | 36.38 |
| MIR210 | MicroRNA 210 | RNA Gene | GC11M000674 | 36.34 |
| PLN | Phospholamban | Protein Coding | GC06P118548 | 36.25 |
| GLB1 | Galactosidase Beta 1 | Protein Coding | GC03M033013 | 36.25 |
| NRG1 | Neuregulin 1 | Protein Coding | GC08P031639 | 36.19 |
| ANKRD1 | Ankyrin Repeat Domain 1 | Protein Coding | GC10M090912 | 36.18 |
| MIR483 | MicroRNA 483 | RNA Gene | GC11M002188 | 36.18 |
| ISL1 | ISL LIM Homeobox 1 | Protein Coding | GC05P051383 | 36.15 |
| GPT | Glutamic--Pyruvic Transaminase | Protein Coding | GC08P144502 | 36.14 |
| MT-CYB | Mitochondrially Encoded Cytochrome B | Protein Coding | GCMTP014749 | 36.09 |
| ABL1 | ABL Proto-Oncogene 1, Non-Receptor Tyrosine Kinase | Protein Coding | GC09P130713 | 36.08 |
| PKD1 | Polycystin 1, Transient Receptor Potential Channel Interacting | Protein Coding | GC16M002348 | 36.07 |
| CYP2D6 | Cytochrome P450 Family 2 Subfamily D Member 6 | Protein Coding | GC22M042126 | 36 |
| MYL3 | Myosin Light Chain 3 | Protein Coding | GC03M046836 | 35.97 |
| CAV3 | Caveolin 3 | Protein Coding | GC03P008733 | 35.96 |
| F9 | Coagulation Factor IX | Protein Coding | GC0XP139530 | 35.86 |
| HLA-B | Major Histocompatibility Complex, Class I, B | Protein Coding | GC06M031315 | 35.85 |
| FGA | Fibrinogen Alpha Chain | Protein Coding | GC04M154583 | 35.83 |
| IL2 | Interleukin 2 | Protein Coding | GC04M122451 | 35.82 |
| SDHA | Succinate Dehydrogenase Complex Flavoprotein Subunit A | Protein Coding | GC05P000208 | 35.82 |
| CYBB | Cytochrome B-245 Beta Chain | Protein Coding | GC0XP037780 | 35.73 |
| CASP3 | Caspase 3 | Protein Coding | GC04M184627 | 35.69 |
| GFAP | Glial Fibrillary Acidic Protein | Protein Coding | GC17M044905 | 35.61 |
| TERT | Telomerase Reverse Transcriptase | Protein Coding | GC05M001253 | 35.59 |
| GGT1 | Gamma-Glutamyltransferase 1 | Protein Coding | GC22P024927 | 35.57 |
| ACTN2 | Actinin Alpha 2 | Protein Coding | GC01P236686 | 35.5 |
| IL17A | Interleukin 17A | Protein Coding | GC06P052186 | 35.49 |
| CCL11 | C-C Motif Chemokine Ligand 11 | Protein Coding | GC17P034285 | 35.45 |
| LIPA | Lipase A, Lysosomal Acid Type | Protein Coding | GC10M089213 | 35.44 |
| ALOX5 | Arachidonate 5-Lipoxygenase | Protein Coding | GC10P045374 | 35.4 |
| CALR | Calreticulin | Protein Coding | GC19P012938 | 35.4 |
| ERBB2 | Erb-B2 Receptor Tyrosine Kinase 2 | Protein Coding | GC17P039687 | 35.37 |
| EVC2 | EvC Ciliary Complex Subunit 2 | Protein Coding | GC04M005534 | 35.33 |
| AKAP9 | A-Kinase Anchoring Protein 9 | Protein Coding | GC07P091940 | 35.31 |
| MYC | MYC Proto-Oncogene, BHLH Transcription Factor | Protein Coding | GC08P127735 | 35.25 |
| SLC25A4 | Solute Carrier Family 25 Member 4 | Protein Coding | GC04P185143 | 35.15 |
| MIR22 | MicroRNA 22 | RNA Gene | GC17M001713 | 35.13 |
| CASQ2 | Calsequestrin 2 | Protein Coding | GC01M115700 | 35.05 |
| CX3CR1 | C-X3-C Motif Chemokine Receptor 1 | Protein Coding | GC03M039279 | 34.99 |
| CD4 | CD4 Molecule | Protein Coding | GC12P006786 | 34.94 |
| CACNA1D | Calcium Voltage-Gated Channel Subunit Alpha1 D | Protein Coding | GC03P053328 | 34.92 |
| CYP7A1 | Cytochrome P450 Family 7 Subfamily A Member 1 | Protein Coding | GC08M058476 | 34.74 |
| BMP6 | Bone Morphogenetic Protein 6 | Protein Coding | GC06P007726 | 34.73 |
| KRAS | KRAS Proto-Oncogene, GTPase | Protein Coding | GC12M025204 | 34.64 |
| MIR499A | MicroRNA 499a | RNA Gene | GC20P034990 | 34.6 |
| RBM20 | RNA Binding Motif Protein 20 | Protein Coding | GC10P110644 | 34.58 |
| BAG3 | BAG Cochaperone 3 | Protein Coding | GC10P119651 | 34.58 |
| MIR214 | MicroRNA 214 | RNA Gene | GC01M172234 | 34.52 |
| LAMA2 | Laminin Subunit Alpha 2 | Protein Coding | GC06P128863 | 34.48 |
| NOD2 | Nucleotide Binding Oligomerization Domain Containing 2 | Protein Coding | GC16P050693 | 34.44 |
| FKRP | Fukutin Related Protein | Protein Coding | GC19P046746 | 34.39 |
| MIR27A | MicroRNA 27a | RNA Gene | GC19M014010 | 34.38 |
| MIR30A | MicroRNA 30a | RNA Gene | GC06M071403 | 34.33 |
| EVC | EvC Ciliary Complex Subunit 1 | Protein Coding | GC04P005712 | 34.32 |
| SLC22A5 | Solute Carrier Family 22 Member 5 | Protein Coding | GC05P132369 | 34.18 |
| ADRB3 | Adrenoceptor Beta 3 | Protein Coding | GC08M037962 | 34.17 |
| ACTA1 | Actin Alpha 1, Skeletal Muscle | Protein Coding | GC01M229431 | 34.1 |
| MB | Myoglobin | Protein Coding | GC22M035606 | 34.09 |
| PPARGC1A | PPARG Coactivator 1 Alpha | Protein Coding | GC04M023755 | 34.06 |
| SERPINA3 | Serpin Family A Member 3 | Protein Coding | GC14P094612 | 34.05 |
| P2RY12 | Purinergic Receptor P2Y12 | Protein Coding | GC03M151336 | 34.05 |
| ABCB1 | ATP Binding Cassette Subfamily B Member 1 | Protein Coding | GC07M087504 | 34.03 |
| MYOT | Myotilin | Protein Coding | GC05P137867 | 33.92 |
| DTNA | Dystrobrevin Alpha | Protein Coding | GC18P034493 | 33.9 |
| ITGA2 | Integrin Subunit Alpha 2 | Protein Coding | GC05P052989 | 33.8 |
| HP | Haptoglobin | Protein Coding | GC16P072089 | 33.8 |
| DCN | Decorin | Protein Coding | GC12M091140 | 33.76 |
| FBN2 | Fibrillin 2 | Protein Coding | GC05M128257 | 33.75 |
| BDNF | Brain Derived Neurotrophic Factor | Protein Coding | GC11M027654 | 33.75 |
| SMAD6 | SMAD Family Member 6 | Protein Coding | GC15P066702 | 33.72 |
| MYOCD | Myocardin | Protein Coding | GC17P012665 | 33.66 |
| MIR195 | MicroRNA 195 | RNA Gene | GC17M007018 | 33.65 |
| CD55 | CD55 Molecule (Cromer Blood Group) | Protein Coding | GC01P207321 | 33.63 |
| KITLG | KIT Ligand | Protein Coding | GC12M088492 | 33.63 |
| NEFL | Neurofilament Light | Protein Coding | GC08M024950 | 33.58 |
| PIK3C2A | Phosphatidylinositol-4-Phosphate 3-Kinase Catalytic Subunit Type 2 Alpha | Protein Coding | GC11M017191 | 33.55 |
| IGF2R | Insulin Like Growth Factor 2 Receptor | Protein Coding | GC06P159969 | 33.44 |
| WT1 | WT1 Transcription Factor | Protein Coding | GC11M032365 | 33.35 |
| SGCD | Sarcoglycan Delta | Protein Coding | GC05P155686 | 33.3 |
| ALMS1 | ALMS1 Centrosome And Basal Body Associated Protein | Protein Coding | GC02P073385 | 33.26 |
| NR1H4 | Nuclear Receptor Subfamily 1 Group H Member 4 | Protein Coding | GC12P100473 | 33.25 |
| KCNJ11 | Potassium Inwardly Rectifying Channel Subfamily J Member 11 | Protein Coding | GC11M017364 | 33.18 |
| KDR | Kinase Insert Domain Receptor | Protein Coding | GC04M055078 | 33.18 |
| BMP2 | Bone Morphogenetic Protein 2 | Protein Coding | GC20P006696 | 33.17 |
| F13A1 | Coagulation Factor XIII A Chain | Protein Coding | GC06M006144 | 33.05 |
| TCAP | Titin-Cap | Protein Coding | GC17P039664 | 33.03 |
| CASR | Calcium Sensing Receptor | Protein Coding | GC03P122183 | 33.02 |
| TIMP1 | TIMP Metallopeptidase Inhibitor 1 | Protein Coding | GC0XP047583 | 33.01 |
| LDLRAP1 | Low Density Lipoprotein Receptor Adaptor Protein 1 | Protein Coding | GC01P025574 | 32.93 |
| MIR34A | MicroRNA 34a | RNA Gene | GC01M009151 | 32.87 |
| SCN4B | Sodium Voltage-Gated Channel Beta Subunit 4 | Protein Coding | GC11M118134 | 32.87 |
| CASP8 | Caspase 8 | Protein Coding | GC02P201233 | 32.8 |
| CACNA1S | Calcium Voltage-Gated Channel Subunit Alpha1 S | Protein Coding | GC01M201008 | 32.77 |
| KCND3 | Potassium Voltage-Gated Channel Subfamily D Member 3 | Protein Coding | GC01M111770 | 32.74 |
| PECAM1 | Platelet And Endothelial Cell Adhesion Molecule 1 | Protein Coding | GC17M064319 | 32.64 |
| LEPR | Leptin Receptor | Protein Coding | GC01P065421 | 32.54 |
| ADM | Adrenomedullin | Protein Coding | GC11P010304 | 32.53 |
| CEP290 | Centrosomal Protein 290 | Protein Coding | GC12M088049 | 32.5 |
| AHSG | Alpha 2-HS Glycoprotein | Protein Coding | GC03P186612 | 32.48 |
| BRAF | B-Raf Proto-Oncogene, Serine/Threonine Kinase | Protein Coding | GC07M140719 | 32.46 |
| G6PD | Glucose-6-Phosphate Dehydrogenase | Protein Coding | GC0XM154531 | 32.43 |
| F10 | Coagulation Factor X | Protein Coding | GC13P113122 | 32.36 |
| SNTA1 | Syntrophin Alpha 1 | Protein Coding | GC20M033407 | 32.22 |
| MIR486-1 | MicroRNA 486-1 | RNA Gene | GC08M041660 | 32.21 |
| MAPT | Microtubule Associated Protein Tau | Protein Coding | GC17P045894 | 32.21 |
| COL5A1 | Collagen Type V Alpha 1 Chain | Protein Coding | GC09P134641 | 32.17 |
| MKKS | McKusick-Kaufman Syndrome | Protein Coding | GC20M010412 | 32.14 |
| MED13L | Mediator Complex Subunit 13L | Protein Coding | GC12M115953 | 32.1 |
| FGF2 | Fibroblast Growth Factor 2 | Protein Coding | GC04P122826 | 32.06 |
| GNB3 | G Protein Subunit Beta 3 | Protein Coding | GC12P006839 | 31.95 |
| ABCC8 | ATP Binding Cassette Subfamily C Member 8 | Protein Coding | GC11M017392 | 31.94 |
| MIR222 | MicroRNA 222 | RNA Gene | GC0XM045747 | 31.89 |
| CACNA2D1 | Calcium Voltage-Gated Channel Auxiliary Subunit Alpha2delta 1 | Protein Coding | GC07M081946 | 31.79 |
| MMP13 | Matrix Metallopeptidase 13 | Protein Coding | GC11M102942 | 31.78 |
| TKT | Transketolase | Protein Coding | GC03M053224 | 31.77 |
| KL | Klotho | Protein Coding | GC13P033016 | 31.74 |
| GALNS | Galactosamine (N-Acetyl)-6-Sulfatase | Protein Coding | GC16M088813 | 31.72 |
| NFE2L2 | Nuclear Factor, Erythroid 2 Like 2 | Protein Coding | GC02M177227 | 31.65 |
| EYA4 | EYA Transcriptional Coactivator And Phosphatase 4 | Protein Coding | GC06P133240 | 31.58 |
| SALL1 | Spalt Like Transcription Factor 1 | Protein Coding | GC16M051135 | 31.51 |
| SYNE1 | Spectrin Repeat Containing Nuclear Envelope Protein 1 | Protein Coding | GC06M152121 | 31.45 |
| PROM1 | Prominin 1 | Protein Coding | GC04M015965 | 31.42 |
| ATM | ATM Serine/Threonine Kinase | Protein Coding | GC11P108222 | 31.39 |
| ZMPSTE24 | Zinc Metallopeptidase STE24 | Protein Coding | GC01P040258 | 31.37 |
| ACADVL | Acyl-CoA Dehydrogenase Very Long Chain | Protein Coding | GC17P007219 | 31.35 |
| SCO2 | Synthesis Of Cytochrome C Oxidase 2 | Protein Coding | GC22M050523 | 31.26 |
| AR | Androgen Receptor | Protein Coding | GC0XP067544 | 31.2 |
| PPARA | Peroxisome Proliferator Activated Receptor Alpha | Protein Coding | GC22P046150 | 31.13 |
| TNNT1 | Troponin T1, Slow Skeletal Type | Protein Coding | GC19M055132 | 31.03 |
| TNNC1 | Troponin C1, Slow Skeletal And Cardiac Type | Protein Coding | GC03M052452 | 31 |
| DAG1 | Dystroglycan 1 | Protein Coding | GC03P049482 | 30.98 |
| ADAM17 | ADAM Metallopeptidase Domain 17 | Protein Coding | GC02M009488 | 30.96 |
| SERPINF2 | Serpin Family F Member 2 | Protein Coding | GC17P001742 | 30.95 |
| HIF1A | Hypoxia Inducible Factor 1 Subunit Alpha | Protein Coding | GC14P061695 | 30.94 |
| GDF1 | Growth Differentiation Factor 1 | Protein Coding | GC19M018843 | 30.91 |
| MIR181A1 | MicroRNA 181a-1 | RNA Gene | GC01M198860 | 30.89 |
| ACADM | Acyl-CoA Dehydrogenase Medium Chain | Protein Coding | GC01P075724 | 30.88 |
| PIGL | Phosphatidylinositol Glycan Anchor Biosynthesis Class L | Protein Coding | GC17P016217 | 30.88 |
| FKTN | Fukutin | Protein Coding | GC09P105558 | 30.87 |
| AGPAT2 | 1-Acylglycerol-3-Phosphate O-Acyltransferase 2 | Protein Coding | GC09M136673 | 30.8 |
| WRN | WRN RecQ Like Helicase | Protein Coding | GC08P031033 | 30.7 |
| BMPR1A | Bone Morphogenetic Protein Receptor Type 1A | Protein Coding | GC10P086756 | 30.65 |
| LAMA4 | Laminin Subunit Alpha 4 | Protein Coding | GC06M112107 | 30.63 |
| ARSB | Arylsulfatase B | Protein Coding | GC05M078777 | 30.61 |
| NEXN | Nexilin F-Actin Binding Protein | Protein Coding | GC01P077898 | 30.59 |
| NPC2 | NPC Intracellular Cholesterol Transporter 2 | Protein Coding | GC14M074476 | 30.55 |
| RBP4 | Retinol Binding Protein 4 | Protein Coding | GC10M093591 | 30.45 |
| IL1A | Interleukin 1 Alpha | Protein Coding | GC02M112773 | 30.34 |
| MIR142 | MicroRNA 142 | RNA Gene | GC17M058331 | 30.34 |
| PAH | Phenylalanine Hydroxylase | Protein Coding | GC12M102836 | 30.32 |
| TRDN | Triadin | Protein Coding | GC06M123198 | 30.31 |
| FMR1 | FMRP Translational Regulator 1 | Protein Coding | GC0XP147913 | 30.19 |
| BBS1 | Bardet-Biedl Syndrome 1 | Protein Coding | GC11P066499 | 30.17 |
| MTOR | Mechanistic Target Of Rapamycin Kinase | Protein Coding | GC01M011106 | 30.17 |
| NF1 | Neurofibromin 1 | Protein Coding | GC17P031094 | 30.09 |
| FCGR2B | Fc Fragment Of IgG Receptor IIb | Protein Coding | GC01P161663 | 30.08 |
| MIR140 | MicroRNA 140 | RNA Gene | GC16P069934 | 30.06 |
| PSAP | Prosaposin | Protein Coding | GC10M071816 | 29.97 |
| SYNE2 | Spectrin Repeat Containing Nuclear Envelope Protein 2 | Protein Coding | GC14P063761 | 29.97 |
| DYNC2H1 | Dynein Cytoplasmic 2 Heavy Chain 1 | Protein Coding | GC11P103109 | 29.96 |
| CALM1 | Calmodulin 1 | Protein Coding | GC14P090396 | 29.94 |
| PDCD1 | Programmed Cell Death 1 | Protein Coding | GC02M241849 | 29.93 |
| DYNC2LI1 | Dynein Cytoplasmic 2 Light Intermediate Chain 1 | Protein Coding | GC02P043737 | 29.86 |
| ARG1 | Arginase 1 | Protein Coding | GC06P131473 | 29.83 |
| FABP3 | Fatty Acid Binding Protein 3 | Protein Coding | GC01M031365 | 29.83 |
| SLC2A10 | Solute Carrier Family 2 Member 10 | Protein Coding | GC20P046709 | 29.73 |
| SMAD9 | SMAD Family Member 9 | Protein Coding | GC13M036844 | 29.71 |
| GP6 | Glycoprotein VI Platelet | Protein Coding | GC19M055013 | 29.67 |
| HBB | Hemoglobin Subunit Beta | Protein Coding | GC11M005352 | 29.65 |
| GHR | Growth Hormone Receptor | Protein Coding | GC05P042429 | 29.61 |
| TFAP2B | Transcription Factor AP-2 Beta | Protein Coding | GC06P050894 | 29.58 |
| NF2 | Neurofibromin 2 | Protein Coding | GC22P029603 | 29.54 |
| H2AC18 | H2A Clustered Histone 18 | Protein Coding | GC01M149961 | 29.52 |
| LBR | Lamin B Receptor | Protein Coding | GC01M225401 | 29.49 |
| BCL2 | BCL2 Apoptosis Regulator | Protein Coding | GC18M063123 | 29.46 |
| FBLN5 | Fibulin 5 | Protein Coding | GC14M091869 | 29.4 |
| PTGS2 | Prostaglandin-Endoperoxide Synthase 2 | Protein Coding | GC01M186640 | 29.39 |
| ADRA2B | Adrenoceptor Alpha 2B | Protein Coding | GC02M096112 | 29.37 |
| CIITA | Class II Major Histocompatibility Complex Transactivator | Protein Coding | GC16P010879 | 29.3 |
| F11 | Coagulation Factor XI | Protein Coding | GC04P186265 | 29.26 |
| CBS | Cystathionine Beta-Synthase | Protein Coding | GC21M043053 | 29.23 |
| PTEN | Phosphatase And Tensin Homolog | Protein Coding | GC10P087863 | 29.18 |
| TPM2 | Tropomyosin 2 | Protein Coding | GC09M035672 | 29.13 |
| PLEC | Plectin | Protein Coding | GC08M143916 | 29.12 |
| MIR143 | MicroRNA 143 | RNA Gene | GC05P149410 | 29.08 |
| BLK | BLK Proto-Oncogene, Src Family Tyrosine Kinase | Protein Coding | GC08P011486 | 29.03 |
| ALPL | Alkaline Phosphatase, Biomineralization Associated | Protein Coding | GC01P021508 | 29.03 |
| SAA1 | Serum Amyloid A1 | Protein Coding | GC11P018267 | 28.96 |
| CELA2A | Chymotrypsin Like Elastase 2A | Protein Coding | GC01P015456 | 28.94 |
| PF4 | Platelet Factor 4 | Protein Coding | GC04M073980 | 28.92 |
| ACVRL1 | Activin A Receptor Like Type 1 | Protein Coding | GC12P051906 | 28.9 |
| TH | Tyrosine Hydroxylase | Protein Coding | GC11M002163 | 28.9 |
| MYL4 | Myosin Light Chain 4 | Protein Coding | GC17P047189 | 28.9 |
| CORIN | Corin, Serine Peptidase | Protein Coding | GC04M047596 | 28.85 |
| MYOZ2 | Myozenin 2 | Protein Coding | GC04P119135 | 28.78 |
| ZFPM2 | Zinc Finger Protein, FOG Family Member 2 | Protein Coding | GC08P104590 | 28.74 |
| NEU1 | Neuraminidase 1 | Protein Coding | GC06M031857 | 28.74 |
| POLG | DNA Polymerase Gamma, Catalytic Subunit | Protein Coding | GC15M089316 | 28.7 |
| AGL | Amylo-Alpha-1, 6-Glucosidase, 4-Alpha-Glucanotransferase | Protein Coding | GC01P099850 | 28.69 |
| VCP | Valosin Containing Protein | Protein Coding | GC09M035056 | 28.69 |
| PDE4D | Phosphodiesterase 4D | Protein Coding | GC05M058969 | 28.64 |
| SGCA | Sarcoglycan Alpha | Protein Coding | GC17P050164 | 28.63 |
| PRKAR1A | Protein Kinase CAMP-Dependent Type I Regulatory Subunit Alpha | Protein Coding | GC17P068414 | 28.63 |
| LRP5 | LDL Receptor Related Protein 5 | Protein Coding | GC11P068298 | 28.54 |
| TNFRSF11A | TNF Receptor Superfamily Member 11a | Protein Coding | GC18P062325 | 28.52 |
| NPY | Neuropeptide Y | Protein Coding | GC07P024290 | 28.43 |
| ADD1 | Adducin 1 | Protein Coding | GC04P002855 | 28.41 |
| SGSH | N-Sulfoglucosamine Sulfohydrolase | Protein Coding | GC17M080206 | 28.36 |
| C1S | Complement C1s | Protein Coding | GC12P008238 | 28.35 |
| MLXIPL | MLX Interacting Protein Like | Protein Coding | GC07M073593 | 28.34 |
| COX5A | Cytochrome C Oxidase Subunit 5A | Protein Coding | GC15M074919 | 28.24 |
| GCK | Glucokinase | Protein Coding | GC07M044145 | 28.24 |
| COL5A2 | Collagen Type V Alpha 2 Chain | Protein Coding | GC02M189031 | 28.23 |
| TIMP3 | TIMP Metallopeptidase Inhibitor 3 | Protein Coding | GC22P032800 | 28.21 |
| GBE1 | 1,4-Alpha-Glucan Branching Enzyme 1 | Protein Coding | GC03M081489 | 28.19 |
| RPGRIP1L | RPGRIP1 Like | Protein Coding | GC16M053597 | 28.17 |
| TYR | Tyrosinase | Protein Coding | GC11P089177 | 28.17 |
| AVPR2 | Arginine Vasopressin Receptor 2 | Protein Coding | GC0XP153902 | 28.03 |
| TRPV4 | Transient Receptor Potential Cation Channel Subfamily V Member 4 | Protein Coding | GC12M109783 | 28.03 |
| SLC25A20 | Solute Carrier Family 25 Member 20 | Protein Coding | GC03M048869 | 27.99 |
| TNNI3K | TNNI3 Interacting Kinase | Protein Coding | GC01P074235 | 27.97 |
| JPH2 | Junctophilin 2 | Protein Coding | GC20M044111 | 27.93 |
| ABCA3 | ATP Binding Cassette Subfamily A Member 3 | Protein Coding | GC16M002275 | 27.93 |
| MIR144 | MicroRNA 144 | RNA Gene | GC17M029965 | 27.92 |
| VHL | Von Hippel-Lindau Tumor Suppressor | Protein Coding | GC03P010211 | 27.92 |
| GDNF | Glial Cell Derived Neurotrophic Factor | Protein Coding | GC05M037812 | 27.87 |
| PNPLA2 | Patatin Like Phospholipase Domain Containing 2 | Protein Coding | GC11P000896 | 27.83 |
| PTGIS | Prostaglandin I2 Synthase | Protein Coding | GC20M049503 | 27.82 |
| PTPN22 | Protein Tyrosine Phosphatase Non-Receptor Type 22 | Protein Coding | GC01M113813 | 27.73 |
| SCN2B | Sodium Voltage-Gated Channel Beta Subunit 2 | Protein Coding | GC11M118163 | 27.72 |
| VKORC1 | Vitamin K Epoxide Reductase Complex Subunit 1 | Protein Coding | GC16M031105 | 27.69 |
| SORL1 | Sortilin Related Receptor 1 | Protein Coding | GC11P121452 | 27.69 |
| MIR125A | MicroRNA 125a | RNA Gene | GC19P051720 | 27.59 |
| SHH | Sonic Hedgehog Signaling Molecule | Protein Coding | GC07M155799 | 27.59 |
| PINK1 | PTEN Induced Kinase 1 | Protein Coding | GC01P020634 | 27.59 |
| RUNX2 | RUNX Family Transcription Factor 2 | Protein Coding | GC06P047549 | 27.57 |
| FGB | Fibrinogen Beta Chain | Protein Coding | GC04P154564 | 27.54 |
| MTTP | Microsomal Triglyceride Transfer Protein | Protein Coding | GC04P099563 | 27.52 |
| PDE5A | Phosphodiesterase 5A | Protein Coding | GC04M119494 | 27.51 |
| COL4A1 | Collagen Type IV Alpha 1 Chain | Protein Coding | GC13M110148 | 27.47 |
| SPTA1 | Spectrin Alpha, Erythrocytic 1 | Protein Coding | GC01M158610 | 27.45 |
| TPM3 | Tropomyosin 3 | Protein Coding | GC01M154127 | 27.43 |
| IL2RA | Interleukin 2 Receptor Subunit Alpha | Protein Coding | GC10M006010 | 27.42 |
| LCN2 | Lipocalin 2 | Protein Coding | GC09P128149 | 27.39 |
| CDH2 | Cadherin 2 | Protein Coding | GC18M027950 | 27.39 |
| NPR2 | Natriuretic Peptide Receptor 2 | Protein Coding | GC09P035782 | 27.38 |
| MAP2K1 | Mitogen-Activated Protein Kinase Kinase 1 | Protein Coding | GC15P066386 | 27.37 |
| MRAP | Melanocortin 2 Receptor Accessory Protein | Protein Coding | GC21P032291 | 27.36 |
| MT-ND1 | Mitochondrially Encoded NADH:Ubiquinone Oxidoreductase Core Subunit 1 | Protein Coding | GCMTP003309 | 27.35 |
| PHACTR1 | Phosphatase And Actin Regulator 1 | Protein Coding | GC06P012717 | 27.33 |
| XK | X-Linked Kx Blood Group | Protein Coding | GC0XP037685 | 27.29 |
| TMPO | Thymopoietin | Protein Coding | GC12P098515 | 27.26 |
| CALCA | Calcitonin Related Polypeptide Alpha | Protein Coding | GC11M014945 | 27.25 |
| PYGM | Glycogen Phosphorylase, Muscle Associated | Protein Coding | GC11M064746 | 27.24 |
| NPHP3 | Nephrocystin 3 | Protein Coding | GC03M132683 | 27.24 |
| FIG4 | FIG4 Phosphoinositide 5-Phosphatase | Protein Coding | GC06P109691 | 27.12 |
| CTNNA3 | Catenin Alpha 3 | Protein Coding | GC10M065912 | 27.1 |
| IGF2 | Insulin Like Growth Factor 2 | Protein Coding | GC11M002130 | 27.05 |
| GAPDH | Glyceraldehyde-3-Phosphate Dehydrogenase | Protein Coding | GC12P008161 | 27.05 |
| MTRR | 5-Methyltetrahydrofolate-Homocysteine Methyltransferase Reductase | Protein Coding | GC05P007851 | 27.04 |
| CALM2 | Calmodulin 2 | Protein Coding | GC02M047124 | 26.98 |
| EEF1A2 | Eukaryotic Translation Elongation Factor 1 Alpha 2 | Protein Coding | GC20M063488 | 26.97 |
| GPX1 | Glutathione Peroxidase 1 | Protein Coding | GC03M049368 | 26.92 |
| TSC2 | TSC Complex Subunit 2 | Protein Coding | GC16P002436 | 26.87 |
| DNASE1 | Deoxyribonuclease 1 | Protein Coding | GC16P003611 | 26.83 |
| CCL5 | C-C Motif Chemokine Ligand 5 | Protein Coding | GC17M035871 | 26.81 |
| NRAS | NRAS Proto-Oncogene, GTPase | Protein Coding | GC01M114704 | 26.79 |
| BBS4 | Bardet-Biedl Syndrome 4 | Protein Coding | GC15P072686 | 26.76 |
| IL12B | Interleukin 12B | Protein Coding | GC05M159314 | 26.76 |
| TNFSF11 | TNF Superfamily Member 11 | Protein Coding | GC13P042562 | 26.74 |
| BAZ1B | Bromodomain Adjacent To Zinc Finger Domain 1B | Protein Coding | GC07M073440 | 26.72 |
| GPD1L | Glycerol-3-Phosphate Dehydrogenase 1 Like | Protein Coding | GC03P032123 | 26.7 |
| APLN | Apelin | Protein Coding | GC0XM129645 | 26.7 |
| DNAJC19 | DnaJ Heat Shock Protein Family (Hsp40) Member C19 | Protein Coding | GC03M180983 | 26.68 |
| HAMP | Hepcidin Antimicrobial Peptide | Protein Coding | GC19P038216 | 26.68 |
| CAVIN1 | Caveolae Associated Protein 1 | Protein Coding | GC17M042404 | 26.67 |
| ACTG1 | Actin Gamma 1 | Protein Coding | GC17M081509 | 26.58 |
| NPHP1 | Nephrocystin 1 | Protein Coding | GC02M110122 | 26.46 |
| SH2B3 | SH2B Adaptor Protein 3 | Protein Coding | GC12P111405 | 26.39 |
| IDS | Iduronate 2-Sulfatase | Protein Coding | GC0XM149476 | 26.36 |
| MSTN | Myostatin | Protein Coding | GC02M190055 | 26.33 |
| SPARC | Secreted Protein Acidic And Cysteine Rich | Protein Coding | GC05M151639 | 26.28 |
| AGER | Advanced Glycosylation End-Product Specific Receptor | Protein Coding | GC06M032180 | 26.27 |
| MAPK14 | Mitogen-Activated Protein Kinase 14 | Protein Coding | GC06P047451 | 26.27 |
| SERPINA1 | Serpin Family A Member 1 | Protein Coding | GC14M094376 | 26.25 |
| NFKBIA | NFKB Inhibitor Alpha | Protein Coding | GC14M035401 | 26.23 |
| MFAP5 | Microfibril Associated Protein 5 | Protein Coding | GC12M008637 | 26.22 |
| MYH9 | Myosin Heavy Chain 9 | Protein Coding | GC22M036281 | 26.15 |
| C4A | Complement C4A (Rodgers Blood Group) | Protein Coding | GC06P047332 | 26.12 |
| MIR92B | MicroRNA 92b | RNA Gene | GC01P155195 | 26.12 |
| FGFR1 | Fibroblast Growth Factor Receptor 1 | Protein Coding | GC08M038400 | 26.09 |
| TNFSF4 | TNF Superfamily Member 4 | Protein Coding | GC01M173183 | 26.09 |
| CP | Ceruloplasmin | Protein Coding | GC03M149162 | 26.08 |
| CSF3 | Colony Stimulating Factor 3 | Protein Coding | GC17P040015 | 26.08 |
| KIT | KIT Proto-Oncogene, Receptor Tyrosine Kinase | Protein Coding | GC04P054657 | 26.07 |
| MIR122 | MicroRNA 122 | RNA Gene | GC18P058451 | 26.07 |
| ACAN | Aggrecan | Protein Coding | GC15P088813 | 25.94 |
| MFN2 | Mitofusin 2 | Protein Coding | GC01P011980 | 25.9 |
| TXNRD2 | Thioredoxin Reductase 2 | Protein Coding | GC22M019863 | 25.89 |
| FOXE3 | Forkhead Box E3 | Protein Coding | GC01P047416 | 25.88 |
| IL6ST | Interleukin 6 Signal Transducer | Protein Coding | GC05M055935 | 25.88 |
| OTC | Ornithine Carbamoyltransferase | Protein Coding | GC0XP038353 | 25.82 |
| MIR30E | MicroRNA 30e | RNA Gene | GC01P040754 | 25.73 |
| LTBP2 | Latent Transforming Growth Factor Beta Binding Protein 2 | Protein Coding | GC14M074498 | 25.73 |
| SLC2A4 | Solute Carrier Family 2 Member 4 | Protein Coding | GC17P007295 | 25.72 |
| MIAT | Myocardial Infarction Associated Transcript | RNA Gene | GC22P026646 | 25.71 |
| CYP19A1 | Cytochrome P450 Family 19 Subfamily A Member 1 | Protein Coding | GC15M051208 | 25.66 |
| GATAD1 | GATA Zinc Finger Domain Containing 1 | Protein Coding | GC07P092447 | 25.65 |
| XYLT2 | Xylosyltransferase 2 | Protein Coding | GC17P050347 | 25.64 |
| MIR182 | MicroRNA 182 | RNA Gene | GC07M129770 | 25.61 |
| SDHB | Succinate Dehydrogenase Complex Iron Sulfur Subunit B | Protein Coding | GC01M017020 | 25.6 |
| GNPTAB | N-Acetylglucosamine-1-Phosphate Transferase Subunits Alpha And Beta | Protein Coding | GC12M101745 | 25.57 |
| NEK9 | NIMA Related Kinase 9 | Protein Coding | GC14M075079 | 25.57 |
| CDKN1C | Cyclin Dependent Kinase Inhibitor 1C | Protein Coding | GC11M002887 | 25.56 |
| APOH | Apolipoprotein H | Protein Coding | GC17M066212 | 25.55 |
| MIR23A | MicroRNA 23a | RNA Gene | GC19M014011 | 25.54 |
| HSPA4 | Heat Shock Protein Family A (Hsp70) Member 4 | Protein Coding | GC05P133051 | 25.42 |
| S100B | S100 Calcium Binding Protein B | Protein Coding | GC21M047431 | 25.41 |
| ITGB2 | Integrin Subunit Beta 2 | Protein Coding | GC21M044885 | 25.35 |
| SGCB | Sarcoglycan Beta | Protein Coding | GC04M052019 | 25.31 |
| RPGR | Retinitis Pigmentosa GTPase Regulator | Protein Coding | GC0XM038269 | 25.27 |
| NSD1 | Nuclear Receptor Binding SET Domain Protein 1 | Protein Coding | GC05P177134 | 25.26 |
| CD34 | CD34 Molecule | Protein Coding | GC01M207880 | 25.23 |
| SELL | Selectin L | Protein Coding | GC01M169690 | 25.15 |
| GUCY1A1 | Guanylate Cyclase 1 Soluble Subunit Alpha 1 | Protein Coding | GC04P155667 | 25.15 |
| MASP2 | Mannan Binding Lectin Serine Peptidase 2 | Protein Coding | GC01M011026 | 25.13 |
| CDKN1A | Cyclin Dependent Kinase Inhibitor 1A | Protein Coding | GC06P047460 | 25.1 |
| GTF2IRD1 | GTF2I Repeat Domain Containing 1 | Protein Coding | GC07P074461 | 25.07 |
| DCAF8 | DDB1 And CUL4 Associated Factor 8 | Protein Coding | GC01M160215 | 25.06 |
| DNM2 | Dynamin 2 | Protein Coding | GC19P010718 | 25.05 |
| GDF15 | Growth Differentiation Factor 15 | Protein Coding | GC19P023329 | 25.05 |
| MYBPC1 | Myosin Binding Protein C1 | Protein Coding | GC12P101568 | 25.04 |
| MIR29A | MicroRNA 29a | RNA Gene | GC07M130876 | 25.02 |
| TFAM | Transcription Factor A, Mitochondrial | Protein Coding | GC10P058385 | 25.02 |
| MTM1 | Myotubularin 1 | Protein Coding | GC0XP150562 | 25.01 |
| EPHX2 | Epoxide Hydrolase 2 | Protein Coding | GC08P027490 | 24.99 |
| BTD | Biotinidase | Protein Coding | GC03P015621 | 24.99 |
| ANKH | ANKH Inorganic Pyrophosphate Transport Regulator | Protein Coding | GC05M014706 | 24.93 |
| DNAH5 | Dynein Axonemal Heavy Chain 5 | Protein Coding | GC05M013745 | 24.91 |
| HGD | Homogentisate 1,2-Dioxygenase | Protein Coding | GC03M120628 | 24.9 |
| FASLG | Fas Ligand | Protein Coding | GC01P172628 | 24.9 |
| YY1AP1 | YY1 Associated Protein 1 | Protein Coding | GC01M155659 | 24.89 |
| SGCG | Sarcoglycan Gamma | Protein Coding | GC13P023160 | 24.86 |
| ARL13B | ADP Ribosylation Factor Like GTPase 13B | Protein Coding | GC03P093980 | 24.85 |
| COL2A1 | Collagen Type II Alpha 1 Chain | Protein Coding | GC12M047972 | 24.84 |
| CLU | Clusterin | Protein Coding | GC08M027596 | 24.84 |
| MIR199A1 | MicroRNA 199a-1 | RNA Gene | GC19M010792 | 24.83 |
| PIK3CA | Phosphatidylinositol-4,5-Bisphosphate 3-Kinase Catalytic Subunit Alpha | Protein Coding | GC03P179148 | 24.83 |
| NLRP3 | NLR Family Pyrin Domain Containing 3 | Protein Coding | GC01P247415 | 24.77 |
| LIMK1 | LIM Domain Kinase 1 | Protein Coding | GC07P074082 | 24.69 |
| DNM1L | Dynamin 1 Like | Protein Coding | GC12P032679 | 24.66 |
| SQSTM1 | Sequestosome 1 | Protein Coding | GC05P179806 | 24.65 |
| EFEMP2 | EGF Containing Fibulin Extracellular Matrix Protein 2 | Protein Coding | GC11M065867 | 24.61 |
| RERE | Arginine-Glutamic Acid Dipeptide Repeats | Protein Coding | GC01M008364 | 24.59 |
| HJV | Hemojuvelin BMP Co-Receptor | Protein Coding | GC01M146018 | 24.58 |
| MEN1 | Menin 1 | Protein Coding | GC11M064803 | 24.57 |
| GNS | Glucosamine (N-Acetyl)-6-Sulfatase | Protein Coding | GC12M064713 | 24.54 |
| ADCY5 | Adenylate Cyclase 5 | Protein Coding | GC03M123282 | 24.51 |
| NRXN1 | Neurexin 1 | Protein Coding | GC02M049918 | 24.49 |
| MT-ATP6 | Mitochondrially Encoded ATP Synthase Membrane Subunit 6 | Protein Coding | GCMTP008531 | 24.49 |
| GYS1 | Glycogen Synthase 1 | Protein Coding | GC19M048970 | 24.47 |
| GGCX | Gamma-Glutamyl Carboxylase | Protein Coding | GC02M085544 | 24.46 |
| NDUFS4 | NADH:Ubiquinone Oxidoreductase Subunit S4 | Protein Coding | GC05P053560 | 24.44 |
| SDHD | Succinate Dehydrogenase Complex Subunit D | Protein Coding | GC11P112087 | 24.37 |
| ZNF687 | Zinc Finger Protein 687 | Protein Coding | GC01P151281 | 24.36 |
| NOS1 | Nitric Oxide Synthase 1 | Protein Coding | GC12M117208 | 24.34 |
| IFT80 | Intraflagellar Transport 80 | Protein Coding | GC03M160256 | 24.3 |
| DMPK | DM1 Protein Kinase | Protein Coding | GC19M045769 | 24.3 |
| ALOX5AP | Arachidonate 5-Lipoxygenase Activating Protein | Protein Coding | GC13P030713 | 24.28 |
| MIR296 | MicroRNA 296 | RNA Gene | GC20M058817 | 24.25 |
| FOXC2 | Forkhead Box C2 | Protein Coding | GC16P086567 | 24.23 |
| PGM1 | Phosphoglucomutase 1 | Protein Coding | GC01P063593 | 24.2 |
| THBS2 | Thrombospondin 2 | Protein Coding | GC06M169215 | 24.17 |
| PPBP | Pro-Platelet Basic Protein | Protein Coding | GC04M073986 | 24.16 |
| DRD2 | Dopamine Receptor D2 | Protein Coding | GC11M113409 | 24.15 |
| CYCS | Cytochrome C, Somatic | Protein Coding | GC07M025118 | 24.14 |
| BGN | Biglycan | Protein Coding | GC0XP153494 | 24.13 |
| HLA-A | Major Histocompatibility Complex, Class I, A | Protein Coding | GC06P047265 | 24.11 |
| TF | Transferrin | Protein Coding | GC03P133666 | 24.11 |
| NT5E | 5'-Nucleotidase Ecto | Protein Coding | GC06P085449 | 24.09 |
| CTSA | Cathepsin A | Protein Coding | GC20P045890 | 24.08 |
| MYRF | Myelin Regulatory Factor | Protein Coding | GC11P061753 | 24.07 |
| BRCA2 | BRCA2 DNA Repair Associated | Protein Coding | GC13P032315 | 24.05 |
| IL23R | Interleukin 23 Receptor | Protein Coding | GC01P067138 | 24.02 |
| EP300 | E1A Binding Protein P300 | Protein Coding | GC22P041091 | 24.01 |
| PALLD | Palladin, Cytoskeletal Associated Protein | Protein Coding | GC04P168497 | 23.99 |
| IFIH1 | Interferon Induced With Helicase C Domain 1 | Protein Coding | GC02M162267 | 23.97 |
| HSPB1 | Heat Shock Protein Family B (Small) Member 1 | Protein Coding | GC07P076302 | 23.94 |
| HGSNAT | Heparan-Alpha-Glucosaminide N-Acetyltransferase | Protein Coding | GC08P043140 | 23.92 |
| RAC1 | Rac Family Small GTPase 1 | Protein Coding | GC07P006380 | 23.9 |
| SLC8A1 | Solute Carrier Family 8 Member A1 | Protein Coding | GC02M040078 | 23.89 |
| DNAH8 | Dynein Axonemal Heavy Chain 8 | Protein Coding | GC06P047481 | 23.88 |
| ETFDH | Electron Transfer Flavoprotein Dehydrogenase | Protein Coding | GC04P158672 | 23.84 |
| CHKB | Choline Kinase Beta | Protein Coding | GC22M050578 | 23.84 |
| MIR33A | MicroRNA 33a | RNA Gene | GC22P041900 | 23.8 |
| PTGS1 | Prostaglandin-Endoperoxide Synthase 1 | Protein Coding | GC09P122370 | 23.75 |
| CLIP2 | CAP-Gly Domain Containing Linker Protein 2 | Protein Coding | GC07P074289 | 23.75 |
| NKX2-6 | NK2 Homeobox 6 | Protein Coding | GC08M023702 | 23.73 |
| PIK3R1 | Phosphoinositide-3-Kinase Regulatory Subunit 1 | Protein Coding | GC05P068215 | 23.72 |
| BBS7 | Bardet-Biedl Syndrome 7 | Protein Coding | GC04M121824 | 23.71 |
| ITGA7 | Integrin Subunit Alpha 7 | Protein Coding | GC12M055684 | 23.68 |
| KALRN | Kalirin RhoGEF Kinase | Protein Coding | GC03P124033 | 23.67 |
| PDGFRA | Platelet Derived Growth Factor Receptor Alpha | Protein Coding | GC04P054229 | 23.63 |
| NOTCH2 | Notch Receptor 2 | Protein Coding | GC01M119911 | 23.57 |
| PTCH1 | Patched 1 | Protein Coding | GC09M095442 | 23.54 |
| LRP8 | LDL Receptor Related Protein 8 | Protein Coding | GC01M053243 | 23.53 |
| GSN | Gelsolin | Protein Coding | GC09P121201 | 23.53 |
| BRCA1 | BRCA1 DNA Repair Associated | Protein Coding | GC17M043044 | 23.53 |
| MC4R | Melanocortin 4 Receptor | Protein Coding | GC18M060371 | 23.52 |
| NOTCH3 | Notch Receptor 3 | Protein Coding | GC19M015159 | 23.48 |
| TBL2 | Transducin Beta Like 2 | Protein Coding | GC07M073568 | 23.46 |
| EPOR | Erythropoietin Receptor | Protein Coding | GC19M011377 | 23.43 |
| LGALS3 | Galectin 3 | Protein Coding | GC14P055124 | 23.33 |
| XYLT1 | Xylosyltransferase 1 | Protein Coding | GC16M017101 | 23.33 |
| MT-ND4 | Mitochondrially Encoded NADH:Ubiquinone Oxidoreductase Core Subunit 4 | Protein Coding | GCMTP010762 | 23.32 |
| GTF2I | General Transcription Factor IIi | Protein Coding | GC07P074658 | 23.31 |
| MAPK8 | Mitogen-Activated Protein Kinase 8 | Protein Coding | GC10P048306 | 23.28 |
| ALDH2 | Aldehyde Dehydrogenase 2 Family Member | Protein Coding | GC12P111766 | 23.27 |
| ANGPT1 | Angiopoietin 1 | Protein Coding | GC08M107246 | 23.26 |
| FUCA1 | Alpha-L-Fucosidase 1 | Protein Coding | GC01M023845 | 23.26 |
| CYP2C19 | Cytochrome P450 Family 2 Subfamily C Member 19 | Protein Coding | GC10P094762 | 23.2 |
| MT-CO3 | Mitochondrially Encoded Cytochrome C Oxidase III | Protein Coding | GCMTP009209 | 23.2 |
| NGF | Nerve Growth Factor | Protein Coding | GC01M115285 | 23.2 |
| SOD3 | Superoxide Dismutase 3 | Protein Coding | GC04P024798 | 23.2 |
| CALM3 | Calmodulin 3 | Protein Coding | GC19P046601 | 23.18 |
| FAH | Fumarylacetoacetate Hydrolase | Protein Coding | GC15P080152 | 23.17 |
| FGFR2 | Fibroblast Growth Factor Receptor 2 | Protein Coding | GC10M121478 | 23.14 |
| RB1 | RB Transcriptional Corepressor 1 | Protein Coding | GC13P048303 | 23.13 |
| NDE1 | NudE Neurodevelopment Protein 1 | Protein Coding | GC16P015661 | 23.1 |
| POSTN | Periostin | Protein Coding | GC13M037562 | 23.08 |
| DDX58 | DExD/H-Box Helicase 58 | Protein Coding | GC09M032455 | 23.07 |
| DDX41 | DEAD-Box Helicase 41 | Protein Coding | GC05M177511 | 23.07 |
| TSC1 | TSC Complex Subunit 1 | Protein Coding | GC09M132891 | 23.06 |
| HSD11B1 | Hydroxysteroid 11-Beta Dehydrogenase 1 | Protein Coding | GC01P209686 | 23.04 |
| APOA4 | Apolipoprotein A4 | Protein Coding | GC11M116820 | 23.03 |
| MIR424 | MicroRNA 424 | RNA Gene | GC0XM134627 | 23.02 |
| NCF1 | Neutrophil Cytosolic Factor 1 | Protein Coding | GC07P074773 | 23.02 |
| BANF1 | BAF Nuclear Assembly Factor 1 | Protein Coding | GC11P066002 | 23.01 |
| MIR196A2 | MicroRNA 196a-2 | RNA Gene | GC12P054200 | 22.98 |
| ANXA5 | Annexin A5 | Protein Coding | GC04M121667 | 22.94 |
| SCARB1 | Scavenger Receptor Class B Member 1 | Protein Coding | GC12M124776 | 22.88 |
| MIR320A | MicroRNA 320a | RNA Gene | GC08M022246 | 22.85 |
| PHEX | Phosphate Regulating Endopeptidase Homolog X-Linked | Protein Coding | GC0XP022032 | 22.84 |
| BTK | Bruton Tyrosine Kinase | Protein Coding | GC0XM101349 | 22.81 |
| MT-TL1 | Mitochondrially Encoded TRNA-Leu (UUA/G) 1 | RNA Gene | GCMTP003232 | 22.81 |
| SLC9A1 | Solute Carrier Family 9 Member A1 | Protein Coding | GC01M027109 | 22.78 |
| CREBBP | CREB Binding Protein | Protein Coding | GC16M003726 | 22.74 |
| COL1A2 | Collagen Type I Alpha 2 Chain | Protein Coding | GC07P094394 | 22.73 |
| MT-CO2 | Mitochondrially Encoded Cytochrome C Oxidase II | Protein Coding | GCMTP007587 | 22.73 |
| SURF1 | SURF1 Cytochrome C Oxidase Assembly Factor | Protein Coding | GC09M133351 | 22.68 |
| SPEG | Striated Muscle Enriched Protein Kinase | Protein Coding | GC02P219434 | 22.66 |
| GYG1 | Glycogenin 1 | Protein Coding | GC03P148991 | 22.59 |
| SBF2 | SET Binding Factor 2 | Protein Coding | GC11M009785 | 22.51 |
| DPP6 | Dipeptidyl Peptidase Like 6 | Protein Coding | GC07P153748 | 22.45 |
| PPP1CB | Protein Phosphatase 1 Catalytic Subunit Beta | Protein Coding | GC02P028752 | 22.45 |
| SOX9 | SRY-Box Transcription Factor 9 | Protein Coding | GC17P072121 | 22.4 |
| TECRL | Trans-2,3-Enoyl-CoA Reductase Like | Protein Coding | GC04M064275 | 22.37 |
| SLC34A3 | Solute Carrier Family 34 Member 3 | Protein Coding | GC09P137230 | 22.37 |
| ACE2 | Angiotensin I Converting Enzyme 2 | Protein Coding | GC0XM015562 | 22.36 |
| CYP21A2 | Cytochrome P450 Family 21 Subfamily A Member 2 | Protein Coding | GC06P047333 | 22.36 |
| BBS5 | Bardet-Biedl Syndrome 5 | Protein Coding | GC02P169480 | 22.34 |
| BBS10 | Bardet-Biedl Syndrome 10 | Protein Coding | GC12M076344 | 22.28 |
| CHDS3 | Coronary Heart Disease, Susceptibility To, 3 | Genetic Locus | GC0XU900293 | 22.26 |
| NOS1AP | Nitric Oxide Synthase 1 Adaptor Protein | Protein Coding | GC01P162069 | 22.25 |
| SPG7 | SPG7 Matrix AAA Peptidase Subunit, Paraplegin | Protein Coding | GC16P089492 | 22.24 |
| CHDS2 | Coronary Heart Disease, Susceptibility To, 2 | Genetic Locus | GC02U900242 | 22.24 |
| PARK7 | Parkinsonism Associated Deglycase | Protein Coding | GC01P007983 | 22.15 |
| MEFV | MEFV Innate Immuity Regulator, Pyrin | Protein Coding | GC16M003281 | 22.11 |
| CYP17A1 | Cytochrome P450 Family 17 Subfamily A Member 1 | Protein Coding | GC10M102830 | 22.09 |
| MT-ND5 | Mitochondrially Encoded NADH:Ubiquinone Oxidoreductase Core Subunit 5 | Protein Coding | GCMTP012339 | 22.03 |
| MAT2A | Methionine Adenosyltransferase 2A | Protein Coding | GC02P085538 | 21.96 |
| SIRT1 | Sirtuin 1 | Protein Coding | GC10P067884 | 21.96 |
| CSF2 | Colony Stimulating Factor 2 | Protein Coding | GC05P132073 | 21.9 |
| PDE3A | Phosphodiesterase 3A | Protein Coding | GC12P020294 | 21.88 |
| PCCA | Propionyl-CoA Carboxylase Subunit Alpha | Protein Coding | GC13P100089 | 21.85 |
| ATXN2 | Ataxin 2 | Protein Coding | GC12M111443 | 21.85 |
| PGF | Placental Growth Factor | Protein Coding | GC14M074941 | 21.83 |
| GUSB | Glucuronidase Beta | Protein Coding | GC07M065960 | 21.81 |
| TIMP2 | TIMP Metallopeptidase Inhibitor 2 | Protein Coding | GC17M078852 | 21.79 |
| SMARCA4 | SWI/SNF Related, Matrix Associated, Actin Dependent Regulator Of Chromatin, Subfamily A, Member 4 | Protein Coding | GC19P010932 | 21.79 |
| CRH | Corticotropin Releasing Hormone | Protein Coding | GC08M066176 | 21.78 |
| HNF1A | HNF1 Homeobox A | Protein Coding | GC12P120978 | 21.78 |
| REST | RE1 Silencing Transcription Factor | Protein Coding | GC04P056907 | 21.77 |
| ERCC4 | ERCC Excision Repair 4, Endonuclease Catalytic Subunit | Protein Coding | GC16P013920 | 21.76 |
| FOXC1 | Forkhead Box C1 | Protein Coding | GC06P001610 | 21.76 |
| NPPC | Natriuretic Peptide C | Protein Coding | GC02M231921 | 21.76 |
| TARDBP | TAR DNA Binding Protein | Protein Coding | GC01P011013 | 21.74 |
| EDN3 | Endothelin 3 | Protein Coding | GC20P059300 | 21.74 |
| ADA | Adenosine Deaminase | Protein Coding | GC20M044620 | 21.74 |
| CEP19 | Centrosomal Protein 19 | Protein Coding | GC03M196706 | 21.73 |
| ANGPTL3 | Angiopoietin Like 3 | Protein Coding | GC01P062597 | 21.73 |
| MIR423 | MicroRNA 423 | RNA Gene | GC17P030117 | 21.72 |
| DOLK | Dolichol Kinase | Protein Coding | GC09M128945 | 21.71 |
| TREX1 | Three Prime Repair Exonuclease 1 | Protein Coding | GC03P048466 | 21.69 |
| CDH23 | Cadherin Related 23 | Protein Coding | GC10P071396 | 21.69 |
| IGF1R | Insulin Like Growth Factor 1 Receptor | Protein Coding | GC15P098648 | 21.69 |
| HMGB1 | High Mobility Group Box 1 | Protein Coding | GC13M030456 | 21.68 |
| MSR1 | Macrophage Scavenger Receptor 1 | Protein Coding | GC08M016107 | 21.64 |
| HAND1 | Heart And Neural Crest Derivatives Expressed 1 | Protein Coding | GC05M154450 | 21.64 |
| SHBG | Sex Hormone Binding Globulin | Protein Coding | GC17P007613 | 21.63 |
| FTO | FTO Alpha-Ketoglutarate Dependent Dioxygenase | Protein Coding | GC16P053737 | 21.61 |
| MT-ND2 | Mitochondrially Encoded NADH:Ubiquinone Oxidoreductase Core Subunit 2 | Protein Coding | GCMTP004472 | 21.59 |
| STAT5B | Signal Transducer And Activator Of Transcription 5B | Protein Coding | GC17M042199 | 21.58 |
| AKT2 | AKT Serine/Threonine Kinase 2 | Protein Coding | GC19M040230 | 21.57 |
| MIF | Macrophage Migration Inhibitory Factor | Protein Coding | GC22P023894 | 21.55 |
| MEG3 | Maternally Expressed 3 | RNA Gene | GC14P104771 | 21.53 |
| PON3 | Paraoxonase 3 | Protein Coding | GC07M095359 | 21.48 |
| UMOD | Uromodulin | Protein Coding | GC16M020344 | 21.48 |
| PRKACA | Protein Kinase CAMP-Activated Catalytic Subunit Alpha | Protein Coding | GC19M014092 | 21.47 |
| HLA-DQA1 | Major Histocompatibility Complex, Class II, DQ Alpha 1 | Protein Coding | GC06P047340 | 21.46 |
| CHDS4 | Coronary Heart Disease, Susceptibility To, 4 | Genetic Locus | GC14U900321 | 21.41 |
| SLC1A2 | Solute Carrier Family 1 Member 2 | Protein Coding | GC11M035272 | 21.41 |
| MRAS | Muscle RAS Oncogene Homolog | Protein Coding | GC03P138347 | 21.41 |
| KCNA5 | Potassium Voltage-Gated Channel Subfamily A Member 5 | Protein Coding | GC12P005043 | 21.41 |
| MIR133B | MicroRNA 133b | RNA Gene | GC06P052148 | 21.38 |
| FHL1 | Four And A Half LIM Domains 1 | Protein Coding | GC0XP136146 | 21.38 |
| LMNB1 | Lamin B1 | Protein Coding | GC05P126776 | 21.34 |
| ACP5 | Acid Phosphatase 5, Tartrate Resistant | Protein Coding | GC19M011574 | 21.34 |
| HADHB | Hydroxyacyl-CoA Dehydrogenase Trifunctional Multienzyme Complex Subunit Beta | Protein Coding | GC02P026243 | 21.33 |
| HLA-DPB1 | Major Histocompatibility Complex, Class II, DP Beta 1 | Protein Coding | GC06P047346 | 21.3 |
| SMC1A | Structural Maintenance Of Chromosomes 1A | Protein Coding | GC0XM053374 | 21.28 |
| CA8 | Carbonic Anhydrase 8 | Protein Coding | GC08M060187 | 21.28 |
| CHDS8 | Coronary Heart Disease, Susceptibility To, 8 | Genetic Locus | GC09U901222 | 21.22 |
| CHDS9 | Coronary Heart Disease, Suscpetibility To, 9 | Genetic Locus | GC08U901174 | 21.22 |
| HBA1 | Hemoglobin Subunit Alpha 1 | Protein Coding | GC16P001370 | 21.15 |
| GCG | Glucagon | Protein Coding | GC02M162142 | 21.15 |
| G6PC | Glucose-6-Phosphatase Catalytic Subunit | Protein Coding | GC17P042900 | 21.14 |
| GPC3 | Glypican 3 | Protein Coding | GC0XM133535 | 21.11 |
| DNMT1 | DNA Methyltransferase 1 | Protein Coding | GC19M010133 | 21.08 |
| GATA3 | GATA Binding Protein 3 | Protein Coding | GC10P008045 | 21.08 |
| PDGFRB | Platelet Derived Growth Factor Receptor Beta | Protein Coding | GC05M150113 | 21.07 |
| ATP2A1 | ATPase Sarcoplasmic/Endoplasmic Reticulum Ca2+ Transporting 1 | Protein Coding | GC16P029020 | 21.02 |
| ARF1 | ADP Ribosylation Factor 1 | Protein Coding | GC01P228082 | 20.93 |
| SLC20A2 | Solute Carrier Family 20 Member 2 | Protein Coding | GC08M042416 | 20.92 |
| BBS12 | Bardet-Biedl Syndrome 12 | Protein Coding | GC04P122702 | 20.91 |
| UTS2 | Urotensin 2 | Protein Coding | GC01M007843 | 20.88 |
| CHDS1 | Coronary Heart Disease, Susceptibility To, 1 | Genetic Locus | GC16U900003 | 20.84 |
| MT-TK | Mitochondrially Encoded TRNA-Lys (AAA/G) | RNA Gene | GCMTP008297 | 20.81 |
| SELENON | Selenoprotein N | Protein Coding | GC01P025800 | 20.79 |
| SLC10A2 | Solute Carrier Family 10 Member 2 | Protein Coding | GC13M103043 | 20.65 |
| FAM20C | FAM20C Golgi Associated Secretory Pathway Kinase | Protein Coding | GC07P000192 | 20.64 |
| CTSD | Cathepsin D | Protein Coding | GC11M001752 | 20.6 |
| CASP1 | Caspase 1 | Protein Coding | GC11M105025 | 20.59 |
| IL6R | Interleukin 6 Receptor | Protein Coding | GC01P154405 | 20.58 |
| WASHC5 | WASH Complex Subunit 5 | Protein Coding | GC08M128215 | 20.57 |
| MT-ND6 | Mitochondrially Encoded NADH:Ubiquinone Oxidoreductase Core Subunit 6 | Protein Coding | GCMTM014151 | 20.57 |
| ENO2 | Enolase 2 | Protein Coding | GC12P006913 | 20.55 |
| ATXN3 | Ataxin 3 | Protein Coding | GC14M094059 | 20.54 |
| WDR35 | WD Repeat Domain 35 | Protein Coding | GC02M019910 | 20.48 |
| CTNNA1 | Catenin Alpha 1 | Protein Coding | GC05P138613 | 20.41 |
| MMP14 | Matrix Metallopeptidase 14 | Protein Coding | GC14P025277 | 20.39 |
| PAPPA | Pappalysin 1 | Protein Coding | GC09P116179 | 20.37 |
| DMP1 | Dentin Matrix Acidic Phosphoprotein 1 | Protein Coding | GC04P087650 | 20.35 |
| MED25 | Mediator Complex Subunit 25 | Protein Coding | GC19P049819 | 20.34 |
| SHOC2 | SHOC2 Leucine Rich Repeat Scaffold Protein | Protein Coding | GC10P110919 | 20.31 |
| PSMA6 | Proteasome 20S Subunit Alpha 6 | Protein Coding | GC14P035278 | 20.31 |
| BCHE | Butyrylcholinesterase | Protein Coding | GC03M165772 | 20.31 |
| TWNK | Twinkle MtDNA Helicase | Protein Coding | GC10P100993 | 20.3 |
| TLR5 | Toll Like Receptor 5 | Protein Coding | GC01M223109 | 20.29 |
| HSPG2 | Heparan Sulfate Proteoglycan 2 | Protein Coding | GC01M021822 | 20.27 |
| LTBP3 | Latent Transforming Growth Factor Beta Binding Protein 3 | Protein Coding | GC11M065538 | 20.27 |
| RHOA | Ras Homolog Family Member A | Protein Coding | GC03M049359 | 20.26 |
| HNF1B | HNF1 Homeobox B | Protein Coding | GC17M037686 | 20.24 |
| MAPK3 | Mitogen-Activated Protein Kinase 3 | Protein Coding | GC16M030117 | 20.13 |
| SERPINA6 | Serpin Family A Member 6 | Protein Coding | GC14M094341 | 20.13 |
| ABCA12 | ATP Binding Cassette Subfamily A Member 12 | Protein Coding | GC02M214931 | 20.11 |
| ABCC2 | ATP Binding Cassette Subfamily C Member 2 | Protein Coding | GC10P099782 | 20.07 |
| HSP90AA1 | Heat Shock Protein 90 Alpha Family Class A Member 1 | Protein Coding | GC14M102080 | 20.07 |
| ARSA | Arylsulfatase A | Protein Coding | GC22M050622 | 20.04 |
| HNRNPA1 | Heterogeneous Nuclear Ribonucleoprotein A1 | Protein Coding | GC12P054280 | 20.02 |
| POMGNT1 | Protein O-Linked Mannose N-Acetylglucosaminyltransferase 1 (Beta 1,2-) | Protein Coding | GC01M046188 | 20.01 |
| GALNT3 | Polypeptide N-Acetylgalactosaminyltransferase 3 | Protein Coding | GC02M165747 | 20.01 |
| CCND1 | Cyclin D1 | Protein Coding | GC11P069641 | 19.99 |
| IFT140 | Intraflagellar Transport 140 | Protein Coding | GC16M001569 | 19.99 |
| TET2 | Tet Methylcytosine Dioxygenase 2 | Protein Coding | GC04P105145 | 19.97 |
| IFNB1 | Interferon Beta 1 | Protein Coding | GC09M021077 | 19.97 |
| PDGFB | Platelet Derived Growth Factor Subunit B | Protein Coding | GC22M045657 | 19.93 |
| COQ2 | Coenzyme Q2, Polyprenyltransferase | Protein Coding | GC04M083261 | 19.91 |
| TSHR | Thyroid Stimulating Hormone Receptor | Protein Coding | GC14P080954 | 19.91 |
| GATA1 | GATA Binding Protein 1 | Protein Coding | GC0XP048786 | 19.86 |
| ESR2 | Estrogen Receptor 2 | Protein Coding | GC14M064084 | 19.84 |
| PPCS | Phosphopantothenoylcysteine Synthetase | Protein Coding | GC01P042456 | 19.81 |
| LMNB2 | Lamin B2 | Protein Coding | GC19M002439 | 19.78 |
| NEK8 | NIMA Related Kinase 8 | Protein Coding | GC17P028725 | 19.78 |
| GCLC | Glutamate-Cysteine Ligase Catalytic Subunit | Protein Coding | GC06M053497 | 19.78 |
| SLC19A3 | Solute Carrier Family 19 Member 3 | Protein Coding | GC02M227685 | 19.78 |
| TWIST1 | Twist Family BHLH Transcription Factor 1 | Protein Coding | GC07M019020 | 19.78 |
| HSPA8 | Heat Shock Protein Family A (Hsp70) Member 8 | Protein Coding | GC11M123057 | 19.77 |
| RRM2B | Ribonucleotide Reductase Regulatory TP53 Inducible Subunit M2B | Protein Coding | GC08M102204 | 19.76 |
| DLD | Dihydrolipoamide Dehydrogenase | Protein Coding | GC07P107890 | 19.76 |
| BCS1L | BCS1 Homolog, Ubiquinol-Cytochrome C Reductase Complex Chaperone | Protein Coding | GC02P218658 | 19.75 |
| CNR1 | Cannabinoid Receptor 1 | Protein Coding | GC06M088139 | 19.72 |
| FLT4 | Fms Related Receptor Tyrosine Kinase 4 | Protein Coding | GC05M180607 | 19.71 |
| TAB2 | TGF-Beta Activated Kinase 1 (MAP3K7) Binding Protein 2 | Protein Coding | GC06P149218 | 19.69 |
| IGFBP3 | Insulin Like Growth Factor Binding Protein 3 | Protein Coding | GC07M045912 | 19.68 |
| NAMPT | Nicotinamide Phosphoribosyltransferase | Protein Coding | GC07M106248 | 19.67 |
| LTBP4 | Latent Transforming Growth Factor Beta Binding Protein 4 | Protein Coding | GC19P040592 | 19.62 |
| RFC2 | Replication Factor C Subunit 2 | Protein Coding | GC07M074231 | 19.59 |
| APC | APC Regulator Of WNT Signaling Pathway | Protein Coding | GC05P112707 | 19.57 |
| CSF1 | Colony Stimulating Factor 1 | Protein Coding | GC01P109911 | 19.56 |
| BTNL2 | Butyrophilin Like 2 | Protein Coding | GC06M032393 | 19.55 |
| KIF6 | Kinesin Family Member 6 | Protein Coding | GC06M042249 | 19.54 |
| PPA2 | Inorganic Pyrophosphatase 2 | Protein Coding | GC04M105369 | 19.54 |
| PHYH | Phytanoyl-CoA 2-Hydroxylase | Protein Coding | GC10M013277 | 19.52 |
| NEB | Nebulin | Protein Coding | GC02M151485 | 19.51 |
| FHL2 | Four And A Half LIM Domains 2 | Protein Coding | GC02M105343 | 19.5 |
| ADCY10 | Adenylate Cyclase 10 | Protein Coding | GC01M167809 | 19.5 |
| ACTN1 | Actinin Alpha 1 | Protein Coding | GC14M068874 | 19.49 |
| HBA2 | Hemoglobin Subunit Alpha 2 | Protein Coding | GC16P001373 | 19.47 |
| NFKB1 | Nuclear Factor Kappa B Subunit 1 | Protein Coding | GC04P102501 | 19.46 |
| CASP9 | Caspase 9 | Protein Coding | GC01M015491 | 19.46 |
| PLAU | Plasminogen Activator, Urokinase | Protein Coding | GC10P073909 | 19.43 |
| DKK1 | Dickkopf WNT Signaling Pathway Inhibitor 1 | Protein Coding | GC10P052314 | 19.43 |
| MUC1 | Mucin 1, Cell Surface Associated | Protein Coding | GC01M155185 | 19.42 |
| CMA1 | Chymase 1 | Protein Coding | GC14M024506 | 19.42 |
| PLA2G6 | Phospholipase A2 Group VI | Protein Coding | GC22M046185 | 19.4 |
| DLL4 | Delta Like Canonical Notch Ligand 4 | Protein Coding | GC15P040929 | 19.4 |
| SEC63 | SEC63 Homolog, Protein Translocation Regulator | Protein Coding | GC06M107867 | 19.39 |
| COL11A2 | Collagen Type XI Alpha 2 Chain | Protein Coding | GC06M033162 | 19.33 |
| CLN3 | CLN3 Lysosomal/Endosomal Transmembrane Protein, Battenin | Protein Coding | GC16M028466 | 19.3 |
| NDUFS2 | NADH:Ubiquinone Oxidoreductase Core Subunit S2 | Protein Coding | GC01P161197 | 19.28 |
| SARDH | Sarcosine Dehydrogenase | Protein Coding | GC09M133663 | 19.26 |
| WFS1 | Wolframin ER Transmembrane Glycoprotein | Protein Coding | GC04P006271 | 19.25 |
| PMS2 | PMS1 Homolog 2, Mismatch Repair System Component | Protein Coding | GC07M005973 | 19.25 |
| LIPE | Lipase E, Hormone Sensitive Type | Protein Coding | GC19M042401 | 19.23 |
| PMM2 | Phosphomannomutase 2 | Protein Coding | GC16P008788 | 19.23 |
| ALPK3 | Alpha Kinase 3 | Protein Coding | GC15P084816 | 19.22 |
| NAGA | Alpha-N-Acetylgalactosaminidase | Protein Coding | GC22M042058 | 19.21 |
| NCF2 | Neutrophil Cytosolic Factor 2 | Protein Coding | GC01M183555 | 19.21 |
| IGFBP1 | Insulin Like Growth Factor Binding Protein 1 | Protein Coding | GC07P046552 | 19.19 |
| TG | Thyroglobulin | Protein Coding | GC08P132866 | 19.17 |
| CTF1 | Cardiotrophin 1 | Protein Coding | GC16P030910 | 19.14 |
| CD14 | CD14 Molecule | Protein Coding | GC05M140631 | 19.14 |
| SLC40A1 | Solute Carrier Family 40 Member 1 | Protein Coding | GC02M189560 | 19.14 |
| RUNX1 | RUNX Family Transcription Factor 1 | Protein Coding | GC21M034787 | 19.14 |
| TFR2 | Transferrin Receptor 2 | Protein Coding | GC07M100620 | 19.12 |
| ERCC1 | ERCC Excision Repair 1, Endonuclease Non-Catalytic Subunit | Protein Coding | GC19M045409 | 19.11 |
| CRYAA | Crystallin Alpha A | Protein Coding | GC21P043169 | 19.08 |
| SFTPC | Surfactant Protein C | Protein Coding | GC08P022156 | 19.08 |
| DPP4 | Dipeptidyl Peptidase 4 | Protein Coding | GC02M161992 | 19.06 |
| RMRP | RNA Component Of Mitochondrial RNA Processing Endoribonuclease | RNA Gene | GC09M035655 | 19.05 |
| CREB1 | CAMP Responsive Element Binding Protein 1 | Protein Coding | GC02P207529 | 19.05 |
| ACAD9 | Acyl-CoA Dehydrogenase Family Member 9 | Protein Coding | GC03P130611 | 19.05 |
| GRK2 | G Protein-Coupled Receptor Kinase 2 | Protein Coding | GC11P067266 | 19.05 |
| SERPIND1 | Serpin Family D Member 1 | Protein Coding | GC22P020836 | 18.98 |
| SLC19A2 | Solute Carrier Family 19 Member 2 | Protein Coding | GC01M169463 | 18.97 |
| TRAPPC9 | Trafficking Protein Particle Complex 9 | Protein Coding | GC08M139728 | 18.94 |
| KAT6B | Lysine Acetyltransferase 6B | Protein Coding | GC10P074827 | 18.94 |
| VEGFC | Vascular Endothelial Growth Factor C | Protein Coding | GC04M176683 | 18.92 |
| ACADS | Acyl-CoA Dehydrogenase Short Chain | Protein Coding | GC12P120843 | 18.89 |
| VDR | Vitamin D Receptor | Protein Coding | GC12M047841 | 18.88 |
| U2AF1 | U2 Small Nuclear RNA Auxiliary Factor 1 | Protein Coding | GC21M043092 | 18.87 |
| CFI | Complement Factor I | Protein Coding | GC04M109740 | 18.87 |
| PTX3 | Pentraxin 3 | Protein Coding | GC03P157436 | 18.87 |
| XIAP | X-Linked Inhibitor Of Apoptosis | Protein Coding | GC0XP123859 | 18.87 |
| MDM2 | MDM2 Proto-Oncogene | Protein Coding | GC12P068808 | 18.86 |
| ITGAL | Integrin Subunit Alpha L | Protein Coding | GC16P030472 | 18.86 |
| PPIG | Peptidylprolyl Isomerase G | Protein Coding | GC02P169584 | 18.84 |
| CCR2 | C-C Motif Chemokine Receptor 2 | Protein Coding | GC03P046356 | 18.84 |
| NDUFB11 | NADH:Ubiquinone Oxidoreductase Subunit B11 | Protein Coding | GC0XM047142 | 18.84 |
| MIR106B | MicroRNA 106b | RNA Gene | GC07M100284 | 18.83 |
| PAX2 | Paired Box 2 | Protein Coding | GC10P100735 | 18.81 |
| NPC1L1 | NPC1 Like Intracellular Cholesterol Transporter 1 | Protein Coding | GC07M044512 | 18.79 |
| AGK | Acylglycerol Kinase | Protein Coding | GC07P141551 | 18.79 |
| CD28 | CD28 Molecule | Protein Coding | GC02P203706 | 18.76 |
| LITAF | Lipopolysaccharide Induced TNF Factor | Protein Coding | GC16M011547 | 18.76 |
| NTRK3 | Neurotrophic Receptor Tyrosine Kinase 3 | Protein Coding | GC15M087859 | 18.75 |
| VIM | Vimentin | Protein Coding | GC10P017227 | 18.74 |
| GNPTG | N-Acetylglucosamine-1-Phosphate Transferase Subunit Gamma | Protein Coding | GC16P001351 | 18.7 |
| HTRA1 | HtrA Serine Peptidase 1 | Protein Coding | GC10P122461 | 18.69 |
| PEX5 | Peroxisomal Biogenesis Factor 5 | Protein Coding | GC12P008247 | 18.69 |
| LRP2 | LDL Receptor Related Protein 2 | Protein Coding | GC02M169127 | 18.64 |
| SYP | Synaptophysin | Protein Coding | GC0XM049187 | 18.64 |
| GLI2 | GLI Family Zinc Finger 2 | Protein Coding | GC02P120735 | 18.63 |
| GCLM | Glutamate-Cysteine Ligase Modifier Subunit | Protein Coding | GC01M093885 | 18.63 |
| CDC42 | Cell Division Cycle 42 | Protein Coding | GC01P022057 | 18.62 |
| ERCC2 | ERCC Excision Repair 2, TFIIH Core Complex Helicase Subunit | Protein Coding | GC19M045349 | 18.61 |
| MLX | MAX Dimerization Protein MLX | Protein Coding | GC17P042567 | 18.61 |
| HLA-C | Major Histocompatibility Complex, Class I, C | Protein Coding | GC06M031272 | 18.59 |
| COX10 | Cytochrome C Oxidase Assembly Factor Heme A:Farnesyltransferase COX10 | Protein Coding | GC17P014069 | 18.56 |
| MYH14 | Myosin Heavy Chain 14 | Protein Coding | GC19P050192 | 18.55 |
| HNRNPA2B1 | Heterogeneous Nuclear Ribonucleoprotein A2/B1 | Protein Coding | GC07M026174 | 18.55 |
| CKM | Creatine Kinase, M-Type | Protein Coding | GC19M045306 | 18.54 |
| ARID1B | AT-Rich Interaction Domain 1B | Protein Coding | GC06P156777 | 18.54 |
| PYGL | Glycogen Phosphorylase L | Protein Coding | GC14M050857 | 18.53 |
| CHIT1 | Chitinase 1 | Protein Coding | GC01M203181 | 18.53 |
| USF1 | Upstream Transcription Factor 1 | Protein Coding | GC01M161039 | 18.51 |
| CS | Citrate Synthase | Protein Coding | GC12M056271 | 18.5 |
| MAOA | Monoamine Oxidase A | Protein Coding | GC0XP043654 | 18.49 |
| RBPJ | Recombination Signal Binding Protein For Immunoglobulin Kappa J Region | Protein Coding | GC04P026165 | 18.49 |
| FLCN | Folliculin | Protein Coding | GC17M017206 | 18.45 |
| TNNI2 | Troponin I2, Fast Skeletal Type | Protein Coding | GC11P001839 | 18.44 |
| TMEM70 | Transmembrane Protein 70 | Protein Coding | GC08P073972 | 18.43 |
| CCM2 | CCM2 Scaffold Protein | Protein Coding | GC07P044999 | 18.42 |
| CDH1 | Cadherin 1 | Protein Coding | GC16P068737 | 18.41 |
| PRPS1 | Phosphoribosyl Pyrophosphate Synthetase 1 | Protein Coding | GC0XP107628 | 18.39 |
| PEX2 | Peroxisomal Biogenesis Factor 2 | Protein Coding | GC08M076980 | 18.38 |
| ZEB2 | Zinc Finger E-Box Binding Homeobox 2 | Protein Coding | GC02M144384 | 18.37 |
| HADH | Hydroxyacyl-CoA Dehydrogenase | Protein Coding | GC04P107989 | 18.36 |
| SCN9A | Sodium Voltage-Gated Channel Alpha Subunit 9 | Protein Coding | GC02M166195 | 18.36 |
| PEX7 | Peroxisomal Biogenesis Factor 7 | Protein Coding | GC06P136822 | 18.35 |
| THBS1 | Thrombospondin 1 | Protein Coding | GC15P039581 | 18.3 |
| PSMB8 | Proteasome 20S Subunit Beta 8 | Protein Coding | GC06M032840 | 18.29 |
| FGF10 | Fibroblast Growth Factor 10 | Protein Coding | GC05M044340 | 18.25 |
| NTRK1 | Neurotrophic Receptor Tyrosine Kinase 1 | Protein Coding | GC01P156786 | 18.25 |
| CCDC151 | Coiled-Coil Domain Containing 151 | Protein Coding | GC19M011420 | 18.25 |
| F2R | Coagulation Factor II Thrombin Receptor | Protein Coding | GC05P076716 | 18.24 |
| SORT1 | Sortilin 1 | Protein Coding | GC01M109310 | 18.24 |
| GH1 | Growth Hormone 1 | Protein Coding | GC17M063917 | 18.19 |
| MYLK2 | Myosin Light Chain Kinase 2 | Protein Coding | GC20P031819 | 18.19 |
| FLNB | Filamin B | Protein Coding | GC03P058008 | 18.18 |
| IL33 | Interleukin 33 | Protein Coding | GC09P006206 | 18.17 |
| SLC4A1 | Solute Carrier Family 4 Member 1 (Diego Blood Group) | Protein Coding | GC17M044282 | 18.16 |
| TLL1 | Tolloid Like 1 | Protein Coding | GC04P165873 | 18.16 |
| PEX12 | Peroxisomal Biogenesis Factor 12 | Protein Coding | GC17M035574 | 18.15 |
| THPO | Thrombopoietin | Protein Coding | GC03M184371 | 18.15 |
| KRIT1 | KRIT1 Ankyrin Repeat Containing | Protein Coding | GC07M092198 | 18.14 |
| SCN3B | Sodium Voltage-Gated Channel Beta Subunit 3 | Protein Coding | GC11M123629 | 18.12 |
| HCRT | Hypocretin Neuropeptide Precursor | Protein Coding | GC17M042185 | 18.11 |
| PIK3CG | Phosphatidylinositol-4,5-Bisphosphate 3-Kinase Catalytic Subunit Gamma | Protein Coding | GC07P106865 | 18.09 |
| IFT172 | Intraflagellar Transport 172 | Protein Coding | GC02M027448 | 18.09 |
| F12 | Coagulation Factor XII | Protein Coding | GC05M177402 | 18.06 |
| NDUFV2 | NADH:Ubiquinone Oxidoreductase Core Subunit V2 | Protein Coding | GC18P009092 | 18.05 |
| RAI1 | Retinoic Acid Induced 1 | Protein Coding | GC17P017682 | 18.05 |
| ANGPT2 | Angiopoietin 2 | Protein Coding | GC08M006499 | 18.05 |
| ITGB1 | Integrin Subunit Beta 1 | Protein Coding | GC10M032900 | 18.01 |
| INPP5E | Inositol Polyphosphate-5-Phosphatase E | Protein Coding | GC09M136428 | 18.01 |
| SUFU | SUFU Negative Regulator Of Hedgehog Signaling | Protein Coding | GC10P102503 | 18 |
| TMEM231 | Transmembrane Protein 231 | Protein Coding | GC16M075536 | 17.99 |
| FANCA | FA Complementation Group A | Protein Coding | GC16M089748 | 17.98 |
| SLC22A4 | Solute Carrier Family 22 Member 4 | Protein Coding | GC05P132294 | 17.97 |
| AIFM1 | Apoptosis Inducing Factor Mitochondria Associated 1 | Protein Coding | GC0XM130129 | 17.96 |
| HTR3A | 5-Hydroxytryptamine Receptor 3A | Protein Coding | GC11P113974 | 17.95 |
| CLCN5 | Chloride Voltage-Gated Channel 5 | Protein Coding | GC0XP049922 | 17.95 |
| FLI1 | Fli-1 Proto-Oncogene, ETS Transcription Factor | Protein Coding | GC11P128686 | 17.95 |
| PEX1 | Peroxisomal Biogenesis Factor 1 | Protein Coding | GC07M092487 | 17.9 |
| MVK | Mevalonate Kinase | Protein Coding | GC12P109573 | 17.89 |
| TPI1 | Triosephosphate Isomerase 1 | Protein Coding | GC12P008208 | 17.88 |
| SCN1A | Sodium Voltage-Gated Channel Alpha Subunit 1 | Protein Coding | GC02M165989 | 17.88 |
| RAD51 | RAD51 Recombinase | Protein Coding | GC15P040694 | 17.87 |
| PEX6 | Peroxisomal Biogenesis Factor 6 | Protein Coding | GC06M042963 | 17.86 |
| HDAC9 | Histone Deacetylase 9 | Protein Coding | GC07P018086 | 17.85 |
| MIR10A | MicroRNA 10a | RNA Gene | GC17M048579 | 17.84 |
| NDUFB8 | NADH:Ubiquinone Oxidoreductase Subunit B8 | Protein Coding | GC10M100523 | 17.84 |
| ACHE | Acetylcholinesterase (Cartwright Blood Group) | Protein Coding | GC07M100889 | 17.82 |
| PLIN1 | Perilipin 1 | Protein Coding | GC15M089664 | 17.8 |
| CHAT | Choline O-Acetyltransferase | Protein Coding | GC10P049609 | 17.79 |
| ANO5 | Anoctamin 5 | Protein Coding | GC11P021799 | 17.77 |
| IL2RB | Interleukin 2 Receptor Subunit Beta | Protein Coding | GC22M037125 | 17.75 |
| ENO1 | Enolase 1 | Protein Coding | GC01M008861 | 17.74 |
| DNMT3A | DNA Methyltransferase 3 Alpha | Protein Coding | GC02M025228 | 17.73 |
| ITGA4 | Integrin Subunit Alpha 4 | Protein Coding | GC02P181456 | 17.71 |
| KCNQ1OT1 | KCNQ1 Opposite Strand/Antisense Transcript 1 | RNA Gene | GC11M002661 | 17.71 |
| SFTPB | Surfactant Protein B | Protein Coding | GC02M085657 | 17.67 |
| C9orf72 | C9orf72-SMCR8 Complex Subunit | Protein Coding | GC09M027539 | 17.67 |
| SAMHD1 | SAM And HD Domain Containing Deoxynucleoside Triphosphate Triphosphohydrolase 1 | Protein Coding | GC20M036890 | 17.64 |
| NDUFS3 | NADH:Ubiquinone Oxidoreductase Core Subunit S3 | Protein Coding | GC11P047567 | 17.63 |
| EPAS1 | Endothelial PAS Domain Protein 1 | Protein Coding | GC02P046293 | 17.62 |
| HSPA1A | Heat Shock Protein Family A (Hsp70) Member 1A | Protein Coding | GC06P047326 | 17.62 |
| CFL2 | Cofilin 2 | Protein Coding | GC14M034706 | 17.59 |
| MMUT | Methylmalonyl-CoA Mutase | Protein Coding | GC06M049430 | 17.59 |
| AOC3 | Amine Oxidase Copper Containing 3 | Protein Coding | GC17P042851 | 17.56 |
| CHGA | Chromogranin A | Protein Coding | GC14P092923 | 17.55 |
| CEP120 | Centrosomal Protein 120 | Protein Coding | GC05M123344 | 17.55 |
| MMP7 | Matrix Metallopeptidase 7 | Protein Coding | GC11M102425 | 17.54 |
| ITPKC | Inositol-Trisphosphate 3-Kinase C | Protein Coding | GC19P040718 | 17.54 |
| TCF7L2 | Transcription Factor 7 Like 2 | Protein Coding | GC10P112950 | 17.47 |
| LGALS2 | Galectin 2 | Protein Coding | GC22M037570 | 17.43 |
| TAGLN | Transgelin | Protein Coding | GC11P117199 | 17.42 |
| GABRA1 | Gamma-Aminobutyric Acid Type A Receptor Subunit Alpha1 | Protein Coding | GC05P161847 | 17.41 |
| RIT1 | Ras Like Without CAAX 1 | Protein Coding | GC01M155897 | 17.41 |
| SETD2 | SET Domain Containing 2, Histone Lysine Methyltransferase | Protein Coding | GC03M047033 | 17.35 |
| NOX4 | NADPH Oxidase 4 | Protein Coding | GC11M089324 | 17.35 |
| BDKRB2 | Bradykinin Receptor B2 | Protein Coding | GC14P096205 | 17.35 |
| ADORA2A | Adenosine A2a Receptor | Protein Coding | GC22P024417 | 17.33 |
| SLC2A1 | Solute Carrier Family 2 Member 1 | Protein Coding | GC01M042925 | 17.32 |
| RNU4ATAC | RNA, U4atac Small Nuclear (U12-Dependent Splicing) | RNA Gene | GC02P121532 | 17.28 |
| HSPB8 | Heat Shock Protein Family B (Small) Member 8 | Protein Coding | GC12P119178 | 17.26 |
| MT-ND3 | Mitochondrially Encoded NADH:Ubiquinone Oxidoreductase Core Subunit 3 | Protein Coding | GCMTP010061 | 17.26 |
| CACNA2D2 | Calcium Voltage-Gated Channel Auxiliary Subunit Alpha2delta 2 | Protein Coding | GC03M050385 | 17.23 |
| PEX10 | Peroxisomal Biogenesis Factor 10 | Protein Coding | GC01M002403 | 17.23 |
| NXN | Nucleoredoxin | Protein Coding | GC17M000799 | 17.23 |
| IKBKG | Inhibitor Of Nuclear Factor Kappa B Kinase Regulatory Subunit Gamma | Protein Coding | GC0XP154541 | 17.22 |
| COL18A1 | Collagen Type XVIII Alpha 1 Chain | Protein Coding | GC21P045405 | 17.22 |
| HSD17B10 | Hydroxysteroid 17-Beta Dehydrogenase 10 | Protein Coding | GC0XM053431 | 17.21 |
| IL12RB1 | Interleukin 12 Receptor Subunit Beta 1 | Protein Coding | GC19M018058 | 17.21 |
| APLNR | Apelin Receptor | Protein Coding | GC11M057233 | 17.19 |
| SETBP1 | SET Binding Protein 1 | Protein Coding | GC18P044680 | 17.19 |
| S100A12 | S100 Calcium Binding Protein A12 | Protein Coding | GC01M153373 | 17.15 |
| GNAQ | G Protein Subunit Alpha Q | Protein Coding | GC09M077716 | 17.15 |
| RAD51C | RAD51 Paralog C | Protein Coding | GC17P058692 | 17.14 |
| CDH5 | Cadherin 5 | Protein Coding | GC16P066366 | 17.12 |
| ABCB4 | ATP Binding Cassette Subfamily B Member 4 | Protein Coding | GC07M087401 | 17.12 |
| ATXN1 | Ataxin 1 | Protein Coding | GC06M016299 | 17.12 |
| PNLIP | Pancreatic Lipase | Protein Coding | GC10P116545 | 17.12 |
| PITX3 | Paired Like Homeodomain 3 | Protein Coding | GC10M102230 | 17.09 |
| APOL1 | Apolipoprotein L1 | Protein Coding | GC22P036253 | 17.08 |
| CIDEC | Cell Death Inducing DFFA Like Effector C | Protein Coding | GC03M009866 | 17.07 |
| HBG2 | Hemoglobin Subunit Gamma 2 | Protein Coding | GC11M005372 | 17.05 |
| PRF1 | Perforin 1 | Protein Coding | GC10M070597 | 17.05 |
| PDCD10 | Programmed Cell Death 10 | Protein Coding | GC03M167683 | 17.04 |
| GP1BB | Glycoprotein Ib Platelet Subunit Beta | Protein Coding | GC22P019722 | 17.04 |
| NEBL | Nebulette | Protein Coding | GC10M020779 | 17.03 |
| SREBF1 | Sterol Regulatory Element Binding Transcription Factor 1 | Protein Coding | GC17M017810 | 17.02 |
| MCTP2 | Multiple C2 And Transmembrane Domain Containing 2 | Protein Coding | GC15P097842 | 17.01 |
| SLC12A3 | Solute Carrier Family 12 Member 3 | Protein Coding | GC16P056865 | 17.01 |
| IRF5 | Interferon Regulatory Factor 5 | Protein Coding | GC07P128937 | 17 |
| BAX | BCL2 Associated X, Apoptosis Regulator | Protein Coding | GC19P048954 | 17 |
| IDH2 | Isocitrate Dehydrogenase (NADP(+)) 2 | Protein Coding | GC15M090083 | 16.96 |
| FTL | Ferritin Light Chain | Protein Coding | GC19P048965 | 16.94 |
| ADORA1 | Adenosine A1 Receptor | Protein Coding | GC01P203090 | 16.92 |
| DSG1 | Desmoglein 1 | Protein Coding | GC18P031318 | 16.9 |
| CACNB4 | Calcium Voltage-Gated Channel Auxiliary Subunit Beta 4 | Protein Coding | GC02M151832 | 16.9 |
| HNF4A | Hepatocyte Nuclear Factor 4 Alpha | Protein Coding | GC20P044355 | 16.86 |
| TWIST2 | Twist Family BHLH Transcription Factor 2 | Protein Coding | GC02P238848 | 16.85 |
| MAX | MYC Associated Factor X | Protein Coding | GC14M065009 | 16.84 |
| BUB1B | BUB1 Mitotic Checkpoint Serine/Threonine Kinase B | Protein Coding | GC15P040161 | 16.84 |
| NDUFV1 | NADH:Ubiquinone Oxidoreductase Core Subunit V1 | Protein Coding | GC11P067632 | 16.83 |
| MMP12 | Matrix Metallopeptidase 12 | Protein Coding | GC11M102862 | 16.82 |
| ABHD5 | Abhydrolase Domain Containing 5, Lysophosphatidic Acid Acyltransferase | Protein Coding | GC03P043707 | 16.82 |
| HMGCL | 3-Hydroxy-3-Methylglutaryl-CoA Lyase | Protein Coding | GC01M023801 | 16.81 |
| IBSP | Integrin Binding Sialoprotein | Protein Coding | GC04P087799 | 16.8 |
| OPA1 | OPA1 Mitochondrial Dynamin Like GTPase | Protein Coding | GC03P193594 | 16.79 |
| FABP4 | Fatty Acid Binding Protein 4 | Protein Coding | GC08M081478 | 16.77 |
| STRA6 | Signaling Receptor And Transporter Of Retinol STRA6 | Protein Coding | GC15M074179 | 16.77 |
| IL3 | Interleukin 3 | Protein Coding | GC05P132060 | 16.76 |
| POLG2 | DNA Polymerase Gamma 2, Accessory Subunit | Protein Coding | GC17M064477 | 16.76 |
| UCP2 | Uncoupling Protein 2 | Protein Coding | GC11M073974 | 16.75 |
| PEX3 | Peroxisomal Biogenesis Factor 3 | Protein Coding | GC06P143450 | 16.73 |
| HSD11B2 | Hydroxysteroid 11-Beta Dehydrogenase 2 | Protein Coding | GC16P067433 | 16.73 |
| MMP8 | Matrix Metallopeptidase 8 | Protein Coding | GC11M102617 | 16.72 |
| CD163 | CD163 Molecule | Protein Coding | GC12M007471 | 16.71 |
| CHI3L1 | Chitinase 3 Like 1 | Protein Coding | GC01M203148 | 16.71 |
| AARS2 | Alanyl-TRNA Synthetase 2, Mitochondrial | Protein Coding | GC06M044297 | 16.7 |
| CKB | Creatine Kinase B | Protein Coding | GC14M103519 | 16.7 |
| AMPD1 | Adenosine Monophosphate Deaminase 1 | Protein Coding | GC01M114673 | 16.69 |
| NFATC1 | Nuclear Factor Of Activated T Cells 1 | Protein Coding | GC18P079395 | 16.67 |
| NDUFA13 | NADH:Ubiquinone Oxidoreductase Subunit A13 | Protein Coding | GC19P019515 | 16.66 |
| PRL | Prolactin | Protein Coding | GC06M022230 | 16.66 |
| HIRA | Histone Cell Cycle Regulator | Protein Coding | GC22M019318 | 16.65 |
| LAMB2 | Laminin Subunit Beta 2 | Protein Coding | GC03M049121 | 16.64 |
| RNF213 | Ring Finger Protein 213 | Protein Coding | GC17P080260 | 16.6 |
| SEMA3A | Semaphorin 3A | Protein Coding | GC07M083955 | 16.58 |
| SOCS1 | Suppressor Of Cytokine Signaling 1 | Protein Coding | GC16M011255 | 16.57 |
| IFT88 | Intraflagellar Transport 88 | Protein Coding | GC13P020566 | 16.56 |
| NDUFAF1 | NADH:Ubiquinone Oxidoreductase Complex Assembly Factor 1 | Protein Coding | GC15M041387 | 16.55 |
| IL1R1 | Interleukin 1 Receptor Type 1 | Protein Coding | GC02P102136 | 16.53 |
| CPT1A | Carnitine Palmitoyltransferase 1A | Protein Coding | GC11M068754 | 16.5 |
| CD59 | CD59 Molecule (CD59 Blood Group) | Protein Coding | GC11M033704 | 16.47 |
| SLC6A2 | Solute Carrier Family 6 Member 2 | Protein Coding | GC16P055656 | 16.46 |
| CD46 | CD46 Molecule | Protein Coding | GC01P207752 | 16.46 |
| SKI | SKI Proto-Oncogene | Protein Coding | GC01P002228 | 16.46 |
| STS | Steroid Sulfatase | Protein Coding | GC0XP007146 | 16.45 |
| KLF5 | Kruppel Like Factor 5 | Protein Coding | GC13P073054 | 16.45 |
| POMT1 | Protein O-Mannosyltransferase 1 | Protein Coding | GC09P131502 | 16.44 |
| MIPEP | Mitochondrial Intermediate Peptidase | Protein Coding | GC13M023730 | 16.44 |
| TK2 | Thymidine Kinase 2 | Protein Coding | GC16M066508 | 16.43 |
| IL12A | Interleukin 12A | Protein Coding | GC03P159988 | 16.43 |
| PEX26 | Peroxisomal Biogenesis Factor 26 | Protein Coding | GC22P018079 | 16.43 |
| S100A1 | S100 Calcium Binding Protein A1 | Protein Coding | GC01P153627 | 16.42 |
| PEX19 | Peroxisomal Biogenesis Factor 19 | Protein Coding | GC01M160276 | 16.41 |
| MIR208B | MicroRNA 208b | RNA Gene | GC14M023417 | 16.41 |
| NDUFS1 | NADH:Ubiquinone Oxidoreductase Core Subunit S1 | Protein Coding | GC02M206114 | 16.4 |
| NDUFA1 | NADH:Ubiquinone Oxidoreductase Subunit A1 | Protein Coding | GC0XP119871 | 16.39 |
| MSH2 | MutS Homolog 2 | Protein Coding | GC02P047402 | 16.38 |
| RPS19 | Ribosomal Protein S19 | Protein Coding | GC19P041859 | 16.37 |
| BCL2L1 | BCL2 Like 1 | Protein Coding | GC20M031664 | 16.35 |
| CBL | Cbl Proto-Oncogene | Protein Coding | GC11P119206 | 16.34 |
| MIR132 | MicroRNA 132 | RNA Gene | GC17M002049 | 16.34 |
| LMOD1 | Leiomodin 1 | Protein Coding | GC01M201896 | 16.31 |
| CYP27A1 | Cytochrome P450 Family 27 Subfamily A Member 1 | Protein Coding | GC02P218781 | 16.31 |
| ALG6 | ALG6 Alpha-1,3-Glucosyltransferase | Protein Coding | GC01P063367 | 16.3 |
| DUOX2 | Dual Oxidase 2 | Protein Coding | GC15M045092 | 16.3 |
| SRA1 | Steroid Receptor RNA Activator 1 | Protein Coding | GC05M140537 | 16.29 |
| CRAT | Carnitine O-Acetyltransferase | Protein Coding | GC09M129094 | 16.29 |
| SST | Somatostatin | Protein Coding | GC03M187668 | 16.29 |
| LEMD3 | LEM Domain Containing 3 | Protein Coding | GC12P065169 | 16.28 |
| UFD1 | Ubiquitin Recognition Factor In ER Associated Degradation 1 | Protein Coding | GC22M019450 | 16.27 |
| BGLAP | Bone Gamma-Carboxyglutamate Protein | Protein Coding | GC01P156242 | 16.26 |
| PEX13 | Peroxisomal Biogenesis Factor 13 | Protein Coding | GC02P061017 | 16.23 |
| SMAD2 | SMAD Family Member 2 | Protein Coding | GC18M047809 | 16.21 |
| PROCR | Protein C Receptor | Protein Coding | GC20P035182 | 16.21 |
| ABCB11 | ATP Binding Cassette Subfamily B Member 11 | Protein Coding | GC02M168922 | 16.21 |
| NDUFAF3 | NADH:Ubiquinone Oxidoreductase Complex Assembly Factor 3 | Protein Coding | GC03P049020 | 16.2 |
| PEX11B | Peroxisomal Biogenesis Factor 11 Beta | Protein Coding | GC01M145911 | 16.19 |
| PROC | Protein C, Inactivator Of Coagulation Factors Va And VIIIa | Protein Coding | GC02P127418 | 16.17 |
| IL7R | Interleukin 7 Receptor | Protein Coding | GC05P035852 | 16.17 |
| CCT7 | Chaperonin Containing TCP1 Subunit 7 | Protein Coding | GC02P073233 | 16.16 |
| EPG5 | Ectopic P-Granules Autophagy Protein 5 Homolog | Protein Coding | GC18M045800 | 16.15 |
| FANCD2 | FA Complementation Group D2 | Protein Coding | GC03P010026 | 16.14 |
| CCR1 | C-C Motif Chemokine Receptor 1 | Protein Coding | GC03M046218 | 16.12 |
| SULT1A3 | Sulfotransferase Family 1A Member 3 | Protein Coding | GC16P030199 | 16.12 |
| TYMP | Thymidine Phosphorylase | Protein Coding | GC22M050525 | 16.11 |
| GSTM1 | Glutathione S-Transferase Mu 1 | Protein Coding | GC01P109687 | 16.1 |
| HPD | 4-Hydroxyphenylpyruvate Dioxygenase | Protein Coding | GC12M121839 | 16.1 |
| CD86 | CD86 Molecule | Protein Coding | GC03P122055 | 16.08 |
| GNE | Glucosamine (UDP-N-Acetyl)-2-Epimerase/N-Acetylmannosamine Kinase | Protein Coding | GC09M036214 | 16.07 |
| NPHS1 | NPHS1 Adhesion Molecule, Nephrin | Protein Coding | GC19M035825 | 16.05 |
| TTPA | Alpha Tocopherol Transfer Protein | Protein Coding | GC08M063048 | 16.02 |
| SOS2 | SOS Ras/Rho Guanine Nucleotide Exchange Factor 2 | Protein Coding | GC14M050117 | 16.02 |
| IL17F | Interleukin 17F | Protein Coding | GC06M052209 | 16.01 |
| GNA11 | G Protein Subunit Alpha 11 | Protein Coding | GC19P003094 | 16 |
| LAMA3 | Laminin Subunit Alpha 3 | Protein Coding | GC18P023689 | 16 |
| PEX14 | Peroxisomal Biogenesis Factor 14 | Protein Coding | GC01P010472 | 15.98 |
| ADRA2C | Adrenoceptor Alpha 2C | Protein Coding | GC04P003766 | 15.97 |
| OXT | Oxytocin/Neurophysin I Prepropeptide | Protein Coding | GC20P003068 | 15.93 |
| NDUFAF2 | NADH:Ubiquinone Oxidoreductase Complex Assembly Factor 2 | Protein Coding | GC05P060945 | 15.92 |
| JUN | Jun Proto-Oncogene, AP-1 Transcription Factor Subunit | Protein Coding | GC01M058780 | 15.92 |
| CYP11B1 | Cytochrome P450 Family 11 Subfamily B Member 1 | Protein Coding | GC08M142872 | 15.91 |
| OPTN | Optineurin | Protein Coding | GC10P013099 | 15.86 |
| NDUFS7 | NADH:Ubiquinone Oxidoreductase Core Subunit S7 | Protein Coding | GC19P001490 | 15.86 |
| CTSK | Cathepsin K | Protein Coding | GC01M150796 | 15.86 |
| FKBP1B | FKBP Prolyl Isomerase 1B | Protein Coding | GC02P024033 | 15.84 |
| FANCG | FA Complementation Group G | Protein Coding | GC09M035073 | 15.84 |
| FANCB | FA Complementation Group B | Protein Coding | GC0XM014690 | 15.84 |
| COL4A2 | Collagen Type IV Alpha 2 Chain | Protein Coding | GC13P110305 | 15.81 |
| CTSB | Cathepsin B | Protein Coding | GC08M011842 | 15.8 |
| PCCB | Propionyl-CoA Carboxylase Subunit Beta | Protein Coding | GC03P136250 | 15.79 |
| EGLN1 | Egl-9 Family Hypoxia Inducible Factor 1 | Protein Coding | GC01M231363 | 15.79 |
| NDUFB9 | NADH:Ubiquinone Oxidoreductase Subunit B9 | Protein Coding | GC08P124539 | 15.78 |
| KCNMA1 | Potassium Calcium-Activated Channel Subfamily M Alpha 1 | Protein Coding | GC10M076869 | 15.77 |
| ATP6AP2 | ATPase H+ Transporting Accessory Protein 2 | Protein Coding | GC0XP040582 | 15.77 |
| DSCAM | DS Cell Adhesion Molecule | Protein Coding | GC21M040010 | 15.76 |
| GRN | Granulin Precursor | Protein Coding | GC17P044345 | 15.75 |
| VTN | Vitronectin | Protein Coding | GC17M029955 | 15.74 |
| UGT1A1 | UDP Glucuronosyltransferase Family 1 Member A1 | Protein Coding | GC02P233760 | 15.73 |
| POMT2 | Protein O-Mannosyltransferase 2 | Protein Coding | GC14M077274 | 15.73 |
| ERCC8 | ERCC Excision Repair 8, CSA Ubiquitin Ligase Complex Subunit | Protein Coding | GC05M060868 | 15.71 |
| RASA2 | RAS P21 Protein Activator 2 | Protein Coding | GC03P141487 | 15.71 |
| DNAH11 | Dynein Axonemal Heavy Chain 11 | Protein Coding | GC07P021543 | 15.7 |
| MPV17 | Mitochondrial Inner Membrane Protein MPV17 | Protein Coding | GC02M027309 | 15.69 |
| COL7A1 | Collagen Type VII Alpha 1 Chain | Protein Coding | GC03M048564 | 15.69 |
| HCCS | Holocytochrome C Synthase | Protein Coding | GC0XP011111 | 15.69 |
| FCGR3B | Fc Fragment Of IgG Receptor IIIb | Protein Coding | GC01M161623 | 15.68 |
| SOCS3 | Suppressor Of Cytokine Signaling 3 | Protein Coding | GC17M078356 | 15.65 |
| STK11 | Serine/Threonine Kinase 11 | Protein Coding | GC19P001177 | 15.64 |
| ITGB6 | Integrin Subunit Beta 6 | Protein Coding | GC02M160099 | 15.63 |
| PEX16 | Peroxisomal Biogenesis Factor 16 | Protein Coding | GC11M061098 | 15.63 |
| PALB2 | Partner And Localizer Of BRCA2 | Protein Coding | GC16M023603 | 15.63 |
| OCRL | OCRL Inositol Polyphosphate-5-Phosphatase | Protein Coding | GC0XP129539 | 15.63 |
| CLCN2 | Chloride Voltage-Gated Channel 2 | Protein Coding | GC03M184346 | 15.62 |
| IFNA1 | Interferon Alpha 1 | Protein Coding | GC09P021494 | 15.62 |
| MARS1 | Methionyl-TRNA Synthetase 1 | Protein Coding | GC12P057476 | 15.61 |
| PAX6 | Paired Box 6 | Protein Coding | GC11M031784 | 15.61 |
| ERBB3 | Erb-B2 Receptor Tyrosine Kinase 3 | Protein Coding | GC12P056094 | 15.58 |
| PLTP | Phospholipid Transfer Protein | Protein Coding | GC20M045898 | 15.56 |
| GNRH1 | Gonadotropin Releasing Hormone 1 | Protein Coding | GC08M025419 | 15.56 |
| EPHB4 | EPH Receptor B4 | Protein Coding | GC07M100803 | 15.56 |
| YARS2 | Tyrosyl-TRNA Synthetase 2 | Protein Coding | GC12M032725 | 15.56 |
| ADAM10 | ADAM Metallopeptidase Domain 10 | Protein Coding | GC15M058588 | 15.54 |
| IL5 | Interleukin 5 | Protein Coding | GC05M132541 | 15.53 |
| GJC2 | Gap Junction Protein Gamma 2 | Protein Coding | GC01P228175 | 15.52 |
| SRF | Serum Response Factor | Protein Coding | GC06P043171 | 15.52 |
| TRIP4 | Thyroid Hormone Receptor Interactor 4 | Protein Coding | GC15P072864 | 15.52 |
| SCNN1A | Sodium Channel Epithelial 1 Subunit Alpha | Protein Coding | GC12M006346 | 15.51 |
| NDUFS8 | NADH:Ubiquinone Oxidoreductase Core Subunit S8 | Protein Coding | GC11P068030 | 15.51 |
| DRD5 | Dopamine Receptor D5 | Protein Coding | GC04P009783 | 15.5 |
| CHST3 | Carbohydrate Sulfotransferase 3 | Protein Coding | GC10P071964 | 15.5 |
| ABO | ABO, Alpha 1-3-N-Acetylgalactosaminyltransferase And Alpha 1-3-Galactosyltransferase | Protein Coding | GC09M133250 | 15.5 |
| HTR1B | 5-Hydroxytryptamine Receptor 1B | Protein Coding | GC06M077478 | 15.49 |
| COX6B1 | Cytochrome C Oxidase Subunit 6B1 | Protein Coding | GC19P038224 | 15.49 |
| THBS4 | Thrombospondin 4 | Protein Coding | GC05P079991 | 15.45 |
| COX15 | Cytochrome C Oxidase Assembly Homolog COX15 | Protein Coding | GC10M099696 | 15.43 |
| FOXRED1 | FAD Dependent Oxidoreductase Domain Containing 1 | Protein Coding | GC11P126269 | 15.4 |
| MLYCD | Malonyl-CoA Decarboxylase | Protein Coding | GC16P083899 | 15.39 |
| TFPI | Tissue Factor Pathway Inhibitor | Protein Coding | GC02M187464 | 15.38 |
| NPR1 | Natriuretic Peptide Receptor 1 | Protein Coding | GC01P153697 | 15.37 |
| TNXB | Tenascin XB | Protein Coding | GC06M032635 | 15.35 |
| MMP21 | Matrix Metallopeptidase 21 | Protein Coding | GC10M125766 | 15.34 |
| TPM4 | Tropomyosin 4 | Protein Coding | GC19P023264 | 15.34 |
| CR1 | Complement C3b/C4b Receptor 1 (Knops Blood Group) | Protein Coding | GC01P207496 | 15.3 |
| CTCF | CCCTC-Binding Factor | Protein Coding | GC16P067563 | 15.3 |
| NUBPL | Nucleotide Binding Protein Like | Protein Coding | GC14P031489 | 15.29 |
| HELLS | Helicase, Lymphoid Specific | Protein Coding | GC10P094501 | 15.29 |
| TNFAIP3 | TNF Alpha Induced Protein 3 | Protein Coding | GC06P137866 | 15.27 |
| COCH | Cochlin | Protein Coding | GC14P030874 | 15.27 |
| AKT3 | AKT Serine/Threonine Kinase 3 | Protein Coding | GC01M243488 | 15.27 |
| KCNE5 | Potassium Voltage-Gated Channel Subfamily E Regulatory Subunit 5 | Protein Coding | GC0XM109623 | 15.27 |
| CAPN3 | Calpain 3 | Protein Coding | GC15P042359 | 15.26 |
| NDUFA12 | NADH:Ubiquinone Oxidoreductase Subunit A12 | Protein Coding | GC12M094898 | 15.25 |
| KRT5 | Keratin 5 | Protein Coding | GC12M052514 | 15.24 |
| ARID1A | AT-Rich Interaction Domain 1A | Protein Coding | GC01P026693 | 15.23 |
| MC2R | Melanocortin 2 Receptor | Protein Coding | GC18M017331 | 15.23 |
| RNLS | Renalase, FAD Dependent Amine Oxidase | Protein Coding | GC10M088180 | 15.21 |
| FANCC | FA Complementation Group C | Protein Coding | GC09M095099 | 15.18 |
| DPM1 | Dolichyl-Phosphate Mannosyltransferase Subunit 1, Catalytic | Protein Coding | GC20M050934 | 15.17 |
| IL7 | Interleukin 7 | Protein Coding | GC08M078689 | 15.16 |
| TMEM126B | Transmembrane Protein 126B | Protein Coding | GC11P085628 | 15.16 |
| KCNJ1 | Potassium Inwardly Rectifying Channel Subfamily J Member 1 | Protein Coding | GC11M128741 | 15.16 |
| SREBF2 | Sterol Regulatory Element Binding Transcription Factor 2 | Protein Coding | GC22P041833 | 15.16 |
| HEY2 | Hes Related Family BHLH Transcription Factor With YRPW Motif 2 | Protein Coding | GC06P125730 | 15.16 |
| GJA4 | Gap Junction Protein Alpha 4 | Protein Coding | GC01P034792 | 15.13 |
| ADRA2A | Adrenoceptor Alpha 2A | Protein Coding | GC10P111077 | 15.12 |
| B9D2 | B9 Domain Containing 2 | Protein Coding | GC19M041354 | 15.12 |
| NDUFA9 | NADH:Ubiquinone Oxidoreductase Subunit A9 | Protein Coding | GC12P004649 | 15.1 |
| WDTC1 | WD And Tetratricopeptide Repeats 1 | Protein Coding | GC01P027245 | 15.1 |
| NDUFAF6 | NADH:Ubiquinone Oxidoreductase Complex Assembly Factor 6 | Protein Coding | GC08P094895 | 15.09 |
| NTRK2 | Neurotrophic Receptor Tyrosine Kinase 2 | Protein Coding | GC09P084668 | 15.06 |
| STAR | Steroidogenic Acute Regulatory Protein | Protein Coding | GC08M038145 | 15.05 |
| P2RX7 | Purinergic Receptor P2X 7 | Protein Coding | GC12P122829 | 15.05 |
| ETS1 | ETS Proto-Oncogene 1, Transcription Factor | Protein Coding | GC11M128458 | 15.05 |
| SP1 | Sp1 Transcription Factor | Protein Coding | GC12P053380 | 15.03 |
| SLMAP | Sarcolemma Associated Protein | Protein Coding | GC03P057767 | 15.02 |
| PLA2G2A | Phospholipase A2 Group IIA | Protein Coding | GC01M019975 | 15.02 |
| FANCI | FA Complementation Group I | Protein Coding | GC15P089243 | 14.99 |
| NHLRC1 | NHL Repeat Containing E3 Ubiquitin Protein Ligase 1 | Protein Coding | GC06M018065 | 14.99 |
| MIR9-1 | MicroRNA 9-1 | RNA Gene | GC01M156420 | 14.98 |
| FAT4 | FAT Atypical Cadherin 4 | Protein Coding | GC04P125315 | 14.98 |
| MHRT | Myosin Heavy Chain Associated RNA Transcript | RNA Gene | GC14P025365 | 14.96 |
| CHKA | Choline Kinase Alpha | Protein Coding | GC11M068052 | 14.95 |
| GCH1 | GTP Cyclohydrolase 1 | Protein Coding | GC14M054842 | 14.95 |
| CDKN1B | Cyclin Dependent Kinase Inhibitor 1B | Protein Coding | GC12P012716 | 14.95 |
| PRDM6 | PR/SET Domain 6 | Protein Coding | GC05P123089 | 14.93 |
| NDUFA6 | NADH:Ubiquinone Oxidoreductase Subunit A6 | Protein Coding | GC22M042085 | 14.92 |
| SKIV2L | Ski2 Like RNA Helicase | Protein Coding | GC06P047331 | 14.92 |
| ECHS1 | Enoyl-CoA Hydratase, Short Chain 1 | Protein Coding | GC10M133362 | 14.9 |
| BMPR1B | Bone Morphogenetic Protein Receptor Type 1B | Protein Coding | GC04P094757 | 14.9 |
| TLR8 | Toll Like Receptor 8 | Protein Coding | GC0XP012924 | 14.88 |
| NDUFB3 | NADH:Ubiquinone Oxidoreductase Subunit B3 | Protein Coding | GC02P201071 | 14.87 |
| EZH2 | Enhancer Of Zeste 2 Polycomb Repressive Complex 2 Subunit | Protein Coding | GC07M148807 | 14.87 |
| TGIF1 | TGFB Induced Factor Homeobox 1 | Protein Coding | GC18P003411 | 14.86 |
| SLC26A4 | Solute Carrier Family 26 Member 4 | Protein Coding | GC07P107660 | 14.86 |
| GTPBP3 | GTP Binding Protein 3, Mitochondrial | Protein Coding | GC19P023303 | 14.85 |
| BRD4 | Bromodomain Containing 4 | Protein Coding | GC19M015236 | 14.83 |
| A2ML1 | Alpha-2-Macroglobulin Like 1 | Protein Coding | GC12P008822 | 14.83 |
| IRF1 | Interferon Regulatory Factor 1 | Protein Coding | GC05M132481 | 14.82 |
| SLC25A3 | Solute Carrier Family 25 Member 3 | Protein Coding | GC12P098593 | 14.82 |
| KCNAB2 | Potassium Voltage-Gated Channel Subfamily A Regulatory Beta Subunit 2 | Protein Coding | GC01P006020 | 14.8 |
| ARSH | Arylsulfatase Family Member H | Protein Coding | GC0XP003006 | 14.8 |
| RTEL1 | Regulator Of Telomere Elongation Helicase 1 | Protein Coding | GC20P063658 | 14.8 |
| HES7 | Hes Family BHLH Transcription Factor 7 | Protein Coding | GC17M008120 | 14.79 |
| MT-TS1 | Mitochondrially Encoded TRNA-Ser (UCN) 1 | RNA Gene | GCMTM007447 | 14.79 |
| NDUFA10 | NADH:Ubiquinone Oxidoreductase Subunit A10 | Protein Coding | GC02M239893 | 14.75 |
| RELA | RELA Proto-Oncogene, NF-KB Subunit | Protein Coding | GC11M065653 | 14.73 |
| LIMS2 | LIM Zinc Finger Domain Containing 2 | Protein Coding | GC02M127638 | 14.71 |
| TBK1 | TANK Binding Kinase 1 | Protein Coding | GC12P064451 | 14.71 |
| PUF60 | Poly(U) Binding Splicing Factor 60 | Protein Coding | GC08M143816 | 14.68 |
| STX1A | Syntaxin 1A | Protein Coding | GC07M073700 | 14.67 |
| XPA | XPA, DNA Damage Recognition And Repair Factor | Protein Coding | GC09M097635 | 14.65 |
| SOST | Sclerostin | Protein Coding | GC17M043753 | 14.65 |
| KCNB1 | Potassium Voltage-Gated Channel Subfamily B Member 1 | Protein Coding | GC20M049293 | 14.65 |
| TLR9 | Toll Like Receptor 9 | Protein Coding | GC03M052222 | 14.65 |
| AHDC1 | AT-Hook DNA Binding Motif Containing 1 | Protein Coding | GC01M027546 | 14.64 |
| ADAR | Adenosine Deaminase RNA Specific | Protein Coding | GC01M154582 | 14.64 |
| MT-TS2 | Mitochondrially Encoded TRNA-Ser (AGU/C) 2 | RNA Gene | GCMTP012215 | 14.63 |
| NDUFAF5 | NADH:Ubiquinone Oxidoreductase Complex Assembly Factor 5 | Protein Coding | GC20P013786 | 14.63 |
| PDLIM1 | PDZ And LIM Domain 1 | Protein Coding | GC10M095237 | 14.62 |
| MAPK8IP1 | Mitogen-Activated Protein Kinase 8 Interacting Protein 1 | Protein Coding | GC11P045988 | 14.62 |
| SCNN1B | Sodium Channel Epithelial 1 Subunit Beta | Protein Coding | GC16P023278 | 14.61 |
| PHOX2B | Paired Like Homeobox 2B | Protein Coding | GC04M041746 | 14.61 |
| ATP1A1 | ATPase Na+/K+ Transporting Subunit Alpha 1 | Protein Coding | GC01P116372 | 14.6 |
| VAC14 | VAC14 Component Of PIKFYVE Complex | Protein Coding | GC16M070688 | 14.58 |
| EBP | EBP Cholestenol Delta-Isomerase | Protein Coding | GC0XP048521 | 14.58 |
| HOTAIR | HOX Transcript Antisense RNA | RNA Gene | GC12M053962 | 14.57 |
| VCAN | Versican | Protein Coding | GC05P083471 | 14.56 |
| FOXH1 | Forkhead Box H1 | Protein Coding | GC08M144473 | 14.56 |
| MIB1 | Mindbomb E3 Ubiquitin Protein Ligase 1 | Protein Coding | GC18P021704 | 14.55 |
| TMEM260 | Transmembrane Protein 260 | Protein Coding | GC14P056488 | 14.55 |
| CD79A | CD79a Molecule | Protein Coding | GC19P041877 | 14.55 |
| FKBP10 | FKBP Prolyl Isomerase 10 | Protein Coding | GC17P041812 | 14.54 |
| SPECC1L | Sperm Antigen With Calponin Homology And Coiled-Coil Domains 1 Like | Protein Coding | GC22P024837 | 14.54 |
| DOCK6 | Dedicator Of Cytokinesis 6 | Protein Coding | GC19M011172 | 14.54 |
| MIR124-1 | MicroRNA 124-1 | RNA Gene | GC08M009903 | 14.52 |
| GAS5 | Growth Arrest Specific 5 | RNA Gene | GC01M173947 | 14.51 |
| MYCN | MYCN Proto-Oncogene, BHLH Transcription Factor | Protein Coding | GC02P015949 | 14.51 |
| COX7B | Cytochrome C Oxidase Subunit 7B | Protein Coding | GC0XP077899 | 14.5 |
| CXCL10 | C-X-C Motif Chemokine Ligand 10 | Protein Coding | GC04M076021 | 14.5 |
| FANCF | FA Complementation Group F | Protein Coding | GC11M022600 | 14.49 |
| ANKRD11 | Ankyrin Repeat Domain 11 | Protein Coding | GC16M089267 | 14.49 |
| ARVCF | ARVCF Delta Catenin Family Member | Protein Coding | GC22M019966 | 14.49 |
| MIR133A1 | MicroRNA 133a-1 | RNA Gene | GC18M021826 | 14.47 |
| ERBB4 | Erb-B2 Receptor Tyrosine Kinase 4 | Protein Coding | GC02M211375 | 14.46 |
| GPC4 | Glypican 4 | Protein Coding | GC0XM133300 | 14.45 |
| NEDD4L | NEDD4 Like E3 Ubiquitin Protein Ligase | Protein Coding | GC18P058044 | 14.45 |
| PDSS2 | Decaprenyl Diphosphate Synthase Subunit 2 | Protein Coding | GC06M107152 | 14.43 |
| CDKN3 | Cyclin Dependent Kinase Inhibitor 3 | Protein Coding | GC14P054398 | 14.43 |
| RBCK1 | RANBP2-Type And C3HC4-Type Zinc Finger Containing 1 | Protein Coding | GC20P000407 | 14.42 |
| ABCB7 | ATP Binding Cassette Subfamily B Member 7 | Protein Coding | GC0XM075053 | 14.42 |
| NDUFS6 | NADH:Ubiquinone Oxidoreductase Subunit S6 | Protein Coding | GC05P001801 | 14.38 |
| KCNIP2 | Potassium Voltage-Gated Channel Interacting Protein 2 | Protein Coding | GC10M101825 | 14.38 |
| CRKL | CRK Like Proto-Oncogene, Adaptor Protein | Protein Coding | GC22P020917 | 14.37 |
| GABRD | Gamma-Aminobutyric Acid Type A Receptor Subunit Delta | Protein Coding | GC01P002019 | 14.37 |
| FANCL | FA Complementation Group L | Protein Coding | GC02M058127 | 14.36 |
| ATP5F1E | ATP Synthase F1 Subunit Epsilon | Protein Coding | GC20M059026 | 14.36 |
| VPS13A | Vacuolar Protein Sorting 13 Homolog A | Protein Coding | GC09P077177 | 14.36 |
| CHRM2 | Cholinergic Receptor Muscarinic 2 | Protein Coding | GC07P136868 | 14.34 |
| M6PR | Mannose-6-Phosphate Receptor, Cation Dependent | Protein Coding | GC12M008955 | 14.34 |
| KLF1 | Kruppel Like Factor 1 | Protein Coding | GC19M012884 | 14.33 |
| CYP27B1 | Cytochrome P450 Family 27 Subfamily B Member 1 | Protein Coding | GC12M057757 | 14.31 |
| C5 | Complement C5 | Protein Coding | GC09M120952 | 14.3 |
| SLC39A8 | Solute Carrier Family 39 Member 8 | Protein Coding | GC04M102252 | 14.27 |
| IKBKB | Inhibitor Of Nuclear Factor Kappa B Kinase Subunit Beta | Protein Coding | GC08P042271 | 14.25 |
| MTFMT | Mitochondrial Methionyl-TRNA Formyltransferase | Protein Coding | GC15M065001 | 14.25 |
| HLA-G | Major Histocompatibility Complex, Class I, G | Protein Coding | GC06P047256 | 14.25 |
| MERTK | MER Proto-Oncogene, Tyrosine Kinase | Protein Coding | GC02P111898 | 14.24 |
| NLRP1 | NLR Family Pyrin Domain Containing 1 | Protein Coding | GC17M005499 | 14.23 |
| SPTB | Spectrin Beta, Erythrocytic | Protein Coding | GC14M064746 | 14.22 |
| NDUFA2 | NADH:Ubiquinone Oxidoreductase Subunit A2 | Protein Coding | GC05M140640 | 14.21 |
| GMPPB | GDP-Mannose Pyrophosphorylase B | Protein Coding | GC03M049716 | 14.21 |
| BSND | Barttin CLCNK Type Accessory Subunit Beta | Protein Coding | GC01P054998 | 14.2 |
| ICOSLG | Inducible T Cell Costimulator Ligand | Protein Coding | GC21M044222 | 14.16 |
| CFB | Complement Factor B | Protein Coding | GC06P031945 | 14.15 |
| CD27 | CD27 Molecule | Protein Coding | GC12P008144 | 14.14 |
| MTPN | Myotrophin | Protein Coding | GC07M135926 | 14.13 |
| SELPLG | Selectin P Ligand | Protein Coding | GC12M108621 | 14.11 |
| PDHA1 | Pyruvate Dehydrogenase E1 Subunit Alpha 1 | Protein Coding | GC0XP019343 | 14.1 |
| AHSP | Alpha Hemoglobin Stabilizing Protein | Protein Coding | GC16P031527 | 14.1 |
| APPL1 | Adaptor Protein, Phosphotyrosine Interacting With PH Domain And Leucine Zipper 1 | Protein Coding | GC03P057227 | 14.09 |
| PHKA2 | Phosphorylase Kinase Regulatory Subunit Alpha 2 | Protein Coding | GC0XM018892 | 14.08 |
| TERC | Telomerase RNA Component | RNA Gene | GC03M169765 | 14.07 |
| MNX1 | Motor Neuron And Pancreas Homeobox 1 | Protein Coding | GC07M156994 | 14.06 |
| RAB23 | RAB23, Member RAS Oncogene Family | Protein Coding | GC06M057187 | 14.05 |
| LBP | Lipopolysaccharide Binding Protein | Protein Coding | GC20P038346 | 14.05 |
| BOLA3 | BolA Family Member 3 | Protein Coding | GC02M074136 | 14.03 |
| FOXO1 | Forkhead Box O1 | Protein Coding | GC13M040555 | 14.03 |
| MEGF8 | Multiple EGF Like Domains 8 | Protein Coding | GC19P042325 | 14.02 |
| FADD | Fas Associated Via Death Domain | Protein Coding | GC11P070203 | 14.02 |
| COQ4 | Coenzyme Q4 | Protein Coding | GC09P128322 | 14.01 |
| TOP3A | DNA Topoisomerase III Alpha | Protein Coding | GC17M018271 | 13.99 |
| XRCC2 | X-Ray Repair Cross Complementing 2 | Protein Coding | GC07M152644 | 13.98 |
| MT-TW | Mitochondrially Encoded TRNA-Trp (UGA/G) | RNA Gene | GCMTP005514 | 13.97 |
| IRS2 | Insulin Receptor Substrate 2 | Protein Coding | GC13M109752 | 13.95 |
| GJA8 | Gap Junction Protein Alpha 8 | Protein Coding | GC01P147902 | 13.95 |
| XRCC4 | X-Ray Repair Cross Complementing 4 | Protein Coding | GC05P083077 | 13.93 |
| PLEKHM2 | Pleckstrin Homology And RUN Domain Containing M2 | Protein Coding | GC01P015691 | 13.92 |
| RASA1 | RAS P21 Protein Activator 1 | Protein Coding | GC05P087267 | 13.91 |
| NNT | Nicotinamide Nucleotide Transhydrogenase | Protein Coding | GC05P043603 | 13.91 |
| MCI2 | Myocardial Infarction, Susceptiblity To, 2 | Genetic Locus | GC13U900611 | 13.9 |
| MT-TF | Mitochondrially Encoded TRNA-Phe (UUU/C) | RNA Gene | GCMTP000580 | 13.9 |
| LTBP1 | Latent Transforming Growth Factor Beta Binding Protein 1 | Protein Coding | GC02P032915 | 13.9 |
| AIP | Aryl Hydrocarbon Receptor Interacting Protein | Protein Coding | GC11P067468 | 13.89 |
| TANGO2 | Transport And Golgi Organization 2 Homolog | Protein Coding | GC22P020017 | 13.87 |
| SLC11A1 | Solute Carrier Family 11 Member 1 | Protein Coding | GC02P218382 | 13.87 |
| CCDC65 | Coiled-Coil Domain Containing 65 | Protein Coding | GC12P048904 | 13.85 |
| CHRM3 | Cholinergic Receptor Muscarinic 3 | Protein Coding | GC01P239386 | 13.84 |
| CPOX | Coproporphyrinogen Oxidase | Protein Coding | GC03M098576 | 13.83 |
| SEC24C | SEC24 Homolog C, COPII Coat Complex Component | Protein Coding | GC10P073744 | 13.83 |
| TNFSF12 | TNF Superfamily Member 12 | Protein Coding | GC17P008027 | 13.83 |
| RREB1 | Ras Responsive Element Binding Protein 1 | Protein Coding | GC06P007107 | 13.8 |
| C1QBP | Complement C1q Binding Protein | Protein Coding | GC17M005432 | 13.79 |
| MSX2 | Msh Homeobox 2 | Protein Coding | GC05P174724 | 13.78 |
| ATPAF2 | ATP Synthase Mitochondrial F1 Complex Assembly Factor 2 | Protein Coding | GC17M017977 | 13.78 |
| NDUFA11 | NADH:Ubiquinone Oxidoreductase Subunit A11 | Protein Coding | GC19M005891 | 13.77 |
| ADAMTS7 | ADAM Metallopeptidase With Thrombospondin Type 1 Motif 7 | Protein Coding | GC15M078759 | 13.76 |
| PTHLH | Parathyroid Hormone Like Hormone | Protein Coding | GC12M027959 | 13.71 |
| GPR101 | G Protein-Coupled Receptor 101 | Protein Coding | GC0XM137030 | 13.7 |
| USP8 | Ubiquitin Specific Peptidase 8 | Protein Coding | GC15P050424 | 13.7 |
| SOAT1 | Sterol O-Acyltransferase 1 | Protein Coding | GC01P179262 | 13.7 |
| MAPK10 | Mitogen-Activated Protein Kinase 10 | Protein Coding | GC04M085990 | 13.64 |
| CPB2 | Carboxypeptidase B2 | Protein Coding | GC13M046053 | 13.63 |
| LIPG | Lipase G, Endothelial Type | Protein Coding | GC18P049560 | 13.62 |
| EPX | Eosinophil Peroxidase | Protein Coding | GC17P058192 | 13.6 |
| ABCG1 | ATP Binding Cassette Subfamily G Member 1 | Protein Coding | GC21P042199 | 13.6 |
| POGZ | Pogo Transposable Element Derived With ZNF Domain | Protein Coding | GC01M151375 | 13.59 |
| BRIP1 | BRCA1 Interacting Protein C-Terminal Helicase 1 | Protein Coding | GC17M061679 | 13.58 |
| MET | MET Proto-Oncogene, Receptor Tyrosine Kinase | Protein Coding | GC07P116672 | 13.57 |
| CYP1B1 | Cytochrome P450 Family 1 Subfamily B Member 1 | Protein Coding | GC02M038034 | 13.57 |
| MESP2 | Mesoderm Posterior BHLH Transcription Factor 2 | Protein Coding | GC15P089764 | 13.56 |
| CYP3A4 | Cytochrome P450 Family 3 Subfamily A Member 4 | Protein Coding | GC07M099759 | 13.56 |
| ADRA1A | Adrenoceptor Alpha 1A | Protein Coding | GC08M026747 | 13.54 |
| SH2D1A | SH2 Domain Containing 1A | Protein Coding | GC0XP124227 | 13.53 |
| BBS9 | Bardet-Biedl Syndrome 9 | Protein Coding | GC07P033112 | 13.52 |
| GATA2 | GATA Binding Protein 2 | Protein Coding | GC03M128479 | 13.51 |
| PPOX | Protoporphyrinogen Oxidase | Protein Coding | GC01P161167 | 13.51 |
| CAMK2D | Calcium/Calmodulin Dependent Protein Kinase II Delta | Protein Coding | GC04M113452 | 13.5 |
| MST1 | Macrophage Stimulating 1 | Protein Coding | GC03M049683 | 13.48 |
| SDHAF1 | Succinate Dehydrogenase Complex Assembly Factor 1 | Protein Coding | GC19P035995 | 13.48 |
| GET1 | Guided Entry Of Tail-Anchored Proteins Factor 1 | Protein Coding | GC21P039377 | 13.44 |
| FANCE | FA Complementation Group E | Protein Coding | GC06P047436 | 13.44 |
| DYRK1A | Dual Specificity Tyrosine Phosphorylation Regulated Kinase 1A | Protein Coding | GC21P037365 | 13.44 |
| ITPA | Inosine Triphosphatase | Protein Coding | GC20P003189 | 13.44 |
| PTPN1 | Protein Tyrosine Phosphatase Non-Receptor Type 1 | Protein Coding | GC20P050510 | 13.43 |
| PRKCE | Protein Kinase C Epsilon | Protein Coding | GC02P045651 | 13.43 |
| DHFR | Dihydrofolate Reductase | Protein Coding | GC05M080626 | 13.42 |
| RNASEH2C | Ribonuclease H2 Subunit C | Protein Coding | GC11M065714 | 13.42 |
| AHCY | Adenosylhomocysteinase | Protein Coding | GC20M034276 | 13.41 |
| HABP2 | Hyaluronan Binding Protein 2 | Protein Coding | GC10P113550 | 13.4 |
| SERPINH1 | Serpin Family H Member 1 | Protein Coding | GC11P075562 | 13.39 |
| HDAC4 | Histone Deacetylase 4 | Protein Coding | GC02M239048 | 13.38 |
| TPO | Thyroid Peroxidase | Protein Coding | GC02P001374 | 13.37 |
| NOX1 | NADPH Oxidase 1 | Protein Coding | GC0XM100843 | 13.36 |
| YY1 | YY1 Transcription Factor | Protein Coding | GC14P100238 | 13.36 |
| RTN4 | Reticulon 4 | Protein Coding | GC02M054934 | 13.36 |
| DLST | Dihydrolipoamide S-Succinyltransferase | Protein Coding | GC14P074881 | 13.36 |
| MORC2 | MORC Family CW-Type Zinc Finger 2 | Protein Coding | GC22M030925 | 13.34 |
| CYP3A5 | Cytochrome P450 Family 3 Subfamily A Member 5 | Protein Coding | GC07M099648 | 13.34 |
| SCNN1G | Sodium Channel Epithelial 1 Subunit Gamma | Protein Coding | GC16P023182 | 13.34 |
| ANK1 | Ankyrin 1 | Protein Coding | GC08M041653 | 13.29 |
| TGM2 | Transglutaminase 2 | Protein Coding | GC20M038127 | 13.28 |
| ERCC3 | ERCC Excision Repair 3, TFIIH Core Complex Helicase Subunit | Protein Coding | GC02M127257 | 13.25 |
| MIR15A | MicroRNA 15a | RNA Gene | GC13M050049 | 13.25 |
| ACVR1 | Activin A Receptor Type 1 | Protein Coding | GC02M157736 | 13.24 |
| TSFM | Ts Translation Elongation Factor, Mitochondrial | Protein Coding | GC12P057778 | 13.23 |
| DDX11 | DEAD/H-Box Helicase 11 | Protein Coding | GC12P031073 | 13.23 |
| KCNH1 | Potassium Voltage-Gated Channel Subfamily H Member 1 | Protein Coding | GC01M210678 | 13.23 |
| TAT | Tyrosine Aminotransferase | Protein Coding | GC16M071565 | 13.22 |
| ATP6V1A | ATPase H+ Transporting V1 Subunit A | Protein Coding | GC03P113747 | 13.21 |
| HACD1 | 3-Hydroxyacyl-CoA Dehydratase 1 | Protein Coding | GC10M017589 | 13.2 |
| RRAS | RAS Related | Protein Coding | GC19M049635 | 13.19 |
| EIF2AK3 | Eukaryotic Translation Initiation Factor 2 Alpha Kinase 3 | Protein Coding | GC02M088637 | 13.19 |
| COX20 | Cytochrome C Oxidase Assembly Factor COX20 | Protein Coding | GC01P244839 | 13.19 |
| FKBP1A | FKBP Prolyl Isomerase 1A | Protein Coding | GC20M001369 | 13.19 |
| CD2 | CD2 Molecule | Protein Coding | GC01P116754 | 13.18 |
| PDX1 | Pancreatic And Duodenal Homeobox 1 | Protein Coding | GC13P027921 | 13.16 |
| MIR20A | MicroRNA 20a | RNA Gene | GC13P091434 | 13.15 |
| MAP1B | Microtubule Associated Protein 1B | Protein Coding | GC05P072107 | 13.14 |
| MT-TV | Mitochondrially Encoded TRNA-Val (GUN) | RNA Gene | GCMTP001605 | 13.14 |
| SCO1 | Synthesis Of Cytochrome C Oxidase 1 | Protein Coding | GC17M010672 | 13.13 |
| PLAG1 | PLAG1 Zinc Finger | Protein Coding | GC08M056161 | 13.12 |
| SLC29A3 | Solute Carrier Family 29 Member 3 | Protein Coding | GC10P071320 | 13.12 |
| ETV6 | ETS Variant Transcription Factor 6 | Protein Coding | GC12P011649 | 13.11 |
| IL21 | Interleukin 21 | Protein Coding | GC04M122612 | 13.1 |
| ADAMTS4 | ADAM Metallopeptidase With Thrombospondin Type 1 Motif 4 | Protein Coding | GC01M161184 | 13.1 |
| HAVCR2 | Hepatitis A Virus Cellular Receptor 2 | Protein Coding | GC05M157063 | 13.09 |
| MGME1 | Mitochondrial Genome Maintenance Exonuclease 1 | Protein Coding | GC20P017952 | 13.09 |
| USP9X | Ubiquitin Specific Peptidase 9 X-Linked | Protein Coding | GC0XP041085 | 13.08 |
| COL17A1 | Collagen Type XVII Alpha 1 Chain | Protein Coding | GC10M104031 | 13.08 |
| VPS33A | VPS33A Core Subunit Of CORVET And HOPS Complexes | Protein Coding | GC12M122229 | 13.07 |
| MT-TE | Mitochondrially Encoded TRNA-Glu (GAA/G) | RNA Gene | GCMTM014676 | 13.07 |
| MT-TH | Mitochondrially Encoded TRNA-His (CAU/C) | RNA Gene | GCMTP012140 | 13.07 |
| THRB | Thyroid Hormone Receptor Beta | Protein Coding | GC03M024117 | 13.06 |
| MICU1 | Mitochondrial Calcium Uptake 1 | Protein Coding | GC10M072367 | 13.05 |
| IGFBP7 | Insulin Like Growth Factor Binding Protein 7 | Protein Coding | GC04M057030 | 13.04 |
| GAL | Galanin And GMAP Prepropeptide | Protein Coding | GC11P068684 | 13.03 |
| JMJD1C | Jumonji Domain Containing 1C | Protein Coding | GC10M063167 | 13.03 |
| HRC | Histidine Rich Calcium Binding Protein | Protein Coding | GC19M049151 | 13.03 |
| ARHGAP31 | Rho GTPase Activating Protein 31 | Protein Coding | GC03P119294 | 13.03 |
| CTSL | Cathepsin L | Protein Coding | GC09P087725 | 13.01 |
| TCIRG1 | T Cell Immune Regulator 1, ATPase H+ Transporting V0 Subunit A3 | Protein Coding | GC11P068038 | 13.01 |
| MIR31 | MicroRNA 31 | RNA Gene | GC09M021513 | 13.01 |
| MRPL44 | Mitochondrial Ribosomal Protein L44 | Protein Coding | GC02P223957 | 13.01 |
| SRSF2 | Serine And Arginine Rich Splicing Factor 2 | Protein Coding | GC17M076734 | 13.01 |
| PDYN | Prodynorphin | Protein Coding | GC20M001978 | 12.99 |
| ALDH7A1 | Aldehyde Dehydrogenase 7 Family Member A1 | Protein Coding | GC05M126541 | 12.99 |
| NDUFAF4 | NADH:Ubiquinone Oxidoreductase Complex Assembly Factor 4 | Protein Coding | GC06M096889 | 12.98 |
| SF3B4 | Splicing Factor 3b Subunit 4 | Protein Coding | GC01M149923 | 12.98 |
| SUN2 | Sad1 And UNC84 Domain Containing 2 | Protein Coding | GC22M045390 | 12.96 |
| SERPING1 | Serpin Family G Member 1 | Protein Coding | GC11P057597 | 12.94 |
| IKZF1 | IKAROS Family Zinc Finger 1 | Protein Coding | GC07P050303 | 12.94 |
| IRAK1 | Interleukin 1 Receptor Associated Kinase 1 | Protein Coding | GC0XM154010 | 12.94 |
| SLC30A10 | Solute Carrier Family 30 Member 10 | Protein Coding | GC01M219685 | 12.94 |
| MRPS22 | Mitochondrial Ribosomal Protein S22 | Protein Coding | GC03P139005 | 12.93 |
| FANCM | FA Complementation Group M | Protein Coding | GC14P045135 | 12.92 |
| ATN1 | Atrophin 1 | Protein Coding | GC12P008222 | 12.92 |
| CHUK | Component Of Inhibitor Of Nuclear Factor Kappa B Kinase Complex | Protein Coding | GC10M100188 | 12.9 |
| STAT6 | Signal Transducer And Activator Of Transcription 6 | Protein Coding | GC12M057095 | 12.89 |
| IL15 | Interleukin 15 | Protein Coding | GC04P141636 | 12.87 |
| AGRP | Agouti Related Neuropeptide | Protein Coding | GC16M067482 | 12.86 |
| XRCC1 | X-Ray Repair Cross Complementing 1 | Protein Coding | GC19M043543 | 12.85 |
| RHO | Rhodopsin | Protein Coding | GC03P130619 | 12.84 |
| CXCR3 | C-X-C Motif Chemokine Receptor 3 | Protein Coding | GC0XM071615 | 12.83 |
| TUBB | Tubulin Beta Class I | Protein Coding | GC06P030720 | 12.83 |
| TFAP2A | Transcription Factor AP-2 Alpha | Protein Coding | GC06M010393 | 12.81 |
| TBX21 | T-Box Transcription Factor 21 | Protein Coding | GC17P047733 | 12.81 |
| FTH1 | Ferritin Heavy Chain 1 | Protein Coding | GC11M061959 | 12.8 |
| CX3CL1 | C-X3-C Motif Chemokine Ligand 1 | Protein Coding | GC16P057372 | 12.8 |
| SLC34A1 | Solute Carrier Family 34 Member 1 | Protein Coding | GC05P177380 | 12.78 |
| PIGV | Phosphatidylinositol Glycan Anchor Biosynthesis Class V | Protein Coding | GC01P026787 | 12.78 |
| NSDHL | NAD(P) Dependent Steroid Dehydrogenase-Like | Protein Coding | GC0XP152830 | 12.78 |
| PLCG1 | Phospholipase C Gamma 1 | Protein Coding | GC20P041136 | 12.78 |
| CYP1A1 | Cytochrome P450 Family 1 Subfamily A Member 1 | Protein Coding | GC15M074719 | 12.78 |
| MIR191 | MicroRNA 191 | RNA Gene | GC03M049247 | 12.77 |
| CLCN1 | Chloride Voltage-Gated Channel 1 | Protein Coding | GC07P143316 | 12.76 |
| UCP1 | Uncoupling Protein 1 | Protein Coding | GC04M140559 | 12.75 |
| COMP | Cartilage Oligomeric Matrix Protein | Protein Coding | GC19M018783 | 12.7 |
| LPXN | Leupaxin | Protein Coding | GC11M061238 | 12.7 |
| DYRK1B | Dual Specificity Tyrosine Phosphorylation Regulated Kinase 1B | Protein Coding | GC19M039825 | 12.69 |
| S100A9 | S100 Calcium Binding Protein A9 | Protein Coding | GC01P153357 | 12.68 |
| ELAC2 | ElaC Ribonuclease Z 2 | Protein Coding | GC17M012991 | 12.67 |
| MT-TQ | Mitochondrially Encoded TRNA-Gln (CAA/G) | RNA Gene | GCMTM004331 | 12.66 |
| DLL3 | Delta Like Canonical Notch Ligand 3 | Protein Coding | GC19P039498 | 12.65 |
| UBE2T | Ubiquitin Conjugating Enzyme E2 T | Protein Coding | GC01M202300 | 12.65 |
| DHCR24 | 24-Dehydrocholesterol Reductase | Protein Coding | GC01M054849 | 12.64 |
| FBXL4 | F-Box And Leucine Rich Repeat Protein 4 | Protein Coding | GC06M098868 | 12.64 |
| CACNA1H | Calcium Voltage-Gated Channel Subunit Alpha1 H | Protein Coding | GC16P001153 | 12.64 |
| C4B | Complement C4B (Chido Blood Group) | Protein Coding | GC06P032014 | 12.64 |
| KCNA1 | Potassium Voltage-Gated Channel Subfamily A Member 1 | Protein Coding | GC12P008111 | 12.61 |
| ANK3 | Ankyrin 3 | Protein Coding | GC10M060026 | 12.59 |
| GZMB | Granzyme B | Protein Coding | GC14M024630 | 12.59 |
| SLC26A1 | Solute Carrier Family 26 Member 1 | Protein Coding | GC04M000979 | 12.58 |
| CD44 | CD44 Molecule (Indian Blood Group) | Protein Coding | GC11P035139 | 12.57 |
| SLX4 | SLX4 Structure-Specific Endonuclease Subunit | Protein Coding | GC16M003581 | 12.57 |
| VIP | Vasoactive Intestinal Peptide | Protein Coding | GC06P152750 | 12.56 |
| RARRES2 | Retinoic Acid Receptor Responder 2 | Protein Coding | GC07M150333 | 12.56 |
| TRIB1 | Tribbles Pseudokinase 1 | Protein Coding | GC08P125430 | 12.56 |
| COX8A | Cytochrome C Oxidase Subunit 8A | Protein Coding | GC11P063977 | 12.55 |
| PAX8 | Paired Box 8 | Protein Coding | GC02M113215 | 12.55 |
| ATG5 | Autophagy Related 5 | Protein Coding | GC06M106045 | 12.55 |
| CEP164 | Centrosomal Protein 164 | Protein Coding | GC11P117314 | 12.54 |
| COLQ | Collagen Like Tail Subunit Of Asymmetric Acetylcholinesterase | Protein Coding | GC03M015815 | 12.54 |
| SERPINF1 | Serpin Family F Member 1 | Protein Coding | GC17P001761 | 12.54 |
| LEMD2 | LEM Domain Nuclear Envelope Protein 2 | Protein Coding | GC06M033772 | 12.54 |
| APOC2 | Apolipoprotein C2 | Protein Coding | GC19P044945 | 12.53 |
| PRPF8 | Pre-MRNA Processing Factor 8 | Protein Coding | GC17M001650 | 12.52 |
| MIR205 | MicroRNA 205 | RNA Gene | GC01P209432 | 12.51 |
| NSMCE2 | NSE2 (MMS21) Homolog, SMC5-SMC6 Complex SUMO Ligase | Protein Coding | GC08P125091 | 12.49 |
| PDSS1 | Decaprenyl Diphosphate Synthase Subunit 1 | Protein Coding | GC10P026697 | 12.48 |
| ATP1A2 | ATPase Na+/K+ Transporting Subunit Alpha 2 | Protein Coding | GC01P160115 | 12.46 |
| ANKS6 | Ankyrin Repeat And Sterile Alpha Motif Domain Containing 6 | Protein Coding | GC09M098731 | 12.46 |
| WARS1 | Tryptophanyl-TRNA Synthetase 1 | Protein Coding | GC14M100334 | 12.44 |
| COA5 | Cytochrome C Oxidase Assembly Factor 5 | Protein Coding | GC02M098599 | 12.44 |
| MIR185 | MicroRNA 185 | RNA Gene | GC22P020034 | 12.44 |
| CGA | Glycoprotein Hormones, Alpha Polypeptide | Protein Coding | GC06M087085 | 12.43 |
| GAST | Gastrin | Protein Coding | GC17P041712 | 12.43 |
| MAOB | Monoamine Oxidase B | Protein Coding | GC0XM043766 | 12.41 |
| SUMO1 | Small Ubiquitin Like Modifier 1 | Protein Coding | GC02M202206 | 12.41 |
| SEMA3C | Semaphorin 3C | Protein Coding | GC07M080742 | 12.41 |
| COX14 | Cytochrome C Oxidase Assembly Factor COX14 | Protein Coding | GC12P050111 | 12.4 |
| TNFRSF13C | TNF Receptor Superfamily Member 13C | Protein Coding | GC22M045411 | 12.39 |
| RAB3GAP2 | RAB3 GTPase Activating Non-Catalytic Protein Subunit 2 | Protein Coding | GC01M220149 | 12.38 |
| RPS27A | Ribosomal Protein S27a | Protein Coding | GC02P055231 | 12.37 |
| CALCRL | Calcitonin Receptor Like Receptor | Protein Coding | GC02M187341 | 12.36 |
| DDIT3 | DNA Damage Inducible Transcript 3 | Protein Coding | GC12M057516 | 12.36 |
| PPARD | Peroxisome Proliferator Activated Receptor Delta | Protein Coding | GC06P047433 | 12.32 |
| CDK5 | Cyclin Dependent Kinase 5 | Protein Coding | GC07M151053 | 12.32 |
| COA8 | Cytochrome C Oxidase Assembly Factor 8 | Protein Coding | GC14P104661 | 12.32 |
| TMPRSS6 | Transmembrane Serine Protease 6 | Protein Coding | GC22M037066 | 12.31 |
| TAC1 | Tachykinin Precursor 1 | Protein Coding | GC07P097731 | 12.3 |
| CPS1 | Carbamoyl-Phosphate Synthase 1 | Protein Coding | GC02P210477 | 12.28 |
| MIR15B | MicroRNA 15b | RNA Gene | GC03P160404 | 12.27 |
| TACO1 | Translational Activator Of Cytochrome C Oxidase I | Protein Coding | GC17P063600 | 12.27 |
| CPLANE1 | Ciliogenesis And Planar Polarity Effector 1 | Protein Coding | GC05M037065 | 12.25 |
| TUG1 | Taurine Up-Regulated 1 | RNA Gene | GC22P030969 | 12.25 |
| MMRN1 | Multimerin 1 | Protein Coding | GC04P089879 | 12.25 |
| NBN | Nibrin | Protein Coding | GC08M089933 | 12.25 |
| NFKB2 | Nuclear Factor Kappa B Subunit 2 | Protein Coding | GC10P102394 | 12.25 |
| HPGD | 15-Hydroxyprostaglandin Dehydrogenase | Protein Coding | GC04M174490 | 12.24 |
| GSTP1 | Glutathione S-Transferase Pi 1 | Protein Coding | GC11P067583 | 12.24 |
| TIMMDC1 | Translocase Of Inner Mitochondrial Membrane Domain Containing 1 | Protein Coding | GC03P119498 | 12.23 |
| TOMM40 | Translocase Of Outer Mitochondrial Membrane 40 | Protein Coding | GC19P044890 | 12.23 |
| ADRA1B | Adrenoceptor Alpha 1B | Protein Coding | GC05P159867 | 12.21 |
| STAT5A | Signal Transducer And Activator Of Transcription 5A | Protein Coding | GC17P042287 | 12.21 |
| MIR204 | MicroRNA 204 | RNA Gene | GC09M070809 | 12.19 |
| RCAN1 | Regulator Of Calcineurin 1 | Protein Coding | GC21M034513 | 12.18 |
| CERS1 | Ceramide Synthase 1 | Protein Coding | GC19M018868 | 12.17 |
| SMO | Smoothened, Frizzled Class Receptor | Protein Coding | GC07P129463 | 12.16 |
| FRAS1 | Fraser Extracellular Matrix Complex Subunit 1 | Protein Coding | GC04P078056 | 12.16 |
| EXT2 | Exostosin Glycosyltransferase 2 | Protein Coding | GC11P044095 | 12.16 |
| LOXL1 | Lysyl Oxidase Like 1 | Protein Coding | GC15P073925 | 12.14 |
| BCR | BCR Activator Of RhoGEF And GTPase | Protein Coding | GC22P023179 | 12.12 |
| OGDH | Oxoglutarate Dehydrogenase | Protein Coding | GC07P044606 | 12.12 |
| KBTBD13 | Kelch Repeat And BTB Domain Containing 13 | Protein Coding | GC15P072875 | 12.11 |
| LPP | LIM Domain Containing Preferred Translocation Partner In Lipoma | Protein Coding | GC03P188153 | 12.1 |
| VLDLR | Very Low Density Lipoprotein Receptor | Protein Coding | GC09P002611 | 12.1 |
| ITGA8 | Integrin Subunit Alpha 8 | Protein Coding | GC10M015513 | 12.09 |
| TRPC3 | Transient Receptor Potential Cation Channel Subfamily C Member 3 | Protein Coding | GC04M121879 | 12.09 |
| ANG | Angiogenin | Protein Coding | GC14P020830 | 12.09 |
| WNT5A | Wnt Family Member 5A | Protein Coding | GC03M055465 | 12.09 |
| PSMD4 | Proteasome 26S Subunit, Non-ATPase 4 | Protein Coding | GC01P151227 | 12.09 |
| MYOC | Myocilin | Protein Coding | GC01M171604 | 12.08 |
| MIR93 | MicroRNA 93 | RNA Gene | GC07M100282 | 12.07 |
| DPH1 | Diphthamide Biosynthesis 1 | Protein Coding | GC17P002030 | 12.06 |
| PDE9A | Phosphodiesterase 9A | Protein Coding | GC21P042653 | 12.06 |
| NBAS | NBAS Subunit Of NRZ Tethering Complex | Protein Coding | GC02M014998 | 12.06 |
| TFEB | Transcription Factor EB | Protein Coding | GC06M042295 | 12.05 |
| ACKR1 | Atypical Chemokine Receptor 1 (Duffy Blood Group) | Protein Coding | GC01P159203 | 12.05 |
| TGFA | Transforming Growth Factor Alpha | Protein Coding | GC02M070447 | 12.05 |
| MAP3K20 | Mitogen-Activated Protein Kinase Kinase Kinase 20 | Protein Coding | GC02P173076 | 12.02 |
| ITPR3 | Inositol 1,4,5-Trisphosphate Receptor Type 3 | Protein Coding | GC06P033620 | 12.02 |
| SLC2A9 | Solute Carrier Family 2 Member 9 | Protein Coding | GC04M009772 | 11.99 |
| COA6 | Cytochrome C Oxidase Assembly Factor 6 | Protein Coding | GC01P234374 | 11.99 |
| ADCY6 | Adenylate Cyclase 6 | Protein Coding | GC12M048766 | 11.97 |
| RPL26 | Ribosomal Protein L26 | Protein Coding | GC17M008377 | 11.97 |
| DICER1 | Dicer 1, Ribonuclease III | Protein Coding | GC14M095086 | 11.94 |
| PRKAA2 | Protein Kinase AMP-Activated Catalytic Subunit Alpha 2 | Protein Coding | GC01P056645 | 11.93 |
| UBE3B | Ubiquitin Protein Ligase E3B | Protein Coding | GC12P109477 | 11.92 |
| CA3 | Carbonic Anhydrase 3 | Protein Coding | GC08P085373 | 11.91 |
| CDON | Cell Adhesion Associated, Oncogene Regulated | Protein Coding | GC11M125955 | 11.91 |
| CTC1 | CST Telomere Replication Complex Component 1 | Protein Coding | GC17M008875 | 11.9 |
| CDH13 | Cadherin 13 | Protein Coding | GC16P082626 | 11.9 |
| MESP1 | Mesoderm Posterior BHLH Transcription Factor 1 | Protein Coding | GC15M089748 | 11.9 |
| LIPT1 | Lipoyltransferase 1 | Protein Coding | GC02P099160 | 11.9 |
| CSNK2A1 | Casein Kinase 2 Alpha 1 | Protein Coding | GC20M000472 | 11.89 |
| SERPINB2 | Serpin Family B Member 2 | Protein Coding | GC18P063871 | 11.89 |
| LAMB3 | Laminin Subunit Beta 3 | Protein Coding | GC01M209614 | 11.89 |
| PRKCD | Protein Kinase C Delta | Protein Coding | GC03P053156 | 11.89 |
| C2 | Complement C2 | Protein Coding | GC06P031897 | 11.89 |
| CCL4 | C-C Motif Chemokine Ligand 4 | Protein Coding | GC17P036103 | 11.87 |
| MIR146B | MicroRNA 146b | RNA Gene | GC10P102436 | 11.85 |
| SLC5A2 | Solute Carrier Family 5 Member 2 | Protein Coding | GC16P031580 | 11.85 |
| HMGA2 | High Mobility Group AT-Hook 2 | Protein Coding | GC12P065824 | 11.84 |
| CSF1R | Colony Stimulating Factor 1 Receptor | Protein Coding | GC05M150053 | 11.82 |
| SDCCAG8 | SHH Signaling And Ciliogenesis Regulator SDCCAG8 | Protein Coding | GC01P243255 | 11.82 |
| MTO1 | Mitochondrial TRNA Translation Optimization 1 | Protein Coding | GC06P073461 | 11.81 |
| S100A8 | S100 Calcium Binding Protein A8 | Protein Coding | GC01M153391 | 11.8 |
| ROCK1 | Rho Associated Coiled-Coil Containing Protein Kinase 1 | Protein Coding | GC18M020946 | 11.79 |
| SPRED1 | Sprouty Related EVH1 Domain Containing 1 | Protein Coding | GC15P038252 | 11.78 |
| PPP3CA | Protein Phosphatase 3 Catalytic Subunit Alpha | Protein Coding | GC04M101024 | 11.76 |
| SRD5A2 | Steroid 5 Alpha-Reductase 2 | Protein Coding | GC02M031522 | 11.76 |
| UBR1 | Ubiquitin Protein Ligase E3 Component N-Recognin 1 | Protein Coding | GC15M042942 | 11.75 |
| COG7 | Component Of Oligomeric Golgi Complex 7 | Protein Coding | GC16M023307 | 11.75 |
| PIGT | Phosphatidylinositol Glycan Anchor Biosynthesis Class T | Protein Coding | GC20P045416 | 11.74 |
| MIR29C | MicroRNA 29c | RNA Gene | GC01M207802 | 11.73 |
| FRZB | Frizzled Related Protein | Protein Coding | GC02M182833 | 11.72 |
| GIGYF2 | GRB10 Interacting GYF Protein 2 | Protein Coding | GC02P232698 | 11.71 |
| NRP1 | Neuropilin 1 | Protein Coding | GC10M033177 | 11.71 |
| EEF2 | Eukaryotic Translation Elongation Factor 2 | Protein Coding | GC19M003976 | 11.71 |
| HS3ST1 | Heparan Sulfate-Glucosamine 3-Sulfotransferase 1 | Protein Coding | GC04M011394 | 11.69 |
| COL4A4 | Collagen Type IV Alpha 4 Chain | Protein Coding | GC02M226967 | 11.69 |
| PDGFA | Platelet Derived Growth Factor Subunit A | Protein Coding | GC07M000497 | 11.66 |
| GSK3B | Glycogen Synthase Kinase 3 Beta | Protein Coding | GC03M119821 | 11.66 |
| POMK | Protein O-Mannose Kinase | Protein Coding | GC08P043093 | 11.66 |
| PCSK1 | Proprotein Convertase Subtilisin/Kexin Type 1 | Protein Coding | GC05M096391 | 11.65 |
| ENTPD1 | Ectonucleoside Triphosphate Diphosphohydrolase 1 | Protein Coding | GC10P095711 | 11.64 |
| TLR7 | Toll Like Receptor 7 | Protein Coding | GC0XP012867 | 11.64 |
| C1R | Complement C1r | Protein Coding | GC12M007135 | 11.63 |
| ATAD3A | ATPase Family AAA Domain Containing 3A | Protein Coding | GC01P001570 | 11.63 |
| UBE2L3 | Ubiquitin Conjugating Enzyme E2 L3 | Protein Coding | GC22P021549 | 11.62 |
| ADH1C | Alcohol Dehydrogenase 1C (Class I), Gamma Polypeptide | Protein Coding | GC04M099336 | 11.62 |
| RAP1A | RAP1A, Member Of RAS Oncogene Family | Protein Coding | GC01P111542 | 11.61 |
| MYOZ1 | Myozenin 1 | Protein Coding | GC10M073631 | 11.61 |
| ZFHX3 | Zinc Finger Homeobox 3 | Protein Coding | GC16M072782 | 11.6 |
| TRPS1 | Transcriptional Repressor GATA Binding 1 | Protein Coding | GC08M115408 | 11.6 |
| ABCC1 | ATP Binding Cassette Subfamily C Member 1 | Protein Coding | GC16P015949 | 11.6 |
| TULP1 | TUB Like Protein 1 | Protein Coding | GC06M042216 | 11.56 |
| TRAF6 | TNF Receptor Associated Factor 6 | Protein Coding | GC11M036467 | 11.56 |
| TLR6 | Toll Like Receptor 6 | Protein Coding | GC04M038828 | 11.56 |
| SLC22A12 | Solute Carrier Family 22 Member 12 | Protein Coding | GC11P064609 | 11.56 |
| MAP3K7 | Mitogen-Activated Protein Kinase Kinase Kinase 7 | Protein Coding | GC06M090513 | 11.55 |
| WRAP53 | WD Repeat Containing Antisense To TP53 | Protein Coding | GC17P008049 | 11.55 |
| VPS33B | VPS33B Late Endosome And Lysosome Associated | Protein Coding | GC15M090998 | 11.55 |
| ACADL | Acyl-CoA Dehydrogenase Long Chain | Protein Coding | GC02M210187 | 11.54 |
| CLCN7 | Chloride Voltage-Gated Channel 7 | Protein Coding | GC16M001444 | 11.53 |
| CLEC7A | C-Type Lectin Domain Containing 7A | Protein Coding | GC12M013863 | 11.52 |
| KLHL41 | Kelch Like Family Member 41 | Protein Coding | GC02P169509 | 11.52 |
| ADAMTSL1 | ADAMTS Like 1 | Protein Coding | GC09P017906 | 11.5 |
| SOX18 | SRY-Box Transcription Factor 18 | Protein Coding | GC20M064047 | 11.49 |
| STIM1 | Stromal Interaction Molecule 1 | Protein Coding | GC11P003855 | 11.49 |
| ATHS | Atherosclerosis Susceptibility (Lipoprotein Associated) | Genetic Locus | GC19U990005 | 11.48 |
| PCYT1A | Phosphate Cytidylyltransferase 1, Choline, Alpha | Protein Coding | GC03M196214 | 11.46 |
| TLR1 | Toll Like Receptor 1 | Protein Coding | GC04M038797 | 11.45 |
| TRPM7 | Transient Receptor Potential Cation Channel Subfamily M Member 7 | Protein Coding | GC15M050552 | 11.44 |
| CD80 | CD80 Molecule | Protein Coding | GC03M119524 | 11.43 |
| ABCG2 | ATP Binding Cassette Subfamily G Member 2 (Junior Blood Group) | Protein Coding | GC04M088090 | 11.42 |
| MIR18A | MicroRNA 18a | RNA Gene | GC13P091432 | 11.41 |
| PYGB | Glycogen Phosphorylase B | Protein Coding | GC20P025248 | 11.41 |
| SLC7A7 | Solute Carrier Family 7 Member 7 | Protein Coding | GC14M022773 | 11.41 |
| KCNN4 | Potassium Calcium-Activated Channel Subfamily N Member 4 | Protein Coding | GC19M043767 | 11.41 |
| MT-TN | Mitochondrially Encoded TRNA-Asn (AAU/C) | RNA Gene | GCMTM005659 | 11.41 |
| CRLF1 | Cytokine Receptor Like Factor 1 | Protein Coding | GC19M018572 | 11.4 |
| IRF8 | Interferon Regulatory Factor 8 | Protein Coding | GC16P085898 | 11.4 |
| DTNBP1 | Dystrobrevin Binding Protein 1 | Protein Coding | GC06M015470 | 11.39 |
| FABP2 | Fatty Acid Binding Protein 2 | Protein Coding | GC04M119317 | 11.38 |
| GC | GC Vitamin D Binding Protein | Protein Coding | GC04M071741 | 11.38 |
| MYH10 | Myosin Heavy Chain 10 | Protein Coding | GC17M008474 | 11.38 |
| INHBA | Inhibin Subunit Beta A | Protein Coding | GC07M041668 | 11.38 |
| RFWD3 | Ring Finger And WD Repeat Domain 3 | Protein Coding | GC16M074621 | 11.37 |
| LTA4H | Leukotriene A4 Hydrolase | Protein Coding | GC12M096000 | 11.37 |
| MIR342 | MicroRNA 342 | RNA Gene | GC14P100109 | 11.37 |
| CFLAR | CASP8 And FADD Like Apoptosis Regulator | Protein Coding | GC02P201117 | 11.36 |
| SYNE4 | Spectrin Repeat Containing Nuclear Envelope Family Member 4 | Protein Coding | GC19M036003 | 11.36 |
| DHCR7 | 7-Dehydrocholesterol Reductase | Protein Coding | GC11M071428 | 11.33 |
| MOCS1 | Molybdenum Cofactor Synthesis 1 | Protein Coding | GC06M039899 | 11.33 |
| LTF | Lactotransferrin | Protein Coding | GC03M046435 | 11.32 |
| PRKCA | Protein Kinase C Alpha | Protein Coding | GC17P066302 | 11.31 |
| FCGR3A | Fc Fragment Of IgG Receptor IIIa | Protein Coding | GC01M161541 | 11.31 |
| POU5F1 | POU Class 5 Homeobox 1 | Protein Coding | GC06M031184 | 11.31 |
| CAPN1 | Calpain 1 | Protein Coding | GC11P065198 | 11.31 |
| CISD2 | CDGSH Iron Sulfur Domain 2 | Protein Coding | GC04P102868 | 11.3 |
| NDUFA4 | NDUFA4 Mitochondrial Complex Associated | Protein Coding | GC07M010938 | 11.28 |
| SMARCAL1 | SWI/SNF Related, Matrix Associated, Actin Dependent Regulator Of Chromatin, Subfamily A Like 1 | Protein Coding | GC02P216412 | 11.28 |
| GRIK2 | Glutamate Ionotropic Receptor Kainate Type Subunit 2 | Protein Coding | GC06P101181 | 11.27 |
| IRAK4 | Interleukin 1 Receptor Associated Kinase 4 | Protein Coding | GC12P043758 | 11.27 |
| GDF2 | Growth Differentiation Factor 2 | Protein Coding | GC10P047322 | 11.27 |
| SIRT3 | Sirtuin 3 | Protein Coding | GC11M000215 | 11.26 |
| MAP3K5 | Mitogen-Activated Protein Kinase Kinase Kinase 5 | Protein Coding | GC06M136557 | 11.26 |
| PARP1 | Poly(ADP-Ribose) Polymerase 1 | Protein Coding | GC01M226360 | 11.26 |
| UMPS | Uridine Monophosphate Synthetase | Protein Coding | GC03P124730 | 11.23 |
| PPP1R3A | Protein Phosphatase 1 Regulatory Subunit 3A | Protein Coding | GC07M113876 | 11.22 |
| TMEM106B | Transmembrane Protein 106B | Protein Coding | GC07P012217 | 11.22 |
| CUBN | Cubilin | Protein Coding | GC10M016824 | 11.22 |
| KLKB1 | Kallikrein B1 | Protein Coding | GC04P186208 | 11.22 |
| DPM3 | Dolichyl-Phosphate Mannosyltransferase Subunit 3, Regulatory | Protein Coding | GC01M155112 | 11.21 |
| RNASE3 | Ribonuclease A Family Member 3 | Protein Coding | GC14P020891 | 11.21 |
| ITGAV | Integrin Subunit Alpha V | Protein Coding | GC02P186589 | 11.21 |
| WNT1 | Wnt Family Member 1 | Protein Coding | GC12P049053 | 11.2 |
| GRP | Gastrin Releasing Peptide | Protein Coding | GC18P059220 | 11.19 |
| NUP188 | Nucleoporin 188 | Protein Coding | GC09P128947 | 11.19 |
| BVES | Blood Vessel Epicardial Substance | Protein Coding | GC06M105096 | 11.18 |
| NR0B2 | Nuclear Receptor Subfamily 0 Group B Member 2 | Protein Coding | GC01M026922 | 11.18 |
| AMH | Anti-Mullerian Hormone | Protein Coding | GC19P002251 | 11.18 |
| NOG | Noggin | Protein Coding | GC17P056593 | 11.17 |
| ATP5F1D | ATP Synthase F1 Subunit Delta | Protein Coding | GC19P001242 | 11.16 |
| PNPLA3 | Patatin Like Phospholipase Domain Containing 3 | Protein Coding | GC22P043923 | 11.16 |
| RPS26 | Ribosomal Protein S26 | Protein Coding | GC12P056043 | 11.15 |
| TRPM3 | Transient Receptor Potential Cation Channel Subfamily M Member 3 | Protein Coding | GC09M070529 | 11.14 |
| RHOD | Ras Homolog Family Member D | Protein Coding | GC11P067057 | 11.14 |
| FLAD1 | Flavin Adenine Dinucleotide Synthetase 1 | Protein Coding | GC01P154983 | 11.1 |
| EGR1 | Early Growth Response 1 | Protein Coding | GC05P138465 | 11.1 |
| ALX4 | ALX Homeobox 4 | Protein Coding | GC11M044238 | 11.1 |
| ATP6V0A2 | ATPase H+ Transporting V0 Subunit A2 | Protein Coding | GC12P123712 | 11.08 |
| LIG4 | DNA Ligase 4 | Protein Coding | GC13M108207 | 11.08 |
| MAD2L2 | Mitotic Arrest Deficient 2 Like 2 | Protein Coding | GC01M011674 | 11.08 |
| CXCR1 | C-X-C Motif Chemokine Receptor 1 | Protein Coding | GC02M218162 | 11.08 |
| HOTTIP | HOXA Distal Transcript Antisense RNA | RNA Gene | GC07P027198 | 11.07 |
| TGFBR3 | Transforming Growth Factor Beta Receptor 3 | Protein Coding | GC01M091680 | 11.06 |
| COL6A1 | Collagen Type VI Alpha 1 Chain | Protein Coding | GC21P045981 | 11.06 |
| FGF4 | Fibroblast Growth Factor 4 | Protein Coding | GC11M069762 | 11.04 |
| C8A | Complement C8 Alpha Chain | Protein Coding | GC01P056854 | 11.04 |
| CYP2E1 | Cytochrome P450 Family 2 Subfamily E Member 1 | Protein Coding | GC10P133520 | 11.03 |
| CXADR | CXADR Ig-Like Cell Adhesion Molecule | Protein Coding | GC21P017512 | 11.03 |
| NTF3 | Neurotrophin 3 | Protein Coding | GC12P005432 | 11.03 |
| PET100 | PET100 Cytochrome C Oxidase Chaperone | Protein Coding | GC19P007630 | 11.02 |
| PLCE1 | Phospholipase C Epsilon 1 | Protein Coding | GC10P093993 | 11.01 |
| HPS1 | HPS1 Biogenesis Of Lysosomal Organelles Complex 3 Subunit 1 | Protein Coding | GC10M098416 | 11.01 |
| HSPA9 | Heat Shock Protein Family A (Hsp70) Member 9 | Protein Coding | GC05M138554 | 11.01 |
| NDP | Norrin Cystine Knot Growth Factor NDP | Protein Coding | GC0XM043948 | 11.01 |
| SEMA3D | Semaphorin 3D | Protein Coding | GC07M084995 | 11.01 |
| CTSH | Cathepsin H | Protein Coding | GC15M078925 | 11 |
| MICA | MHC Class I Polypeptide-Related Sequence A | Protein Coding | GC06P031399 | 11 |
| ITGA6 | Integrin Subunit Alpha 6 | Protein Coding | GC02P172427 | 11 |
| ARID2 | AT-Rich Interaction Domain 2 | Protein Coding | GC12P045729 | 11 |
| SRY | Sex Determining Region Y | Protein Coding | GC0YM002698 | 11 |
| AHR | Aryl Hydrocarbon Receptor | Protein Coding | GC07P016916 | 11 |
| NR4A2 | Nuclear Receptor Subfamily 4 Group A Member 2 | Protein Coding | GC02M156324 | 10.98 |
| CACNA1B | Calcium Voltage-Gated Channel Subunit Alpha1 B | Protein Coding | GC09P137877 | 10.97 |
| OBSCN | Obscurin, Cytoskeletal Calmodulin And Titin-Interacting RhoGEF | Protein Coding | GC01P228208 | 10.96 |
| CARD9 | Caspase Recruitment Domain Family Member 9 | Protein Coding | GC09M136361 | 10.96 |
| PLA2G4A | Phospholipase A2 Group IVA | Protein Coding | GC01P186798 | 10.95 |
| LFNG | LFNG O-Fucosylpeptide 3-Beta-N-Acetylglucosaminyltransferase | Protein Coding | GC07P002512 | 10.94 |
| PAFAH1B1 | Platelet Activating Factor Acetylhydrolase 1b Regulatory Subunit 1 | Protein Coding | GC17P002593 | 10.94 |
| RBBP8 | RB Binding Protein 8, Endonuclease | Protein Coding | GC18P022798 | 10.94 |
| PPP1R12A | Protein Phosphatase 1 Regulatory Subunit 12A | Protein Coding | GC12M079773 | 10.92 |
| THRA | Thyroid Hormone Receptor Alpha | Protein Coding | GC17P040058 | 10.91 |
| ADCY3 | Adenylate Cyclase 3 | Protein Coding | GC02M024819 | 10.91 |
| PGR | Progesterone Receptor | Protein Coding | GC11M100943 | 10.89 |
| PRSS1 | Serine Protease 1 | Protein Coding | GC07P144938 | 10.88 |
| EPB42 | Erythrocyte Membrane Protein Band 4.2 | Protein Coding | GC15M043214 | 10.87 |
| LOC110806262 | Solute Carrier Family 6 Member 4 Gene Promoter | Biological Region | GC17P030235 | 10.87 |
| ICOS | Inducible T Cell Costimulator | Protein Coding | GC02P203937 | 10.86 |
| LAMC2 | Laminin Subunit Gamma 2 | Protein Coding | GC01P183155 | 10.86 |
| PROKR2 | Prokineticin Receptor 2 | Protein Coding | GC20M005303 | 10.86 |
| TNC | Tenascin C | Protein Coding | GC09M115019 | 10.84 |
| HTR1A | 5-Hydroxytryptamine Receptor 1A | Protein Coding | GC05M063960 | 10.84 |
| ADAMTS3 | ADAM Metallopeptidase With Thrombospondin Type 1 Motif 3 | Protein Coding | GC04M072280 | 10.84 |
| IL4I1 | Interleukin 4 Induced 1 | Protein Coding | GC19M049890 | 10.81 |
| DNAJC13 | DnaJ Heat Shock Protein Family (Hsp40) Member C13 | Protein Coding | GC03P132417 | 10.8 |
| MIR99A | MicroRNA 99a | RNA Gene | GC21P016539 | 10.79 |
| CTH | Cystathionine Gamma-Lyase | Protein Coding | GC01P070411 | 10.79 |
| SLC2A2 | Solute Carrier Family 2 Member 2 | Protein Coding | GC03M170996 | 10.78 |
| FAN1 | FANCD2 And FANCI Associated Nuclease 1 | Protein Coding | GC15P031408 | 10.78 |
| IFNA2 | Interferon Alpha 2 | Protein Coding | GC09M021384 | 10.77 |
| SEMA3E | Semaphorin 3E | Protein Coding | GC07M083363 | 10.76 |
| NLRP12 | NLR Family Pyrin Domain Containing 12 | Protein Coding | GC19M053793 | 10.76 |
| KIAA1109 | KIAA1109 | Protein Coding | GC04P122152 | 10.76 |
| WNT3 | Wnt Family Member 3 | Protein Coding | GC17M046762 | 10.76 |
| LDHA | Lactate Dehydrogenase A | Protein Coding | GC11P018394 | 10.75 |
| SUCLG1 | Succinate-CoA Ligase GDP/ADP-Forming Subunit Alpha | Protein Coding | GC02M084423 | 10.75 |
| HDAC6 | Histone Deacetylase 6 | Protein Coding | GC0XP048801 | 10.74 |
| JPH3 | Junctophilin 3 | Protein Coding | GC16P087601 | 10.74 |
| AGRN | Agrin | Protein Coding | GC01P001020 | 10.74 |
| DNAH9 | Dynein Axonemal Heavy Chain 9 | Protein Coding | GC17P011598 | 10.74 |
| MTHFD1 | Methylenetetrahydrofolate Dehydrogenase, Cyclohydrolase And Formyltetrahydrofolate Synthetase 1 | Protein Coding | GC14P064388 | 10.73 |
| KIF3A | Kinesin Family Member 3A | Protein Coding | GC05M132689 | 10.73 |
| ZEB1 | Zinc Finger E-Box Binding Homeobox 1 | Protein Coding | GC10P031318 | 10.72 |
| AKAP10 | A-Kinase Anchoring Protein 10 | Protein Coding | GC17M019904 | 10.72 |
| RFC1 | Replication Factor C Subunit 1 | Protein Coding | GC04M039291 | 10.72 |
| PPIF | Peptidylprolyl Isomerase F | Protein Coding | GC10P083661 | 10.72 |
| TRAF3IP2 | TRAF3 Interacting Protein 2 | Protein Coding | GC06M111555 | 10.71 |
| BSG | Basigin (Ok Blood Group) | Protein Coding | GC19P000571 | 10.7 |
| ADCY9 | Adenylate Cyclase 9 | Protein Coding | GC16M003953 | 10.7 |
| GLS | Glutaminase | Protein Coding | GC02P190880 | 10.7 |
| COQ8B | Coenzyme Q8B | Protein Coding | GC19M042476 | 10.7 |
| TNNI1 | Troponin I1, Slow Skeletal Type | Protein Coding | GC01M201404 | 10.69 |
| SFTPD | Surfactant Protein D | Protein Coding | GC10M079937 | 10.69 |
| PSMD12 | Proteasome 26S Subunit, Non-ATPase 12 | Protein Coding | GC17M067337 | 10.68 |
| APOC1 | Apolipoprotein C1 | Protein Coding | GC19P044914 | 10.67 |
| PCNA | Proliferating Cell Nuclear Antigen | Protein Coding | GC20M005114 | 10.67 |
| MIR324 | MicroRNA 324 | RNA Gene | GC17M007223 | 10.66 |
| DIO2 | Iodothyronine Deiodinase 2 | Protein Coding | GC14M080197 | 10.64 |
| MT-LIPCAR | Mitochondrially Encoded Long Non-Coding Cardiac Associated RNA | Uncategorized | GC00U936426 | 10.62 |
| MAP2 | Microtubule Associated Protein 2 | Protein Coding | GC02P209424 | 10.62 |
| MALAT1 | Metastasis Associated Lung Adenocarcinoma Transcript 1 | RNA Gene | GC11P065806 | 10.61 |
| MYO7A | Myosin VIIA | Protein Coding | GC11P077128 | 10.6 |
| GREM1 | Gremlin 1, DAN Family BMP Antagonist | Protein Coding | GC15P032720 | 10.59 |
| GSTT1 | Glutathione S-Transferase Theta 1 | Protein Coding | GC22Mi00270 | 10.59 |
| SLC27A6 | Solute Carrier Family 27 Member 6 | Protein Coding | GC05P128538 | 10.58 |
| ATP2A3 | ATPase Sarcoplasmic/Endoplasmic Reticulum Ca2+ Transporting 3 | Protein Coding | GC17M003923 | 10.58 |
| CXCL9 | C-X-C Motif Chemokine Ligand 9 | Protein Coding | GC04M076001 | 10.58 |
| TNFSF15 | TNF Superfamily Member 15 | Protein Coding | GC09M114784 | 10.58 |
| TTN-AS1 | TTN Antisense RNA 1 | RNA Gene | GC02P178521 | 10.57 |
| COL6A2 | Collagen Type VI Alpha 2 Chain | Protein Coding | GC21P046098 | 10.57 |
| PCSK2 | Proprotein Convertase Subtilisin/Kexin Type 2 | Protein Coding | GC20P017226 | 10.57 |
| ILK | Integrin Linked Kinase | Protein Coding | GC11P006581 | 10.56 |
| PVR | PVR Cell Adhesion Molecule | Protein Coding | GC19P044644 | 10.56 |
| ITGA3 | Integrin Subunit Alpha 3 | Protein Coding | GC17P050055 | 10.55 |
| GCM2 | Glial Cells Missing Transcription Factor 2 | Protein Coding | GC06M010873 | 10.55 |
| SELENBP1 | Selenium Binding Protein 1 | Protein Coding | GC01M151364 | 10.54 |
| PGK1 | Phosphoglycerate Kinase 1 | Protein Coding | GC0XP077928 | 10.53 |
| LAMA1 | Laminin Subunit Alpha 1 | Protein Coding | GC18M006941 | 10.52 |
| RMND1 | Required For Meiotic Nuclear Division 1 Homolog | Protein Coding | GC06M151404 | 10.5 |
| FURIN | Furin, Paired Basic Amino Acid Cleaving Enzyme | Protein Coding | GC15P090868 | 10.5 |
| KRT18 | Keratin 18 | Protein Coding | GC12P052948 | 10.49 |
| KCNQ3 | Potassium Voltage-Gated Channel Subfamily Q Member 3 | Protein Coding | GC08M132120 | 10.49 |
| ALG1 | ALG1 Chitobiosyldiphosphodolichol Beta-Mannosyltransferase | Protein Coding | GC16P005033 | 10.48 |
| CAPN10 | Calpain 10 | Protein Coding | GC02P240586 | 10.46 |
| CTNND1 | Catenin Delta 1 | Protein Coding | GC11P057788 | 10.45 |
| IL1RAPL2 | Interleukin 1 Receptor Accessory Protein Like 2 | Protein Coding | GC0XP104566 | 10.45 |
| MPLKIP | M-Phase Specific PLK1 Interacting Protein | Protein Coding | GC07M040126 | 10.44 |
| TOR1A | Torsin Family 1 Member A | Protein Coding | GC09M129812 | 10.44 |
| POR | Cytochrome P450 Oxidoreductase | Protein Coding | GC07P075899 | 10.44 |
| PRKCB | Protein Kinase C Beta | Protein Coding | GC16P023892 | 10.43 |
| NUP107 | Nucleoporin 107 | Protein Coding | GC12P068686 | 10.43 |
| GYPA | Glycophorin A (MNS Blood Group) | Protein Coding | GC04M144109 | 10.42 |
| MRE11 | MRE11 Homolog, Double Strand Break Repair Nuclease | Protein Coding | GC11M094444 | 10.4 |
| ITGA1 | Integrin Subunit Alpha 1 | Protein Coding | GC05P052788 | 10.4 |
| CANT1 | Calcium Activated Nucleotidase 1 | Protein Coding | GC17M078992 | 10.39 |
| PCDH15 | Protocadherin Related 15 | Protein Coding | GC10M053802 | 10.39 |
| TBXA2R | Thromboxane A2 Receptor | Protein Coding | GC19M003594 | 10.39 |
| ECM1 | Extracellular Matrix Protein 1 | Protein Coding | GC01P150508 | 10.39 |
| DZIP1L | DAZ Interacting Zinc Finger Protein 1 Like | Protein Coding | GC03M138061 | 10.38 |
| MIR130A | MicroRNA 130a | RNA Gene | GC11P057641 | 10.38 |
| ODC1 | Ornithine Decarboxylase 1 | Protein Coding | GC02M010432 | 10.38 |
| RAB27A | RAB27A, Member RAS Oncogene Family | Protein Coding | GC15M055202 | 10.37 |
| FREM1 | FRAS1 Related Extracellular Matrix 1 | Protein Coding | GC09M014730 | 10.36 |
| IGF2-AS | IGF2 Antisense RNA | RNA Gene | GC11P002140 | 10.36 |
| SUGCT | Succinyl-CoA:Glutarate-CoA Transferase | Protein Coding | GC07P040134 | 10.36 |
| STN1 | STN1 Subunit Of CST Complex | Protein Coding | GC10M103878 | 10.36 |
| BPGM | Bisphosphoglycerate Mutase | Protein Coding | GC07P134646 | 10.35 |
| DRD1 | Dopamine Receptor D1 | Protein Coding | GC05M175440 | 10.35 |
| PDLIM3 | PDZ And LIM Domain 3 | Protein Coding | GC04M185500 | 10.33 |
| UROD | Uroporphyrinogen Decarboxylase | Protein Coding | GC01P045012 | 10.32 |
| EPRS1 | Glutamyl-Prolyl-TRNA Synthetase 1 | Protein Coding | GC01M219969 | 10.32 |
| GJA3 | Gap Junction Protein Alpha 3 | Protein Coding | GC13M020139 | 10.31 |
| CD274 | CD274 Molecule | Protein Coding | GC09P005450 | 10.31 |
| NCAM1 | Neural Cell Adhesion Molecule 1 | Protein Coding | GC11P112961 | 10.3 |
| PTS | 6-Pyruvoyltetrahydropterin Synthase | Protein Coding | GC11P112226 | 10.3 |
| HAVCR1 | Hepatitis A Virus Cellular Receptor 1 | Protein Coding | GC05M157028 | 10.3 |
| NFATC4 | Nuclear Factor Of Activated T Cells 4 | Protein Coding | GC14P024365 | 10.29 |
| RNASEH2A | Ribonuclease H2 Subunit A | Protein Coding | GC19P012809 | 10.28 |
| TJP2 | Tight Junction Protein 2 | Protein Coding | GC09P069121 | 10.28 |
| TCN2 | Transcobalamin 2 | Protein Coding | GC22P030606 | 10.27 |
| RHOB | Ras Homolog Family Member B | Protein Coding | GC02P020447 | 10.26 |
| HBEGF | Heparin Binding EGF Like Growth Factor | Protein Coding | GC05M140332 | 10.26 |
| SIN3A | SIN3 Transcription Regulator Family Member A | Protein Coding | GC15M075369 | 10.26 |
| MAFB | MAF BZIP Transcription Factor B | Protein Coding | GC20M040685 | 10.25 |
| KISS1 | KiSS-1 Metastasis Suppressor | Protein Coding | GC01M204190 | 10.25 |
| FHIT | Fragile Histidine Triad Diadenosine Triphosphatase | Protein Coding | GC03M059747 | 10.25 |
| GALNT11 | Polypeptide N-Acetylgalactosaminyltransferase 11 | Protein Coding | GC07P152025 | 10.25 |
| ANXA6 | Annexin A6 | Protein Coding | GC05M151077 | 10.25 |
| PAX5 | Paired Box 5 | Protein Coding | GC09M036828 | 10.24 |
| WWOX | WW Domain Containing Oxidoreductase | Protein Coding | GC16P078099 | 10.23 |
| MT-TT | Mitochondrially Encoded TRNA-Thr (ACN) | RNA Gene | GCMTP015890 | 10.23 |
| SCD | Stearoyl-CoA Desaturase | Protein Coding | GC10P100347 | 10.21 |
| HTRA2 | HtrA Serine Peptidase 2 | Protein Coding | GC02P074529 | 10.2 |
| IL4R | Interleukin 4 Receptor | Protein Coding | GC16P027325 | 10.2 |
| BACE1-AS | BACE1 Antisense RNA | RNA Gene | GC11P117304 | 10.19 |
| IGHE | Immunoglobulin Heavy Constant Epsilon | Protein Coding | GC14M109515 | 10.19 |
| STAP1 | Signal Transducing Adaptor Family Member 1 | Protein Coding | GC04P067558 | 10.19 |
| MAPK7 | Mitogen-Activated Protein Kinase 7 | Protein Coding | GC17P019379 | 10.18 |
| ADRA1D | Adrenoceptor Alpha 1D | Protein Coding | GC20M004220 | 10.17 |
| EFEMP1 | EGF Containing Fibulin Extracellular Matrix Protein 1 | Protein Coding | GC02M055865 | 10.17 |
| FGF9 | Fibroblast Growth Factor 9 | Protein Coding | GC13P021671 | 10.16 |
| SYNM | Synemin | Protein Coding | GC15P099098 | 10.16 |
| ADD3 | Adducin 3 | Protein Coding | GC10P109996 | 10.16 |
| PHGDH | Phosphoglycerate Dehydrogenase | Protein Coding | GC01P119660 | 10.15 |
| ADAMTSL4 | ADAMTS Like 4 | Protein Coding | GC01P150549 | 10.14 |
| KCNMB1 | Potassium Calcium-Activated Channel Subfamily M Regulatory Beta Subunit 1 | Protein Coding | GC05M170374 | 10.14 |
| TAP1 | Transporter 1, ATP Binding Cassette Subfamily B Member | Protein Coding | GC06M032865 | 10.14 |
| MIR378A | MicroRNA 378a | RNA Gene | GC05P149732 | 10.13 |
| VANGL2 | VANGL Planar Cell Polarity Protein 2 | Protein Coding | GC01P160370 | 10.12 |
| PPP1R1B | Protein Phosphatase 1 Regulatory Inhibitor Subunit 1B | Protein Coding | GC17P039626 | 10.1 |
| CLPB | Caseinolytic Mitochondrial Matrix Peptidase Chaperone Subunit B | Protein Coding | GC11M072292 | 10.09 |
| NEUROD1 | Neuronal Differentiation 1 | Protein Coding | GC02M181673 | 10.09 |
| GPX4 | Glutathione Peroxidase 4 | Protein Coding | GC19P001103 | 10.08 |
| KCNQ4 | Potassium Voltage-Gated Channel Subfamily Q Member 4 | Protein Coding | GC01P040784 | 10.08 |
| OGT | O-Linked N-Acetylglucosamine (GlcNAc) Transferase | Protein Coding | GC0XP071534 | 10.07 |
| CD3D | CD3d Molecule | Protein Coding | GC11M118338 | 10.07 |
| NR1H3 | Nuclear Receptor Subfamily 1 Group H Member 3 | Protein Coding | GC11P047248 | 10.06 |
| HSPB7 | Heat Shock Protein Family B (Small) Member 7 | Protein Coding | GC01M016014 | 10.06 |
| GPR35 | G Protein-Coupled Receptor 35 | Protein Coding | GC02P240605 | 10.06 |
| BRINP3 | BMP/Retinoic Acid Inducible Neural Specific 3 | Protein Coding | GC01M190067 | 10.05 |
| MIR127 | MicroRNA 127 | RNA Gene | GC14P104580 | 10.05 |
| RXRA | Retinoid X Receptor Alpha | Protein Coding | GC09P134317 | 10.05 |
| CHRNA3 | Cholinergic Receptor Nicotinic Alpha 3 Subunit | Protein Coding | GC15M078594 | 10.04 |
| CH25H | Cholesterol 25-Hydroxylase | Protein Coding | GC10M089205 | 10.04 |
| RPS10 | Ribosomal Protein S10 | Protein Coding | GC06M042209 | 10.04 |
| OBSL1 | Obscurin Like Cytoskeletal Adaptor 1 | Protein Coding | GC02M219550 | 10.04 |
| SPON1 | Spondin 1 | Protein Coding | GC11P013940 | 10.04 |
| RBFOX1 | RNA Binding Fox-1 Homolog 1 | Protein Coding | GC16P005240 | 10.02 |
| GK | Glycerol Kinase | Protein Coding | GC0XP030671 | 10 |
| CD68 | CD68 Molecule | Protein Coding | GC17P007579 | 9.98 |
| KISS1R | KISS1 Receptor | Protein Coding | GC19P000917 | 9.96 |
| NAT2 | N-Acetyltransferase 2 | Protein Coding | GC08P018391 | 9.96 |
| D2HGDH | D-2-Hydroxyglutarate Dehydrogenase | Protein Coding | GC02P241734 | 9.96 |
| GRIN1 | Glutamate Ionotropic Receptor NMDA Type Subunit 1 | Protein Coding | GC09P137138 | 9.95 |
| XRCC5 | X-Ray Repair Cross Complementing 5 | Protein Coding | GC02P216107 | 9.95 |
| AQP4 | Aquaporin 4 | Protein Coding | GC18M026852 | 9.95 |
| TXN | Thioredoxin | Protein Coding | GC09M110243 | 9.95 |
| HMCN1 | Hemicentin 1 | Protein Coding | GC01P185734 | 9.94 |
| TAP2 | Transporter 2, ATP Binding Cassette Subfamily B Member | Protein Coding | GC06M032821 | 9.94 |
| AKR1B1 | Aldo-Keto Reductase Family 1 Member B | Protein Coding | GC07M134442 | 9.93 |
| AMT | Aminomethyltransferase | Protein Coding | GC03M049417 | 9.92 |
| PROK2 | Prokineticin 2 | Protein Coding | GC03M071771 | 9.92 |
| ADH1B | Alcohol Dehydrogenase 1B (Class I), Beta Polypeptide | Protein Coding | GC04M099304 | 9.92 |
| SLC30A8 | Solute Carrier Family 30 Member 8 | Protein Coding | GC08P116950 | 9.92 |
| MMACHC | Metabolism Of Cobalamin Associated C | Protein Coding | GC01P045500 | 9.92 |
| PLAUR | Plasminogen Activator, Urokinase Receptor | Protein Coding | GC19M043646 | 9.92 |
| KCNJ12 | Potassium Inwardly Rectifying Channel Subfamily J Member 12 | Protein Coding | GC17P026750 | 9.91 |
| MIR148A | MicroRNA 148a | RNA Gene | GC07M025993 | 9.91 |
| CD244 | CD244 Molecule | Protein Coding | GC01M160830 | 9.89 |
| MIR23B | MicroRNA 23b | RNA Gene | GC09P095085 | 9.89 |
| NTS | Neurotensin | Protein Coding | GC12P085876 | 9.88 |
| SCAP | SREBF Chaperone | Protein Coding | GC03M047413 | 9.88 |
| PDE4A | Phosphodiesterase 4A | Protein Coding | GC19P010416 | 9.87 |
| CD209 | CD209 Molecule | Protein Coding | GC19M007739 | 9.87 |
| MYH7B | Myosin Heavy Chain 7B | Protein Coding | GC20P034956 | 9.87 |
| SIX5 | SIX Homeobox 5 | Protein Coding | GC19M045764 | 9.83 |
| HS6ST1 | Heparan Sulfate 6-O-Sulfotransferase 1 | Protein Coding | GC02M128236 | 9.83 |
| PHB | Prohibitin | Protein Coding | GC17M049404 | 9.82 |
| NRTN | Neurturin | Protein Coding | GC19P005805 | 9.82 |
| KRT1 | Keratin 1 | Protein Coding | GC12M052674 | 9.81 |
| PRPF3 | Pre-MRNA Processing Factor 3 | Protein Coding | GC01P150321 | 9.81 |
| COL6A3 | Collagen Type VI Alpha 3 Chain | Protein Coding | GC02M237324 | 9.81 |
| FHOD3 | Formin Homology 2 Domain Containing 3 | Protein Coding | GC18P036297 | 9.81 |
| MIR197 | MicroRNA 197 | RNA Gene | GC01P109549 | 9.8 |
| CFP | Complement Factor Properdin | Protein Coding | GC0XM047624 | 9.8 |
| MECOM | MDS1 And EVI1 Complex Locus | Protein Coding | GC03M169083 | 9.79 |
| GLUL | Glutamate-Ammonia Ligase | Protein Coding | GC01M182350 | 9.79 |
| NDUFB10 | NADH:Ubiquinone Oxidoreductase Subunit B10 | Protein Coding | GC16P002430 | 9.77 |
| FGFR4 | Fibroblast Growth Factor Receptor 4 | Protein Coding | GC05P177086 | 9.76 |
| MIRLET7B | MicroRNA Let-7b | RNA Gene | GC22P046119 | 9.74 |
| EIF2B4 | Eukaryotic Translation Initiation Factor 2B Subunit Delta | Protein Coding | GC02M027364 | 9.74 |
| ACAD8 | Acyl-CoA Dehydrogenase Family Member 8 | Protein Coding | GC11P134253 | 9.73 |
| RPL27 | Ribosomal Protein L27 | Protein Coding | GC17P042998 | 9.73 |
| TPH2 | Tryptophan Hydroxylase 2 | Protein Coding | GC12P071938 | 9.73 |
| TNFSF10 | TNF Superfamily Member 10 | Protein Coding | GC03M172505 | 9.72 |
| PLCG2 | Phospholipase C Gamma 2 | Protein Coding | GC16P081773 | 9.71 |
| NQO1 | NAD(P)H Quinone Dehydrogenase 1 | Protein Coding | GC16M069706 | 9.71 |
| PNP | Purine Nucleoside Phosphorylase | Protein Coding | GC14P020468 | 9.7 |
| MIR494 | MicroRNA 494 | RNA Gene | GC14P104814 | 9.69 |
| ATG16L1 | Autophagy Related 16 Like 1 | Protein Coding | GC02P233215 | 9.67 |
| MIR33B | MicroRNA 33b | RNA Gene | GC17M017813 | 9.66 |
| MIRLET7D | MicroRNA Let-7d | RNA Gene | GC09P094178 | 9.66 |
| TMSB4X | Thymosin Beta 4 X-Linked | Protein Coding | GC0XP012975 | 9.65 |
| ACTG2 | Actin Gamma 2, Smooth Muscle | Protein Coding | GC02P073892 | 9.64 |
| APBB1 | Amyloid Beta Precursor Protein Binding Family B Member 1 | Protein Coding | GC11M006396 | 9.63 |
| FCN3 | Ficolin 3 | Protein Coding | GC01M027379 | 9.63 |
| LIPI | Lipase I | Protein Coding | GC21M014108 | 9.62 |
| TIMP4 | TIMP Metallopeptidase Inhibitor 4 | Protein Coding | GC03M012153 | 9.61 |
| CTSG | Cathepsin G | Protein Coding | GC14M024573 | 9.6 |
| MIRLET7A1 | MicroRNA Let-7a-1 | RNA Gene | GC09P094175 | 9.6 |
| CERT1 | Ceramide Transporter 1 | Protein Coding | GC05M075356 | 9.6 |
| MYDGF | Myeloid Derived Growth Factor | Protein Coding | GC19M004641 | 9.58 |
| IAPP | Islet Amyloid Polypeptide | Protein Coding | GC12P021354 | 9.57 |
| CSTB | Cystatin B | Protein Coding | GC21M043772 | 9.57 |
| MIR30B | MicroRNA 30b | RNA Gene | GC08M134800 | 9.56 |
| COX4I1 | Cytochrome C Oxidase Subunit 4I1 | Protein Coding | GC16P085798 | 9.56 |
| NID1 | Nidogen 1 | Protein Coding | GC01M235975 | 9.54 |
| RNASEH2B | Ribonuclease H2 Subunit B | Protein Coding | GC13P050909 | 9.54 |
| IL16 | Interleukin 16 | Protein Coding | GC15P081159 | 9.54 |
| CXCL1 | C-X-C Motif Chemokine Ligand 1 | Protein Coding | GC04P073869 | 9.52 |
| AOMS1 | Abdominal Obesity-Metabolic Syndrome QTL1 | Genetic Locus | GC03U990183 | 9.52 |
| FBXO32 | F-Box Protein 32 | Protein Coding | GC08M123498 | 9.51 |
| MIR335 | MicroRNA 335 | RNA Gene | GC07P130496 | 9.51 |
| PSMB4 | Proteasome 20S Subunit Beta 4 | Protein Coding | GC01P151372 | 9.51 |
| H3-2 | H3.2 Histone (Putative) | Protein Coding | GC01M143894 | 9.51 |
| LARGE1 | LARGE Xylosyl- And Glucuronyltransferase 1 | Protein Coding | GC22M033163 | 9.49 |
| SHANK3 | SH3 And Multiple Ankyrin Repeat Domains 3 | Protein Coding | GC22P050674 | 9.49 |
| AXL | AXL Receptor Tyrosine Kinase | Protein Coding | GC19P041219 | 9.48 |
| CDH11 | Cadherin 11 | Protein Coding | GC16M064882 | 9.47 |
| LIG3 | DNA Ligase 3 | Protein Coding | GC17P034980 | 9.46 |
| MYBPC2 | Myosin Binding Protein C2 | Protein Coding | GC19P050432 | 9.46 |
| MIR192 | MicroRNA 192 | RNA Gene | GC11M064891 | 9.46 |
| TAPVR1 | Total Anomalous Pulmonary Venous Return 1 | Genetic Locus | GC04U990048 | 9.45 |
| KCNJ13 | Potassium Inwardly Rectifying Channel Subfamily J Member 13 | Protein Coding | GC02M232765 | 9.44 |
| YWHAQ | Tyrosine 3-Monooxygenase/Tryptophan 5-Monooxygenase Activation Protein Theta | Protein Coding | GC02M009583 | 9.44 |
| PSMB9 | Proteasome 20S Subunit Beta 9 | Protein Coding | GC06P047342 | 9.44 |
| LAMP1 | Lysosomal Associated Membrane Protein 1 | Protein Coding | GC13P113297 | 9.44 |
| RARA | Retinoic Acid Receptor Alpha | Protein Coding | GC17P040309 | 9.44 |
| PDPK1 | 3-Phosphoinositide Dependent Protein Kinase 1 | Protein Coding | GC16P002537 | 9.43 |
| RLBP1 | Retinaldehyde Binding Protein 1 | Protein Coding | GC15M089209 | 9.43 |
| BMP1 | Bone Morphogenetic Protein 1 | Protein Coding | GC08P022164 | 9.42 |
| CNTNAP2 | Contactin Associated Protein 2 | Protein Coding | GC07P146116 | 9.41 |
| AGGF1 | Angiogenic Factor With G-Patch And FHA Domains 1 | Protein Coding | GC05P077029 | 9.39 |
| MIR24-1 | MicroRNA 24-1 | RNA Gene | GC09P095086 | 9.39 |
| BIRC3 | Baculoviral IAP Repeat Containing 3 | Protein Coding | GC11P102317 | 9.36 |
| CASP7 | Caspase 7 | Protein Coding | GC10P113679 | 9.36 |
| GOSR2 | Golgi SNAP Receptor Complex Member 2 | Protein Coding | GC17P046924 | 9.34 |
| PEPD | Peptidase D | Protein Coding | GC19M033386 | 9.33 |
| HDC | Histidine Decarboxylase | Protein Coding | GC15M050241 | 9.33 |
| ADORA3 | Adenosine A3 Receptor | Protein Coding | GC01M111499 | 9.32 |
| ANKRD2 | Ankyrin Repeat Domain 2 | Protein Coding | GC10P097572 | 9.31 |
| MTHFD1L | Methylenetetrahydrofolate Dehydrogenase (NADP+ Dependent) 1 Like | Protein Coding | GC06P150865 | 9.31 |
| MIR338 | MicroRNA 338 | RNA Gene | GC17M081126 | 9.31 |
| SPATA7 | Spermatogenesis Associated 7 | Protein Coding | GC14P088384 | 9.3 |
| NPR3 | Natriuretic Peptide Receptor 3 | Protein Coding | GC05P032689 | 9.3 |
| CARS2 | Cysteinyl-TRNA Synthetase 2, Mitochondrial | Protein Coding | GC13M110641 | 9.29 |
| TGFBI | Transforming Growth Factor Beta Induced | Protein Coding | GC05P136027 | 9.27 |
| CYP2J2 | Cytochrome P450 Family 2 Subfamily J Member 2 | Protein Coding | GC01M059893 | 9.27 |
| IFNGR2 | Interferon Gamma Receptor 2 | Protein Coding | GC21P033402 | 9.27 |
| MASP1 | Mannan Binding Lectin Serine Peptidase 1 | Protein Coding | GC03M187216 | 9.26 |
| BCAR1 | BCAR1 Scaffold Protein, Cas Family Member | Protein Coding | GC16M075228 | 9.26 |
| MIR183 | MicroRNA 183 | RNA Gene | GC07M129789 | 9.24 |
| MIR181B1 | MicroRNA 181b-1 | RNA Gene | GC01M198858 | 9.24 |
| SHC1 | SHC Adaptor Protein 1 | Protein Coding | GC01M154962 | 9.22 |
| CCND2 | Cyclin D2 | Protein Coding | GC12P008103 | 9.22 |
| P4HB | Prolyl 4-Hydroxylase Subunit Beta | Protein Coding | GC17M081843 | 9.21 |
| IL9 | Interleukin 9 | Protein Coding | GC05M135891 | 9.21 |
| CENPE | Centromere Protein E | Protein Coding | GC04M103105 | 9.21 |
| SLPI | Secretory Leukocyte Peptidase Inhibitor | Protein Coding | GC20M045252 | 9.2 |
| MAGI2 | Membrane Associated Guanylate Kinase, WW And PDZ Domain Containing 2 | Protein Coding | GC07M078017 | 9.2 |
| ADK | Adenosine Kinase | Protein Coding | GC10P074152 | 9.19 |
| CCK | Cholecystokinin | Protein Coding | GC03M042274 | 9.19 |
| ATP1B1 | ATPase Na+/K+ Transporting Subunit Beta 1 | Protein Coding | GC01P169105 | 9.19 |
| FUT2 | Fucosyltransferase 2 | Protein Coding | GC19P048695 | 9.19 |
| TARS1 | Threonyl-TRNA Synthetase 1 | Protein Coding | GC05P033441 | 9.19 |
| CLCN4 | Chloride Voltage-Gated Channel 4 | Protein Coding | GC0XP010085 | 9.18 |
| BHMT | Betaine--Homocysteine S-Methyltransferase | Protein Coding | GC05P079111 | 9.17 |
| IL18BP | Interleukin 18 Binding Protein | Protein Coding | GC11P071998 | 9.17 |
| WARS2 | Tryptophanyl TRNA Synthetase 2, Mitochondrial | Protein Coding | GC01M119031 | 9.16 |
| EDN2 | Endothelin 2 | Protein Coding | GC01M041478 | 9.15 |
| IGFBP2 | Insulin Like Growth Factor Binding Protein 2 | Protein Coding | GC02P216632 | 9.15 |
| APELA | Apelin Receptor Early Endogenous Ligand | Protein Coding | GC04P164877 | 9.15 |
| DDX59 | DEAD-Box Helicase 59 | Protein Coding | GC01M200594 | 9.14 |
| GRK5 | G Protein-Coupled Receptor Kinase 5 | Protein Coding | GC10P119207 | 9.14 |
| FNDC5 | Fibronectin Type III Domain Containing 5 | Protein Coding | GC01M032864 | 9.13 |
| KIF2C | Kinesin Family Member 2C | Protein Coding | GC01P044739 | 9.13 |
| MYLIP | Myosin Regulatory Light Chain Interacting Protein | Protein Coding | GC06P016129 | 9.13 |
| SPRY2 | Sprouty RTK Signaling Antagonist 2 | Protein Coding | GC13M080335 | 9.11 |
| KYNU | Kynureninase | Protein Coding | GC02P142877 | 9.09 |
| KRT8 | Keratin 8 | Protein Coding | GC12M052897 | 9.08 |
| TLR10 | Toll Like Receptor 10 | Protein Coding | GC04M038773 | 9.08 |
| CACNB1 | Calcium Voltage-Gated Channel Auxiliary Subunit Beta 1 | Protein Coding | GC17M039173 | 9.08 |
| TPH1 | Tryptophan Hydroxylase 1 | Protein Coding | GC11M018040 | 9.08 |
| NRON | Non-Coding Repressor Of NFAT | RNA Gene | GC09M126407 | 9.06 |
| CCR3 | C-C Motif Chemokine Receptor 3 | Protein Coding | GC03P046227 | 9.06 |
| FGF1 | Fibroblast Growth Factor 1 | Protein Coding | GC05M142555 | 9.05 |
| CAMTA1 | Calmodulin Binding Transcription Activator 1 | Protein Coding | GC01P006845 | 9.04 |
| HLA-DRB5 | Major Histocompatibility Complex, Class II, DR Beta 5 | Protein Coding | GC06M032683 | 9.04 |
| TELO2 | Telomere Maintenance 2 | Protein Coding | GC16P001493 | 9.03 |
| ALPP | Alkaline Phosphatase, Placental | Protein Coding | GC02P232378 | 9.03 |
| IDO1 | Indoleamine 2,3-Dioxygenase 1 | Protein Coding | GC08P039891 | 9.02 |
| TCF21 | Transcription Factor 21 | Protein Coding | GC06P133889 | 9.02 |
| COG5 | Component Of Oligomeric Golgi Complex 5 | Protein Coding | GC07M107201 | 9.01 |
| MUC16 | Mucin 16, Cell Surface Associated | Protein Coding | GC19M008848 | 9.01 |
| IVD | Isovaleryl-CoA Dehydrogenase | Protein Coding | GC15P040405 | 9.01 |
| FADS1 | Fatty Acid Desaturase 1 | Protein Coding | GC11M061799 | 9.01 |
| TLN1 | Talin 1 | Protein Coding | GC09M035687 | 9.01 |
| PRICKLE1 | Prickle Planar Cell Polarity Protein 1 | Protein Coding | GC12M042456 | 9 |
| CAMK2G | Calcium/Calmodulin Dependent Protein Kinase II Gamma | Protein Coding | GC10M073812 | 8.99 |
| ADIPOR1 | Adiponectin Receptor 1 | Protein Coding | GC01M202940 | 8.99 |
| THRIL | TNF And HNRNPL Related Immunoregulatory Long Non-Coding RNA | RNA Gene | GC12M125025 | 8.98 |
| SHOX | Short Stature Homeobox | Protein Coding | GC0XP000624 | 8.97 |
| GPC6 | Glypican 6 | Protein Coding | GC13P093226 | 8.96 |
| FAM13A | Family With Sequence Similarity 13 Member A | Protein Coding | GC04M088725 | 8.95 |
| SEMA5A | Semaphorin 5A | Protein Coding | GC05M009036 | 8.94 |
| ATR | ATR Serine/Threonine Kinase | Protein Coding | GC03M142449 | 8.93 |
| INPPL1 | Inositol Polyphosphate Phosphatase Like 1 | Protein Coding | GC11P072223 | 8.93 |
| BECN1 | Beclin 1 | Protein Coding | GC17M042810 | 8.93 |
| CD69 | CD69 Molecule | Protein Coding | GC12M013857 | 8.92 |
| CYSLTR2 | Cysteinyl Leukotriene Receptor 2 | Protein Coding | GC13P048653 | 8.91 |
| GPX3 | Glutathione Peroxidase 3 | Protein Coding | GC05P150997 | 8.91 |
| DSPP | Dentin Sialophosphoprotein | Protein Coding | GC04P087608 | 8.91 |
| ENPP3 | Ectonucleotide Pyrophosphatase/Phosphodiesterase 3 | Protein Coding | GC06P131617 | 8.9 |
| EZR | Ezrin | Protein Coding | GC06M158765 | 8.9 |
| CALB2 | Calbindin 2 | Protein Coding | GC16P071392 | 8.89 |
| DOK7 | Docking Protein 7 | Protein Coding | GC04P003465 | 8.88 |
| MYOM1 | Myomesin 1 | Protein Coding | GC18M003066 | 8.88 |
| NR1H2 | Nuclear Receptor Subfamily 1 Group H Member 2 | Protein Coding | GC19P050329 | 8.88 |
| CCR7 | C-C Motif Chemokine Receptor 7 | Protein Coding | GC17M040556 | 8.87 |
| ADAMTS17 | ADAM Metallopeptidase With Thrombospondin Type 1 Motif 17 | Protein Coding | GC15M099971 | 8.87 |
| CYP24A1 | Cytochrome P450 Family 24 Subfamily A Member 1 | Protein Coding | GC20M054153 | 8.86 |
| CD38 | CD38 Molecule | Protein Coding | GC04P015779 | 8.86 |
| PRKCH | Protein Kinase C Eta | Protein Coding | GC14P061187 | 8.85 |
| SH2B1 | SH2B Adaptor Protein 1 | Protein Coding | GC16P029024 | 8.85 |
| RELB | RELB Proto-Oncogene, NF-KB Subunit | Protein Coding | GC19P045002 | 8.85 |
| FSTL1 | Follistatin Like 1 | Protein Coding | GC03M120392 | 8.84 |
| RARS2 | Arginyl-TRNA Synthetase 2, Mitochondrial | Protein Coding | GC06M087514 | 8.84 |
| HULC | Hepatocellular Carcinoma Up-Regulated Long Non-Coding RNA | RNA Gene | GC06P008438 | 8.84 |
| BMP7 | Bone Morphogenetic Protein 7 | Protein Coding | GC20M057168 | 8.84 |
| PROS1 | Protein S | Protein Coding | GC03M093873 | 8.83 |
| GATM | Glycine Amidinotransferase | Protein Coding | GC15M045361 | 8.83 |
| MGAM | Maltase-Glucoamylase | Protein Coding | GC07P145052 | 8.82 |
| TNFRSF10B | TNF Receptor Superfamily Member 10b | Protein Coding | GC08M023020 | 8.81 |
| KLF4 | Kruppel Like Factor 4 | Protein Coding | GC09M107484 | 8.8 |
| HSPA5 | Heat Shock Protein Family A (Hsp70) Member 5 | Protein Coding | GC09M125234 | 8.8 |
| OPRM1 | Opioid Receptor Mu 1 | Protein Coding | GC06P154075 | 8.79 |
| CD247 | CD247 Molecule | Protein Coding | GC01M167399 | 8.79 |
| MRPS14 | Mitochondrial Ribosomal Protein S14 | Protein Coding | GC01M175010 | 8.79 |
| IL11 | Interleukin 11 | Protein Coding | GC19M055364 | 8.78 |
| MAP3K1 | Mitogen-Activated Protein Kinase Kinase Kinase 1 | Protein Coding | GC05P056815 | 8.78 |
| CFL1 | Cofilin 1 | Protein Coding | GC11M065823 | 8.77 |
| AKAP13 | A-Kinase Anchoring Protein 13 | Protein Coding | GC15P085381 | 8.77 |
| PDXK | Pyridoxal Kinase | Protein Coding | GC21P043719 | 8.77 |
| FGF14 | Fibroblast Growth Factor 14 | Protein Coding | GC13M101710 | 8.77 |
| ADIPOR2 | Adiponectin Receptor 2 | Protein Coding | GC12P001670 | 8.77 |
| MIR30D | MicroRNA 30d | RNA Gene | GC08M134804 | 8.76 |
| LRRC10 | Leucine Rich Repeat Containing 10 | Protein Coding | GC12M069608 | 8.75 |
| PKP1 | Plakophilin 1 | Protein Coding | GC01P201252 | 8.74 |
| PER2 | Period Circadian Regulator 2 | Protein Coding | GC02M238244 | 8.74 |
| JCAD | Junctional Cadherin 5 Associated | Protein Coding | GC10M030012 | 8.73 |
| SDC1 | Syndecan 1 | Protein Coding | GC02M020200 | 8.72 |
| CACNA2D3 | Calcium Voltage-Gated Channel Auxiliary Subunit Alpha2delta 3 | Protein Coding | GC03P054156 | 8.69 |
| CCL17 | C-C Motif Chemokine Ligand 17 | Protein Coding | GC16P057400 | 8.69 |
| PML | PML Nuclear Body Scaffold | Protein Coding | GC15P073994 | 8.68 |
| AMBP | Alpha-1-Microglobulin/Bikunin Precursor | Protein Coding | GC09M114060 | 8.68 |
| IL1RL1 | Interleukin 1 Receptor Like 1 | Protein Coding | GC02P102294 | 8.67 |
| MYOM2 | Myomesin 2 | Protein Coding | GC08P002045 | 8.67 |
| P2RX1 | Purinergic Receptor P2X 1 | Protein Coding | GC17M003896 | 8.66 |
| LACTB | Lactamase Beta | Protein Coding | GC15P073008 | 8.66 |
| WFDC21P | WAP Four-Disulfide Core Domain 21, Pseudogene | Pseudogene | GC17M060085 | 8.66 |
| PRKDC | Protein Kinase, DNA-Activated, Catalytic Subunit | Protein Coding | GC08M047773 | 8.65 |
| ACP1 | Acid Phosphatase 1 | Protein Coding | GC02P000254 | 8.64 |
| TUBB4A | Tubulin Beta 4A Class IVa | Protein Coding | GC19M006496 | 8.64 |
| TBX6 | T-Box Transcription Factor 6 | Protein Coding | GC16M030085 | 8.64 |
| MIRLET7E | MicroRNA Let-7e | RNA Gene | GC19P051718 | 8.64 |
| SUN1 | Sad1 And UNC84 Domain Containing 1 | Protein Coding | GC07P000857 | 8.63 |
| TUBB1 | Tubulin Beta 1 Class VI | Protein Coding | GC20P059020 | 8.63 |
| PDLIM5 | PDZ And LIM Domain 5 | Protein Coding | GC04P094451 | 8.63 |
| OCLN | Occludin | Protein Coding | GC05P069492 | 8.62 |
| CXCR2 | C-X-C Motif Chemokine Receptor 2 | Protein Coding | GC02P218125 | 8.62 |
| TSPO | Translocator Protein | Protein Coding | GC22P043151 | 8.62 |
| COL12A1 | Collagen Type XII Alpha 1 Chain | Protein Coding | GC06M075084 | 8.61 |
| ATIC | 5-Aminoimidazole-4-Carboxamide Ribonucleotide Formyltransferase/IMP Cyclohydrolase | Protein Coding | GC02P215311 | 8.61 |
| GJC1 | Gap Junction Protein Gamma 1 | Protein Coding | GC17M044800 | 8.59 |
| ITIH4 | Inter-Alpha-Trypsin Inhibitor Heavy Chain 4 | Protein Coding | GC03M052812 | 8.59 |
| EPHX1 | Epoxide Hydrolase 1 | Protein Coding | GC01P225810 | 8.59 |
| CIB1 | Calcium And Integrin Binding 1 | Protein Coding | GC15M090229 | 8.58 |
| TP53BP1 | Tumor Protein P53 Binding Protein 1 | Protein Coding | GC15M043403 | 8.58 |
| WNT7A | Wnt Family Member 7A | Protein Coding | GC03M015784 | 8.56 |
| CTSF | Cathepsin F | Protein Coding | GC11M066640 | 8.55 |
| ITGB5 | Integrin Subunit Beta 5 | Protein Coding | GC03M124761 | 8.55 |
| RPS6KB1 | Ribosomal Protein S6 Kinase B1 | Protein Coding | GC17P059893 | 8.54 |
| HOPX | HOP Homeobox | Protein Coding | GC04M056647 | 8.53 |
| NOTCH4 | Notch Receptor 4 | Protein Coding | GC06M032648 | 8.53 |
| MCL1 | MCL1 Apoptosis Regulator, BCL2 Family Member | Protein Coding | GC01M150707 | 8.53 |
| KLK3 | Kallikrein Related Peptidase 3 | Protein Coding | GC19P050854 | 8.52 |
| MSH3 | MutS Homolog 3 | Protein Coding | GC05P080654 | 8.51 |
| H19-ICR | H19/IGF2 Imprinting Control Region | Biological Region | GC11P001999 | 8.5 |
| GTF2E2 | General Transcription Factor IIE Subunit 2 | Protein Coding | GC08M030578 | 8.5 |
| MOCOS | Molybdenum Cofactor Sulfurase | Protein Coding | GC18P036187 | 8.49 |
| AIF1 | Allograft Inflammatory Factor 1 | Protein Coding | GC06P047304 | 8.49 |
| FGF3 | Fibroblast Growth Factor 3 | Protein Coding | GC11M069811 | 8.48 |
| GRK1 | G Protein-Coupled Receptor Kinase 1 | Protein Coding | GC13P113645 | 8.48 |
| MYLK3 | Myosin Light Chain Kinase 3 | Protein Coding | GC16M046714 | 8.47 |
| DAOA | D-Amino Acid Oxidase Activator | Protein Coding | GC13P105465 | 8.47 |
| SFRP4 | Secreted Frizzled Related Protein 4 | Protein Coding | GC07M037912 | 8.47 |
| LMO7 | LIM Domain 7 | Protein Coding | GC13P075620 | 8.46 |
| TXNIP | Thioredoxin Interacting Protein | Protein Coding | GC01M145992 | 8.46 |
| CD151 | CD151 Molecule (Raph Blood Group) | Protein Coding | GC11P000895 | 8.45 |
| HEY1 | Hes Related Family BHLH Transcription Factor With YRPW Motif 1 | Protein Coding | GC08M079764 | 8.44 |
| TRPV1 | Transient Receptor Potential Cation Channel Subfamily V Member 1 | Protein Coding | GC17M003565 | 8.44 |
| MX1 | MX Dynamin Like GTPase 1 | Protein Coding | GC21P041420 | 8.44 |
| FADS2 | Fatty Acid Desaturase 2 | Protein Coding | GC11P061792 | 8.44 |
| PUS1 | Pseudouridine Synthase 1 | Protein Coding | GC12P131929 | 8.42 |
| STUB1 | STIP1 Homology And U-Box Containing Protein 1 | Protein Coding | GC16P001405 | 8.42 |
| ARNTL | Aryl Hydrocarbon Receptor Nuclear Translocator Like | Protein Coding | GC11P013276 | 8.41 |
| SLC27A1 | Solute Carrier Family 27 Member 1 | Protein Coding | GC19P023305 | 8.41 |
| MAP2K7 | Mitogen-Activated Protein Kinase Kinase 7 | Protein Coding | GC19P007903 | 8.41 |
| HTR2B | 5-Hydroxytryptamine Receptor 2B | Protein Coding | GC02M231108 | 8.4 |
| DDAH2 | Dimethylarginine Dimethylaminohydrolase 2 | Protein Coding | GC06M031727 | 8.4 |
| RAC2 | Rac Family Small GTPase 2 | Protein Coding | GC22M037227 | 8.39 |
| TRPA1 | Transient Receptor Potential Cation Channel Subfamily A Member 1 | Protein Coding | GC08M072019 | 8.39 |
| GTF2H5 | General Transcription Factor IIH Subunit 5 | Protein Coding | GC06P158168 | 8.39 |
| FOXO3 | Forkhead Box O3 | Protein Coding | GC06P108559 | 8.39 |
| MIR25 | MicroRNA 25 | RNA Gene | GC07M100093 | 8.37 |
| MIR26B | MicroRNA 26b | RNA Gene | GC02P218402 | 8.37 |
| EIF2B2 | Eukaryotic Translation Initiation Factor 2B Subunit Beta | Protein Coding | GC14P075002 | 8.37 |
| RAB3GAP1 | RAB3 GTPase Activating Protein Catalytic Subunit 1 | Protein Coding | GC02P135052 | 8.36 |
| MIR371A | MicroRNA 371a | RNA Gene | GC19P053787 | 8.36 |
| IFNGR1 | Interferon Gamma Receptor 1 | Protein Coding | GC06M137197 | 8.36 |
| ACTN3 | Actinin Alpha 3 | Protein Coding | GC11P066546 | 8.35 |
| HRH2 | Histamine Receptor H2 | Protein Coding | GC05P175659 | 8.35 |
| COG6 | Component Of Oligomeric Golgi Complex 6 | Protein Coding | GC13P039655 | 8.34 |
| SLC22A3 | Solute Carrier Family 22 Member 3 | Protein Coding | GC06P160348 | 8.34 |
| SLC25A1 | Solute Carrier Family 25 Member 1 | Protein Coding | GC22M019176 | 8.33 |
| MYSM1 | Myb Like, SWIRM And MPN Domains 1 | Protein Coding | GC01M058654 | 8.33 |
| MS4A1 | Membrane Spanning 4-Domains A1 | Protein Coding | GC11P060474 | 8.33 |
| PRKAA1 | Protein Kinase AMP-Activated Catalytic Subunit Alpha 1 | Protein Coding | GC05M040759 | 8.32 |
| LMOD2 | Leiomodin 2 | Protein Coding | GC07P123655 | 8.32 |
| CYP4F2 | Cytochrome P450 Family 4 Subfamily F Member 2 | Protein Coding | GC19M015878 | 8.32 |
| MIR10B | MicroRNA 10b | RNA Gene | GC02P176150 | 8.32 |
| HMOX2 | Heme Oxygenase 2 | Protein Coding | GC16P004474 | 8.32 |
| MIR451A | MicroRNA 451a | RNA Gene | GC17M028861 | 8.3 |
| MIR331 | MicroRNA 331 | RNA Gene | GC12P095308 | 8.29 |
| DIABLO | Diablo IAP-Binding Mitochondrial Protein | Protein Coding | GC12M122208 | 8.29 |
| AMPD3 | Adenosine Monophosphate Deaminase 3 | Protein Coding | GC11P010309 | 8.28 |
| RGS5 | Regulator Of G Protein Signaling 5 | Protein Coding | GC01M163111 | 8.28 |
| UCP3 | Uncoupling Protein 3 | Protein Coding | GC11M074000 | 8.27 |
| QDPR | Quinoid Dihydropteridine Reductase | Protein Coding | GC04M017460 | 8.27 |
| NRAP | Nebulin Related Anchoring Protein | Protein Coding | GC10M113588 | 8.27 |
| RNPC3 | RNA Binding Region (RNP1, RRM) Containing 3 | Protein Coding | GC01P103525 | 8.26 |
| LIFR | LIF Receptor Subunit Alpha | Protein Coding | GC05M038475 | 8.26 |
| LEPQTL1 | Leptin, Serum Levels Of | Genetic Locus | GC02U903086 | 8.26 |
| AP3B1 | Adaptor Related Protein Complex 3 Subunit Beta 1 | Protein Coding | GC05M078000 | 8.26 |
| ALDH1A2 | Aldehyde Dehydrogenase 1 Family Member A2 | Protein Coding | GC15M060613 | 8.26 |
| ANKRD23 | Ankyrin Repeat Domain 23 | Protein Coding | GC02M096826 | 8.25 |
| CSNK1D | Casein Kinase 1 Delta | Protein Coding | GC17M082239 | 8.25 |
| ALOX15 | Arachidonate 15-Lipoxygenase | Protein Coding | GC17M004630 | 8.25 |
| CCL26 | C-C Motif Chemokine Ligand 26 | Protein Coding | GC07M075769 | 8.25 |
| TECPR2 | Tectonin Beta-Propeller Repeat Containing 2 | Protein Coding | GC14P102362 | 8.24 |
| GLMN | Glomulin, FKBP Associated Protein | Protein Coding | GC01M092246 | 8.24 |
| GCKR | Glucokinase Regulator | Protein Coding | GC02P027496 | 8.23 |
| IL22 | Interleukin 22 | Protein Coding | GC12M068248 | 8.23 |
| COL14A1 | Collagen Type XIV Alpha 1 Chain | Protein Coding | GC08P120062 | 8.23 |
| BUD23 | BUD23 RRNA Methyltransferase And Ribosome Maturation Factor | Protein Coding | GC07P073685 | 8.22 |
| NUP133 | Nucleoporin 133 | Protein Coding | GC01M229441 | 8.21 |
| ASCL1 | Achaete-Scute Family BHLH Transcription Factor 1 | Protein Coding | GC12P102957 | 8.2 |
| PHOSPHO1 | Phosphoethanolamine/Phosphocholine Phosphatase 1 | Protein Coding | GC17M049223 | 8.2 |
| PKD2L1 | Polycystin 2 Like 1, Transient Receptor Potential Cation Channel | Protein Coding | GC10M100288 | 8.19 |
| EIF2AK2 | Eukaryotic Translation Initiation Factor 2 Alpha Kinase 2 | Protein Coding | GC02M037099 | 8.19 |
| MLH3 | MutL Homolog 3 | Protein Coding | GC14M075013 | 8.19 |
| MIR224 | MicroRNA 224 | RNA Gene | GC0XM151958 | 8.18 |
| XRCC3 | X-Ray Repair Cross Complementing 3 | Protein Coding | GC14M103697 | 8.17 |
| NDST1 | N-Deacetylase And N-Sulfotransferase 1 | Protein Coding | GC05P150484 | 8.16 |
| STAG3 | Stromal Antigen 3 | Protein Coding | GC07P100177 | 8.15 |
| MIR1-2 | MicroRNA 1-2 | RNA Gene | GC18M021828 | 8.15 |
| SPAG17 | Sperm Associated Antigen 17 | Protein Coding | GC01M117953 | 8.15 |
| MIR19A | MicroRNA 19a | RNA Gene | GC13P091433 | 8.14 |
| PTGDS | Prostaglandin D2 Synthase | Protein Coding | GC09P136982 | 8.14 |
| IFI27 | Interferon Alpha Inducible Protein 27 | Protein Coding | GC14P094104 | 8.14 |
| IL17RC | Interleukin 17 Receptor C | Protein Coding | GC03P009917 | 8.13 |
| CEL | Carboxyl Ester Lipase | Protein Coding | GC09P133061 | 8.11 |
| FGF16 | Fibroblast Growth Factor 16 | Protein Coding | GC0XP077447 | 8.1 |
| SPRY4 | Sprouty RTK Signaling Antagonist 4 | Protein Coding | GC05M142310 | 8.1 |
| DMRT1 | Doublesex And Mab-3 Related Transcription Factor 1 | Protein Coding | GC09P000831 | 8.08 |
| NES | Nestin | Protein Coding | GC01M156668 | 8.08 |
| ENSG00000225544 |  | Pseudogene | GC22M021885 | 8.08 |
| SCGB1A1 | Secretoglobin Family 1A Member 1 | Protein Coding | GC11P062405 | 8.07 |
| MYO18B | Myosin XVIIIB | Protein Coding | GC22P025742 | 8.07 |
| DPYD | Dihydropyrimidine Dehydrogenase | Protein Coding | GC01M097015 | 8.07 |
| ITLN1 | Intelectin 1 | Protein Coding | GC01M160876 | 8.06 |
| EMC10 | ER Membrane Protein Complex Subunit 10 | Protein Coding | GC19P050476 | 8.06 |
| MYOM3 | Myomesin 3 | Protein Coding | GC01M024056 | 8.06 |
| CNNM2 | Cyclin And CBS Domain Divalent Metal Cation Transport Mediator 2 | Protein Coding | GC10P102918 | 8.05 |
| SIK1 | Salt Inducible Kinase 1 | Protein Coding | GC21M043414 | 8.05 |
| TRPC1 | Transient Receptor Potential Cation Channel Subfamily C Member 1 | Protein Coding | GC03P142724 | 8.05 |
| SOX17 | SRY-Box Transcription Factor 17 | Protein Coding | GC08P054457 | 8.03 |
| ABRAXAS2 | Abraxas 2, BRISC Complex Subunit | Protein Coding | GC10P124802 | 8.03 |
| MIR130B | MicroRNA 130b | RNA Gene | GC22P024903 | 8.03 |
| SGK1 | Serum/Glucocorticoid Regulated Kinase 1 | Protein Coding | GC06M134169 | 8.01 |
| LCT | Lactase | Protein Coding | GC02M135787 | 8.01 |
| AEBP1 | AE Binding Protein 1 | Protein Coding | GC07P044106 | 8.01 |
| RIPPLY2 | Ripply Transcriptional Repressor 2 | Protein Coding | GC06P083854 | 8.01 |
| HTR2C | 5-Hydroxytryptamine Receptor 2C | Protein Coding | GC0XP114584 | 8.01 |
| TOP1 | DNA Topoisomerase I | Protein Coding | GC20P041028 | 7.98 |
| CNTF | Ciliary Neurotrophic Factor | Protein Coding | GC11P058622 | 7.97 |
| UCN | Urocortin | Protein Coding | GC02M027308 | 7.96 |
| NGFR | Nerve Growth Factor Receptor | Protein Coding | GC17P049495 | 7.95 |
| KCNN2 | Potassium Calcium-Activated Channel Subfamily N Member 2 | Protein Coding | GC05P114058 | 7.95 |
| CHRDL1 | Chordin Like 1 | Protein Coding | GC0XM110674 | 7.95 |
| GRIK1 | Glutamate Ionotropic Receptor Kainate Type Subunit 1 | Protein Coding | GC21M029536 | 7.95 |
| SYK | Spleen Associated Tyrosine Kinase | Protein Coding | GC09P091171 | 7.95 |
| ASH1L | ASH1 Like Histone Lysine Methyltransferase | Protein Coding | GC01M155335 | 7.94 |
| MIR134 | MicroRNA 134 | RNA Gene | GC14P104777 | 7.93 |
| SLC19A1 | Solute Carrier Family 19 Member 1 | Protein Coding | GC21M045493 | 7.93 |
| PLXND1 | Plexin D1 | Protein Coding | GC03M129555 | 7.93 |
| RNASEH1 | Ribonuclease H1 | Protein Coding | GC02M003544 | 7.93 |
| TNFRSF8 | TNF Receptor Superfamily Member 8 | Protein Coding | GC01P012063 | 7.91 |
| OSM | Oncostatin M | Protein Coding | GC22M030262 | 7.91 |
| CHRM1 | Cholinergic Receptor Muscarinic 1 | Protein Coding | GC11M063457 | 7.91 |
| ARSJ | Arylsulfatase Family Member J | Protein Coding | GC04M113901 | 7.91 |
| LAMB1 | Laminin Subunit Beta 1 | Protein Coding | GC07M107923 | 7.9 |
| GRIA1 | Glutamate Ionotropic Receptor AMPA Type Subunit 1 | Protein Coding | GC05P153467 | 7.9 |
| BCL11B | BAF Chromatin Remodeling Complex Subunit BCL11B | Protein Coding | GC14M099169 | 7.89 |
| LIAS | Lipoic Acid Synthetase | Protein Coding | GC04P039461 | 7.89 |
| FGF12 | Fibroblast Growth Factor 12 | Protein Coding | GC03M192139 | 7.89 |
| PPP1R17 | Protein Phosphatase 1 Regulatory Subunit 17 | Protein Coding | GC07P031726 | 7.89 |
| BPI | Bactericidal Permeability Increasing Protein | Protein Coding | GC20P038304 | 7.87 |
| AQP1 | Aquaporin 1 (Colton Blood Group) | Protein Coding | GC07P030911 | 7.87 |
| MCPH1 | Microcephalin 1 | Protein Coding | GC08P006406 | 7.87 |
| HECTD4 | HECT Domain E3 Ubiquitin Protein Ligase 4 | Protein Coding | GC12M112160 | 7.86 |
| CELSR2 | Cadherin EGF LAG Seven-Pass G-Type Receptor 2 | Protein Coding | GC01P109250 | 7.86 |
| H2AX | H2A.X Variant Histone | Protein Coding | GC11M119097 | 7.86 |
| POLD1 | DNA Polymerase Delta 1, Catalytic Subunit | Protein Coding | GC19P050385 | 7.86 |
| CILP | Cartilage Intermediate Layer Protein | Protein Coding | GC15M065194 | 7.85 |
| H6PD | Hexose-6-Phosphate Dehydrogenase/Glucose 1-Dehydrogenase | Protein Coding | GC01P009234 | 7.85 |
| TNFRSF4 | TNF Receptor Superfamily Member 4 | Protein Coding | GC01M001211 | 7.84 |
| IL37 | Interleukin 37 | Protein Coding | GC02P115304 | 7.84 |
| KIF20A | Kinesin Family Member 20A | Protein Coding | GC05P138189 | 7.84 |
| A2M | Alpha-2-Macroglobulin | Protein Coding | GC12M009067 | 7.83 |
| ABCA7 | ATP Binding Cassette Subfamily A Member 7 | Protein Coding | GC19P001040 | 7.83 |
| MIR328 | MicroRNA 328 | RNA Gene | GC16M067203 | 7.82 |
| STXBP2 | Syntaxin Binding Protein 2 | Protein Coding | GC19P007637 | 7.81 |
| PTPA | Protein Phosphatase 2 Phosphatase Activator | Protein Coding | GC09P129111 | 7.81 |
| CYP1A2 | Cytochrome P450 Family 1 Subfamily A Member 2 | Protein Coding | GC15P074748 | 7.8 |
| GHSR | Growth Hormone Secretagogue Receptor | Protein Coding | GC03M172443 | 7.79 |
| PRPF31 | Pre-MRNA Processing Factor 31 | Protein Coding | GC19P055363 | 7.79 |
| RNF113A | Ring Finger Protein 113A | Protein Coding | GC0XM119870 | 7.79 |
| BIRC5 | Baculoviral IAP Repeat Containing 5 | Protein Coding | GC17P078214 | 7.78 |
| GFER | Growth Factor, Augmenter Of Liver Regeneration | Protein Coding | GC16P001984 | 7.78 |
| MYO6 | Myosin VI | Protein Coding | GC06P075749 | 7.78 |
| CAPN5 | Calpain 5 | Protein Coding | GC11P077066 | 7.78 |
| CALR3 | Calreticulin 3 | Protein Coding | GC19M016450 | 7.78 |
| PHF6 | PHD Finger Protein 6 | Protein Coding | GC0XP134373 | 7.78 |
| UBC | Ubiquitin C | Protein Coding | GC12M124911 | 7.75 |
| VRK1 | VRK Serine/Threonine Kinase 1 | Protein Coding | GC14P096797 | 7.75 |
| COQ10A | Coenzyme Q10A | Protein Coding | GC12P056266 | 7.75 |
| GLP1R | Glucagon Like Peptide 1 Receptor | Protein Coding | GC06P039048 | 7.74 |
| TP53COR1 | Tumor Protein P53 Pathway Corepressor 1 | RNA Gene | GC06U903133 | 7.74 |
| BNC2 | Basonuclin 2 | Protein Coding | GC09M016410 | 7.73 |
| CFHR2 | Complement Factor H Related 2 | Protein Coding | GC01P196943 | 7.73 |
| ITGAX | Integrin Subunit Alpha X | Protein Coding | GC16P031570 | 7.73 |
| ITGA5 | Integrin Subunit Alpha 5 | Protein Coding | GC12M054396 | 7.73 |
| FDXR | Ferredoxin Reductase | Protein Coding | GC17M074862 | 7.73 |
| MYO9A | Myosin IXA | Protein Coding | GC15M071822 | 7.73 |
| AAT1 | Aortic Aneurysm, Familial Thoracic 1 | Genetic Locus | GC11U900016 | 7.73 |
| AK1 | Adenylate Kinase 1 | Protein Coding | GC09M127866 | 7.72 |
| TARID | TCF21 Antisense RNA Inducing Promoter Demethylation | RNA Gene | GC06M133502 | 7.72 |
| VEGFB | Vascular Endothelial Growth Factor B | Protein Coding | GC11P064234 | 7.71 |
| MIRLET7G | MicroRNA Let-7g | RNA Gene | GC03M052268 | 7.71 |
| TBC1D7 | TBC1 Domain Family Member 7 | Protein Coding | GC06M013266 | 7.7 |
| FBLN2 | Fibulin 2 | Protein Coding | GC03P013565 | 7.7 |
| MACROD2 | Mono-ADP Ribosylhydrolase 2 | Protein Coding | GC20P013925 | 7.69 |
| IL18R1 | Interleukin 18 Receptor 1 | Protein Coding | GC02P102311 | 7.68 |
| YWHAB | Tyrosine 3-Monooxygenase/Tryptophan 5-Monooxygenase Activation Protein Beta | Protein Coding | GC20P044885 | 7.67 |
| ITGB1BP2 | Integrin Subunit Beta 1 Binding Protein 2 | Protein Coding | GC0XP071302 | 7.67 |
| MTAP | Methylthioadenosine Phosphorylase | Protein Coding | GC09P021792 | 7.66 |
| DCC | DCC Netrin 1 Receptor | Protein Coding | GC18P052340 | 7.66 |
| NEUROG3 | Neurogenin 3 | Protein Coding | GC10M069571 | 7.66 |
| ALOX12 | Arachidonate 12-Lipoxygenase, 12S Type | Protein Coding | GC17P006995 | 7.65 |
| CCL18 | C-C Motif Chemokine Ligand 18 | Protein Coding | GC17P036064 | 7.64 |
| ZNF335 | Zinc Finger Protein 335 | Protein Coding | GC20M045948 | 7.64 |
| DAB2 | DAB Adaptor Protein 2 | Protein Coding | GC05M039371 | 7.63 |
| HYAL1 | Hyaluronidase 1 | Protein Coding | GC03M050299 | 7.63 |
| ZNHIT3 | Zinc Finger HIT-Type Containing 3 | Protein Coding | GC17P036486 | 7.63 |
| SERPINA7 | Serpin Family A Member 7 | Protein Coding | GC0XM106032 | 7.63 |
| PRDX1 | Peroxiredoxin 1 | Protein Coding | GC01M045511 | 7.62 |
| ARHGEF2 | Rho/Rac Guanine Nucleotide Exchange Factor 2 | Protein Coding | GC01M155946 | 7.62 |
| NSUN6 | NOP2/Sun RNA Methyltransferase 6 | Protein Coding | GC10M018520 | 7.61 |
| NISCH | Nischarin | Protein Coding | GC03P052455 | 7.61 |
| PRMT7 | Protein Arginine Methyltransferase 7 | Protein Coding | GC16P068339 | 7.61 |
| CCL21 | C-C Motif Chemokine Ligand 21 | Protein Coding | GC09M034709 | 7.6 |
| KDSR | 3-Ketodihydrosphingosine Reductase | Protein Coding | GC18M063327 | 7.6 |
| FGF21 | Fibroblast Growth Factor 21 | Protein Coding | GC19P048766 | 7.59 |
| MIR100 | MicroRNA 100 | RNA Gene | GC11M122152 | 7.55 |
| TCF12 | Transcription Factor 12 | Protein Coding | GC15P056918 | 7.55 |
| CEP85L | Centrosomal Protein 85 Like | Protein Coding | GC06M118460 | 7.55 |
| NADSYN1 | NAD Synthetase 1 | Protein Coding | GC11P071454 | 7.54 |
| IL23A | Interleukin 23 Subunit Alpha | Protein Coding | GC12P056335 | 7.54 |
| YAP1 | Yes1 Associated Transcriptional Regulator | Protein Coding | GC11P102110 | 7.54 |
| ROBO2 | Roundabout Guidance Receptor 2 | Protein Coding | GC03P075955 | 7.54 |
| ERN1 | Endoplasmic Reticulum To Nucleus Signaling 1 | Protein Coding | GC17M064039 | 7.54 |
| FOLR1 | Folate Receptor Alpha | Protein Coding | GC11P072190 | 7.53 |
| CYLD | CYLD Lysine 63 Deubiquitinase | Protein Coding | GC16P050742 | 7.53 |
| LYZ | Lysozyme | Protein Coding | GC12P069348 | 7.52 |
| SMARCA1 | SWI/SNF Related, Matrix Associated, Actin Dependent Regulator Of Chromatin, Subfamily A, Member 1 | Protein Coding | GC0XM129447 | 7.52 |
| DLG4 | Discs Large MAGUK Scaffold Protein 4 | Protein Coding | GC17M007189 | 7.51 |
| RHOH | Ras Homolog Family Member H | Protein Coding | GC04P040192 | 7.51 |
| MIR125B1 | MicroRNA 125b-1 | RNA Gene | GC11M122100 | 7.5 |
| SOAT2 | Sterol O-Acyltransferase 2 | Protein Coding | GC12P053103 | 7.5 |
| IGF2BP2 | Insulin Like Growth Factor 2 MRNA Binding Protein 2 | Protein Coding | GC03M185643 | 7.5 |
| HAPLN1 | Hyaluronan And Proteoglycan Link Protein 1 | Protein Coding | GC05M083637 | 7.5 |
| HLA-DRA | Major Histocompatibility Complex, Class II, DR Alpha | Protein Coding | GC06P032439 | 7.5 |
| TUBA4A | Tubulin Alpha 4a | Protein Coding | GC02M219249 | 7.5 |
| FUCA2 | Alpha-L-Fucosidase 2 | Protein Coding | GC06M143494 | 7.5 |
| APOD | Apolipoprotein D | Protein Coding | GC03M195568 | 7.48 |
| APOA1-AS | APOA1 Antisense RNA | RNA Gene | GC11P116836 | 7.48 |
| CDK1 | Cyclin Dependent Kinase 1 | Protein Coding | GC10P060772 | 7.47 |
| DCPS | Decapping Enzyme, Scavenger | Protein Coding | GC11P126303 | 7.47 |
| SLC25A5 | Solute Carrier Family 25 Member 5 | Protein Coding | GC0XP119468 | 7.47 |
| GLRB | Glycine Receptor Beta | Protein Coding | GC04P157076 | 7.47 |
| RNF213-AS1 | RNF213 Antisense RNA 1 | RNA Gene | GC17M080335 | 7.47 |
| ADCY1 | Adenylate Cyclase 1 | Protein Coding | GC07P045580 | 7.47 |
| FBXO7 | F-Box Protein 7 | Protein Coding | GC22P032474 | 7.46 |
| MIR106A | MicroRNA 106a | RNA Gene | GC0XM134219 | 7.46 |
| lnc-KDM5D-4 |  | RNA Gene | GC0YM020519 | 7.46 |
| RAB5A | RAB5A, Member RAS Oncogene Family | Protein Coding | GC03P019963 | 7.45 |
| MCU | Mitochondrial Calcium Uniporter | Protein Coding | GC10P072692 | 7.44 |
| PTGIR | Prostaglandin I2 Receptor | Protein Coding | GC19M046611 | 7.44 |
| OGA | O-GlcNAcase | Protein Coding | GC10M101785 | 7.44 |
| KCNT2 | Potassium Sodium-Activated Channel Subfamily T Member 2 | Protein Coding | GC01M196225 | 7.44 |
| FST | Follistatin | Protein Coding | GC05P053480 | 7.43 |
| STAG1 | Stromal Antigen 1 | Protein Coding | GC03M136336 | 7.42 |
| DYM | Dymeclin | Protein Coding | GC18M049041 | 7.42 |
| RUNX1T1 | RUNX1 Partner Transcriptional Co-Repressor 1 | Protein Coding | GC08M091954 | 7.41 |
| HMGA1 | High Mobility Group AT-Hook 1 | Protein Coding | GC06P047417 | 7.41 |
| SRP72 | Signal Recognition Particle 72 | Protein Coding | GC04P056466 | 7.4 |
| FAM230H | Family With Sequence Similarity 230 Member H | RNA Gene | GC22M021316 | 7.4 |
| ANXA1 | Annexin A1 | Protein Coding | GC09P073151 | 7.4 |
| GHRH | Growth Hormone Releasing Hormone | Protein Coding | GC20M037251 | 7.4 |
| ANTXR1 | ANTXR Cell Adhesion Molecule 1 | Protein Coding | GC02P068977 | 7.39 |
| DAPK1 | Death Associated Protein Kinase 1 | Protein Coding | GC09P087497 | 7.39 |
| PNMT | Phenylethanolamine N-Methyltransferase | Protein Coding | GC17P039667 | 7.39 |
| SLC4A5 | Solute Carrier Family 4 Member 5 | Protein Coding | GC02M074217 | 7.38 |
| RPS15A | Ribosomal Protein S15a | Protein Coding | GC16M018781 | 7.38 |
| TSLP | Thymic Stromal Lymphopoietin | Protein Coding | GC05P111070 | 7.38 |
| C5AR1 | Complement C5a Receptor 1 | Protein Coding | GC19P047290 | 7.37 |
| CDK2 | Cyclin Dependent Kinase 2 | Protein Coding | GC12P055966 | 7.37 |
| MICOS13 | Mitochondrial Contact Site And Cristae Organizing System Subunit 13 | Protein Coding | GC19M005688 | 7.37 |
| NFKBIL1 | NFKB Inhibitor Like 1 | Protein Coding | GC06P047302 | 7.36 |
| DDAH1 | Dimethylarginine Dimethylaminohydrolase 1 | Protein Coding | GC01M085318 | 7.36 |
| TRNT1 | TRNA Nucleotidyl Transferase 1 | Protein Coding | GC03P003126 | 7.36 |
| AQP5 | Aquaporin 5 | Protein Coding | GC12P049961 | 7.35 |
| BID | BH3 Interacting Domain Death Agonist | Protein Coding | GC22M017734 | 7.35 |
| KCND2 | Potassium Voltage-Gated Channel Subfamily D Member 2 | Protein Coding | GC07P120273 | 7.35 |
| CCR4 | C-C Motif Chemokine Receptor 4 | Protein Coding | GC03P032951 | 7.34 |
| ATP2B1 | ATPase Plasma Membrane Ca2+ Transporting 1 | Protein Coding | GC12M089588 | 7.34 |
| SLC20A1 | Solute Carrier Family 20 Member 1 | Protein Coding | GC02P115301 | 7.34 |
| RRAGC | Ras Related GTP Binding C | Protein Coding | GC01M038844 | 7.34 |
| ADCY8 | Adenylate Cyclase 8 | Protein Coding | GC08M130780 | 7.34 |
| ANKRD26 | Ankyrin Repeat Domain 26 | Protein Coding | GC10M026938 | 7.33 |
| UGCG | UDP-Glucose Ceramide Glucosyltransferase | Protein Coding | GC09P111896 | 7.33 |
| GABRB1 | Gamma-Aminobutyric Acid Type A Receptor Subunit Beta1 | Protein Coding | GC04P046949 | 7.32 |
| CHD3 | Chromodomain Helicase DNA Binding Protein 3 | Protein Coding | GC17P008060 | 7.31 |
| FOXP2 | Forkhead Box P2 | Protein Coding | GC07P114086 | 7.3 |
| RAD50 | RAD50 Double Strand Break Repair Protein | Protein Coding | GC05P132556 | 7.3 |
| NTN1 | Netrin 1 | Protein Coding | GC17P009021 | 7.3 |
| IGHM | Immunoglobulin Heavy Constant Mu | Protein Coding | GC14M109532 | 7.29 |
| OXA1L | OXA1L Mitochondrial Inner Membrane Protein | Protein Coding | GC14P022766 | 7.29 |
| TMEM126A | Transmembrane Protein 126A | Protein Coding | GC11P085647 | 7.29 |
| HDLCQ1 | High Density Lipoprotein Cholesterol Level QTL 1 | Genetic Locus | GC09U900262 | 7.28 |
| ITGA9 | Integrin Subunit Alpha 9 | Protein Coding | GC03P037468 | 7.28 |
| LOC113939944 | Sharpr-MPRA Regulatory Region 9539 | Biological Region | GC15P048520 | 7.27 |
| ANXA2 | Annexin A2 | Protein Coding | GC15M060347 | 7.27 |
| CRLF2 | Cytokine Receptor Like Factor 2 | Protein Coding | GC0XM001190 | 7.27 |
| ZPR1 | ZPR1 Zinc Finger | Protein Coding | GC11M116777 | 7.26 |
| ROS1 | ROS Proto-Oncogene 1, Receptor Tyrosine Kinase | Protein Coding | GC06M117287 | 7.26 |
| HEYL | Hes Related Family BHLH Transcription Factor With YRPW Motif Like | Protein Coding | GC01M039623 | 7.26 |
| RAN | RAN, Member RAS Oncogene Family | Protein Coding | GC12P130871 | 7.25 |
| NAB2 | NGFI-A Binding Protein 2 | Protein Coding | GC12P057088 | 7.24 |
| MCAM | Melanoma Cell Adhesion Molecule | Protein Coding | GC11M119308 | 7.23 |
| KCNN3 | Potassium Calcium-Activated Channel Subfamily N Member 3 | Protein Coding | GC01M154697 | 7.23 |
| MIR199B | MicroRNA 199b | RNA Gene | GC09M128244 | 7.22 |
| TRPM1 | Transient Receptor Potential Cation Channel Subfamily M Member 1 | Protein Coding | GC15M031001 | 7.22 |
| CYP2C8 | Cytochrome P450 Family 2 Subfamily C Member 8 | Protein Coding | GC10M095038 | 7.21 |
| APEX1 | Apurinic/Apyrimidinic Endodeoxyribonuclease 1 | Protein Coding | GC14P020455 | 7.21 |
| NOD1 | Nucleotide Binding Oligomerization Domain Containing 1 | Protein Coding | GC07M030424 | 7.2 |
| SLCO1B1 | Solute Carrier Organic Anion Transporter Family Member 1B1 | Protein Coding | GC12P021132 | 7.19 |
| KAT2B | Lysine Acetyltransferase 2B | Protein Coding | GC03P020081 | 7.19 |
| GRHL2 | Grainyhead Like Transcription Factor 2 | Protein Coding | GC08P101492 | 7.19 |
| TAPT1 | Transmembrane Anterior Posterior Transformation 1 | Protein Coding | GC04M016162 | 7.18 |
| CABIN1 | Calcineurin Binding Protein 1 | Protein Coding | GC22P024011 | 7.18 |
| PTK2 | Protein Tyrosine Kinase 2 | Protein Coding | GC08M140657 | 7.18 |
| MIR26A1 | MicroRNA 26a-1 | RNA Gene | GC03P037969 | 7.17 |
| CXCL16 | C-X-C Motif Chemokine Ligand 16 | Protein Coding | GC17M004733 | 7.16 |
| CRYGC | Crystallin Gamma C | Protein Coding | GC02M208128 | 7.16 |
| FCN2 | Ficolin 2 | Protein Coding | GC09P134864 | 7.16 |
| MRPL3 | Mitochondrial Ribosomal Protein L3 | Protein Coding | GC03M131462 | 7.15 |
| RGS2 | Regulator Of G Protein Signaling 2 | Protein Coding | GC01P192809 | 7.15 |
| FMN2 | Formin 2 | Protein Coding | GC01P240014 | 7.14 |
| PPIA | Peptidylprolyl Isomerase A | Protein Coding | GC07P044807 | 7.14 |
| HHEX | Hematopoietically Expressed Homeobox | Protein Coding | GC10P092689 | 7.14 |
| HDAC2 | Histone Deacetylase 2 | Protein Coding | GC06M113933 | 7.14 |
| CLOCK | Clock Circadian Regulator | Protein Coding | GC04M055427 | 7.14 |
| LIPF | Lipase F, Gastric Type | Protein Coding | GC10P088664 | 7.14 |
| CLDN4 | Claudin 4 | Protein Coding | GC07P073799 | 7.13 |
| HBS1L | HBS1 Like Translational GTPase | Protein Coding | GC06M134960 | 7.13 |
| NONO | Non-POU Domain Containing Octamer Binding | Protein Coding | GC0XP071283 | 7.13 |
| RYR3 | Ryanodine Receptor 3 | Protein Coding | GC15P033310 | 7.13 |
| CLCN6 | Chloride Voltage-Gated Channel 6 | Protein Coding | GC01P011806 | 7.13 |
| HPSE | Heparanase | Protein Coding | GC04M083292 | 7.13 |
| SIRT6 | Sirtuin 6 | Protein Coding | GC19M004174 | 7.12 |
| TACR1 | Tachykinin Receptor 1 | Protein Coding | GC02M075010 | 7.12 |
| SMTN | Smoothelin | Protein Coding | GC22P031081 | 7.11 |
| TRPV2 | Transient Receptor Potential Cation Channel Subfamily V Member 2 | Protein Coding | GC17P016415 | 7.1 |
| TRH | Thyrotropin Releasing Hormone | Protein Coding | GC03P129974 | 7.1 |
| CRHR1 | Corticotropin Releasing Hormone Receptor 1 | Protein Coding | GC17P045784 | 7.1 |
| TREM1 | Triggering Receptor Expressed On Myeloid Cells 1 | Protein Coding | GC06M041267 | 7.1 |
| SRR | Serine Racemase | Protein Coding | GC17P002303 | 7.09 |
| HIP1 | Huntingtin Interacting Protein 1 | Protein Coding | GC07M075533 | 7.08 |
| PRKAB1 | Protein Kinase AMP-Activated Non-Catalytic Subunit Beta 1 | Protein Coding | GC12P119632 | 7.08 |
| CAMK2A | Calcium/Calmodulin Dependent Protein Kinase II Alpha | Protein Coding | GC05M150219 | 7.08 |
| FDPS | Farnesyl Diphosphate Synthase | Protein Coding | GC01P155308 | 7.07 |
| FKBP6 | FKBP Prolyl Isomerase 6 | Protein Coding | GC07P073328 | 7.07 |
| KDM6B | Lysine Demethylase 6B | Protein Coding | GC17P007834 | 7.07 |
| NPB | Neuropeptide B | Protein Coding | GC17P081900 | 7.06 |
| PARS2 | Prolyl-TRNA Synthetase 2, Mitochondrial | Protein Coding | GC01M054756 | 7.06 |
| CXCL11 | C-X-C Motif Chemokine Ligand 11 | Protein Coding | GC04M076033 | 7.06 |
| MYOG | Myogenin | Protein Coding | GC01M203083 | 7.06 |
| LIMS1 | LIM Zinc Finger Domain Containing 1 | Protein Coding | GC02P108609 | 7.06 |
| DUOX1 | Dual Oxidase 1 | Protein Coding | GC15P045129 | 7.06 |
| AP2B1 | Adaptor Related Protein Complex 2 Subunit Beta 1 | Protein Coding | GC17P035578 | 7.06 |
| CNTN1 | Contactin 1 | Protein Coding | GC12P040692 | 7.05 |
| KDM5B | Lysine Demethylase 5B | Protein Coding | GC01M202696 | 7.05 |
| SLC22A2 | Solute Carrier Family 22 Member 2 | Protein Coding | GC06M160173 | 7.05 |
| IL5RA | Interleukin 5 Receptor Subunit Alpha | Protein Coding | GC03M003066 | 7.04 |
| VPS11 | VPS11 Core Subunit Of CORVET And HOPS Complexes | Protein Coding | GC11P119067 | 7.04 |
| ADPRH | ADP-Ribosylarginine Hydrolase | Protein Coding | GC03P119579 | 7.03 |
| MAPRE2 | Microtubule Associated Protein RP/EB Family Member 2 | Protein Coding | GC18P034976 | 7.02 |
| FEN1 | Flap Structure-Specific Endonuclease 1 | Protein Coding | GC11P061811 | 7.02 |
| GAP43 | Growth Associated Protein 43 | Protein Coding | GC03P115623 | 7.01 |
| HLA-DQA2 | Major Histocompatibility Complex, Class II, DQ Alpha 2 | Protein Coding | GC06P032741 | 7.01 |
| ABCG4 | ATP Binding Cassette Subfamily G Member 4 | Protein Coding | GC11P119150 | 7.01 |
| DLC1 | DLC1 Rho GTPase Activating Protein | Protein Coding | GC08M013083 | 7 |
| DUSP6 | Dual Specificity Phosphatase 6 | Protein Coding | GC12M089347 | 7 |
| KAT2A | Lysine Acetyltransferase 2A | Protein Coding | GC17M042113 | 6.99 |
| UTRN | Utrophin | Protein Coding | GC06P144285 | 6.99 |
| SMDT1 | Single-Pass Membrane Protein With Aspartate Rich Tail 1 | Protein Coding | GC22P042079 | 6.97 |
| BCL2L11 | BCL2 Like 11 | Protein Coding | GC02P111119 | 6.97 |
| SLC2A3 | Solute Carrier Family 2 Member 3 | Protein Coding | GC12M007919 | 6.97 |
| FABP1 | Fatty Acid Binding Protein 1 | Protein Coding | GC02M088122 | 6.96 |
| MBNL1 | Muscleblind Like Splicing Regulator 1 | Protein Coding | GC03P152243 | 6.95 |
| CXCL5 | C-X-C Motif Chemokine Ligand 5 | Protein Coding | GC04M073995 | 6.95 |
| TYMS | Thymidylate Synthetase | Protein Coding | GC18P000657 | 6.94 |
| FBXO38 | F-Box Protein 38 | Protein Coding | GC05P148383 | 6.94 |
| TRB | T Cell Receptor Beta Locus | Protein Coding | GC07P145138 | 6.94 |
| FKBP5 | FKBP Prolyl Isomerase 5 | Protein Coding | GC06M042217 | 6.93 |
| GAS6 | Growth Arrest Specific 6 | Protein Coding | GC13M113820 | 6.92 |
| ACTA2-AS1 | ACTA2 Antisense RNA 1 | RNA Gene | GC10P088932 | 6.92 |
| SMARCA5 | SWI/SNF Related, Matrix Associated, Actin Dependent Regulator Of Chromatin, Subfamily A, Member 5 | Protein Coding | GC04P143513 | 6.92 |
| MIR149 | MicroRNA 149 | RNA Gene | GC02P240456 | 6.92 |
| SLC5A1 | Solute Carrier Family 5 Member 1 | Protein Coding | GC22P032043 | 6.92 |
| GPC1 | Glypican 1 | Protein Coding | GC02P240435 | 6.92 |
| SAA4 | Serum Amyloid A4, Constitutive | Protein Coding | GC11M018234 | 6.92 |
| RPS3A | Ribosomal Protein S3A | Protein Coding | GC04P151099 | 6.91 |
| SOX6 | SRY-Box Transcription Factor 6 | Protein Coding | GC11M015949 | 6.91 |
| CACNG5 | Calcium Voltage-Gated Channel Auxiliary Subunit Gamma 5 | Protein Coding | GC17P066835 | 6.91 |
| PTPN3 | Protein Tyrosine Phosphatase Non-Receptor Type 3 | Protein Coding | GC09M109375 | 6.9 |
| NEAT1 | Nuclear Paraspeckle Assembly Transcript 1 | RNA Gene | GC11P065794 | 6.9 |
| INO80 | INO80 Complex ATPase Subunit | Protein Coding | GC15M040979 | 6.9 |
| CAMP | Cathelicidin Antimicrobial Peptide | Protein Coding | GC03P048266 | 6.9 |
| SETDB1 | SET Domain Bifurcated Histone Lysine Methyltransferase 1 | Protein Coding | GC01P150926 | 6.89 |
| CXCL13 | C-X-C Motif Chemokine Ligand 13 | Protein Coding | GC04P077511 | 6.89 |
| CCDC92 | Coiled-Coil Domain Containing 92 | Protein Coding | GC12M123918 | 6.88 |
| GSS | Glutathione Synthetase | Protein Coding | GC20M034928 | 6.88 |
| MARK3 | Microtubule Affinity Regulating Kinase 3 | Protein Coding | GC14P103385 | 6.87 |
| OGG1 | 8-Oxoguanine DNA Glycosylase | Protein Coding | GC03P009751 | 6.87 |
| SNAI1 | Snail Family Transcriptional Repressor 1 | Protein Coding | GC20P049982 | 6.86 |
| GPD2 | Glycerol-3-Phosphate Dehydrogenase 2 | Protein Coding | GC02P156435 | 6.86 |
| MIR590 | MicroRNA 590 | RNA Gene | GC07P074191 | 6.85 |
| HDAC1 | Histone Deacetylase 1 | Protein Coding | GC01P032292 | 6.85 |
| BAG6 | BAG Cochaperone 6 | Protein Coding | GC06M031639 | 6.84 |
| LRP4 | LDL Receptor Related Protein 4 | Protein Coding | GC11M061111 | 6.84 |
| MRPS34 | Mitochondrial Ribosomal Protein S34 | Protein Coding | GC16M001771 | 6.84 |
| MYBPHL | Myosin Binding Protein H Like | Protein Coding | GC01M109292 | 6.84 |
| MKI67 | Marker Of Proliferation Ki-67 | Protein Coding | GC10M128096 | 6.84 |
| FGF5 | Fibroblast Growth Factor 5 | Protein Coding | GC04P080266 | 6.83 |
| SH3BP2 | SH3 Domain Binding Protein 2 | Protein Coding | GC04P002794 | 6.83 |
| HIC1 | HIC ZBTB Transcriptional Repressor 1 | Protein Coding | GC17P002054 | 6.82 |
| PRDM8 | PR/SET Domain 8 | Protein Coding | GC04P080183 | 6.81 |
| DNAH10 | Dynein Axonemal Heavy Chain 10 | Protein Coding | GC12P123762 | 6.81 |
| SRD5A1 | Steroid 5 Alpha-Reductase 1 | Protein Coding | GC05P006633 | 6.81 |
| GGT2 | Gamma-Glutamyltransferase 2 | Protein Coding | GC22M021207 | 6.81 |
| LOC106029312 | Williams-Beuren Syndrome Medial Block B Recombination Region | Biological Region | GC07P074733 | 6.81 |
| MIR16-1 | MicroRNA 16-1 | RNA Gene | GC13M050048 | 6.8 |
| ANGPTL4 | Angiopoietin Like 4 | Protein Coding | GC19P008363 | 6.8 |
| CLDN3 | Claudin 3 | Protein Coding | GC07M073768 | 6.8 |
| ATRIP | ATR Interacting Protein | Protein Coding | GC03P048449 | 6.8 |
| MACROD1 | Mono-ADP Ribosylhydrolase 1 | Protein Coding | GC11M063998 | 6.78 |
| MIR92A1 | MicroRNA 92a-1 | RNA Gene | GC13P091431 | 6.77 |
| TNNC2 | Troponin C2, Fast Skeletal Type | Protein Coding | GC20M045823 | 6.77 |
| ESRRB | Estrogen Related Receptor Beta | Protein Coding | GC14P076310 | 6.77 |
| FGF7 | Fibroblast Growth Factor 7 | Protein Coding | GC15P049423 | 6.77 |
| TRIM2 | Tripartite Motif Containing 2 | Protein Coding | GC04P153152 | 6.76 |
| ARHGEF9 | Cdc42 Guanine Nucleotide Exchange Factor 9 | Protein Coding | GC0XM063634 | 6.76 |
| WDR4 | WD Repeat Domain 4 | Protein Coding | GC21M042843 | 6.75 |
| TJP1 | Tight Junction Protein 1 | Protein Coding | GC15M029699 | 6.75 |
| SERPINB1 | Serpin Family B Member 1 | Protein Coding | GC06M002833 | 6.75 |
| NPS | Neuropeptide S | Protein Coding | GC10P127549 | 6.74 |
| MIRLET7C | MicroRNA Let-7c | RNA Gene | GC21P016551 | 6.74 |
| SRRT | Serrate, RNA Effector Molecule | Protein Coding | GC07P100875 | 6.74 |
| NUDT6 | Nudix Hydrolase 6 | Protein Coding | GC04M122888 | 6.73 |
| UBAP1 | Ubiquitin Associated Protein 1 | Protein Coding | GC09P034179 | 6.73 |
| NT5C2 | 5'-Nucleotidase, Cytosolic II | Protein Coding | GC10M103088 | 6.72 |
| TNFRSF10A | TNF Receptor Superfamily Member 10a | Protein Coding | GC08M023190 | 6.71 |
| ST3GAL4 | ST3 Beta-Galactoside Alpha-2,3-Sialyltransferase 4 | Protein Coding | GC11P126355 | 6.7 |
| RAB8A | RAB8A, Member RAS Oncogene Family | Protein Coding | GC19P016111 | 6.7 |
| FMOD | Fibromodulin | Protein Coding | GC01M203340 | 6.69 |
| CACNA1E | Calcium Voltage-Gated Channel Subunit Alpha1 E | Protein Coding | GC01P181317 | 6.69 |
| HSPB2 | Heat Shock Protein Family B (Small) Member 2 | Protein Coding | GC11P111913 | 6.69 |
| KLRK1 | Killer Cell Lectin Like Receptor K1 | Protein Coding | GC12M013865 | 6.68 |
| MMAB | Metabolism Of Cobalamin Associated B | Protein Coding | GC12M109553 | 6.68 |
| NSUN5 | NOP2/Sun RNA Methyltransferase 5 | Protein Coding | GC07M073302 | 6.67 |
| B4GALT1 | Beta-1,4-Galactosyltransferase 1 | Protein Coding | GC09M033100 | 6.67 |
| PKM | Pyruvate Kinase M1/2 | Protein Coding | GC15M072199 | 6.65 |
| SMS | Spermine Synthase | Protein Coding | GC0XP021958 | 6.65 |
| PI4KA | Phosphatidylinositol 4-Kinase Alpha | Protein Coding | GC22M020707 | 6.65 |
| FOLH1 | Folate Hydrolase 1 | Protein Coding | GC11M061160 | 6.64 |
| CACNB3 | Calcium Voltage-Gated Channel Auxiliary Subunit Beta 3 | Protein Coding | GC12P048813 | 6.64 |
| MLPH | Melanophilin | Protein Coding | GC02P237485 | 6.63 |
| SIRT2 | Sirtuin 2 | Protein Coding | GC19M038878 | 6.63 |
| TAF1 | TATA-Box Binding Protein Associated Factor 1 | Protein Coding | GC0XP071366 | 6.63 |
| DDR2 | Discoidin Domain Receptor Tyrosine Kinase 2 | Protein Coding | GC01P162631 | 6.62 |
| OPHN1 | Oligophrenin 1 | Protein Coding | GC0XM068042 | 6.62 |
| HAND2-AS1 | HAND2 Antisense RNA 1 | RNA Gene | GC04P173527 | 6.62 |
| ARHGEF10 | Rho Guanine Nucleotide Exchange Factor 10 | Protein Coding | GC08P001823 | 6.62 |
| SMAD1 | SMAD Family Member 1 | Protein Coding | GC04P145481 | 6.62 |
| OPCML | Opioid Binding Protein/Cell Adhesion Molecule Like | Protein Coding | GC11M132414 | 6.61 |
| PTPN2 | Protein Tyrosine Phosphatase Non-Receptor Type 2 | Protein Coding | GC18M017325 | 6.61 |
| HSP90B1 | Heat Shock Protein 90 Beta Family Member 1 | Protein Coding | GC12P103930 | 6.61 |
| EPHA3 | EPH Receptor A3 | Protein Coding | GC03P089077 | 6.6 |
| BCL7B | BAF Chromatin Remodeling Complex Subunit BCL7B | Protein Coding | GC07M073536 | 6.6 |
| PYY | Peptide YY | Protein Coding | GC17M043952 | 6.6 |
| MS4A2 | Membrane Spanning 4-Domains A2 | Protein Coding | GC11P060088 | 6.59 |
| GLRX | Glutaredoxin | Protein Coding | GC05M095752 | 6.59 |
| ADAMTS2 | ADAM Metallopeptidase With Thrombospondin Type 1 Motif 2 | Protein Coding | GC05M179110 | 6.59 |
| PSRC1 | Proline And Serine Rich Coiled-Coil 1 | Protein Coding | GC01M109279 | 6.59 |
| TRIO | Trio Rho Guanine Nucleotide Exchange Factor | Protein Coding | GC05P014143 | 6.59 |
| TXNDC15 | Thioredoxin Domain Containing 15 | Protein Coding | GC05P134873 | 6.58 |
| NOP2 | NOP2 Nucleolar Protein | Protein Coding | GC12M006556 | 6.58 |
| RGS9BP | Regulator Of G Protein Signaling 9 Binding Protein | Protein Coding | GC19P032675 | 6.58 |
| TAF6 | TATA-Box Binding Protein Associated Factor 6 | Protein Coding | GC07M100107 | 6.58 |
| KLF6 | Kruppel Like Factor 6 | Protein Coding | GC10M003779 | 6.57 |
| ICMT | Isoprenylcysteine Carboxyl Methyltransferase | Protein Coding | GC01M006222 | 6.57 |
| PITPNA | Phosphatidylinositol Transfer Protein Alpha | Protein Coding | GC17M001525 | 6.56 |
| TRAF1 | TNF Receptor Associated Factor 1 | Protein Coding | GC09M120902 | 6.55 |
| NAXD | NAD(P)HX Dehydratase | Protein Coding | GC13P110616 | 6.55 |
| KLK1 | Kallikrein 1 | Protein Coding | GC19M050819 | 6.55 |
| CACNG1 | Calcium Voltage-Gated Channel Auxiliary Subunit Gamma 1 | Protein Coding | GC17P067044 | 6.54 |
| SIK3 | SIK Family Kinase 3 | Protein Coding | GC11M116843 | 6.54 |
| MIR206 | MicroRNA 206 | RNA Gene | GC06P052144 | 6.54 |
| CTSS | Cathepsin S | Protein Coding | GC01M150730 | 6.54 |
| ADIRF | Adipogenesis Regulatory Factor | Protein Coding | GC10P086968 | 6.54 |
| MCM6 | Minichromosome Maintenance Complex Component 6 | Protein Coding | GC02M135839 | 6.53 |
| POC1B | POC1 Centriolar Protein B | Protein Coding | GC12M089419 | 6.53 |
| FOXD4 | Forkhead Box D4 | Protein Coding | GC09M000116 | 6.53 |
| CRYGD | Crystallin Gamma D | Protein Coding | GC02M208121 | 6.52 |
| ACACA | Acetyl-CoA Carboxylase Alpha | Protein Coding | GC17M037084 | 6.52 |
| CAVIN4 | Caveolae Associated Protein 4 | Protein Coding | GC09P100576 | 6.51 |
| ADAM8 | ADAM Metallopeptidase Domain 8 | Protein Coding | GC10M133262 | 6.51 |
| PLCB1 | Phospholipase C Beta 1 | Protein Coding | GC20P008061 | 6.51 |
| KANK2 | KN Motif And Ankyrin Repeat Domains 2 | Protein Coding | GC19M011165 | 6.51 |
| LCK | LCK Proto-Oncogene, Src Family Tyrosine Kinase | Protein Coding | GC01P032251 | 6.51 |
| TTF2 | Transcription Termination Factor 2 | Protein Coding | GC01P117060 | 6.49 |
| HYOU1 | Hypoxia Up-Regulated 1 | Protein Coding | GC11M119045 | 6.49 |
| CSK | C-Terminal Src Kinase | Protein Coding | GC15P074782 | 6.49 |
| NR1I2 | Nuclear Receptor Subfamily 1 Group I Member 2 | Protein Coding | GC03P119780 | 6.48 |
| PDPN | Podoplanin | Protein Coding | GC01P013583 | 6.48 |
| PEMT | Phosphatidylethanolamine N-Methyltransferase | Protein Coding | GC17M017506 | 6.48 |
| EDC3 | Enhancer Of MRNA Decapping 3 | Protein Coding | GC15M074631 | 6.47 |
| ALG14 | ALG14 UDP-N-Acetylglucosaminyltransferase Subunit | Protein Coding | GC01M094974 | 6.47 |
| BARD1 | BRCA1 Associated RING Domain 1 | Protein Coding | GC02M214725 | 6.47 |
| CAP2 | Cyclase Associated Actin Cytoskeleton Regulatory Protein 2 | Protein Coding | GC06P017393 | 6.47 |
| EIF4E | Eukaryotic Translation Initiation Factor 4E | Protein Coding | GC04M098871 | 6.47 |
| PDHB | Pyruvate Dehydrogenase E1 Subunit Beta | Protein Coding | GC03M058428 | 6.47 |
| ELAVL1 | ELAV Like RNA Binding Protein 1 | Protein Coding | GC19M007958 | 6.45 |
| MIR27B | MicroRNA 27b | RNA Gene | GC09P095097 | 6.45 |
| MIR20B | MicroRNA 20b | RNA Gene | GC0XM134217 | 6.45 |
| ADCYAP1 | Adenylate Cyclase Activating Polypeptide 1 | Protein Coding | GC18P000895 | 6.45 |
| FGG | Fibrinogen Gamma Chain | Protein Coding | GC04M154604 | 6.45 |
| TBC1D4 | TBC1 Domain Family Member 4 | Protein Coding | GC13M075284 | 6.44 |
| SLC29A1 | Solute Carrier Family 29 Member 1 (Augustine Blood Group) | Protein Coding | GC06P044219 | 6.43 |
| NEDD4 | NEDD4 E3 Ubiquitin Protein Ligase | Protein Coding | GC15M055826 | 6.43 |
| CCL19 | C-C Motif Chemokine Ligand 19 | Protein Coding | GC09M034692 | 6.41 |
| HHIP | Hedgehog Interacting Protein | Protein Coding | GC04P144645 | 6.41 |
| LYVE1 | Lymphatic Vessel Endothelial Hyaluronan Receptor 1 | Protein Coding | GC11M010653 | 6.41 |
| FCGR1A | Fc Fragment Of IgG Receptor Ia | Protein Coding | GC01P149754 | 6.4 |
| MAPK9 | Mitogen-Activated Protein Kinase 9 | Protein Coding | GC05M180234 | 6.4 |
| DRP2 | Dystrophin Related Protein 2 | Protein Coding | GC0XP101219 | 6.4 |
| AOC1 | Amine Oxidase Copper Containing 1 | Protein Coding | GC07P150824 | 6.39 |
| IMMT | Inner Membrane Mitochondrial Protein | Protein Coding | GC02M086144 | 6.39 |
| GNAL | G Protein Subunit Alpha L | Protein Coding | GC18P011689 | 6.39 |
| RPS6 | Ribosomal Protein S6 | Protein Coding | GC09M019375 | 6.38 |
| LNPEP | Leucyl And Cystinyl Aminopeptidase | Protein Coding | GC05P096935 | 6.38 |
| PRKCG | Protein Kinase C Gamma | Protein Coding | GC19P053879 | 6.37 |
| SLN | Sarcolipin | Protein Coding | GC11M107709 | 6.36 |
| EIF2S1 | Eukaryotic Translation Initiation Factor 2 Subunit Alpha | Protein Coding | GC14P067359 | 6.34 |
| CDKAL1 | CDK5 Regulatory Subunit Associated Protein 1 Like 1 | Protein Coding | GC06P020534 | 6.34 |
| PXK | PX Domain Containing Serine/Threonine Kinase Like | Protein Coding | GC03P058333 | 6.34 |
| THSD4 | Thrombospondin Type 1 Domain Containing 4 | Protein Coding | GC15P071096 | 6.34 |
| FABP12 | Fatty Acid Binding Protein 12 | Protein Coding | GC08M081524 | 6.33 |
| CYSLTR1 | Cysteinyl Leukotriene Receptor 1 | Protein Coding | GC0XM078271 | 6.32 |
| ACAT1 | Acetyl-CoA Acetyltransferase 1 | Protein Coding | GC11P108121 | 6.31 |
| GPC5 | Glypican 5 | Protein Coding | GC13P091398 | 6.31 |
| ARMS2 | Age-Related Maculopathy Susceptibility 2 | Protein Coding | GC10P122454 | 6.3 |
| MIR148B | MicroRNA 148b | RNA Gene | GC12P054337 | 6.3 |
| CCND3 | Cyclin D3 | Protein Coding | GC06M041934 | 6.29 |
| SLC1A4 | Solute Carrier Family 1 Member 4 | Protein Coding | GC02P064988 | 6.28 |
| CCN1 | Cellular Communication Network Factor 1 | Protein Coding | GC01P085581 | 6.27 |
| TPR | Translocated Promoter Region, Nuclear Basket Protein | Protein Coding | GC01M186319 | 6.27 |
| GUCY1B1 | Guanylate Cyclase 1 Soluble Subunit Beta 1 | Protein Coding | GC04P155759 | 6.27 |
| HPS6 | HPS6 Biogenesis Of Lysosomal Organelles Complex 2 Subunit 3 | Protein Coding | GC10P102065 | 6.26 |
| KLHDC8B | Kelch Domain Containing 8B | Protein Coding | GC03P049171 | 6.26 |
| ANPEP | Alanyl Aminopeptidase, Membrane | Protein Coding | GC15M089784 | 6.25 |
| MIR139 | MicroRNA 139 | RNA Gene | GC11M072615 | 6.25 |
| ESRRA | Estrogen Related Receptor Alpha | Protein Coding | GC11P064305 | 6.25 |
| CD1C | CD1c Molecule | Protein Coding | GC01P158289 | 6.25 |
| GRM5 | Glutamate Metabotropic Receptor 5 | Protein Coding | GC11M088504 | 6.23 |
| FBLN1 | Fibulin 1 | Protein Coding | GC22P045502 | 6.23 |
| CNR2 | Cannabinoid Receptor 2 | Protein Coding | GC01M023870 | 6.23 |
| DNAH6 | Dynein Axonemal Heavy Chain 6 | Protein Coding | GC02P084459 | 6.23 |
| FDFT1 | Farnesyl-Diphosphate Farnesyltransferase 1 | Protein Coding | GC08P011795 | 6.22 |
| DGAT2 | Diacylglycerol O-Acyltransferase 2 | Protein Coding | GC11P075759 | 6.22 |
| ADAMTS6 | ADAM Metallopeptidase With Thrombospondin Type 1 Motif 6 | Protein Coding | GC05M065148 | 6.21 |
| MYO15A | Myosin XVA | Protein Coding | GC17P018108 | 6.21 |
| PSMD5 | Proteasome 26S Subunit, Non-ATPase 5 | Protein Coding | GC09M120815 | 6.21 |
| PLA2G5 | Phospholipase A2 Group V | Protein Coding | GC01P020028 | 6.21 |
| RAPGEF2 | Rap Guanine Nucleotide Exchange Factor 2 | Protein Coding | GC04P159106 | 6.19 |
| LGALS1 | Galectin 1 | Protein Coding | GC22P037675 | 6.19 |
| CFD | Complement Factor D | Protein Coding | GC19P000859 | 6.18 |
| DPP9 | Dipeptidyl Peptidase 9 | Protein Coding | GC19M004675 | 6.18 |
| PCK2 | Phosphoenolpyruvate Carboxykinase 2, Mitochondrial | Protein Coding | GC14P024094 | 6.18 |
| AASS | Aminoadipate-Semialdehyde Synthase | Protein Coding | GC07M122073 | 6.18 |
| REG1A | Regenerating Family Member 1 Alpha | Protein Coding | GC02P079120 | 6.18 |
| VPS37D | VPS37D Subunit Of ESCRT-I | Protein Coding | GC07P073666 | 6.17 |
| PTK2B | Protein Tyrosine Kinase 2 Beta | Protein Coding | GC08P027311 | 6.16 |
| PTGER4 | Prostaglandin E Receptor 4 | Protein Coding | GC05P040679 | 6.15 |
| MIR181A2 | MicroRNA 181a-2 | RNA Gene | GC09P124692 | 6.15 |
| FCRL3 | Fc Receptor Like 3 | Protein Coding | GC01M157674 | 6.15 |
| SEC24D | SEC24 Homolog D, COPII Coat Complex Component | Protein Coding | GC04M118722 | 6.14 |
| ASPN | Asporin | Protein Coding | GC09M092458 | 6.13 |
| SLC1A1 | Solute Carrier Family 1 Member 1 | Protein Coding | GC09P004490 | 6.13 |
| CD63 | CD63 Molecule | Protein Coding | GC12M055725 | 6.12 |
| MFGE8 | Milk Fat Globule EGF And Factor V/VIII Domain Containing | Protein Coding | GC15M088898 | 6.11 |
| F2RL1 | F2R Like Trypsin Receptor 1 | Protein Coding | GC05P076818 | 6.11 |
| ZBTB17 | Zinc Finger And BTB Domain Containing 17 | Protein Coding | GC01M015943 | 6.1 |
| GATAD2B | GATA Zinc Finger Domain Containing 2B | Protein Coding | GC01M153805 | 6.1 |
| CCL7 | C-C Motif Chemokine Ligand 7 | Protein Coding | GC17P034270 | 6.1 |
| KIRREL3 | Kirre Like Nephrin Family Adhesion Molecule 3 | Protein Coding | GC11M126423 | 6.09 |
| SEC23A | Sec23 Homolog A, COPII Coat Complex Component | Protein Coding | GC14M039031 | 6.09 |
| CES1 | Carboxylesterase 1 | Protein Coding | GC16M055836 | 6.08 |
| H3-3B | H3.3 Histone B | Protein Coding | GC17M075780 | 6.08 |
| GTF2IRD2 | GTF2I Repeat Domain Containing 2 | Protein Coding | GC07M074796 | 6.07 |
| DUSP1 | Dual Specificity Phosphatase 1 | Protein Coding | GC05M172768 | 6.07 |
| GMPR | Guanosine Monophosphate Reductase | Protein Coding | GC06P016238 | 6.07 |
| ARHGAP26 | Rho GTPase Activating Protein 26 | Protein Coding | GC05P142770 | 6.07 |
| WDR81 | WD Repeat Domain 81 | Protein Coding | GC17P001716 | 6.06 |
| MIR32 | MicroRNA 32 | RNA Gene | GC09M109046 | 6.05 |
| YWHAZ | Tyrosine 3-Monooxygenase/Tryptophan 5-Monooxygenase Activation Protein Zeta | Protein Coding | GC08M100917 | 6.05 |
| NOX5 | NADPH Oxidase 5 | Protein Coding | GC15P072888 | 6.05 |
| MYO9B | Myosin IXB | Protein Coding | GC19P023294 | 6.04 |
| STK24 | Serine/Threonine Kinase 24 | Protein Coding | GC13M098445 | 6.03 |
| PIP5K1C | Phosphatidylinositol-4-Phosphate 5-Kinase Type 1 Gamma | Protein Coding | GC19M003631 | 6.02 |
| PLK4 | Polo Like Kinase 4 | Protein Coding | GC04P127880 | 6.02 |
| HAS2 | Hyaluronan Synthase 2 | Protein Coding | GC08M121594 | 6.01 |
| HRH1 | Histamine Receptor H1 | Protein Coding | GC03P011113 | 6.01 |
| SIRT5 | Sirtuin 5 | Protein Coding | GC06P013574 | 6 |
| PI3 | Peptidase Inhibitor 3 | Protein Coding | GC20P045174 | 6 |
| DVL2 | Dishevelled Segment Polarity Protein 2 | Protein Coding | GC17M007225 | 6 |
| MIR532 | MicroRNA 532 | RNA Gene | GC0XP050004 | 6 |
| MIR181C | MicroRNA 181c | RNA Gene | GC19P013876 | 6 |
| CD70 | CD70 Molecule | Protein Coding | GC19M006583 | 5.99 |
| APOC4 | Apolipoprotein C4 | Protein Coding | GC19P044943 | 5.99 |
| CLEC16A | C-Type Lectin Domain Containing 16A | Protein Coding | GC16P010944 | 5.99 |
| LIN9 | Lin-9 DREAM MuvB Core Complex Component | Protein Coding | GC01M226231 | 5.99 |
| MADD | MAP Kinase Activating Death Domain | Protein Coding | GC11P047290 | 5.99 |
| CNN1 | Calponin 1 | Protein Coding | GC19P011539 | 5.98 |
| MYB | MYB Proto-Oncogene, Transcription Factor | Protein Coding | GC06P135180 | 5.97 |
| SLC10A1 | Solute Carrier Family 10 Member 1 | Protein Coding | GC14M069775 | 5.97 |
| AAGAB | Alpha And Gamma Adaptin Binding Protein | Protein Coding | GC15M067200 | 5.97 |
| TNS1 | Tensin 1 | Protein Coding | GC02M217799 | 5.97 |
| POLR2A | RNA Polymerase II Subunit A | Protein Coding | GC17P008025 | 5.96 |
| DBN1 | Drebrin 1 | Protein Coding | GC05M177456 | 5.95 |
| MIR375 | MicroRNA 375 | RNA Gene | GC02M219001 | 5.95 |
| SPTBN4 | Spectrin Beta, Non-Erythrocytic 4 | Protein Coding | GC19P040466 | 5.95 |
| CLIP1 | CAP-Gly Domain Containing Linker Protein 1 | Protein Coding | GC12M122271 | 5.95 |
| POLB | DNA Polymerase Beta | Protein Coding | GC08P042338 | 5.94 |
| PBRM1 | Polybromo 1 | Protein Coding | GC03M052545 | 5.94 |
| TRPC4AP | Transient Receptor Potential Cation Channel Subfamily C Member 4 Associated Protein | Protein Coding | GC20M035002 | 5.94 |
| LPIN1 | Lipin 1 | Protein Coding | GC02P011649 | 5.92 |
| APEX2 | Apurinic/Apyrimidinic Endodeoxyribonuclease 2 | Protein Coding | GC0XP055000 | 5.91 |
| SIRT4 | Sirtuin 4 | Protein Coding | GC12P120291 | 5.91 |
| EIF4H | Eukaryotic Translation Initiation Factor 4H | Protein Coding | GC07P074174 | 5.91 |
| ATP6V1G2-DDX39B | ATP6V1G2-DDX39B Readthrough (NMD Candidate) | RNA Gene | GC06M032588 | 5.9 |
| MTNR1B | Melatonin Receptor 1B | Protein Coding | GC11P092969 | 5.9 |
| EGLN3 | Egl-9 Family Hypoxia Inducible Factor 3 | Protein Coding | GC14M033924 | 5.9 |
| PRG2 | Proteoglycan 2, Pro Eosinophil Major Basic Protein | Protein Coding | GC11M057386 | 5.89 |
| FZD9 | Frizzled Class Receptor 9 | Protein Coding | GC07P073433 | 5.88 |
| BMPER | BMP Binding Endothelial Regulator | Protein Coding | GC07P033944 | 5.88 |
| MNT | MAX Network Transcriptional Repressor | Protein Coding | GC17M002384 | 5.87 |
| KCNAB1 | Potassium Voltage-Gated Channel Subfamily A Member Regulatory Beta Subunit 1 | Protein Coding | GC03P156037 | 5.87 |
| NR1I3 | Nuclear Receptor Subfamily 1 Group I Member 3 | Protein Coding | GC01M161229 | 5.87 |
| PNPLA5 | Patatin Like Phospholipase Domain Containing 5 | Protein Coding | GC22M043879 | 5.87 |
| PDE4B | Phosphodiesterase 4B | Protein Coding | GC01P065792 | 5.87 |
| EMILIN1 | Elastin Microfibril Interfacer 1 | Protein Coding | GC02P027078 | 5.87 |
| DOCK7 | Dedicator Of Cytokinesis 7 | Protein Coding | GC01M062454 | 5.86 |
| CCNA2 | Cyclin A2 | Protein Coding | GC04M121816 | 5.86 |
| LEXM | Lymphocyte Expansion Molecule | Protein Coding | GC01P054807 | 5.85 |
| HBE1 | Hemoglobin Subunit Epsilon 1 | Protein Coding | GC11M005268 | 5.85 |
| CSN1S1 | Casein Alpha S1 | Protein Coding | GC04P069932 | 5.85 |
| PLXNA2 | Plexin A2 | Protein Coding | GC01M208023 | 5.85 |
| RPL7 | Ribosomal Protein L7 | Protein Coding | GC08M073290 | 5.84 |
| FOXL1 | Forkhead Box L1 | Protein Coding | GC16P086576 | 5.83 |
| ADD2 | Adducin 2 | Protein Coding | GC02M070626 | 5.83 |
| KEAP1 | Kelch Like ECH Associated Protein 1 | Protein Coding | GC19M010486 | 5.83 |
| LOC110973015 | NOS3 5' Regulatory Region | Biological Region | GC07P150988 | 5.83 |
| HOXC-AS1 | HOXC Cluster Antisense RNA 1 | RNA Gene | GC12M053999 | 5.82 |
| ST8SIA4 | ST8 Alpha-N-Acetyl-Neuraminide Alpha-2,8-Sialyltransferase 4 | Protein Coding | GC05M100806 | 5.82 |
| OXLD1 | Oxidoreductase Like Domain Containing 1 | Protein Coding | GC17M081665 | 5.8 |
| POU2F3 | POU Class 2 Homeobox 3 | Protein Coding | GC11P120236 | 5.8 |
| PER1 | Period Circadian Regulator 1 | Protein Coding | GC17M008853 | 5.78 |
| ENPEP | Glutamyl Aminopeptidase | Protein Coding | GC04P110365 | 5.77 |
| FAM20A | FAM20A Golgi Associated Secretory Pathway Pseudokinase | Protein Coding | GC17M068535 | 5.77 |
| ADM2 | Adrenomedullin 2 | Protein Coding | GC22P050481 | 5.77 |
| HYPLIP2 | Hyperlipidemia, Combined, 2 | Genetic Locus | GC11U990241 | 5.77 |
| CACNG7 | Calcium Voltage-Gated Channel Auxiliary Subunit Gamma 7 | Protein Coding | GC19P053909 | 5.77 |
| RASD1 | Ras Related Dexamethasone Induced 1 | Protein Coding | GC17M017494 | 5.77 |
| CYBC1 | Cytochrome B-245 Chaperone 1 | Protein Coding | GC17M082443 | 5.76 |
| CYBRD1 | Cytochrome B Reductase 1 | Protein Coding | GC02P171522 | 5.76 |
| CECR7 | Cat Eye Syndrome Chromosome Region, Candidate 7 | RNA Gene | GC22P017072 | 5.76 |
| TRAF3 | TNF Receptor Associated Factor 3 | Protein Coding | GC14P104639 | 5.76 |
| RPLP0 | Ribosomal Protein Lateral Stalk Subunit P0 | Protein Coding | GC12M120196 | 5.76 |
| CLDN5 | Claudin 5 | Protein Coding | GC22M019523 | 5.76 |
| RXRG | Retinoid X Receptor Gamma | Protein Coding | GC01M165401 | 5.75 |
| UNC5C | Unc-5 Netrin Receptor C | Protein Coding | GC04M095162 | 5.75 |
| MTMR3 | Myotubularin Related Protein 3 | Protein Coding | GC22P029885 | 5.75 |
| NFIB | Nuclear Factor I B | Protein Coding | GC09M014077 | 5.74 |
| SHMT1 | Serine Hydroxymethyltransferase 1 | Protein Coding | GC17M019857 | 5.74 |
| FASN | Fatty Acid Synthase | Protein Coding | GC17M082078 | 5.74 |
| TBXAS1 | Thromboxane A Synthase 1 | Protein Coding | GC07P139777 | 5.74 |
| CAD | Carbamoyl-Phosphate Synthetase 2, Aspartate Transcarbamylase, And Dihydroorotase | Protein Coding | GC02P027217 | 5.73 |
| RAMP2 | Receptor Activity Modifying Protein 2 | Protein Coding | GC17P042758 | 5.73 |
| UBE2D1 | Ubiquitin Conjugating Enzyme E2 D1 | Protein Coding | GC10P058334 | 5.72 |
| IL19 | Interleukin 19 | Protein Coding | GC01P206770 | 5.7 |
| PRELP | Proline And Arginine Rich End Leucine Rich Repeat Protein | Protein Coding | GC01P203444 | 5.7 |
| VPS13C | Vacuolar Protein Sorting 13 Homolog C | Protein Coding | GC15M061852 | 5.7 |
| VASP | Vasodilator Stimulated Phosphoprotein | Protein Coding | GC19P045507 | 5.7 |
| OGN | Osteoglycin | Protein Coding | GC09M092383 | 5.69 |
| MFAP4 | Microfibril Associated Protein 4 | Protein Coding | GC17M019383 | 5.68 |
| CACNG8 | Calcium Voltage-Gated Channel Auxiliary Subunit Gamma 8 | Protein Coding | GC19P054637 | 5.68 |
| CHGB | Chromogranin B | Protein Coding | GC20P005911 | 5.68 |
| SH3PXD2A | SH3 And PX Domains 2A | Protein Coding | GC10M103594 | 5.68 |
| DYNLL1 | Dynein Light Chain LC8-Type 1 | Protein Coding | GC12P120469 | 5.68 |
| PLCB4 | Phospholipase C Beta 4 | Protein Coding | GC20P009024 | 5.68 |
| NOMO3 | NODAL Modulator 3 | Protein Coding | GC16P016232 | 5.67 |
| CA2 | Carbonic Anhydrase 2 | Protein Coding | GC08P085463 | 5.67 |
| IQGAP1 | IQ Motif Containing GTPase Activating Protein 1 | Protein Coding | GC15P090388 | 5.66 |
| PRDM1 | PR/SET Domain 1 | Protein Coding | GC06P105993 | 5.66 |
| SCRN3 | Secernin 3 | Protein Coding | GC02P174395 | 5.66 |
| MSN | Moesin | Protein Coding | GC0XP065588 | 5.65 |
| GOT2 | Glutamic-Oxaloacetic Transaminase 2 | Protein Coding | GC16M058707 | 5.64 |
| PIK3CB | Phosphatidylinositol-4,5-Bisphosphate 3-Kinase Catalytic Subunit Beta | Protein Coding | GC03M138652 | 5.64 |
| IL36RN | Interleukin 36 Receptor Antagonist | Protein Coding | GC02P115306 | 5.63 |
| P2RX4 | Purinergic Receptor P2X 4 | Protein Coding | GC12P122831 | 5.61 |
| CYP26B1 | Cytochrome P450 Family 26 Subfamily B Member 1 | Protein Coding | GC02M072129 | 5.61 |
| TRIM63 | Tripartite Motif Containing 63 | Protein Coding | GC01M026062 | 5.6 |
| EML1 | EMAP Like 1 | Protein Coding | GC14P099737 | 5.59 |
| SBF2-AS1 | SBF2 Antisense RNA 1 | RNA Gene | GC11P009758 | 5.59 |
| S100A4 | S100 Calcium Binding Protein A4 | Protein Coding | GC01M153543 | 5.59 |
| SUMO4 | Small Ubiquitin Like Modifier 4 | Protein Coding | GC06P149401 | 5.59 |
| ATP5IF1 | ATP Synthase Inhibitory Factor Subunit 1 | Protein Coding | GC01P028237 | 5.58 |
| PRKCZ | Protein Kinase C Zeta | Protein Coding | GC01P002050 | 5.57 |
| TMEM116 | Transmembrane Protein 116 | Protein Coding | GC12M111931 | 5.57 |
| KLK15 | Kallikrein Related Peptidase 15 | Protein Coding | GC19M050825 | 5.57 |
| HPS5 | HPS5 Biogenesis Of Lysosomal Organelles Complex 2 Subunit 2 | Protein Coding | GC11M018278 | 5.56 |
| SNRPA | Small Nuclear Ribonucleoprotein Polypeptide A | Protein Coding | GC19P040750 | 5.56 |
| ARHGEF1 | Rho Guanine Nucleotide Exchange Factor 1 | Protein Coding | GC19P041883 | 5.56 |
| RPS2 | Ribosomal Protein S2 | Protein Coding | GC16M002198 | 5.56 |
| CATSPER2 | Cation Channel Sperm Associated 2 | Protein Coding | GC15M043628 | 5.55 |
| WNT9B | Wnt Family Member 9B | Protein Coding | GC17P046833 | 5.54 |
| P2RX3 | Purinergic Receptor P2X 3 | Protein Coding | GC11P057356 | 5.54 |
| ID4 | Inhibitor Of DNA Binding 4, HLH Protein | Protein Coding | GC06P019837 | 5.54 |
| STK32B | Serine/Threonine Kinase 32B | Protein Coding | GC04P005053 | 5.54 |
| S1PR1 | Sphingosine-1-Phosphate Receptor 1 | Protein Coding | GC01P101236 | 5.54 |
| NR4A1 | Nuclear Receptor Subfamily 4 Group A Member 1 | Protein Coding | GC12P052022 | 5.53 |
| LAMA5 | Laminin Subunit Alpha 5 | Protein Coding | GC20M062307 | 5.53 |
| CCDC174 | Coiled-Coil Domain Containing 174 | Protein Coding | GC03P014655 | 5.53 |
| CNTLN | Centlein | Protein Coding | GC09P017124 | 5.52 |
| PRDX5 | Peroxiredoxin 5 | Protein Coding | GC11P064317 | 5.52 |
| ADAMTS20 | ADAM Metallopeptidase With Thrombospondin Type 1 Motif 20 | Protein Coding | GC12M043353 | 5.52 |
| CTNS | Cystinosin, Lysosomal Cystine Transporter | Protein Coding | GC17P003636 | 5.51 |
| TAF1A | TATA-Box Binding Protein Associated Factor, RNA Polymerase I Subunit A | Protein Coding | GC01M222557 | 5.51 |
| NFIA-AS1 | NFIA Antisense RNA 1 | RNA Gene | GC01M061248 | 5.51 |
| NOMO2 | NODAL Modulator 2 | Protein Coding | GC16M018512 | 5.5 |
| CCL22 | C-C Motif Chemokine Ligand 22 | Protein Coding | GC16P057359 | 5.5 |
| RCC1L | RCC1 Like | Protein Coding | GC07M075029 | 5.49 |
| HCN2 | Hyperpolarization Activated Cyclic Nucleotide Gated Potassium And Sodium Channel 2 | Protein Coding | GC19P000589 | 5.48 |
| TONSL | Tonsoku Like, DNA Repair Protein | Protein Coding | GC08M144428 | 5.48 |
| MDK | Midkine | Protein Coding | GC11P046402 | 5.47 |
| CLCN3 | Chloride Voltage-Gated Channel 3 | Protein Coding | GC04P169612 | 5.46 |
| AKR1A1 | Aldo-Keto Reductase Family 1 Member A1 | Protein Coding | GC01P045550 | 5.46 |
| CPT1B | Carnitine Palmitoyltransferase 1B | Protein Coding | GC22M050569 | 5.46 |
| HK2 | Hexokinase 2 | Protein Coding | GC02P074833 | 5.46 |
| MIR502 | MicroRNA 502 | RNA Gene | GC0XP050014 | 5.46 |
| LIPJ | Lipase Family Member J | Protein Coding | GC10P088583 | 5.46 |
| P2RY2 | Purinergic Receptor P2Y2 | Protein Coding | GC11P073217 | 5.45 |
| GIP | Gastric Inhibitory Polypeptide | Protein Coding | GC17M048958 | 5.45 |
| MLF1 | Myeloid Leukemia Factor 1 | Protein Coding | GC03P158571 | 5.45 |
| BCL3 | BCL3 Transcription Coactivator | Protein Coding | GC19P044747 | 5.44 |
| FGR | FGR Proto-Oncogene, Src Family Tyrosine Kinase | Protein Coding | GC01M027622 | 5.44 |
| RCE1 | Ras Converting CAAX Endopeptidase 1 | Protein Coding | GC11P066842 | 5.44 |
| NSUN3 | NOP2/Sun RNA Methyltransferase 3 | Protein Coding | GC03P094062 | 5.43 |
| BLVRA | Biliverdin Reductase A | Protein Coding | GC07P043758 | 5.43 |
| HERPUD1 | Homocysteine Inducible ER Protein With Ubiquitin Like Domain 1 | Protein Coding | GC16P056931 | 5.43 |
| PRKCQ | Protein Kinase C Theta | Protein Coding | GC10M006393 | 5.43 |
| VSTM4 | V-Set And Transmembrane Domain Containing 4 | Protein Coding | GC10M049014 | 5.43 |
| MIR505 | MicroRNA 505 | RNA Gene | GC0XM139924 | 5.42 |
| NOMO1 | NODAL Modulator 1 | Protein Coding | GC16P015131 | 5.41 |
| LPAR1 | Lysophosphatidic Acid Receptor 1 | Protein Coding | GC09M110873 | 5.41 |
| NUP153 | Nucleoporin 153 | Protein Coding | GC06M017615 | 5.4 |
| LIPM | Lipase Family Member M | Protein Coding | GC10P088804 | 5.4 |
| MIR1-1 | MicroRNA 1-1 | RNA Gene | GC20P062893 | 5.4 |
| ARNT | Aryl Hydrocarbon Receptor Nuclear Translocator | Protein Coding | GC01M150809 | 5.4 |
| ESM1 | Endothelial Cell Specific Molecule 1 | Protein Coding | GC05M054977 | 5.4 |
| CHRNA5 | Cholinergic Receptor Nicotinic Alpha 5 Subunit | Protein Coding | GC15P078565 | 5.4 |
| BLOC1S3 | Biogenesis Of Lysosomal Organelles Complex 1 Subunit 3 | Protein Coding | GC19P045178 | 5.39 |
| SLC5A3 | Solute Carrier Family 5 Member 3 | Protein Coding | GC21P034111 | 5.39 |
| CCL1 | C-C Motif Chemokine Ligand 1 | Protein Coding | GC17M034402 | 5.37 |
| RBP5 | Retinol Binding Protein 5 | Protein Coding | GC12M007125 | 5.37 |
| ULK1 | Unc-51 Like Autophagy Activating Kinase 1 | Protein Coding | GC12P131894 | 5.37 |
| IRX5 | Iroquois Homeobox 5 | Protein Coding | GC16P054930 | 5.37 |
| ADAMTS9 | ADAM Metallopeptidase With Thrombospondin Type 1 Motif 9 | Protein Coding | GC03M064501 | 5.36 |
| CPE | Carboxypeptidase E | Protein Coding | GC04P165361 | 5.36 |
| PTGER2 | Prostaglandin E Receptor 2 | Protein Coding | GC14P052314 | 5.36 |
| S1PR3 | Sphingosine-1-Phosphate Receptor 3 | Protein Coding | GC09P088991 | 5.36 |
| PDE3B | Phosphodiesterase 3B | Protein Coding | GC11P014643 | 5.36 |
| BTRC | Beta-Transducin Repeat Containing E3 Ubiquitin Protein Ligase | Protein Coding | GC10P101354 | 5.36 |
| GALNT2 | Polypeptide N-Acetylgalactosaminyltransferase 2 | Protein Coding | GC01P230057 | 5.35 |
| VEGFD | Vascular Endothelial Growth Factor D | Protein Coding | GC0XM015345 | 5.35 |
| DAB2IP | DAB2 Interacting Protein | Protein Coding | GC09P121566 | 5.35 |
| CRHR2 | Corticotropin Releasing Hormone Receptor 2 | Protein Coding | GC07M030651 | 5.35 |
| AREG | Amphiregulin | Protein Coding | GC04P074445 | 5.33 |
| IL27 | Interleukin 27 | Protein Coding | GC16M028511 | 5.33 |
| NCOR2 | Nuclear Receptor Corepressor 2 | Protein Coding | GC12M124324 | 5.33 |
| TNXA | Tenascin XA (Pseudogene) | Pseudogene | GC06M032638 | 5.33 |
| TIE1 | Tyrosine Kinase With Immunoglobulin Like And EGF Like Domains 1 | Protein Coding | GC01P043300 | 5.32 |
| RBBP4 | RB Binding Protein 4, Chromatin Remodeling Factor | Protein Coding | GC01P032651 | 5.32 |
| IRGM | Immunity Related GTPase M | Protein Coding | GC05P150846 | 5.32 |
| CYP4A11 | Cytochrome P450 Family 4 Subfamily A Member 11 | Protein Coding | GC01M046929 | 5.31 |
| SUV39H1 | Suppressor Of Variegation 3-9 Homolog 1 | Protein Coding | GC0XP048698 | 5.31 |
| TYRO3 | TYRO3 Protein Tyrosine Kinase | Protein Coding | GC15P041557 | 5.31 |
| CAPNS1 | Calpain Small Subunit 1 | Protein Coding | GC19P038254 | 5.31 |
| PLA2G10 | Phospholipase A2 Group X | Protein Coding | GC16M014672 | 5.31 |
| NAT10 | N-Acetyltransferase 10 | Protein Coding | GC11P034105 | 5.3 |
| NCOR1 | Nuclear Receptor Corepressor 1 | Protein Coding | GC17M016029 | 5.3 |
| SS18 | SS18 Subunit Of BAF Chromatin Remodeling Complex | Protein Coding | GC18M026016 | 5.29 |
| MIR28 | MicroRNA 28 | RNA Gene | GC03P188688 | 5.29 |
| SGMS2 | Sphingomyelin Synthase 2 | Protein Coding | GC04P107824 | 5.28 |
| INPP5B | Inositol Polyphosphate-5-Phosphatase B | Protein Coding | GC01M037860 | 5.28 |
| POLR2B | RNA Polymerase II Subunit B | Protein Coding | GC04P056977 | 5.27 |
| NME7 | NME/NM23 Family Member 7 | Protein Coding | GC01M169101 | 5.27 |
| RUNX3 | RUNX Family Transcription Factor 3 | Protein Coding | GC01M024899 | 5.27 |
| CBX5 | Chromobox 5 | Protein Coding | GC12M054230 | 5.26 |
| TNFSF13 | TNF Superfamily Member 13 | Protein Coding | GC17P007558 | 5.26 |
| ATG7 | Autophagy Related 7 | Protein Coding | GC03P011273 | 5.26 |
| TAB1 | TGF-Beta Activated Kinase 1 (MAP3K7) Binding Protein 1 | Protein Coding | GC22P039401 | 5.26 |
| RGS9 | Regulator Of G Protein Signaling 9 | Protein Coding | GC17P065137 | 5.25 |
| NFATC2 | Nuclear Factor Of Activated T Cells 2 | Protein Coding | GC20M051386 | 5.24 |
| CSMD1 | CUB And Sushi Multiple Domains 1 | Protein Coding | GC08M002953 | 5.24 |
| P4HA3 | Prolyl 4-Hydroxylase Subunit Alpha 3 | Protein Coding | GC11M074235 | 5.24 |
| APCS | Amyloid P Component, Serum | Protein Coding | GC01P159587 | 5.23 |
| TRIM50 | Tripartite Motif Containing 50 | Protein Coding | GC07M073312 | 5.23 |
| SLC22A1 | Solute Carrier Family 22 Member 1 | Protein Coding | GC06P160121 | 5.23 |
| MYLK-AS1 | MYLK Antisense RNA 1 | RNA Gene | GC03P123588 | 5.23 |
| RORA | RAR Related Orphan Receptor A | Protein Coding | GC15M060488 | 5.23 |
| ZCCHC8 | Zinc Finger CCHC-Type Containing 8 | Protein Coding | GC12M122472 | 5.23 |
| PSMC6 | Proteasome 26S Subunit, ATPase 6 | Protein Coding | GC14P052707 | 5.22 |
| KLF2 | Kruppel Like Factor 2 | Protein Coding | GC19P023268 | 5.22 |
| GALNT17 | Polypeptide N-Acetylgalactosaminyltransferase 17 | Protein Coding | GC07P071133 | 5.22 |
| MAP2K6 | Mitogen-Activated Protein Kinase Kinase 6 | Protein Coding | GC17P069414 | 5.21 |
| THSD1 | Thrombospondin Type 1 Domain Containing 1 | Protein Coding | GC13M052377 | 5.21 |
| UTS2R | Urotensin 2 Receptor | Protein Coding | GC17P082374 | 5.2 |
| WDR12 | WD Repeat Domain 12 | Protein Coding | GC02M202874 | 5.2 |
| PLPP3 | Phospholipid Phosphatase 3 | Protein Coding | GC01M056495 | 5.2 |
| ADORA2B | Adenosine A2b Receptor | Protein Coding | GC17P015927 | 5.19 |
| RPA1 | Replication Protein A1 | Protein Coding | GC17P001829 | 5.19 |
| SMG6 | SMG6 Nonsense Mediated MRNA Decay Factor | Protein Coding | GC17M002059 | 5.19 |
| LAT2 | Linker For Activation Of T Cells Family Member 2 | Protein Coding | GC07P074199 | 5.18 |
| HSPA1B | Heat Shock Protein Family A (Hsp70) Member 1B | Protein Coding | GC06P047325 | 5.18 |
| MIR138-1 | MicroRNA 138-1 | RNA Gene | GC03P044115 | 5.18 |
| LDB1 | LIM Domain Binding 1 | Protein Coding | GC10M102106 | 5.18 |
| METTL27 | Methyltransferase Like 27 | Protein Coding | GC07M073835 | 5.17 |
| LAMC1 | Laminin Subunit Gamma 1 | Protein Coding | GC01P182992 | 5.17 |
| LY96 | Lymphocyte Antigen 96 | Protein Coding | GC08P073991 | 5.17 |
| PRPS1L1 | Phosphoribosyl Pyrophosphate Synthetase 1 Like 1 | Protein Coding | GC07M018026 | 5.17 |
| CDH7 | Cadherin 7 | Protein Coding | GC18P065750 | 5.17 |
| MRC1 | Mannose Receptor C-Type 1 | Protein Coding | GC10P017809 | 5.17 |
| MIR19B1 | MicroRNA 19b-1 | RNA Gene | GC13P091435 | 5.16 |
| IL32 | Interleukin 32 | Protein Coding | GC16P004242 | 5.16 |
| WNT10B | Wnt Family Member 10B | Protein Coding | GC12M048965 | 5.16 |
| DDX39B | DExD-Box Helicase 39B | Protein Coding | GC06M031530 | 5.16 |
| ENSG00000247287 |  | RNA Gene | GC14M061298 | 5.15 |
| TRPC5 | Transient Receptor Potential Cation Channel Subfamily C Member 5 | Protein Coding | GC0XM111774 | 5.15 |
| CCDC154 | Coiled-Coil Domain Containing 154 | Protein Coding | GC16M001434 | 5.15 |
| PTCRA | Pre T Cell Antigen Receptor Alpha | Protein Coding | GC06P042915 | 5.15 |
| STAG3L4 | Stromal Antigen 3-Like 4 (Pseudogene) | Pseudogene | GC07P067302 | 5.14 |
| CFAP251 | Cilia And Flagella Associated Protein 251 | Protein Coding | GC12P122871 | 5.14 |
| ANO10 | Anoctamin 10 | Protein Coding | GC03M043396 | 5.14 |
| SRSF3 | Serine And Arginine Rich Splicing Factor 3 | Protein Coding | GC06P047457 | 5.14 |
| KAT5 | Lysine Acetyltransferase 5 | Protein Coding | GC11P065711 | 5.14 |
| ZNF513 | Zinc Finger Protein 513 | Protein Coding | GC02M027377 | 5.13 |
| MRPS6 | Mitochondrial Ribosomal Protein S6 | Protein Coding | GC21P034110 | 5.13 |
| ATG9B | Autophagy Related 9B | Protein Coding | GC07M151012 | 5.12 |
| ADAMTS5 | ADAM Metallopeptidase With Thrombospondin Type 1 Motif 5 | Protein Coding | GC21M026918 | 5.12 |
| OMD | Osteomodulin | Protein Coding | GC09M092414 | 5.12 |
| PPP3CB | Protein Phosphatase 3 Catalytic Subunit Beta | Protein Coding | GC10M073436 | 5.12 |
| IFIT3 | Interferon Induced Protein With Tetratricopeptide Repeats 3 | Protein Coding | GC10P089327 | 5.12 |
| C3AR1 | Complement C3a Receptor 1 | Protein Coding | GC12M008058 | 5.11 |
| CLTCL1 | Clathrin Heavy Chain Like 1 | Protein Coding | GC22M019171 | 5.11 |
| KCTD1 | Potassium Channel Tetramerization Domain Containing 1 | Protein Coding | GC18M026454 | 5.11 |
| ASZ1 | Ankyrin Repeat, SAM And Basic Leucine Zipper Domain Containing 1 | Protein Coding | GC07M117363 | 5.11 |
| MIR151A | MicroRNA 151a | RNA Gene | GC08M140733 | 5.11 |
| SERPINA12 | Serpin Family A Member 12 | Protein Coding | GC14M094526 | 5.1 |
| TNFRSF10C | TNF Receptor Superfamily Member 10c | Protein Coding | GC08P023102 | 5.1 |
| IGFBP5 | Insulin Like Growth Factor Binding Protein 5 | Protein Coding | GC02M216672 | 5.09 |
| TSR1 | TSR1 Ribosome Maturation Factor | Protein Coding | GC17M002322 | 5.09 |
| GRK4 | G Protein-Coupled Receptor Kinase 4 | Protein Coding | GC04P002963 | 5.08 |
| CRYBB2 | Crystallin Beta B2 | Protein Coding | GC22P025213 | 5.08 |
| NSUN7 | NOP2/Sun RNA Methyltransferase Family Member 7 | Protein Coding | GC04P040751 | 5.08 |
| BLZF1 | Basic Leucine Zipper Nuclear Factor 1 | Protein Coding | GC01P169367 | 5.08 |
| MXD4 | MAX Dimerization Protein 4 | Protein Coding | GC04M002295 | 5.07 |
| LPGAT1 | Lysophosphatidylglycerol Acyltransferase 1 | Protein Coding | GC01M211743 | 5.07 |
| ITGA11 | Integrin Subunit Alpha 11 | Protein Coding | GC15M068296 | 5.07 |
| LDB2 | LIM Domain Binding 2 | Protein Coding | GC04M016445 | 5.06 |
| ADAM12 | ADAM Metallopeptidase Domain 12 | Protein Coding | GC10M126012 | 5.06 |
| WASF2 | WASP Family Member 2 | Protein Coding | GC01M027404 | 5.06 |
| REV3L | REV3 Like, DNA Directed Polymerase Zeta Catalytic Subunit | Protein Coding | GC06M111299 | 5.05 |
| KCNK2 | Potassium Two Pore Domain Channel Subfamily K Member 2 | Protein Coding | GC01P215005 | 5.04 |
| FHL5 | Four And A Half LIM Domains 5 | Protein Coding | GC06P096562 | 5.04 |
| HIF1A-AS1 | HIF1A Antisense RNA 1 | RNA Gene | GC14M061681 | 5.04 |
| RXRB | Retinoid X Receptor Beta | Protein Coding | GC06M033193 | 5.03 |
| ICAM2 | Intercellular Adhesion Molecule 2 | Protein Coding | GC17M064002 | 5.03 |
| BAK1 | BCL2 Antagonist/Killer 1 | Protein Coding | GC06M033572 | 5.03 |
| GTPBP1 | GTP Binding Protein 1 | Protein Coding | GC22P038705 | 5.02 |
| WASF1 | WASP Family Member 1 | Protein Coding | GC06M110099 | 5.02 |
| CCNB1 | Cyclin B1 | Protein Coding | GC05P069167 | 5.02 |
| EFNB2 | Ephrin B2 | Protein Coding | GC13M106489 | 5.02 |
| TERF1 | Telomeric Repeat Binding Factor 1 | Protein Coding | GC08P073003 | 5.02 |
| CROCC | Ciliary Rootlet Coiled-Coil, Rootletin | Protein Coding | GC01P016798 | 5.02 |
| ABHD11 | Abhydrolase Domain Containing 11 | Protein Coding | GC07M073736 | 5.01 |
| C19orf33 | Chromosome 19 Open Reading Frame 33 | Protein Coding | GC19P038304 | 5.01 |
| ARHGAP29 | Rho GTPase Activating Protein 29 | Protein Coding | GC01M094148 | 5.01 |
| SRI | Sorcin | Protein Coding | GC07M088205 | 5.01 |
| UPF1 | UPF1 RNA Helicase And ATPase | Protein Coding | GC19P018831 | 5.01 |
| MIR152 | MicroRNA 152 | RNA Gene | GC17M048037 | 5.01 |
| HSPB3 | Heat Shock Protein Family B (Small) Member 3 | Protein Coding | GC05P054456 | 5.01 |
| SIRT7 | Sirtuin 7 | Protein Coding | GC17M081911 | 5 |
| PRICKLE2 | Prickle Planar Cell Polarity Protein 2 | Protein Coding | GC03M064079 | 4.99 |
| BUD13 | BUD13 Homolog | Protein Coding | GC11M116749 | 4.98 |
| PROZ | Protein Z, Vitamin K Dependent Plasma Glycoprotein | Protein Coding | GC13P113158 | 4.98 |
| MIR141 | MicroRNA 141 | RNA Gene | GC12P008231 | 4.98 |
| NECTIN2 | Nectin Cell Adhesion Molecule 2 | Protein Coding | GC19P044849 | 4.98 |
| GSTZ1 | Glutathione S-Transferase Zeta 1 | Protein Coding | GC14P077320 | 4.98 |
| ADPRS | ADP-Ribosylserine Hydrolase | Protein Coding | GC01P036089 | 4.97 |
| FZD7 | Frizzled Class Receptor 7 | Protein Coding | GC02P202034 | 4.97 |
| ROCK2 | Rho Associated Coiled-Coil Containing Protein Kinase 2 | Protein Coding | GC02M011192 | 4.97 |
| ASIC2 | Acid Sensing Ion Channel Subunit 2 | Protein Coding | GC17M033013 | 4.97 |
| NPTXR | Neuronal Pentraxin Receptor | Protein Coding | GC22M038818 | 4.96 |
| GSK3A | Glycogen Synthase Kinase 3 Alpha | Protein Coding | GC19M042230 | 4.96 |
| TIMD4 | T Cell Immunoglobulin And Mucin Domain Containing 4 | Protein Coding | GC05M156919 | 4.95 |
| HLA-E | Major Histocompatibility Complex, Class I, E | Protein Coding | GC06P047281 | 4.94 |
| MIA3 | MIA SH3 Domain ER Export Factor 3 | Protein Coding | GC01P222618 | 4.94 |
| SLC9A3R2 | SLC9A3 Regulator 2 | Protein Coding | GC16P002434 | 4.94 |
| CCN4 | Cellular Communication Network Factor 4 | Protein Coding | GC08P133192 | 4.93 |
| GLO1 | Glyoxalase I | Protein Coding | GC06M042239 | 4.93 |
| OXTR | Oxytocin Receptor | Protein Coding | GC03M008767 | 4.93 |
| LRPAP1 | LDL Receptor Related Protein Associated Protein 1 | Protein Coding | GC04M003508 | 4.93 |
| POU2F1 | POU Class 2 Homeobox 1 | Protein Coding | GC01P167190 | 4.93 |
| HACL1 | 2-Hydroxyacyl-CoA Lyase 1 | Protein Coding | GC03M015823 | 4.93 |
| CNP | 2',3'-Cyclic Nucleotide 3' Phosphodiesterase | Protein Coding | GC17P041966 | 4.93 |
| ACP6 | Acid Phosphatase 6, Lysophosphatidic | Protein Coding | GC01M147630 | 4.92 |
| NOL3 | Nucleolar Protein 3 | Protein Coding | GC16P067174 | 4.92 |
| AIM2 | Absent In Melanoma 2 | Protein Coding | GC01M159062 | 4.92 |
| SDC3 | Syndecan 3 | Protein Coding | GC01M030869 | 4.92 |
| SLC5A6 | Solute Carrier Family 5 Member 6 | Protein Coding | GC02M027201 | 4.92 |
| MYL1 | Myosin Light Chain 1 | Protein Coding | GC02M210290 | 4.91 |
| TNFRSF9 | TNF Receptor Superfamily Member 9 | Protein Coding | GC01M007915 | 4.91 |
| CES3 | Carboxylesterase 3 | Protein Coding | GC16P066963 | 4.9 |
| DAB1 | DAB Adaptor Protein 1 | Protein Coding | GC01M056994 | 4.9 |
| ETS2 | ETS Proto-Oncogene 2, Transcription Factor | Protein Coding | GC21P038805 | 4.89 |
| TRIM25 | Tripartite Motif Containing 25 | Protein Coding | GC17M056836 | 4.89 |
| RENBP | Renin Binding Protein | Protein Coding | GC0XM153935 | 4.89 |
| UBA7 | Ubiquitin Like Modifier Activating Enzyme 7 | Protein Coding | GC03M049805 | 4.88 |
| WNT2 | Wnt Family Member 2 | Protein Coding | GC07M117276 | 4.87 |
| PDGFD | Platelet Derived Growth Factor D | Protein Coding | GC11M103907 | 4.87 |
| GNAI1 | G Protein Subunit Alpha I1 | Protein Coding | GC07P079769 | 4.87 |
| SYMPK | Symplekin | Protein Coding | GC19M045815 | 4.87 |
| NIT2 | Nitrilase Family Member 2 | Protein Coding | GC03P100334 | 4.87 |
| FOXA2 | Forkhead Box A2 | Protein Coding | GC20M022581 | 4.86 |
| PANX1 | Pannexin 1 | Protein Coding | GC11P094128 | 4.86 |
| CAV2 | Caveolin 2 | Protein Coding | GC07P116287 | 4.86 |
| MYO1H | Myosin IH | Protein Coding | GC12P109347 | 4.86 |
| ORMDL3 | ORMDL Sphingolipid Biosynthesis Regulator 3 | Protein Coding | GC17M039921 | 4.85 |
| PRDX2 | Peroxiredoxin 2 | Protein Coding | GC19M012796 | 4.84 |
| PTK7 | Protein Tyrosine Kinase 7 (Inactive) | Protein Coding | GC06P043076 | 4.84 |
| ANO3 | Anoctamin 3 | Protein Coding | GC11P026188 | 4.83 |
| LGALS3BP | Galectin 3 Binding Protein | Protein Coding | GC17M078971 | 4.83 |
| DYNLT1 | Dynein Light Chain Tctex-Type 1 | Protein Coding | GC06M158636 | 4.83 |
| MED1 | Mediator Complex Subunit 1 | Protein Coding | GC17M039404 | 4.83 |
| SLC26A8 | Solute Carrier Family 26 Member 8 | Protein Coding | GC06M042225 | 4.83 |
| EXOSC10 | Exosome Component 10 | Protein Coding | GC01M011067 | 4.82 |
| MAP2K3 | Mitogen-Activated Protein Kinase Kinase 3 | Protein Coding | GC17P026749 | 4.82 |
| CD276 | CD276 Molecule | Protein Coding | GC15P073683 | 4.82 |
| CRABP2 | Cellular Retinoic Acid Binding Protein 2 | Protein Coding | GC01M156701 | 4.82 |
| SCX | Scleraxis BHLH Transcription Factor | Protein Coding | GC08P144265 | 4.81 |
| PRKAG1 | Protein Kinase AMP-Activated Non-Catalytic Subunit Gamma 1 | Protein Coding | GC12M049002 | 4.81 |
| ACVR2A | Activin A Receptor Type 2A | Protein Coding | GC02P147844 | 4.81 |
| PITRM1 | Pitrilysin Metallopeptidase 1 | Protein Coding | GC10M003138 | 4.81 |
| HDAC3 | Histone Deacetylase 3 | Protein Coding | GC05M141583 | 4.81 |
| SSTR2 | Somatostatin Receptor 2 | Protein Coding | GC17P073165 | 4.81 |
| CDK9 | Cyclin Dependent Kinase 9 | Protein Coding | GC09P127814 | 4.8 |
| MIR574 | MicroRNA 574 | RNA Gene | GC04P038872 | 4.8 |
| ABHD11-AS1 | ABHD11 Antisense RNA 1 (Tail To Tail) | RNA Gene | GC07P073735 | 4.79 |
| ADAM33 | ADAM Metallopeptidase Domain 33 | Protein Coding | GC20M003596 | 4.79 |
| FES | FES Proto-Oncogene, Tyrosine Kinase | Protein Coding | GC15P090883 | 4.79 |
| MMP10 | Matrix Metallopeptidase 10 | Protein Coding | GC11M102770 | 4.79 |
| ATP5PF | ATP Synthase Peripheral Stalk Subunit F6 | Protein Coding | GC21M025718 | 4.79 |
| HRG | Histidine Rich Glycoprotein | Protein Coding | GC03P186660 | 4.78 |
| CENPA | Centromere Protein A | Protein Coding | GC02P026750 | 4.78 |
| SULF1 | Sulfatase 1 | Protein Coding | GC08P069466 | 4.78 |
| NPNT | Nephronectin | Protein Coding | GC04P105894 | 4.78 |
| MIR376C | MicroRNA 376c | RNA Gene | GC14P104793 | 4.78 |
| MAPRE3 | Microtubule Associated Protein RP/EB Family Member 3 | Protein Coding | GC02P026935 | 4.77 |
| GRM7 | Glutamate Metabotropic Receptor 7 | Protein Coding | GC03P006770 | 4.76 |
| OPRK1 | Opioid Receptor Kappa 1 | Protein Coding | GC08M053227 | 4.76 |
| PHETA1 | PH Domain Containing Endocytic Trafficking Adaptor 1 | Protein Coding | GC12M111361 | 4.76 |
| APLP2 | Amyloid Beta Precursor Like Protein 2 | Protein Coding | GC11P130069 | 4.75 |
| DMBT1 | Deleted In Malignant Brain Tumors 1 | Protein Coding | GC10P122560 | 4.75 |
| AGTRAP | Angiotensin II Receptor Associated Protein | Protein Coding | GC01P011736 | 4.75 |
| CEP295 | Centrosomal Protein 295 | Protein Coding | GC11P093661 | 4.75 |
| PSD3 | Pleckstrin And Sec7 Domain Containing 3 | Protein Coding | GC08M018527 | 4.74 |
| HTN3 | Histatin 3 | Protein Coding | GC04P070028 | 4.74 |
| CNTN5 | Contactin 5 | Protein Coding | GC11P099020 | 4.74 |
| TCF7 | Transcription Factor 7 | Protein Coding | GC05P134114 | 4.74 |
| PTPN13 | Protein Tyrosine Phosphatase Non-Receptor Type 13 | Protein Coding | GC04P086594 | 4.74 |
| FAM126A | Family With Sequence Similarity 126 Member A | Protein Coding | GC07M022889 | 4.74 |
| CDC25A | Cell Division Cycle 25A | Protein Coding | GC03M048173 | 4.74 |
| DGCR5 | DiGeorge Syndrome Critical Region Gene 5 | RNA Gene | GC22P019556 | 4.73 |
| HNRNPC | Heterogeneous Nuclear Ribonucleoprotein C | Protein Coding | GC14M021210 | 4.73 |
| CCL13 | C-C Motif Chemokine Ligand 13 | Protein Coding | GC17P034356 | 4.73 |
| MIR495 | MicroRNA 495 | RNA Gene | GC14P104815 | 4.72 |
| SHROOM3 | Shroom Family Member 3 | Protein Coding | GC04P076435 | 4.72 |
| LGALS4 | Galectin 4 | Protein Coding | GC19M042441 | 4.72 |
| PFN1 | Profilin 1 | Protein Coding | GC17M004945 | 4.71 |
| MGST2 | Microsomal Glutathione S-Transferase 2 | Protein Coding | GC04P139665 | 4.71 |
| FERD3L | Fer3 Like BHLH Transcription Factor | Protein Coding | GC07M019184 | 4.71 |
| SLC6A6 | Solute Carrier Family 6 Member 6 | Protein Coding | GC03P014402 | 4.71 |
| PTPRM | Protein Tyrosine Phosphatase Receptor Type M | Protein Coding | GC18P007557 | 4.71 |
| TIAM1 | TIAM Rac1 Associated GEF 1 | Protein Coding | GC21M031118 | 4.71 |
| P2RY1 | Purinergic Receptor P2Y1 | Protein Coding | GC03P152835 | 4.71 |
| CYP46A1 | Cytochrome P450 Family 46 Subfamily A Member 1 | Protein Coding | GC14P099684 | 4.71 |
| CFDP1 | Craniofacial Development Protein 1 | Protein Coding | GC16M075294 | 4.7 |
| LIPK | Lipase Family Member K | Protein Coding | GC10P088724 | 4.7 |
| APH1B | Aph-1 Homolog B, Gamma-Secretase Subunit | Protein Coding | GC15P072972 | 4.7 |
| IRAK3 | Interleukin 1 Receptor Associated Kinase 3 | Protein Coding | GC12P066188 | 4.7 |
| MACF1 | Microtubule Actin Crosslinking Factor 1 | Protein Coding | GC01P039082 | 4.7 |
| RPS3 | Ribosomal Protein S3 | Protein Coding | GC11P075836 | 4.7 |
| CTTN | Cortactin | Protein Coding | GC11P070398 | 4.7 |
| APOM | Apolipoprotein M | Protein Coding | GC06P047315 | 4.69 |
| SCG2 | Secretogranin II | Protein Coding | GC02M223596 | 4.69 |
| PTPN6 | Protein Tyrosine Phosphatase Non-Receptor Type 6 | Protein Coding | GC12P008223 | 4.69 |
| EHBP1 | EH Domain Binding Protein 1 | Protein Coding | GC02P062673 | 4.69 |
| SLC25A28 | Solute Carrier Family 25 Member 28 | Protein Coding | GC10M099610 | 4.68 |
| FUT8 | Fucosyltransferase 8 | Protein Coding | GC14P065411 | 4.68 |
| SULT1A1 | Sulfotransferase Family 1A Member 1 | Protein Coding | GC16M028606 | 4.68 |
| MRTFA | Myocardin Related Transcription Factor A | Protein Coding | GC22M045655 | 4.68 |
| MYO1C | Myosin IC | Protein Coding | GC17M001464 | 4.68 |
| LOC102723692 | Uncharacterized LOC102723692 | RNA Gene | GC16P017134 | 4.67 |
| HLX | H2.0 Like Homeobox | Protein Coding | GC01P220879 | 4.67 |
| TERF2 | Telomeric Repeat Binding Factor 2 | Protein Coding | GC16M069355 | 4.67 |
| PPM1K | Protein Phosphatase, Mg2+/Mn2+ Dependent 1K | Protein Coding | GC04M088258 | 4.67 |
| CHURC1 | Churchill Domain Containing 1 | Protein Coding | GC14P064915 | 4.66 |
| TTLL5 | Tubulin Tyrosine Ligase Like 5 | Protein Coding | GC14P075633 | 4.66 |
| CD7 | CD7 Molecule | Protein Coding | GC17M082314 | 4.66 |
| PPL | Periplakin | Protein Coding | GC16M004872 | 4.66 |
| LRP1B | LDL Receptor Related Protein 1B | Protein Coding | GC02M140231 | 4.65 |
| ESRRG | Estrogen Related Receptor Gamma | Protein Coding | GC01M216503 | 4.65 |
| OPLAH | 5-Oxoprolinase, ATP-Hydrolysing | Protein Coding | GC08M144051 | 4.65 |
| AGBL1 | ATP/GTP Binding Protein Like 1 | Protein Coding | GC15P086141 | 4.64 |
| DSC1 | Desmocollin 1 | Protein Coding | GC18M031129 | 4.64 |
| GPT2 | Glutamic--Pyruvic Transaminase 2 | Protein Coding | GC16P046885 | 4.64 |
| UGT1A8 | UDP Glucuronosyltransferase Family 1 Member A8 | Protein Coding | GC02P233618 | 4.64 |
| NAV2 | Neuron Navigator 2 | Protein Coding | GC11P019345 | 4.64 |
| INPP5D | Inositol Polyphosphate-5-Phosphatase D | Protein Coding | GC02P233059 | 4.64 |
| CMKLR1 | Chemerin Chemokine-Like Receptor 1 | Protein Coding | GC12M108288 | 4.63 |
| PTPRD | Protein Tyrosine Phosphatase Receptor Type D | Protein Coding | GC09M008307 | 4.63 |
| HSPB6 | Heat Shock Protein Family B (Small) Member 6 | Protein Coding | GC19M042410 | 4.63 |
| LOC106029311 | Williams-Beuren Syndrome Centromeric Block B Recombination Region | Biological Region | GC07P073209 | 4.63 |
| LOC106029313 | Williams-Beuren Syndrome Telomeric Block B Recombination Region | Biological Region | GC07P075074 | 4.63 |
| AFA1 | Alopecia, Androgenetic | Genetic Locus | GC03U901250 | 4.63 |
| AGA2 | Alopecia, Androgenetic, 2 | Genetic Locus | GC0XU901287 | 4.63 |
| AGA3 | Alopecia, Androgenetic, 3 | Genetic Locus | GC20U900412 | 4.63 |
| ANGPTL8 | Angiopoietin Like 8 | Protein Coding | GC19P011238 | 4.62 |
| LTB4R | Leukotriene B4 Receptor | Protein Coding | GC14P024311 | 4.62 |
| SYNE3 | Spectrin Repeat Containing Nuclear Envelope Family Member 3 | Protein Coding | GC14M095408 | 4.62 |
| ACSS2 | Acyl-CoA Synthetase Short Chain Family Member 2 | Protein Coding | GC20P034873 | 4.62 |
| IGES | Immunoglobulin E Concentration, Serum | Genetic Locus | GC05U990033 | 4.62 |
| NEDD9 | Neural Precursor Cell Expressed, Developmentally Down-Regulated 9 | Protein Coding | GC06M011183 | 4.61 |
| MIP | Major Intrinsic Protein Of Lens Fiber | Protein Coding | GC12M056449 | 4.61 |
| HLF | HLF Transcription Factor, PAR BZIP Family Member | Protein Coding | GC17P055264 | 4.61 |
| PIM1 | Pim-1 Proto-Oncogene, Serine/Threonine Kinase | Protein Coding | GC06P047469 | 4.61 |
| CRIM1 | Cysteine Rich Transmembrane BMP Regulator 1 | Protein Coding | GC02P036355 | 4.61 |
| PRRC2A | Proline Rich Coiled-Coil 2A | Protein Coding | GC06P047307 | 4.61 |
| CEBPB | CCAAT Enhancer Binding Protein Beta | Protein Coding | GC20P050190 | 4.61 |
| MIR103A1 | MicroRNA 103a-1 | RNA Gene | GC05M168560 | 4.6 |
| ANKRD50 | Ankyrin Repeat Domain 50 | Protein Coding | GC04M124664 | 4.6 |
| TCF7L1 | Transcription Factor 7 Like 1 | Protein Coding | GC02P085133 | 4.6 |
| FLRT2 | Fibronectin Leucine Rich Transmembrane Protein 2 | Protein Coding | GC14P085530 | 4.6 |
| RFX5 | Regulatory Factor X5 | Protein Coding | GC01M151340 | 4.6 |
| F2RL3 | F2R Like Thrombin Or Trypsin Receptor 3 | Protein Coding | GC19P016888 | 4.6 |
| ZBTB20 | Zinc Finger And BTB Domain Containing 20 | Protein Coding | GC03M114315 | 4.59 |
| NRXN3 | Neurexin 3 | Protein Coding | GC14P077980 | 4.59 |
| MLST8 | MTOR Associated Protein, LST8 Homolog | Protein Coding | GC16P002204 | 4.59 |
| MRRF | Mitochondrial Ribosome Recycling Factor | Protein Coding | GC09P122264 | 4.59 |
| POLE | DNA Polymerase Epsilon, Catalytic Subunit | Protein Coding | GC12M132624 | 4.58 |
| ANXA11 | Annexin A11 | Protein Coding | GC10M080150 | 4.58 |
| ARID5B | AT-Rich Interaction Domain 5B | Protein Coding | GC10P061901 | 4.58 |
| RHOC | Ras Homolog Family Member C | Protein Coding | GC01M112701 | 4.58 |
| RIPK2 | Receptor Interacting Serine/Threonine Kinase 2 | Protein Coding | GC08P089758 | 4.57 |
| BCAM | Basal Cell Adhesion Molecule (Lutheran Blood Group) | Protein Coding | GC19P044810 | 4.57 |
| BIRC2 | Baculoviral IAP Repeat Containing 2 | Protein Coding | GC11P102347 | 4.57 |
| SDC2 | Syndecan 2 | Protein Coding | GC08P096494 | 4.57 |
| NRF1 | Nuclear Respiratory Factor 1 | Protein Coding | GC07P129611 | 4.57 |
| ENPP2 | Ectonucleotide Pyrophosphatase/Phosphodiesterase 2 | Protein Coding | GC08M119556 | 4.57 |
| CUX2 | Cut Like Homeobox 2 | Protein Coding | GC12P111034 | 4.56 |
| SLC45A2 | Solute Carrier Family 45 Member 2 | Protein Coding | GC05M033944 | 4.56 |
| PRKAG3 | Protein Kinase AMP-Activated Non-Catalytic Subunit Gamma 3 | Protein Coding | GC02M218823 | 4.56 |
| PLSCR3 | Phospholipid Scramblase 3 | Protein Coding | GC17M007389 | 4.56 |
| CYP2R1 | Cytochrome P450 Family 2 Subfamily R Member 1 | Protein Coding | GC11M014877 | 4.56 |
| PCSK5 | Proprotein Convertase Subtilisin/Kexin Type 5 | Protein Coding | GC09P075890 | 4.55 |
| KHK | Ketohexokinase | Protein Coding | GC02P027086 | 4.55 |
| LIMK2 | LIM Domain Kinase 2 | Protein Coding | GC22P031212 | 4.55 |
| RDX | Radixin | Protein Coding | GC11M109864 | 4.54 |
| PLIN2 | Perilipin 2 | Protein Coding | GC09M019115 | 4.53 |
| NDUFS5 | NADH:Ubiquinone Oxidoreductase Subunit S5 | Protein Coding | GC01P039026 | 4.53 |
| NBPF12 | NBPF Member 12 | Protein Coding | GC01P146938 | 4.53 |
| CHD6 | Chromodomain Helicase DNA Binding Protein 6 | Protein Coding | GC20M041402 | 4.53 |
| GPS1 | G Protein Pathway Suppressor 1 | Protein Coding | GC17P082050 | 4.52 |
| LMAN1 | Lectin, Mannose Binding 1 | Protein Coding | GC18M059327 | 4.52 |
| C1QTNF3 | C1q And TNF Related 3 | Protein Coding | GC05M034017 | 4.52 |
| CARD8 | Caspase Recruitment Domain Family Member 8 | Protein Coding | GC19M048183 | 4.52 |
| UGT2B7 | UDP Glucuronosyltransferase Family 2 Member B7 | Protein Coding | GC04P069051 | 4.52 |
| C5AR2 | Complement Component 5a Receptor 2 | Protein Coding | GC19P047333 | 4.52 |
| MAPKAPK2 | MAPK Activated Protein Kinase 2 | Protein Coding | GC01P206684 | 4.51 |
| SERPINA4 | Serpin Family A Member 4 | Protein Coding | GC14P094561 | 4.51 |
| EXOSC3 | Exosome Component 3 | Protein Coding | GC09M037772 | 4.51 |
| DPEP1 | Dipeptidase 1 | Protein Coding | GC16P089613 | 4.51 |
| SLC24A3 | Solute Carrier Family 24 Member 3 | Protein Coding | GC20P019212 | 4.5 |
| TAL1 | TAL BHLH Transcription Factor 1, Erythroid Differentiation Factor | Protein Coding | GC01M047216 | 4.5 |
| SORCS1 | Sortilin Related VPS10 Domain Containing Receptor 1 | Protein Coding | GC10M106573 | 4.49 |
| MIR432 | MicroRNA 432 | RNA Gene | GC14P104581 | 4.49 |
| ADAMTS1 | ADAM Metallopeptidase With Thrombospondin Type 1 Motif 1 | Protein Coding | GC21M026835 | 4.49 |
| DLGAP1 | DLG Associated Protein 1 | Protein Coding | GC18M003488 | 4.48 |
| BAZ2B | Bromodomain Adjacent To Zinc Finger Domain 2B | Protein Coding | GC02M159318 | 4.48 |
| FMO1 | Flavin Containing Dimethylaniline Monoxygenase 1 | Protein Coding | GC01P171248 | 4.48 |
| PPM1D | Protein Phosphatase, Mg2+/Mn2+ Dependent 1D | Protein Coding | GC17P060600 | 4.48 |
| ABCC3 | ATP Binding Cassette Subfamily C Member 3 | Protein Coding | GC17P050634 | 4.48 |
| TRIB2 | Tribbles Pseudokinase 2 | Protein Coding | GC02P012706 | 4.48 |
| ART4 | ADP-Ribosyltransferase 4 (Dombrock Blood Group) | Protein Coding | GC12M014825 | 4.47 |
| COL8A1 | Collagen Type VIII Alpha 1 Chain | Protein Coding | GC03P099638 | 4.47 |
| PARVB | Parvin Beta | Protein Coding | GC22P043999 | 4.47 |
| TNFRSF12A | TNF Receptor Superfamily Member 12A | Protein Coding | GC16P003018 | 4.46 |
| CA1 | Carbonic Anhydrase 1 | Protein Coding | GC08M085327 | 4.46 |
| DNAJC30 | DnaJ Heat Shock Protein Family (Hsp40) Member C30 | Protein Coding | GC07M073680 | 4.46 |
| NFE2 | Nuclear Factor, Erythroid 2 | Protein Coding | GC12M054292 | 4.45 |
| FAP | Fibroblast Activation Protein Alpha | Protein Coding | GC02M162170 | 4.45 |
| RPS13 | Ribosomal Protein S13 | Protein Coding | GC11M017179 | 4.45 |
| DMRTA1 | DMRT Like Family A1 | Protein Coding | GC09P022436 | 4.45 |
| SOBP | Sine Oculis Binding Protein Homolog | Protein Coding | GC06P107489 | 4.45 |
| TYROBP | Transmembrane Immune Signaling Adaptor TYROBP | Protein Coding | GC19M035904 | 4.45 |
| ARHGAP9 | Rho GTPase Activating Protein 9 | Protein Coding | GC12M057472 | 4.44 |
| OPRL1 | Opioid Related Nociceptin Receptor 1 | Protein Coding | GC20P064080 | 4.44 |
| ST2 | Suppression Of Tumorigenicity 2 | Genetic Locus | GC11U990127 | 4.44 |
| MIR1306 | MicroRNA 1306 | RNA Gene | GC22P020086 | 4.44 |
| ZHX3 | Zinc Fingers And Homeoboxes 3 | Protein Coding | GC20M041178 | 4.44 |
| MIR6886 | MicroRNA 6886 | RNA Gene | GC19P011114 | 4.44 |
| NIF3L1 | NGG1 Interacting Factor 3 Like 1 | Protein Coding | GC02P200889 | 4.44 |
| CYP26A1 | Cytochrome P450 Family 26 Subfamily A Member 1 | Protein Coding | GC10P093073 | 4.43 |
| TDP2 | Tyrosyl-DNA Phosphodiesterase 2 | Protein Coding | GC06M024651 | 4.43 |
| LPAL2 | Lipoprotein(A) Like 2, Pseudogene | Pseudogene | GC06M160453 | 4.42 |
| MIR374B | MicroRNA 374b | RNA Gene | GC0XM074227 | 4.42 |
| CPNE4 | Copine 4 | Protein Coding | GC03M131533 | 4.42 |
| OPRD1 | Opioid Receptor Delta 1 | Protein Coding | GC01P028812 | 4.42 |
| ARL15 | ADP Ribosylation Factor Like GTPase 15 | Protein Coding | GC05M053883 | 4.42 |
| TBPL1 | TATA-Box Binding Protein Like 1 | Protein Coding | GC06P133897 | 4.42 |
| PRKAB2 | Protein Kinase AMP-Activated Non-Catalytic Subunit Beta 2 | Protein Coding | GC01M147155 | 4.41 |
| NDUFC2 | NADH:Ubiquinone Oxidoreductase Subunit C2 | Protein Coding | GC11M078068 | 4.41 |
| C1QTNF1 | C1q And TNF Related 1 | Protein Coding | GC17P079022 | 4.41 |
| TOX | Thymocyte Selection Associated High Mobility Group Box | Protein Coding | GC08M058791 | 4.41 |
| TCF15 | Transcription Factor 15 | Protein Coding | GC20M000603 | 4.41 |
| EPN1 | Epsin 1 | Protein Coding | GC19P055677 | 4.41 |
| TOM1L2 | Target Of Myb1 Like 2 Membrane Trafficking Protein | Protein Coding | GC17M017843 | 4.4 |
| ZNF365 | Zinc Finger Protein 365 | Protein Coding | GC10P062374 | 4.4 |
| RPTOR | Regulatory Associated Protein Of MTOR Complex 1 | Protein Coding | GC17P080544 | 4.4 |
| SPDYE12P | Speedy/RINGO Cell Cycle Regulator Family Member E12, Pseudogene | Protein Coding | GC07M074904 | 4.4 |
| FADS3 | Fatty Acid Desaturase 3 | Protein Coding | GC11M061873 | 4.4 |
| GPR89A | G Protein-Coupled Receptor 89A | Protein Coding | GC01P145607 | 4.4 |
| CHST6 | Carbohydrate Sulfotransferase 6 | Protein Coding | GC16M075472 | 4.4 |
| KCNJ16 | Potassium Inwardly Rectifying Channel Subfamily J Member 16 | Protein Coding | GC17P070053 | 4.39 |
| NSUN5P2 | NSUN5 Pseudogene 2 | Pseudogene | GC07M072948 | 4.39 |
| SIGLEC1 | Sialic Acid Binding Ig Like Lectin 1 | Protein Coding | GC20M003686 | 4.39 |
| RER1 | Retention In Endoplasmic Reticulum Sorting Receptor 1 | Protein Coding | GC01P002391 | 4.39 |
| BLVRB | Biliverdin Reductase B | Protein Coding | GC19M040447 | 4.39 |
| SMPD2 | Sphingomyelin Phosphodiesterase 2 | Protein Coding | GC06P109440 | 4.39 |
| CCR8 | C-C Motif Chemokine Receptor 8 | Protein Coding | GC03P039330 | 4.39 |
| PTAFR | Platelet Activating Factor Receptor | Protein Coding | GC01M028147 | 4.38 |
| PCMT1 | Protein-L-Isoaspartate (D-Aspartate) O-Methyltransferase | Protein Coding | GC06P149749 | 4.38 |
| SLC2A13 | Solute Carrier Family 2 Member 13 | Protein Coding | GC12M039755 | 4.37 |
| PKD1L3 | Polycystin 1 Like 3, Transient Receptor Potential Channel Interacting | Protein Coding | GC16M071962 | 4.37 |
| SKP1 | S-Phase Kinase Associated Protein 1 | Protein Coding | GC05M134148 | 4.37 |
| SSRP1 | Structure Specific Recognition Protein 1 | Protein Coding | GC11M061223 | 4.36 |
| ITPK1 | Inositol-Tetrakisphosphate 1-Kinase | Protein Coding | GC14M092936 | 4.36 |
| GNLY | Granulysin | Protein Coding | GC02P085685 | 4.36 |
| PPP1R12B | Protein Phosphatase 1 Regulatory Subunit 12B | Protein Coding | GC01P202348 | 4.36 |
| FBLIM1 | Filamin Binding LIM Protein 1 | Protein Coding | GC01P015756 | 4.35 |
| UGT1A6 | UDP Glucuronosyltransferase Family 1 Member A6 | Protein Coding | GC02P233691 | 4.34 |
| NLRP6 | NLR Family Pyrin Domain Containing 6 | Protein Coding | GC11P000269 | 4.34 |
| TOMM20 | Translocase Of Outer Mitochondrial Membrane 20 | Protein Coding | GC01M235109 | 4.34 |
| POM121C | POM121 Transmembrane Nucleoporin C | Protein Coding | GC07M075416 | 4.34 |
| MIR17HG | MiR-17-92a-1 Cluster Host Gene | RNA Gene | GC13P091347 | 4.33 |
| TYRP1 | Tyrosinase Related Protein 1 | Protein Coding | GC09P012683 | 4.33 |
| BDKRB1 | Bradykinin Receptor B1 | Protein Coding | GC14P096263 | 4.33 |
| HSPE1 | Heat Shock Protein Family E (Hsp10) Member 1 | Protein Coding | GC02P197501 | 4.33 |
| IL18RAP | Interleukin 18 Receptor Accessory Protein | Protein Coding | GC02P102418 | 4.32 |
| GP5 | Glycoprotein V Platelet | Protein Coding | GC03M194395 | 4.32 |
| ACAD10 | Acyl-CoA Dehydrogenase Family Member 10 | Protein Coding | GC12P111686 | 4.32 |
| PPP2R5A | Protein Phosphatase 2 Regulatory Subunit B'Alpha | Protein Coding | GC01P212285 | 4.32 |
| GTF2IRD2B | GTF2I Repeat Domain Containing 2B | Protein Coding | GC07P075092 | 4.31 |
| ORAI1 | ORAI Calcium Release-Activated Calcium Modulator 1 | Protein Coding | GC12P122835 | 4.31 |
| FMN1 | Formin 1 | Protein Coding | GC15M032765 | 4.31 |
| TRIM5 | Tripartite Motif Containing 5 | Protein Coding | GC11M005693 | 4.31 |
| HPX | Hemopexin | Protein Coding | GC11M006435 | 4.3 |
| TCN1 | Transcobalamin 1 | Protein Coding | GC11M061283 | 4.3 |
| CXCR6 | C-X-C Motif Chemokine Receptor 6 | Protein Coding | GC03P045982 | 4.29 |
| SSBP3 | Single Stranded DNA Binding Protein 3 | Protein Coding | GC01M054225 | 4.29 |
| AQP9 | Aquaporin 9 | Protein Coding | GC15P058138 | 4.29 |
| PLCD1 | Phospholipase C Delta 1 | Protein Coding | GC03M038008 | 4.28 |
| TMPRSS2 | Transmembrane Serine Protease 2 | Protein Coding | GC21M041464 | 4.28 |
| RBM24 | RNA Binding Motif Protein 24 | Protein Coding | GC06P017281 | 4.28 |
| LTC4S | Leukotriene C4 Synthase | Protein Coding | GC05P179793 | 4.28 |
| LRP12 | LDL Receptor Related Protein 12 | Protein Coding | GC08M104489 | 4.28 |
| RTN4R | Reticulon 4 Receptor | Protein Coding | GC22M020234 | 4.28 |
| COQ5 | Coenzyme Q5, Methyltransferase | Protein Coding | GC12M120503 | 4.28 |
| AIMP1 | Aminoacyl TRNA Synthetase Complex Interacting Multifunctional Protein 1 | Protein Coding | GC04P106315 | 4.27 |
| MSH5 | MutS Homolog 5 | Protein Coding | GC06P047322 | 4.27 |
| WWTR1 | WW Domain Containing Transcription Regulator 1 | Protein Coding | GC03M149517 | 4.26 |
| ELMOD2 | ELMO Domain Containing 2 | Protein Coding | GC04P140524 | 4.26 |
| MMP23B | Matrix Metallopeptidase 23B | Protein Coding | GC01P001631 | 4.26 |
| AGPAT1 | 1-Acylglycerol-3-Phosphate O-Acyltransferase 1 | Protein Coding | GC06M032168 | 4.26 |
| AQP7 | Aquaporin 7 | Protein Coding | GC09M033384 | 4.26 |
| MAPKAPK3 | MAPK Activated Protein Kinase 3 | Protein Coding | GC03P050648 | 4.25 |
| CASC15 | Cancer Susceptibility 15 | RNA Gene | GC06P021669 | 4.25 |
| MIRLET7I | MicroRNA Let-7i | RNA Gene | GC12P062606 | 4.25 |
| MIR186 | MicroRNA 186 | RNA Gene | GC01M071067 | 4.25 |
| PSMG1 | Proteasome Assembly Chaperone 1 | Protein Coding | GC21M039174 | 4.25 |
| SSTR5 | Somatostatin Receptor 5 | Protein Coding | GC16P001072 | 4.24 |
| RAPGEF3 | Rap Guanine Nucleotide Exchange Factor 3 | Protein Coding | GC12M047736 | 4.24 |
| TRIM74 | Tripartite Motif Containing 74 | Protein Coding | GC07M072954 | 4.24 |
| PAWR | Pro-Apoptotic WT1 Regulator | Protein Coding | GC12M079574 | 4.23 |
| MIR361 | MicroRNA 361 | RNA Gene | GC0XM085903 | 4.23 |
| CD226 | CD226 Molecule | Protein Coding | GC18M069831 | 4.23 |
| MYO1E | Myosin IE | Protein Coding | GC15M059132 | 4.23 |
| TNFRSF6B | TNF Receptor Superfamily Member 6b | Protein Coding | GC20P063696 | 4.22 |
| VAV3 | Vav Guanine Nucleotide Exchange Factor 3 | Protein Coding | GC01M107571 | 4.22 |
| CYP4V2 | Cytochrome P450 Family 4 Subfamily V Member 2 | Protein Coding | GC04P186191 | 4.22 |
| FCER1A | Fc Fragment Of IgE Receptor Ia | Protein Coding | GC01P159259 | 4.22 |
| BRCC3 | BRCA1/BRCA2-Containing Complex Subunit 3 | Protein Coding | GC0XP155071 | 4.21 |
| CLDN11 | Claudin 11 | Protein Coding | GC03P170418 | 4.21 |
| TMEM87B | Transmembrane Protein 87B | Protein Coding | GC02P115702 | 4.21 |
| NAXE | NAD(P)HX Epimerase | Protein Coding | GC01P156591 | 4.2 |
| C4BPA | Complement Component 4 Binding Protein Alpha | Protein Coding | GC01P207105 | 4.2 |
| SFXN4 | Sideroflexin 4 | Protein Coding | GC10M119140 | 4.2 |
| SLC16A1 | Solute Carrier Family 16 Member 1 | Protein Coding | GC01M112913 | 4.2 |
| CYP2B6 | Cytochrome P450 Family 2 Subfamily B Member 6 | Protein Coding | GC19P040991 | 4.2 |
| SVIP | Small VCP Interacting Protein | Protein Coding | GC11M022799 | 4.19 |
| NCOA6 | Nuclear Receptor Coactivator 6 | Protein Coding | GC20M034696 | 4.19 |
| CMYA5 | Cardiomyopathy Associated 5 | Protein Coding | GC05P079689 | 4.18 |
| RBP1 | Retinol Binding Protein 1 | Protein Coding | GC03M139517 | 4.18 |
| DMWD | DM1 Locus, WD Repeat Containing | Protein Coding | GC19M045782 | 4.17 |
| USP24 | Ubiquitin Specific Peptidase 24 | Protein Coding | GC01M055066 | 4.17 |
| HTR4 | 5-Hydroxytryptamine Receptor 4 | Protein Coding | GC05M148451 | 4.17 |
| FBN3 | Fibrillin 3 | Protein Coding | GC19M008065 | 4.16 |
| B4GALNT2 | Beta-1,4-N-Acetyl-Galactosaminyltransferase 2 | Protein Coding | GC17P049132 | 4.16 |
| MROS | Melkersson-Rosenthal Syndrome | Genetic Locus | GC09U990083 | 4.16 |
| TANC1 | Tetratricopeptide Repeat, Ankyrin Repeat And Coiled-Coil Containing 1 | Protein Coding | GC02P158968 | 4.16 |
| PRSS2 | Serine Protease 2 | Protein Coding | GC07P144959 | 4.15 |
| ERG | ETS Transcription Factor ERG | Protein Coding | GC21M038367 | 4.15 |
| SPDYE1 | Speedy/RINGO Cell Cycle Regulator Family Member E1 | Protein Coding | GC07P043998 | 4.15 |
| STRN | Striatin | Protein Coding | GC02M036815 | 4.15 |
| LTB | Lymphotoxin Beta | Protein Coding | GC06M032590 | 4.15 |
| MLC1 | Modulator Of VRAC Current 1 | Protein Coding | GC22M050059 | 4.14 |
| APH1A | Aph-1 Homolog A, Gamma-Secretase Subunit | Protein Coding | GC01M150265 | 4.14 |
| CHRNB4 | Cholinergic Receptor Nicotinic Beta 4 Subunit | Protein Coding | GC15M078624 | 4.14 |
| DNALI1 | Dynein Axonemal Light Intermediate Chain 1 | Protein Coding | GC01P037567 | 4.14 |
| SULT2B1 | Sulfotransferase Family 2B Member 1 | Protein Coding | GC19P048552 | 4.14 |
| APEH | Acylaminoacyl-Peptide Hydrolase | Protein Coding | GC03P049673 | 4.14 |
| GIMAP5 | GTPase, IMAP Family Member 5 | Protein Coding | GC07P150722 | 4.14 |
| HSF1 | Heat Shock Transcription Factor 1 | Protein Coding | GC08P144291 | 4.13 |
| TNKS | Tankyrase | Protein Coding | GC08P009555 | 4.12 |
| LY86 | Lymphocyte Antigen 86 | Protein Coding | GC06P006588 | 4.12 |
| SH3GL2 | SH3 Domain Containing GRB2 Like 2, Endophilin A1 | Protein Coding | GC09P017569 | 4.12 |
| ZNF648 | Zinc Finger Protein 648 | Protein Coding | GC01M182053 | 4.12 |
| PROX2 | Prospero Homeobox 2 | Protein Coding | GC14M074852 | 4.11 |
| STAG3L3 | Stromal Antigen 3-Like 3 (Pseudogene) | Pseudogene | GC07M072969 | 4.11 |
| FABP5 | Fatty Acid Binding Protein 5 | Protein Coding | GC08P081282 | 4.1 |
| MFSD10 | Major Facilitator Superfamily Domain Containing 10 | Protein Coding | GC04M002903 | 4.1 |
| HPR | Haptoglobin-Related Protein | Protein Coding | GC16P072097 | 4.1 |
| ITGAE | Integrin Subunit Alpha E | Protein Coding | GC17M003722 | 4.1 |
| PSMC5 | Proteasome 26S Subunit, ATPase 5 | Protein Coding | GC17P063827 | 4.1 |
| MXD1 | MAX Dimerization Protein 1 | Protein Coding | GC02P069897 | 4.1 |
| ALDH9A1 | Aldehyde Dehydrogenase 9 Family Member A1 | Protein Coding | GC01M165667 | 4.09 |
| BAD | BCL2 Associated Agonist Of Cell Death | Protein Coding | GC11M064273 | 4.09 |
| CHD9 | Chromodomain Helicase DNA Binding Protein 9 | Protein Coding | GC16P053041 | 4.09 |
| MAD1L1 | Mitotic Arrest Deficient 1 Like 1 | Protein Coding | GC07M001815 | 4.08 |
| TRIM55 | Tripartite Motif Containing 55 | Protein Coding | GC08P066196 | 4.08 |
| DLG2 | Discs Large MAGUK Scaffold Protein 2 | Protein Coding | GC11M083455 | 4.08 |
| THADA | THADA Armadillo Repeat Containing | Protein Coding | GC02M043193 | 4.08 |
| SKP2 | S-Phase Kinase Associated Protein 2 | Protein Coding | GC05P036103 | 4.08 |
| ZNF627 | Zinc Finger Protein 627 | Protein Coding | GC19P011570 | 4.08 |
| PRIMPOL | Primase And DNA Directed Polymerase | Protein Coding | GC04P184649 | 4.08 |
| NBPF3 | NBPF Member 3 | Protein Coding | GC01P021442 | 4.08 |
| ARHGAP10 | Rho GTPase Activating Protein 10 | Protein Coding | GC04P147732 | 4.07 |
| LGALS9 | Galectin 9 | Protein Coding | GC17P027629 | 4.07 |
| FGF19 | Fibroblast Growth Factor 19 | Protein Coding | GC11M069699 | 4.07 |
| FCAR | Fc Fragment Of IgA Receptor | Protein Coding | GC19P055445 | 4.07 |
| NSUN5P1 | NSUN5 Pseudogene 1 | Pseudogene | GC07P075410 | 4.07 |
| IGFBP6 | Insulin Like Growth Factor Binding Protein 6 | Protein Coding | GC12P053097 | 4.07 |
| NCL | Nucleolin | Protein Coding | GC02M231453 | 4.06 |
| RAB11B | RAB11B, Member RAS Oncogene Family | Protein Coding | GC19P008393 | 4.06 |
| STAG3L1 | Stromal Antigen 3-Like 1 (Pseudogene) | Pseudogene | GC07P075359 | 4.06 |
| SPRED2 | Sprouty Related EVH1 Domain Containing 2 | Protein Coding | GC02M065307 | 4.06 |
| PPM1G | Protein Phosphatase, Mg2+/Mn2+ Dependent 1G | Protein Coding | GC02M027382 | 4.06 |
| HNRNPM | Heterogeneous Nuclear Ribonucleoprotein M | Protein Coding | GC19P008444 | 4.06 |
| YRDC | YrdC N6-Threonylcarbamoyltransferase Domain Containing | Protein Coding | GC01M037802 | 4.06 |
| TEAD3 | TEA Domain Transcription Factor 3 | Protein Coding | GC06M042215 | 4.05 |
| MEOX2 | Mesenchyme Homeobox 2 | Protein Coding | GC07M015617 | 4.05 |
| CPQ | Carboxypeptidase Q | Protein Coding | GC08P096645 | 4.05 |
| GTF2IRD2P1 | GTF2I Repeat Domain Containing 2 Pseudogene 1 | Pseudogene | GC07M073242 | 4.04 |
| BDH1 | 3-Hydroxybutyrate Dehydrogenase 1 | Protein Coding | GC03M197519 | 4.04 |
| ACKR2 | Atypical Chemokine Receptor 2 | Protein Coding | GC03P042804 | 4.04 |
| RFXAP | Regulatory Factor X Associated Protein | Protein Coding | GC13P036819 | 4.04 |
| PMVK | Phosphomevalonate Kinase | Protein Coding | GC01M154924 | 4.04 |
| FTMT | Ferritin Mitochondrial | Protein Coding | GC05P121851 | 4.04 |
| TRIM73 | Tripartite Motif Containing 73 | Protein Coding | GC07P075395 | 4.04 |
| E2F1 | E2F Transcription Factor 1 | Protein Coding | GC20M033675 | 4.03 |
| S100A6 | S100 Calcium Binding Protein A6 | Protein Coding | GC01M153535 | 4.03 |
| CRADD | CASP2 And RIPK1 Domain Containing Adaptor With Death Domain | Protein Coding | GC12P093677 | 4.03 |
| NR1D1 | Nuclear Receptor Subfamily 1 Group D Member 1 | Protein Coding | GC17M040092 | 4.02 |
| AMPD2 | Adenosine Monophosphate Deaminase 2 | Protein Coding | GC01P109616 | 4.01 |
| FBF1 | Fas Binding Factor 1 | Protein Coding | GC17M075909 | 4.01 |
| ACKR3 | Atypical Chemokine Receptor 3 | Protein Coding | GC02P236537 | 4 |
| COL22A1 | Collagen Type XXII Alpha 1 Chain | Protein Coding | GC08M138588 | 4 |
| KLRD1 | Killer Cell Lectin Like Receptor D1 | Protein Coding | GC12P010226 | 4 |
| TMEM270 | Transmembrane Protein 270 | Protein Coding | GC07P073862 | 4 |
| AGXT2 | Alanine--Glyoxylate Aminotransferase 2 | Protein Coding | GC05M034998 | 4 |
| GTF2IP4 | General Transcription Factor IIi Pseudogene 4 | Pseudogene | GC07P073206 | 4 |
| AFF1 | AF4/FMR2 Family Member 1 | Protein Coding | GC04P086934 | 3.98 |
| PTN | Pleiotrophin | Protein Coding | GC07M137227 | 3.98 |
| FPR1 | Formyl Peptide Receptor 1 | Protein Coding | GC19M051745 | 3.98 |
| STAG3L2 | Stromal Antigen 3-Like 2 (Pseudogene) | Pseudogene | GC07M074843 | 3.98 |
| CD177 | CD177 Molecule | Protein Coding | GC19P043353 | 3.98 |
| GTF2IP1 | General Transcription Factor IIi Pseudogene 1 | Pseudogene | GC07M075185 | 3.98 |
| SPDYE10P | Speedy/RINGO Cell Cycle Regulator Family Member E10, Pseudogene | Protein Coding | GC07M073104 | 3.98 |
| IL1R2 | Interleukin 1 Receptor Type 2 | Protein Coding | GC02P101991 | 3.97 |
| CEBPD | CCAAT Enhancer Binding Protein Delta | Protein Coding | GC08M047759 | 3.97 |
| ETV1 | ETS Variant Transcription Factor 1 | Protein Coding | GC07M013891 | 3.97 |
| ADHFE1 | Alcohol Dehydrogenase Iron Containing 1 | Protein Coding | GC08P066432 | 3.96 |
| MIR34B | MicroRNA 34b | RNA Gene | GC11P111546 | 3.96 |
| KANTR | KDM5C Adjacent Transcript | RNA Gene | GC0XP053094 | 3.96 |
| NRG3 | Neuregulin 3 | Protein Coding | GC10P083672 | 3.96 |
| PPP3R1 | Protein Phosphatase 3 Regulatory Subunit B, Alpha | Protein Coding | GC02M068143 | 3.96 |
| SLC44A2 | Solute Carrier Family 44 Member 2 | Protein Coding | GC19P010602 | 3.95 |
| PLCL1 | Phospholipase C Like 1 (Inactive) | Protein Coding | GC02P197804 | 3.95 |
| PPY | Pancreatic Polypeptide | Protein Coding | GC17M043940 | 3.95 |
| LINC00851 | Long Intergenic Non-Protein Coding RNA 851 | RNA Gene | GC20P018378 | 3.95 |
| WDR6 | WD Repeat Domain 6 | Protein Coding | GC03P049007 | 3.94 |
| IGFBP4 | Insulin Like Growth Factor Binding Protein 4 | Protein Coding | GC17P040443 | 3.94 |
| ILF3 | Interleukin Enhancer Binding Factor 3 | Protein Coding | GC19P010625 | 3.94 |
| ECM2 | Extracellular Matrix Protein 2 | Protein Coding | GC09M092493 | 3.94 |
| NRBP1 | Nuclear Receptor Binding Protein 1 | Protein Coding | GC02P027427 | 3.94 |
| NLRP5 | NLR Family Pyrin Domain Containing 5 | Protein Coding | GC19P056000 | 3.93 |
| HIVEP2 | HIVEP Zinc Finger 2 | Protein Coding | GC06M142751 | 3.93 |
| CCL24 | C-C Motif Chemokine Ligand 24 | Protein Coding | GC07M075811 | 3.93 |
| STC1 | Stanniocalcin 1 | Protein Coding | GC08M023841 | 3.93 |
| IL31 | Interleukin 31 | Protein Coding | GC12M122173 | 3.93 |
| VIPR1 | Vasoactive Intestinal Peptide Receptor 1 | Protein Coding | GC03P042490 | 3.93 |
| SPDYE13 | Speedy/RINGO Cell Cycle Regulator Family Member E13 | Protein Coding | GC07P075284 | 3.93 |
| BMX | BMX Non-Receptor Tyrosine Kinase | Protein Coding | GC0XP015392 | 3.93 |
| RHOJ | Ras Homolog Family Member J | Protein Coding | GC14P063204 | 3.93 |
| GLB1L3 | Galactosidase Beta 1 Like 3 | Protein Coding | GC11P134269 | 3.92 |
| KLHL26 | Kelch Like Family Member 26 | Protein Coding | GC19P023332 | 3.92 |
| CNDP1 | Carnosine Dipeptidase 1 | Protein Coding | GC18P074534 | 3.92 |
| MED18 | Mediator Complex Subunit 18 | Protein Coding | GC01P028340 | 3.92 |
| ARHGAP15 | Rho GTPase Activating Protein 15 | Protein Coding | GC02P143070 | 3.92 |
| MTNR1A | Melatonin Receptor 1A | Protein Coding | GC04M186533 | 3.92 |
| SPDYE9 | Speedy/RINGO Cell Cycle Regulator Family Member E9 | Protein Coding | GC07M073078 | 3.92 |
| THSD7A | Thrombospondin Type 1 Domain Containing 7A | Protein Coding | GC07M011371 | 3.92 |
| CNOT3 | CCR4-NOT Transcription Complex Subunit 3 | Protein Coding | GC19P055360 | 3.92 |
| ALOX12B | Arachidonate 12-Lipoxygenase, 12R Type | Protein Coding | GC17M008851 | 3.91 |
| COL21A1 | Collagen Type XXI Alpha 1 Chain | Protein Coding | GC06M056057 | 3.91 |
| GTDC1 | Glycosyltransferase Like Domain Containing 1 | Protein Coding | GC02M143938 | 3.91 |
| OAT | Ornithine Aminotransferase | Protein Coding | GC10M124397 | 3.91 |
| PFKP | Phosphofructokinase, Platelet | Protein Coding | GC10P003066 | 3.91 |
| NTM | Neurotrimin | Protein Coding | GC11P131370 | 3.91 |
| SPDYE7P | Speedy/RINGO Cell Cycle Regulator Family Member E7, Pseudogene | Protein Coding | GC07M072862 | 3.9 |
| SPDYE15 | Speedy/RINGO Cell Cycle Regulator Family Member E15 | Protein Coding | GC07P075335 | 3.9 |
| EIF4HP1 | Eukaryotic Translation Initiation Factor 4H Pseudogene 1 | Pseudogene | GC07M027456 | 3.9 |
| GPR182 | G Protein-Coupled Receptor 182 | Protein Coding | GC12P056994 | 3.9 |
| TUBA1B | Tubulin Alpha 1b | Protein Coding | GC12M049127 | 3.9 |
| BHMT2 | Betaine--Homocysteine S-Methyltransferase 2 | Protein Coding | GC05P079071 | 3.9 |
| NDUFAB1 | NADH:Ubiquinone Oxidoreductase Subunit AB1 | Protein Coding | GC16M023582 | 3.9 |
| PFAS | Phosphoribosylformylglycinamidine Synthase | Protein Coding | GC17P008247 | 3.9 |
| ESAM | Endothelial Cell Adhesion Molecule | Protein Coding | GC11M124752 | 3.89 |
| PPP1R3B | Protein Phosphatase 1 Regulatory Subunit 3B | Protein Coding | GC08M009136 | 3.89 |
| BDNF-AS | BDNF Antisense RNA | RNA Gene | GC11P027513 | 3.89 |
| SSTR1 | Somatostatin Receptor 1 | Protein Coding | GC14P038207 | 3.88 |
| HSD17B12 | Hydroxysteroid 17-Beta Dehydrogenase 12 | Protein Coding | GC11P043636 | 3.88 |
| NBPF20 | NBPF Member 20 | Protein Coding | GC01M145289 | 3.88 |
| AGAP1 | ArfGAP With GTPase Domain, Ankyrin Repeat And PH Domain 1 | Protein Coding | GC02P235494 | 3.88 |
| DHX36 | DEAH-Box Helicase 36 | Protein Coding | GC03M154272 | 3.88 |
| CCNE1 | Cyclin E1 | Protein Coding | GC19P029811 | 3.87 |
| ABHD16A | Abhydrolase Domain Containing 16A, Phospholipase | Protein Coding | GC06M032614 | 3.87 |
| TRPC4 | Transient Receptor Potential Cation Channel Subfamily C Member 4 | Protein Coding | GC13M037636 | 3.87 |
| MTSS1 | MTSS I-BAR Domain Containing 1 | Protein Coding | GC08M124550 | 3.86 |
| MIR381 | MicroRNA 381 | RNA Gene | GC14P104797 | 3.86 |
| NUP210 | Nucleoporin 210 | Protein Coding | GC03M015777 | 3.85 |
| MAZ | MYC Associated Zinc Finger Protein | Protein Coding | GC16P029806 | 3.85 |
| DUOXA1 | Dual Oxidase Maturation Factor 1 | Protein Coding | GC15M045119 | 3.85 |
| CXCL6 | C-X-C Motif Chemokine Ligand 6 | Protein Coding | GC04P073837 | 3.85 |
| LOC108228208 | 7q11.23 Proximal Recombination Region | Biological Region | GC07P075442 | 3.85 |
| LOC108228209 | 7q11.23 Distal Recombination Region | Biological Region | GC07P076626 | 3.85 |
| PCOLCE2 | Procollagen C-Endopeptidase Enhancer 2 | Protein Coding | GC03M142815 | 3.85 |
| RND2 | Rho Family GTPase 2 | Protein Coding | GC17P043995 | 3.84 |
| SPDYE8 | Speedy/RINGO Cell Cycle Regulator Family Member E8 | Protein Coding | GC07M073022 | 3.84 |
| SPDYE14 | Speedy/RINGO Cell Cycle Regulator Family Member E14 | Protein Coding | GC07P075300 | 3.84 |
| WBSCR2 | Williams-Beuren Syndrome Chromosome Region 2 | Uncategorized | GC07U990122 | 3.84 |
| WBSCR23 | Williams-Beuren Syndrome Chromosome Region 23 | RNA Gene | GC07P074532 | 3.84 |
| NKX2-3 | NK2 Homeobox 3 | Protein Coding | GC10P099532 | 3.84 |
| RAD51B | RAD51 Paralog B | Protein Coding | GC14P067819 | 3.84 |
| UBIAD1 | UbiA Prenyltransferase Domain Containing 1 | Protein Coding | GC01P011273 | 3.84 |
| MT3 | Metallothionein 3 | Protein Coding | GC16P056589 | 3.83 |
| CADPS | Calcium Dependent Secretion Activator | Protein Coding | GC03M062398 | 3.83 |
| PLEKHO1 | Pleckstrin Homology Domain Containing O1 | Protein Coding | GC01P150121 | 3.83 |
| GINS2 | GINS Complex Subunit 2 | Protein Coding | GC16M085676 | 3.82 |
| EMP1 | Epithelial Membrane Protein 1 | Protein Coding | GC12P013196 | 3.82 |
| RSAD2 | Radical S-Adenosyl Methionine Domain Containing 2 | Protein Coding | GC02P006865 | 3.82 |
| HHAT | Hedgehog Acyltransferase | Protein Coding | GC01P210328 | 3.82 |
| PSMC3 | Proteasome 26S Subunit, ATPase 3 | Protein Coding | GC11M061126 | 3.82 |
| ALMS1P1 | ALMS1 Pseudogene 1 | Pseudogene | GC02P073644 | 3.82 |
| PDGFC | Platelet Derived Growth Factor C | Protein Coding | GC04M156760 | 3.81 |
| PCNX3 | Pecanex 3 | Protein Coding | GC11P065830 | 3.81 |
| CD47 | CD47 Molecule | Protein Coding | GC03M108043 | 3.81 |
| DSTN | Destrin, Actin Depolymerizing Factor | Protein Coding | GC20P017550 | 3.81 |
| MIR107 | MicroRNA 107 | RNA Gene | GC10M089600 | 3.81 |
| LRRC37A2 | Leucine Rich Repeat Containing 37 Member A2 | Protein Coding | GC17P046511 | 3.8 |
| CSH1 | Chorionic Somatomammotropin Hormone 1 | Protein Coding | GC17M063894 | 3.8 |
| PPP1R14C | Protein Phosphatase 1 Regulatory Inhibitor Subunit 14C | Protein Coding | GC06P150143 | 3.8 |
| KLF12 | Kruppel Like Factor 12 | Protein Coding | GC13M073686 | 3.8 |
| BACH1 | BTB Domain And CNC Homolog 1 | Protein Coding | GC21P029194 | 3.8 |
| DCBLD2 | Discoidin, CUB And LCCL Domain Containing 2 | Protein Coding | GC03M098795 | 3.8 |
| ST3GAL3 | ST3 Beta-Galactoside Alpha-2,3-Sialyltransferase 3 | Protein Coding | GC01P043705 | 3.8 |
| DUSP19 | Dual Specificity Phosphatase 19 | Protein Coding | GC02P183078 | 3.8 |
| PRDM9 | PR/SET Domain 9 | Protein Coding | GC05P023443 | 3.79 |
| GPC2 | Glypican 2 | Protein Coding | GC07M100288 | 3.79 |
| TXNRD1 | Thioredoxin Reductase 1 | Protein Coding | GC12P104215 | 3.79 |
| ZFPM2-AS1 | ZFPM2 Antisense RNA 1 | RNA Gene | GC08M105546 | 3.79 |
| PPP1R14A | Protein Phosphatase 1 Regulatory Inhibitor Subunit 14A | Protein Coding | GC19M038251 | 3.79 |
| TICAM1 | Toll Like Receptor Adaptor Molecule 1 | Protein Coding | GC19M004815 | 3.78 |
| RCC1 | Regulator Of Chromosome Condensation 1 | Protein Coding | GC01P028505 | 3.78 |
| KCNK17 | Potassium Two Pore Domain Channel Subfamily K Member 17 | Protein Coding | GC06M042251 | 3.78 |
| PAPPA2 | Pappalysin 2 | Protein Coding | GC01P176463 | 3.78 |
| NAT1 | N-Acetyltransferase 1 | Protein Coding | GC08P018179 | 3.78 |
| EDC4 | Enhancer Of MRNA Decapping 4 | Protein Coding | GC16P067873 | 3.78 |
| RPLP1 | Ribosomal Protein Lateral Stalk Subunit P1 | Protein Coding | GC15P072895 | 3.77 |
| ANGPTL2 | Angiopoietin Like 2 | Protein Coding | GC09M127087 | 3.77 |
| DNM3 | Dynamin 3 | Protein Coding | GC01P171810 | 3.76 |
| MAML3 | Mastermind Like Transcriptional Coactivator 3 | Protein Coding | GC04M139716 | 3.76 |
| TNP1 | Transition Protein 1 | Protein Coding | GC02M216859 | 3.76 |
| NBPF10 | NBPF Member 10 | Protein Coding | GC01M146064 | 3.76 |
| MIR638 | MicroRNA 638 | RNA Gene | GC19P010719 | 3.75 |
| MIR485 | MicroRNA 485 | RNA Gene | GC14P104810 | 3.75 |
| FAM189A2 | Family With Sequence Similarity 189 Member A2 | Protein Coding | GC09P069324 | 3.75 |
| C16orf95 | Chromosome 16 Open Reading Frame 95 | Protein Coding | GC16M087119 | 3.75 |
| CLEC12A | C-Type Lectin Domain Family 12 Member A | Protein Coding | GC12P009951 | 3.75 |
| SLC29A2 | Solute Carrier Family 29 Member 2 | Protein Coding | GC11M066363 | 3.75 |
| PGPEP1 | Pyroglutamyl-Peptidase I | Protein Coding | GC19P023328 | 3.75 |
| SEMA3F | Semaphorin 3F | Protein Coding | GC03P050167 | 3.74 |
| SLC12A9 | Solute Carrier Family 12 Member 9 | Protein Coding | GC07P100826 | 3.74 |
| CABLES1 | Cdk5 And Abl Enzyme Substrate 1 | Protein Coding | GC18P023134 | 3.74 |
| LNX1 | Ligand Of Numb-Protein X 1 | Protein Coding | GC04M053459 | 3.74 |
| ARHGDIA | Rho GDP Dissociation Inhibitor Alpha | Protein Coding | GC17M081867 | 3.73 |
| MIR409 | MicroRNA 409 | RNA Gene | GC14P104806 | 3.73 |
| GPER1 | G Protein-Coupled Estrogen Receptor 1 | Protein Coding | GC07P001188 | 3.73 |
| MTHFSD | Methenyltetrahydrofolate Synthetase Domain Containing | Protein Coding | GC16M086530 | 3.73 |
| GSTO1 | Glutathione S-Transferase Omega 1 | Protein Coding | GC10P104235 | 3.73 |
| PCDH7 | Protocadherin 7 | Protein Coding | GC04P030722 | 3.73 |
| PTPRN2 | Protein Tyrosine Phosphatase Receptor Type N2 | Protein Coding | GC07M157539 | 3.73 |
| IL3RA | Interleukin 3 Receptor Subunit Alpha | Protein Coding | GC0XP001336 | 3.73 |
| GSTM3 | Glutathione S-Transferase Mu 3 | Protein Coding | GC01M109733 | 3.73 |
| AKAP7 | A-Kinase Anchoring Protein 7 | Protein Coding | GC06P131126 | 3.72 |
| IFNAR2 | Interferon Alpha And Beta Receptor Subunit 2 | Protein Coding | GC21P033229 | 3.72 |
| CSRP1 | Cysteine And Glycine Rich Protein 1 | Protein Coding | GC01M201484 | 3.72 |
| TES | Testin LIM Domain Protein | Protein Coding | GC07P116210 | 3.72 |
| POFUT2 | Protein O-Fucosyltransferase 2 | Protein Coding | GC21M045263 | 3.72 |
| DDT | D-Dopachrome Tautomerase | Protein Coding | GC22M023971 | 3.72 |
| ABCC4 | ATP Binding Cassette Subfamily C Member 4 | Protein Coding | GC13M095019 | 3.71 |
| CD93 | CD93 Molecule | Protein Coding | GC20M023079 | 3.71 |
| RMI2 | RecQ Mediated Genome Instability 2 | Protein Coding | GC16P011250 | 3.71 |
| QRICH1 | Glutamine Rich 1 | Protein Coding | GC03M049238 | 3.71 |
| HTR6 | 5-Hydroxytryptamine Receptor 6 | Protein Coding | GC01P019666 | 3.7 |
| FAM189B | Family With Sequence Similarity 189 Member B | Protein Coding | GC01M155248 | 3.7 |
| PGGT1B | Protein Geranylgeranyltransferase Type I Subunit Beta | Protein Coding | GC05M115210 | 3.7 |
| WDFY4 | WDFY Family Member 4 | Protein Coding | GC10P048684 | 3.7 |
| CLASRP | CLK4 Associating Serine/Arginine Rich Protein | Protein Coding | GC19P045039 | 3.7 |
| GUCA2A | Guanylate Cyclase Activator 2A | Protein Coding | GC01M042162 | 3.7 |
| MPRIP | Myosin Phosphatase Rho Interacting Protein | Protein Coding | GC17P017042 | 3.69 |
| HEPHL1 | Hephaestin Like 1 | Protein Coding | GC11P094021 | 3.69 |
| CDH4 | Cadherin 4 | Protein Coding | GC20P061252 | 3.69 |
| HSPA14 | Heat Shock Protein Family A (Hsp70) Member 14 | Protein Coding | GC10P014790 | 3.69 |
| HMGCS2 | 3-Hydroxy-3-Methylglutaryl-CoA Synthase 2 | Protein Coding | GC01M119747 | 3.69 |
| AKAP12 | A-Kinase Anchoring Protein 12 | Protein Coding | GC06P151239 | 3.69 |
| LIPN | Lipase Family Member N | Protein Coding | GC10P088760 | 3.68 |
| PAK1 | P21 (RAC1) Activated Kinase 1 | Protein Coding | GC11M077321 | 3.68 |
| BNIP3 | BCL2 Interacting Protein 3 | Protein Coding | GC10M131966 | 3.68 |
| PFDN4 | Prefoldin Subunit 4 | Protein Coding | GC20P054207 | 3.68 |
| ZNF845 | Zinc Finger Protein 845 | Protein Coding | GC19P053333 | 3.68 |
| LDLR-AS1 | LDLR-AS1 | RNA Gene | GC19M011090 | 3.68 |
| AOPEP | Aminopeptidase O (Putative) | Protein Coding | GC09P094728 | 3.67 |
| SEMA6D | Semaphorin 6D | Protein Coding | GC15P047184 | 3.67 |
| ASIP | Agouti Signaling Protein | Protein Coding | GC20P034194 | 3.67 |
| BCL2A1 | BCL2 Related Protein A1 | Protein Coding | GC15M079961 | 3.67 |
| PSMD9 | Proteasome 26S Subunit, Non-ATPase 9 | Protein Coding | GC12P122847 | 3.67 |
| MAST4 | Microtubule Associated Serine/Threonine Kinase Family Member 4 | Protein Coding | GC05P066596 | 3.67 |
| SYNCRIP | Synaptotagmin Binding Cytoplasmic RNA Interacting Protein | Protein Coding | GC06M085607 | 3.66 |
| RNF215 | Ring Finger Protein 215 | Protein Coding | GC22M030377 | 3.66 |
| MED13 | Mediator Complex Subunit 13 | Protein Coding | GC17M061942 | 3.66 |
| RLN1 | Relaxin 1 | Protein Coding | GC09M005309 | 3.66 |
| BRAP | BRCA1 Associated Protein | Protein Coding | GC12M111642 | 3.66 |
| OFCC1 | Orofacial Cleft 1 Candidate 1 | Protein Coding | GC06M009596 | 3.66 |
| F13B | Coagulation Factor XIII B Chain | Protein Coding | GC01M197008 | 3.66 |
| RGS6 | Regulator Of G Protein Signaling 6 | Protein Coding | GC14P071867 | 3.66 |
| SLC4A7 | Solute Carrier Family 4 Member 7 | Protein Coding | GC03M027372 | 3.66 |
| S1PR2 | Sphingosine-1-Phosphate Receptor 2 | Protein Coding | GC19M010223 | 3.65 |
| GTF3A | General Transcription Factor IIIA | Protein Coding | GC13P027427 | 3.65 |
| RPL10A | Ribosomal Protein L10a | Protein Coding | GC06P047437 | 3.65 |
| EGFLAM | EGF Like, Fibronectin Type III And Laminin G Domains | Protein Coding | GC05P038295 | 3.65 |
| CLCA1 | Chloride Channel Accessory 1 | Protein Coding | GC01P086468 | 3.64 |
| MAPKAP1 | MAPK Associated Protein 1 | Protein Coding | GC09M125437 | 3.64 |
| RASIP1 | Ras Interacting Protein 1 | Protein Coding | GC19M048720 | 3.63 |
| VSNL1 | Visinin Like 1 | Protein Coding | GC02P017539 | 3.63 |
| HES1 | Hes Family BHLH Transcription Factor 1 | Protein Coding | GC03P194136 | 3.63 |
| ADAM9 | ADAM Metallopeptidase Domain 9 | Protein Coding | GC08P038996 | 3.63 |
| GPAA1 | Glycosylphosphatidylinositol Anchor Attachment 1 | Protein Coding | GC08P144082 | 3.62 |
| SNX19 | Sorting Nexin 19 | Protein Coding | GC11M130868 | 3.62 |
| MEAF6 | MYST/Esa1 Associated Factor 6 | Protein Coding | GC01M037489 | 3.62 |
| GOT1 | Glutamic-Oxaloacetic Transaminase 1 | Protein Coding | GC10M099396 | 3.62 |
| ZNF180 | Zinc Finger Protein 180 | Protein Coding | GC19M044475 | 3.62 |
| ZNF202 | Zinc Finger Protein 202 | Protein Coding | GC11M123724 | 3.61 |
| SLCO1B3 | Solute Carrier Organic Anion Transporter Family Member 1B3 | Protein Coding | GC12P020810 | 3.61 |
| GPR108 | G Protein-Coupled Receptor 108 | Protein Coding | GC19M006729 | 3.61 |
| GIMAP4 | GTPase, IMAP Family Member 4 | Protein Coding | GC07P150568 | 3.61 |
| PSMD1 | Proteasome 26S Subunit, Non-ATPase 1 | Protein Coding | GC02P231056 | 3.61 |
| PTPRF | Protein Tyrosine Phosphatase Receptor Type F | Protein Coding | GC01P043527 | 3.6 |
| MARCKS | Myristoylated Alanine Rich Protein Kinase C Substrate | Protein Coding | GC06P113857 | 3.6 |
| G6PC2 | Glucose-6-Phosphatase Catalytic Subunit 2 | Protein Coding | GC02P168901 | 3.6 |
| C12orf43 | Chromosome 12 Open Reading Frame 43 | Protein Coding | GC12M121000 | 3.6 |
| MLIP | Muscular LMNA Interacting Protein | Protein Coding | GC06P053929 | 3.59 |
| PIGR | Polymeric Immunoglobulin Receptor | Protein Coding | GC01M206928 | 3.59 |
| CSPG4 | Chondroitin Sulfate Proteoglycan 4 | Protein Coding | GC15M075674 | 3.59 |
| MTUS2 | Microtubule Associated Scaffold Protein 2 | Protein Coding | GC13P028820 | 3.59 |
| RIT2 | Ras Like Without CAAX 2 | Protein Coding | GC18M042743 | 3.59 |
| IPO5 | Importin 5 | Protein Coding | GC13P097953 | 3.59 |
| KLF10 | Kruppel Like Factor 10 | Protein Coding | GC08M102648 | 3.59 |
| HTR1D | 5-Hydroxytryptamine Receptor 1D | Protein Coding | GC01M023191 | 3.58 |
| NPPA-AS1 | NPPA Antisense RNA 1 | RNA Gene | GC01P011863 | 3.58 |
| CRTC2 | CREB Regulated Transcription Coactivator 2 | Protein Coding | GC01M153947 | 3.58 |
| SPC24 | SPC24 Component Of NDC80 Kinetochore Complex | Protein Coding | GC19M011131 | 3.57 |
| CAPN7 | Calpain 7 | Protein Coding | GC03P015521 | 3.57 |
| LPIN3 | Lipin 3 | Protein Coding | GC20P041340 | 3.57 |
| SIRPA | Signal Regulatory Protein Alpha | Protein Coding | GC20P001894 | 3.57 |
| TUB | TUB Bipartite Transcription Factor | Protein Coding | GC11P008040 | 3.57 |
| MIR363 | MicroRNA 363 | RNA Gene | GC0XM134205 | 3.56 |
| VEZF1 | Vascular Endothelial Zinc Finger 1 | Protein Coding | GC17M057971 | 3.56 |
| CCHCR1 | Coiled-Coil Alpha-Helical Rod Protein 1 | Protein Coding | GC06M031145 | 3.56 |
| AZU1 | Azurocidin 1 | Protein Coding | GC19P000825 | 3.55 |
| ATP6V1B2 | ATPase H+ Transporting V1 Subunit B2 | Protein Coding | GC08P020197 | 3.55 |
| MAP1LC3A | Microtubule Associated Protein 1 Light Chain 3 Alpha | Protein Coding | GC20P034546 | 3.55 |
| VAMP8 | Vesicle Associated Membrane Protein 8 | Protein Coding | GC02P085561 | 3.55 |
| VSIR | V-Set Immunoregulatory Receptor | Protein Coding | GC10M071748 | 3.54 |
| ZGPAT | Zinc Finger CCCH-Type And G-Patch Domain Containing | Protein Coding | GC20P063707 | 3.54 |
| SEC24B | SEC24 Homolog B, COPII Coat Complex Component | Protein Coding | GC04P109433 | 3.54 |
| DMXL2 | Dmx Like 2 | Protein Coding | GC15M051447 | 3.54 |
| LGR6 | Leucine Rich Repeat Containing G Protein-Coupled Receptor 6 | Protein Coding | GC01P202193 | 3.53 |
| APOBEC2 | Apolipoprotein B MRNA Editing Enzyme Catalytic Subunit 2 | Protein Coding | GC06P041053 | 3.53 |
| CEACAM3 | CEA Cell Adhesion Molecule 3 | Protein Coding | GC19P041796 | 3.53 |
| SELENOP | Selenoprotein P | Protein Coding | GC05M042800 | 3.53 |
| SVEP1 | Sushi, Von Willebrand Factor Type A, EGF And Pentraxin Domain Containing 1 | Protein Coding | GC09M110365 | 3.53 |
| RRS1 | Ribosome Biogenesis Regulator 1 Homolog | Protein Coding | GC08P066429 | 3.53 |
| TARBP1 | TAR (HIV-1) RNA Binding Protein 1 | Protein Coding | GC01M234391 | 3.53 |
| TSC22D3 | TSC22 Domain Family Member 3 | Protein Coding | GC0XM107713 | 3.52 |
| IL1RAP | Interleukin 1 Receptor Accessory Protein | Protein Coding | GC03P190514 | 3.52 |
| ATP5MC1 | ATP Synthase Membrane Subunit C Locus 1 | Protein Coding | GC17P048893 | 3.52 |
| HDLBP | High Density Lipoprotein Binding Protein | Protein Coding | GC02M241227 | 3.52 |
| MSRA | Methionine Sulfoxide Reductase A | Protein Coding | GC08P010054 | 3.52 |
| ANKRD55 | Ankyrin Repeat Domain 55 | Protein Coding | GC05M056099 | 3.51 |
| ABI3BP | ABI Family Member 3 Binding Protein | Protein Coding | GC03M100749 | 3.51 |
| EBF1 | EBF Transcription Factor 1 | Protein Coding | GC05M158695 | 3.51 |
| POC5 | POC5 Centriolar Protein | Protein Coding | GC05M075674 | 3.5 |
| PRDX6 | Peroxiredoxin 6 | Protein Coding | GC01P173477 | 3.5 |
| MED9 | Mediator Complex Subunit 9 | Protein Coding | GC17P017476 | 3.5 |
| LYZL1 | Lysozyme Like 1 | Protein Coding | GC10P029297 | 3.5 |
| HNRNPUL1 | Heterogeneous Nuclear Ribonucleoprotein U Like 1 | Protein Coding | GC19P041262 | 3.5 |
| NEXMIF | Neurite Extension And Migration Factor | Protein Coding | GC0XM074733 | 3.5 |
| GIPR | Gastric Inhibitory Polypeptide Receptor | Protein Coding | GC19P045668 | 3.5 |
| DYDC2 | DPY30 Domain Containing 2 | Protein Coding | GC10P080344 | 3.49 |
| SEMA4D | Semaphorin 4D | Protein Coding | GC09M089360 | 3.49 |
| SIK2 | Salt Inducible Kinase 2 | Protein Coding | GC11P111605 | 3.49 |
| DTNB | Dystrobrevin Beta | Protein Coding | GC02M025378 | 3.49 |
| PAPLN | Papilin, Proteoglycan Like Sulfated Glycoprotein | Protein Coding | GC14P073237 | 3.48 |
| SLC15A4 | Solute Carrier Family 15 Member 4 | Protein Coding | GC12M128793 | 3.48 |
| C1orf210 | Chromosome 1 Open Reading Frame 210 | Protein Coding | GC01M043281 | 3.48 |
| TMEM258 | Transmembrane Protein 258 | Protein Coding | GC11M061768 | 3.48 |
| TCP1 | T-Complex 1 | Protein Coding | GC06M159778 | 3.47 |
| C11orf58 | Chromosome 11 Open Reading Frame 58 | Protein Coding | GC11P016613 | 3.47 |
| ALKBH8 | AlkB Homolog 8, TRNA Methyltransferase | Protein Coding | GC11M107502 | 3.47 |
| SMCR5 | Smith-Magenis Syndrome Chromosome Region, Candidate 5 | RNA Gene | GC17M017776 | 3.47 |
| MAP1S | Microtubule Associated Protein 1S | Protein Coding | GC19P023311 | 3.46 |
| H4C3 | H4 Clustered Histone 3 | Protein Coding | GC06P028681 | 3.46 |
| DSCAML1 | DS Cell Adhesion Molecule Like 1 | Protein Coding | GC11M117427 | 3.46 |
| HDGF | Heparin Binding Growth Factor | Protein Coding | GC01M156754 | 3.45 |
| WEE1 | WEE1 G2 Checkpoint Kinase | Protein Coding | GC11P009573 | 3.45 |
| LEPROT | Leptin Receptor Overlapping Transcript | Protein Coding | GC01P065420 | 3.45 |
| SNRPD2 | Small Nuclear Ribonucleoprotein D2 Polypeptide | Protein Coding | GC19M045688 | 3.45 |
| TSBP1 | Testis Expressed Basic Protein 1 | Protein Coding | GC06M032288 | 3.45 |
| HPSE2 | Heparanase 2 (Inactive) | Protein Coding | GC10M098457 | 3.44 |
| SLC2A12 | Solute Carrier Family 2 Member 12 | Protein Coding | GC06M133987 | 3.44 |
| IAH1 | Isoamyl Acetate Hydrolyzing Esterase 1 (Putative) | Protein Coding | GC02P009473 | 3.44 |
| USP16 | Ubiquitin Specific Peptidase 16 | Protein Coding | GC21P029024 | 3.44 |
| MIR345 | MicroRNA 345 | RNA Gene | GC14P100307 | 3.44 |
| HEXD | Hexosaminidase D | Protein Coding | GC17P082419 | 3.43 |
| GLCCI1 | Glucocorticoid Induced 1 | Protein Coding | GC07P007974 | 3.43 |
| E2F2 | E2F Transcription Factor 2 | Protein Coding | GC01M023527 | 3.43 |
| SGIP1 | SH3GL Interacting Endocytic Adaptor 1 | Protein Coding | GC01P066533 | 3.43 |
| SRFBP1 | Serum Response Factor Binding Protein 1 | Protein Coding | GC05P121961 | 3.43 |
| FAM223A | Family With Sequence Similarity 223 Member A | RNA Gene | GC0XP154571 | 3.42 |
| DYDC1 | DPY30 Domain Containing 1 | Protein Coding | GC10M080336 | 3.42 |
| ASH2L | ASH2 Like, Histone Lysine Methyltransferase Complex Subunit | Protein Coding | GC08P038104 | 3.41 |
| ADH7 | Alcohol Dehydrogenase 7 (Class IV), Mu Or Sigma Polypeptide | Protein Coding | GC04M099412 | 3.41 |
| EPGN | Epithelial Mitogen | Protein Coding | GC04P074309 | 3.41 |
| OSR1 | Odd-Skipped Related Transcription Factor 1 | Protein Coding | GC02M019351 | 3.4 |
| TNIK | TRAF2 And NCK Interacting Kinase | Protein Coding | GC03M171061 | 3.4 |
| KCNB2 | Potassium Voltage-Gated Channel Subfamily B Member 2 | Protein Coding | GC08P072532 | 3.4 |
| SLC7A9 | Solute Carrier Family 7 Member 9 | Protein Coding | GC19M032830 | 3.4 |
| EIF4A1 | Eukaryotic Translation Initiation Factor 4A1 | Protein Coding | GC17P007572 | 3.4 |
| MEDAG | Mesenteric Estrogen Dependent Adipogenesis | Protein Coding | GC13P030906 | 3.4 |
| FAM234B | Family With Sequence Similarity 234 Member B | Protein Coding | GC12P013048 | 3.4 |
| CLEC4C | C-Type Lectin Domain Family 4 Member C | Protein Coding | GC12M007695 | 3.4 |
| FNDC3B | Fibronectin Type III Domain Containing 3B | Protein Coding | GC03P172039 | 3.4 |
| AP3D1 | Adaptor Related Protein Complex 3 Subunit Delta 1 | Protein Coding | GC19M002101 | 3.4 |
| RSPO1 | R-Spondin 1 | Protein Coding | GC01M037612 | 3.39 |
| NAALADL2 | N-Acetylated Alpha-Linked Acidic Dipeptidase Like 2 | Protein Coding | GC03P174438 | 3.39 |
| NFIC | Nuclear Factor I C | Protein Coding | GC19P003314 | 3.39 |
| PPIC | Peptidylprolyl Isomerase C | Protein Coding | GC05M123023 | 3.39 |
| PRKCI | Protein Kinase C Iota | Protein Coding | GC03P170222 | 3.39 |
| MCM10 | Minichromosome Maintenance 10 Replication Initiation Factor | Protein Coding | GC10P013161 | 3.39 |
| MYO1D | Myosin ID | Protein Coding | GC17M032492 | 3.39 |
| BAIAP2L1 | BAR/IMD Domain Containing Adaptor Protein 2 Like 1 | Protein Coding | GC07M098294 | 3.39 |
| RPL9 | Ribosomal Protein L9 | Protein Coding | GC04M039452 | 3.39 |
| POMP | Proteasome Maturation Protein | Protein Coding | GC13P028659 | 3.38 |
| USP48 | Ubiquitin Specific Peptidase 48 | Protein Coding | GC01M021678 | 3.38 |
| SORCS2 | Sortilin Related VPS10 Domain Containing Receptor 2 | Protein Coding | GC04P007196 | 3.38 |
| COPS3 | COP9 Signalosome Subunit 3 | Protein Coding | GC17M017246 | 3.38 |
| DMGDH | Dimethylglycine Dehydrogenase | Protein Coding | GC05M078997 | 3.38 |
| DDX31 | DEAD-Box Helicase 31 | Protein Coding | GC09M132499 | 3.38 |
| G3BP1 | G3BP Stress Granule Assembly Factor 1 | Protein Coding | GC05P151771 | 3.38 |
| HAP1 | Huntingtin Associated Protein 1 | Protein Coding | GC17M041717 | 3.37 |
| DRC3 | Dynein Regulatory Complex Subunit 3 | Protein Coding | GC17P017973 | 3.37 |
| POLR3C | RNA Polymerase III Subunit C | Protein Coding | GC01P145824 | 3.37 |
| NEUROG1 | Neurogenin 1 | Protein Coding | GC05M135581 | 3.37 |
| ACAD11 | Acyl-CoA Dehydrogenase Family Member 11 | Protein Coding | GC03M132559 | 3.37 |
| C16orf71 | Chromosome 16 Open Reading Frame 71 | Protein Coding | GC16P004734 | 3.37 |
| CCN3 | Cellular Communication Network Factor 3 | Protein Coding | GC08P119416 | 3.37 |
| XPO5 | Exportin 5 | Protein Coding | GC06M043522 | 3.37 |
| C1GALT1 | Core 1 Synthase, Glycoprotein-N-Acetylgalactosamine 3-Beta-Galactosyltransferase 1 | Protein Coding | GC07P007156 | 3.36 |
| SLC18A1 | Solute Carrier Family 18 Member A1 | Protein Coding | GC08M020144 | 3.36 |
| GUK1 | Guanylate Kinase 1 | Protein Coding | GC01P228139 | 3.36 |
| NCOA1 | Nuclear Receptor Coactivator 1 | Protein Coding | GC02P024492 | 3.36 |
| QKI | QKI, KH Domain Containing RNA Binding | Protein Coding | GC06P163414 | 3.36 |
| IRF2BP2 | Interferon Regulatory Factor 2 Binding Protein 2 | Protein Coding | GC01M234604 | 3.36 |
| SCARA3 | Scavenger Receptor Class A Member 3 | Protein Coding | GC08P027633 | 3.36 |
| CSNK1A1L | Casein Kinase 1 Alpha 1 Like | Protein Coding | GC13M037103 | 3.36 |
| COL15A1 | Collagen Type XV Alpha 1 Chain | Protein Coding | GC09P098943 | 3.35 |
| DEPTOR | DEP Domain Containing MTOR Interacting Protein | Protein Coding | GC08P119873 | 3.35 |
| SF3A1 | Splicing Factor 3a Subunit 1 | Protein Coding | GC22M030331 | 3.35 |
| GPN1 | GPN-Loop GTPase 1 | Protein Coding | GC02P027628 | 3.35 |
| SPRY4-AS1 | SPRY4 Antisense RNA 1 | RNA Gene | GC05P143074 | 3.35 |
| LMCD1 | LIM And Cysteine Rich Domains 1 | Protein Coding | GC03P008518 | 3.34 |
| SVIL | Supervillin | Protein Coding | GC10M030482 | 3.34 |
| MIR101-1 | MicroRNA 101-1 | RNA Gene | GC01M065058 | 3.34 |
| RECK | Reversion Inducing Cysteine Rich Protein With Kazal Motifs | Protein Coding | GC09P036036 | 3.34 |
| PDZK1 | PDZ Domain Containing 1 | Protein Coding | GC01M145670 | 3.34 |
| EYA3 | EYA Transcriptional Coactivator And Phosphatase 3 | Protein Coding | GC01M027970 | 3.34 |
| DNAH2 | Dynein Axonemal Heavy Chain 2 | Protein Coding | GC17P007717 | 3.33 |
| CHMP4B | Charged Multivesicular Body Protein 4B | Protein Coding | GC20P033812 | 3.33 |
| GIMAP2 | GTPase, IMAP Family Member 2 | Protein Coding | GC07P150686 | 3.33 |
| GPAM | Glycerol-3-Phosphate Acyltransferase, Mitochondrial | Protein Coding | GC10M112148 | 3.33 |
| IER3 | Immediate Early Response 3 | Protein Coding | GC06M030743 | 3.32 |
| FAM219B | Family With Sequence Similarity 219 Member B | Protein Coding | GC15M074899 | 3.32 |
| FERMT3 | Fermitin Family Member 3 | Protein Coding | GC11P064206 | 3.32 |
| OXR1 | Oxidation Resistance 1 | Protein Coding | GC08P106271 | 3.32 |
| C1D | C1D Nuclear Receptor Corepressor | Protein Coding | GC02M068041 | 3.32 |
| EML5 | EMAP Like 5 | Protein Coding | GC14M094085 | 3.32 |
| ALKBH5 | AlkB Homolog 5, RNA Demethylase | Protein Coding | GC17P018183 | 3.31 |
| TACC1 | Transforming Acidic Coiled-Coil Containing Protein 1 | Protein Coding | GC08P038728 | 3.31 |
| PI4KB | Phosphatidylinositol 4-Kinase Beta | Protein Coding | GC01M151291 | 3.31 |
| EMSY | EMSY Transcriptional Repressor, BRCA2 Interacting | Protein Coding | GC11P076445 | 3.31 |
| UBE2Z | Ubiquitin Conjugating Enzyme E2 Z | Protein Coding | GC17P048908 | 3.31 |
| MYH15 | Myosin Heavy Chain 15 | Protein Coding | GC03M108380 | 3.31 |
| MAML2 | Mastermind Like Transcriptional Coactivator 2 | Protein Coding | GC11M095976 | 3.31 |
| TATDN1 | TatD DNase Domain Containing 1 | Protein Coding | GC08M124488 | 3.31 |
| E2F4 | E2F Transcription Factor 4 | Protein Coding | GC16P067192 | 3.31 |
| TENT5A | Terminal Nucleotidyltransferase 5A | Protein Coding | GC06M081495 | 3.3 |
| MAPKAPK5 | MAPK Activated Protein Kinase 5 | Protein Coding | GC12P111842 | 3.3 |
| GRIK5 | Glutamate Ionotropic Receptor Kainate Type Subunit 5 | Protein Coding | GC19M041998 | 3.3 |
| NT5C1B | 5'-Nucleotidase, Cytosolic IB | Protein Coding | GC02M018562 | 3.3 |
| MIR92A2 | MicroRNA 92a-2 | RNA Gene | GC0XM134206 | 3.3 |
| SPDEF | SAM Pointed Domain Containing ETS Transcription Factor | Protein Coding | GC06M042210 | 3.3 |
| ACYP2 | Acylphosphatase 2 | Protein Coding | GC02P053970 | 3.29 |
| ACSL1 | Acyl-CoA Synthetase Long Chain Family Member 1 | Protein Coding | GC04M184755 | 3.29 |
| SESN2 | Sestrin 2 | Protein Coding | GC01P028270 | 3.29 |
| INSIG2 | Insulin Induced Gene 2 | Protein Coding | GC02P118088 | 3.29 |
| SLC10A7 | Solute Carrier Family 10 Member 7 | Protein Coding | GC04M146253 | 3.29 |
| ENSG00000272379 |  | RNA Gene | GC06M013290 | 3.29 |
| STRIP1 | Striatin Interacting Protein 1 | Protein Coding | GC01P110032 | 3.28 |
| HSPA1L | Heat Shock Protein Family A (Hsp70) Member 1 Like | Protein Coding | GC06M031809 | 3.28 |
| SDC4 | Syndecan 4 | Protein Coding | GC20M045325 | 3.28 |
| PRMT1 | Protein Arginine Methyltransferase 1 | Protein Coding | GC19P049675 | 3.28 |
| HAS1 | Hyaluronan Synthase 1 | Protein Coding | GC19M051714 | 3.28 |
| DEFA1 | Defensin Alpha 1 | Protein Coding | GC08M006977 | 3.28 |
| RAB31 | RAB31, Member RAS Oncogene Family | Protein Coding | GC18P009701 | 3.28 |
| ACTR2 | Actin Related Protein 2 | Protein Coding | GC02P065227 | 3.28 |
| NPY1R | Neuropeptide Y Receptor Y1 | Protein Coding | GC04M163323 | 3.28 |
| LRGUK | Leucine Rich Repeats And Guanylate Kinase Domain Containing | Protein Coding | GC07P134130 | 3.28 |
| C4B_2 | Complement Component 4B (Chido Blood Group), Copy 2 | Protein Coding | GC06Po03283 | 3.27 |
| PRKD2 | Protein Kinase D2 | Protein Coding | GC19M046674 | 3.27 |
| ARHGEF16 | Rho Guanine Nucleotide Exchange Factor 16 | Protein Coding | GC01P003454 | 3.26 |
| DRAM2 | DNA Damage Regulated Autophagy Modulator 2 | Protein Coding | GC01M111117 | 3.26 |
| TMOD4 | Tropomodulin 4 | Protein Coding | GC01M151169 | 3.26 |
| TENT4A | Terminal Nucleotidyltransferase 4A | Protein Coding | GC05P006713 | 3.26 |
| GADD45A | Growth Arrest And DNA Damage Inducible Alpha | Protein Coding | GC01P067685 | 3.25 |
| S100A11 | S100 Calcium Binding Protein A11 | Protein Coding | GC01M152032 | 3.25 |
| DHX38 | DEAH-Box Helicase 38 | Protein Coding | GC16P072127 | 3.25 |
| EPHA1 | EPH Receptor A1 | Protein Coding | GC07M143390 | 3.25 |
| TSPAN2 | Tetraspanin 2 | Protein Coding | GC01M115050 | 3.25 |
| MLEC | Malectin | Protein Coding | GC12P120687 | 3.25 |
| SLC27A4 | Solute Carrier Family 27 Member 4 | Protein Coding | GC09P128340 | 3.25 |
| SNORD94 | Small Nucleolar RNA, C/D Box 94 | RNA Gene | GC02P086136 | 3.24 |
| MIR615 | MicroRNA 615 | RNA Gene | GC12P054033 | 3.24 |
| MIR129-2 | MicroRNA 129-2 | RNA Gene | GC11P043640 | 3.24 |
| HSF2 | Heat Shock Transcription Factor 2 | Protein Coding | GC06P122399 | 3.24 |
| DTX2 | Deltex E3 Ubiquitin Ligase 2 | Protein Coding | GC07P076461 | 3.24 |
| EGFL7 | EGF Like Domain Multiple 7 | Protein Coding | GC09P136658 | 3.23 |
| HORMAD1 | HORMA Domain Containing 1 | Protein Coding | GC01M150700 | 3.23 |
| IL34 | Interleukin 34 | Protein Coding | GC16P070613 | 3.23 |
| MRPL12 | Mitochondrial Ribosomal Protein L12 | Protein Coding | GC17P081704 | 3.23 |
| TRA | T Cell Receptor Alpha Locus | Protein Coding | GC14P021621 | 3.23 |
| ELF5 | E74 Like ETS Transcription Factor 5 | Protein Coding | GC11M034500 | 3.22 |
| TSR3 | TSR3 Ribosome Maturation Factor | Protein Coding | GC16M001351 | 3.22 |
| HAL | Histidine Ammonia-Lyase | Protein Coding | GC12M095972 | 3.22 |
| ARHGEF12 | Rho Guanine Nucleotide Exchange Factor 12 | Protein Coding | GC11P120336 | 3.22 |
| RSPH6A | Radial Spoke Head 6 Homolog A | Protein Coding | GC19M045795 | 3.22 |
| SNX17 | Sorting Nexin 17 | Protein Coding | GC02P027370 | 3.22 |
| F11-AS1 | F11 Antisense RNA 1 | RNA Gene | GC04M186286 | 3.21 |
| TMEM163 | Transmembrane Protein 163 | Protein Coding | GC02M134455 | 3.21 |
| STEAP4 | STEAP4 Metalloreductase | Protein Coding | GC07M088288 | 3.21 |
| PRRX2 | Paired Related Homeobox 2 | Protein Coding | GC09P129665 | 3.21 |
| EBI3 | Epstein-Barr Virus Induced 3 | Protein Coding | GC19P004232 | 3.2 |
| ANKS1A | Ankyrin Repeat And Sterile Alpha Motif Domain Containing 1A | Protein Coding | GC06P047428 | 3.2 |
| DIO3 | Iodothyronine Deiodinase 3 | Protein Coding | GC14P104662 | 3.19 |
| PTGES | Prostaglandin E Synthase | Protein Coding | GC09M129738 | 3.19 |
| SENP3 | SUMO Specific Peptidase 3 | Protein Coding | GC17P008040 | 3.19 |
| HTR7 | 5-Hydroxytryptamine Receptor 7 | Protein Coding | GC10M090740 | 3.19 |
| NCAN | Neurocan | Protein Coding | GC19P023336 | 3.19 |
| CARMIL1 | Capping Protein Regulator And Myosin 1 Linker 1 | Protein Coding | GC06P025309 | 3.18 |
| ATRAID | All-Trans Retinoic Acid Induced Differentiation Factor | Protein Coding | GC02P027212 | 3.18 |
| SEMA5B | Semaphorin 5B | Protein Coding | GC03M122909 | 3.18 |
| CORO1C | Coronin 1C | Protein Coding | GC12M108645 | 3.18 |
| HIF1AN | Hypoxia Inducible Factor 1 Subunit Alpha Inhibitor | Protein Coding | GC10P100529 | 3.18 |
| ENSG00000236013 |  | Uncategorized | GC06P139976 | 3.18 |
| PTGFR | Prostaglandin F Receptor | Protein Coding | GC01P078303 | 3.18 |
| TRDMT1 | TRNA Aspartic Acid Methyltransferase 1 | Protein Coding | GC10M017098 | 3.17 |
| GSTO2 | Glutathione S-Transferase Omega 2 | Protein Coding | GC10P104268 | 3.17 |
| MORF4L1 | Mortality Factor 4 Like 1 | Protein Coding | GC15P078810 | 3.16 |
| SLC1A5 | Solute Carrier Family 1 Member 5 | Protein Coding | GC19M046760 | 3.16 |
| MT2A | Metallothionein 2A | Protein Coding | GC16P056627 | 3.16 |
| SURF4 | Surfeit 4 | Protein Coding | GC09M133361 | 3.15 |
| IDI1 | Isopentenyl-Diphosphate Delta Isomerase 1 | Protein Coding | GC10M001039 | 3.14 |
| SETD1B | SET Domain Containing 1B, Histone Lysine Methyltransferase | Protein Coding | GC12P122842 | 3.14 |
| RRP1B | Ribosomal RNA Processing 1B | Protein Coding | GC21P043659 | 3.14 |
| FARP2 | FERM, ARH/RhoGEF And Pleckstrin Domain Protein 2 | Protein Coding | GC02P241357 | 3.14 |
| ACAT2 | Acetyl-CoA Acetyltransferase 2 | Protein Coding | GC06P159760 | 3.14 |
| TRIB3 | Tribbles Pseudokinase 3 | Protein Coding | GC20P000361 | 3.14 |
| IMPA1 | Inositol Monophosphatase 1 | Protein Coding | GC08M081656 | 3.14 |
| SYT14 | Synaptotagmin 14 | Protein Coding | GC01P209900 | 3.13 |
| GBP1 | Guanylate Binding Protein 1 | Protein Coding | GC01M089052 | 3.13 |
| ZNF664 | Zinc Finger Protein 664 | Protein Coding | GC12P123971 | 3.13 |
| TBC1D7-LOC100130357 | TBC1D7-LOC100130357 Readthrough | Protein Coding | GC06M013267 | 3.13 |
| BSN | Bassoon Presynaptic Cytomatrix Protein | Protein Coding | GC03P049554 | 3.12 |
| TTLL6 | Tubulin Tyrosine Ligase Like 6 | Protein Coding | GC17M048757 | 3.12 |
| MTHFD2 | Methylenetetrahydrofolate Dehydrogenase (NADP+ Dependent) 2, Methenyltetrahydrofolate Cyclohydrolase | Protein Coding | GC02P074198 | 3.12 |
| AZGP1 | Alpha-2-Glycoprotein 1, Zinc-Binding | Protein Coding | GC07M099967 | 3.12 |
| PIGF | Phosphatidylinositol Glycan Anchor Biosynthesis Class F | Protein Coding | GC02M046580 | 3.12 |
| C1QTNF9 | C1q And TNF Related 9 | Protein Coding | GC13P024307 | 3.12 |
| BYSL | Bystin Like | Protein Coding | GC06P047504 | 3.12 |
| SELENOS | Selenoprotein S | Protein Coding | GC15M103936 | 3.12 |
| ZHX2 | Zinc Fingers And Homeoboxes 2 | Protein Coding | GC08P122781 | 3.11 |
| PEAR1 | Platelet Endothelial Aggregation Receptor 1 | Protein Coding | GC01P156893 | 3.11 |
| LUM | Lumican | Protein Coding | GC12M091102 | 3.11 |
| GALNT1 | Polypeptide N-Acetylgalactosaminyltransferase 1 | Protein Coding | GC18P035581 | 3.11 |
| USP4 | Ubiquitin Specific Peptidase 4 | Protein Coding | GC03M049277 | 3.11 |
| SAA2 | Serum Amyloid A2 | Protein Coding | GC11M018238 | 3.1 |
| PGLYRP1 | Peptidoglycan Recognition Protein 1 | Protein Coding | GC19M046021 | 3.1 |
| SPHK1 | Sphingosine Kinase 1 | Protein Coding | GC17P076376 | 3.1 |
| CYFIP1 | Cytoplasmic FMR1 Interacting Protein 1 | Protein Coding | GC15M022867 | 3.1 |
| INMT | Indolethylamine N-Methyltransferase | Protein Coding | GC07P030737 | 3.1 |
| NEURL1 | Neuralized E3 Ubiquitin Protein Ligase 1 | Protein Coding | GC10P103493 | 3.1 |
| HIPK2 | Homeodomain Interacting Protein Kinase 2 | Protein Coding | GC07M139561 | 3.1 |
| TAS2R38 | Taste 2 Receptor Member 38 | Protein Coding | GC07M141972 | 3.09 |
| KRT74 | Keratin 74 | Protein Coding | GC12M052565 | 3.09 |
| KIAA0319 | KIAA0319 | Protein Coding | GC06M024544 | 3.09 |
| FHII | Hyperaldosteronism, Familial, Type II | Genetic Locus | GC00U936837 | 3.09 |
| GALNT13 | Polypeptide N-Acetylgalactosaminyltransferase 13 | Protein Coding | GC02P153871 | 3.09 |
| EFNB3 | Ephrin B3 | Protein Coding | GC17P008051 | 3.09 |
| MIR323A | MicroRNA 323a | RNA Gene | GC14P104782 | 3.09 |
| ITGA10 | Integrin Subunit Alpha 10 | Protein Coding | GC01M145891 | 3.09 |
| AKR1C3 | Aldo-Keto Reductase Family 1 Member C3 | Protein Coding | GC10P005035 | 3.09 |
| MGLL | Monoglyceride Lipase | Protein Coding | GC03M127689 | 3.09 |
| MSRB1 | Methionine Sulfoxide Reductase B1 | Protein Coding | GC16M001939 | 3.08 |
| IFNL3 | Interferon Lambda 3 | Protein Coding | GC19M039243 | 3.08 |
| PLD2 | Phospholipase D2 | Protein Coding | GC17P004808 | 3.08 |
| PDS5B | PDS5 Cohesin Associated Factor B | Protein Coding | GC13P032586 | 3.08 |
| LAG3 | Lymphocyte Activating 3 | Protein Coding | GC12P008203 | 3.08 |
| ATP5F1B | ATP Synthase F1 Subunit Beta | Protein Coding | GC12M056639 | 3.08 |
| CLCA2 | Chloride Channel Accessory 2 | Protein Coding | GC01P086424 | 3.07 |
| GRHL1 | Grainyhead Like Transcription Factor 1 | Protein Coding | GC02P009942 | 3.07 |
| TAF11 | TATA-Box Binding Protein Associated Factor 11 | Protein Coding | GC06M042211 | 3.07 |
| SEZ6L | Seizure Related 6 Homolog Like | Protein Coding | GC22P026169 | 3.07 |
| BAG2 | BAG Cochaperone 2 | Protein Coding | GC06P057172 | 3.06 |
| PLXNA4 | Plexin A4 | Protein Coding | GC07M132123 | 3.06 |
| PIK3R3 | Phosphoinositide-3-Kinase Regulatory Subunit 3 | Protein Coding | GC01M046041 | 3.05 |
| INSIG1 | Insulin Induced Gene 1 | Protein Coding | GC07P155297 | 3.05 |
| NETO1 | Neuropilin And Tolloid Like 1 | Protein Coding | GC18M072742 | 3.05 |
| USP1 | Ubiquitin Specific Peptidase 1 | Protein Coding | GC01P062436 | 3.05 |
| H2AZ1 | H2A.Z Variant Histone 1 | Protein Coding | GC04M099949 | 3.04 |
| CARM1 | Coactivator Associated Arginine Methyltransferase 1 | Protein Coding | GC19P010871 | 3.04 |
| LGMN | Legumain | Protein Coding | GC14M092703 | 3.04 |
| SLC17A1 | Solute Carrier Family 17 Member 1 | Protein Coding | GC06M025723 | 3.04 |
| CHL1 | Cell Adhesion Molecule L1 Like | Protein Coding | GC03P000213 | 3.04 |
| STARD3 | StAR Related Lipid Transfer Domain Containing 3 | Protein Coding | GC17P039637 | 3.04 |
| SOWAHB | Sosondowah Ankyrin Repeat Domain Family Member B | Protein Coding | GC04M076894 | 3.04 |
| TRMT10A | TRNA Methyltransferase 10A | Protein Coding | GC04M099546 | 3.04 |
| TSPAN9 | Tetraspanin 9 | Protein Coding | GC12P003078 | 3.04 |
| TNFAIP6 | TNF Alpha Induced Protein 6 | Protein Coding | GC02P151357 | 3.03 |
| NCOA2 | Nuclear Receptor Coactivator 2 | Protein Coding | GC08M070109 | 3.03 |
| GPIHBP1 | Glycosylphosphatidylinositol Anchored High Density Lipoprotein Binding Protein 1 | Protein Coding | GC08P143213 | 3.03 |
| CCDC97 | Coiled-Coil Domain Containing 97 | Protein Coding | GC19P041310 | 3.03 |
| HOMER1 | Homer Scaffold Protein 1 | Protein Coding | GC05M079372 | 3.03 |
| RSL24D1 | Ribosomal L24 Domain Containing 1 | Protein Coding | GC15M055180 | 3.03 |
| LRRC17 | Leucine Rich Repeat Containing 17 | Protein Coding | GC07P102912 | 3.03 |
| MTF1 | Metal Regulatory Transcription Factor 1 | Protein Coding | GC01M037810 | 3.02 |
| ACTR3 | Actin Related Protein 3 | Protein Coding | GC02P113889 | 3.02 |
| ZC3HC1 | Zinc Finger C3HC-Type Containing 1 | Protein Coding | GC07M130026 | 3.02 |
| UFSP2 | UFM1 Specific Peptidase 2 | Protein Coding | GC04M185399 | 3.02 |
| ZNF45 | Zinc Finger Protein 45 | Protein Coding | GC19M043914 | 3.02 |
| WDR33 | WD Repeat Domain 33 | Protein Coding | GC02M127701 | 3.02 |
| CD180 | CD180 Molecule | Protein Coding | GC05M067181 | 3.02 |
| AS3MT | Arsenite Methyltransferase | Protein Coding | GC10P102869 | 3.01 |
| ZNF526 | Zinc Finger Protein 526 | Protein Coding | GC19P042220 | 3.01 |
| LAP3 | Leucine Aminopeptidase 3 | Protein Coding | GC04P017578 | 3.01 |
| DPT | Dermatopontin | Protein Coding | GC01M168664 | 3.01 |
| BCAR3 | BCAR3 Adaptor Protein, NSP Family Member | Protein Coding | GC01M093561 | 3 |
| UCN3 | Urocortin 3 | Protein Coding | GC10P005396 | 3 |
| MIR500A | MicroRNA 500a | RNA Gene | GC0XP050008 | 3 |
| PKNOX1 | PBX/Knotted 1 Homeobox 1 | Protein Coding | GC21P042974 | 3 |
| QARS1 | Glutaminyl-TRNA Synthetase 1 | Protein Coding | GC03M049240 | 2.99 |
| POLR1F | RNA Polymerase I Subunit F | Protein Coding | GC07M019695 | 2.99 |
| ATF3 | Activating Transcription Factor 3 | Protein Coding | GC01P212565 | 2.99 |
| MBOAT7 | Membrane Bound O-Acyltransferase Domain Containing 7 | Protein Coding | GC19M054173 | 2.99 |
| RBPMS2 | RNA Binding Protein, MRNA Processing Factor 2 | Protein Coding | GC15M064739 | 2.99 |
| NXPH4 | Neurexophilin 4 | Protein Coding | GC12P057218 | 2.99 |
| ZFAND6 | Zinc Finger AN1-Type Containing 6 | Protein Coding | GC15P080059 | 2.99 |
| SLC17A3 | Solute Carrier Family 17 Member 3 | Protein Coding | GC06M025833 | 2.98 |
| TCEA3 | Transcription Elongation Factor A3 | Protein Coding | GC01M023382 | 2.98 |
| CARMIL3 | Capping Protein Regulator And Myosin 1 Linker 3 | Protein Coding | GC14P024052 | 2.98 |
| SNF8 | SNF8 Subunit Of ESCRT-II | Protein Coding | GC17M048929 | 2.97 |
| MICAL3 | Microtubule Associated Monooxygenase, Calponin And LIM Domain Containing 3 | Protein Coding | GC22M017788 | 2.97 |
| TMEM184C | Transmembrane Protein 184C | Protein Coding | GC04P147617 | 2.97 |
| PRMT8 | Protein Arginine Methyltransferase 8 | Protein Coding | GC12P003381 | 2.97 |
| TMEFF2 | Transmembrane Protein With EGF Like And Two Follistatin Like Domains 2 | Protein Coding | GC02M191950 | 2.97 |
| WNT8B | Wnt Family Member 8B | Protein Coding | GC10P100463 | 2.97 |
| BTC | Betacellulin | Protein Coding | GC04M074744 | 2.97 |
| L3MBTL3 | L3MBTL Histone Methyl-Lysine Binding Protein 3 | Protein Coding | GC06P130013 | 2.96 |
| LGR5 | Leucine Rich Repeat Containing G Protein-Coupled Receptor 5 | Protein Coding | GC12P071439 | 2.96 |
| IL15RA | Interleukin 15 Receptor Subunit Alpha | Protein Coding | GC10M005943 | 2.96 |
| INTS4 | Integrator Complex Subunit 4 | Protein Coding | GC11M077878 | 2.96 |
| UNC119B | Unc-119 Lipid Binding Chaperone B | Protein Coding | GC12P120710 | 2.96 |
| FOSL1 | FOS Like 1, AP-1 Transcription Factor Subunit | Protein Coding | GC11M065909 | 2.95 |
| FER | FER Tyrosine Kinase | Protein Coding | GC05P108747 | 2.95 |
| PHC1 | Polyhomeotic Homolog 1 | Protein Coding | GC12P008913 | 2.95 |
| SLC35G5 | Solute Carrier Family 35 Member G5 | Protein Coding | GC08P011331 | 2.95 |
| SRSF1 | Serine And Arginine Rich Splicing Factor 1 | Protein Coding | GC17M058000 | 2.95 |
| ENSG00000254851 |  | Pseudogene | GC11P117136 | 2.95 |
| CD300LF | CD300 Molecule Like Family Member F | Protein Coding | GC17M074694 | 2.95 |
| LMF1 | Lipase Maturation Factor 1 | Protein Coding | GC16M000853 | 2.94 |
| CAMK1D | Calcium/Calmodulin Dependent Protein Kinase ID | Protein Coding | GC10P012349 | 2.94 |
| COPS8 | COP9 Signalosome Subunit 8 | Protein Coding | GC02P237085 | 2.94 |
| TOX3 | TOX High Mobility Group Box Family Member 3 | Protein Coding | GC16M052471 | 2.94 |
| PHYHIP | Phytanoyl-CoA 2-Hydroxylase Interacting Protein | Protein Coding | GC08M022219 | 2.94 |
| ANO6 | Anoctamin 6 | Protein Coding | GC12P045215 | 2.94 |
| ADAM1A | ADAM Metallopeptidase Domain 1A (Pseudogene) | Pseudogene | GC12P111899 | 2.94 |
| SDF4 | Stromal Cell Derived Factor 4 | Protein Coding | GC01M001216 | 2.93 |
| HHIPL1 | HHIP Like 1 | Protein Coding | GC14P099605 | 2.93 |
| MIR125B2 | MicroRNA 125b-2 | RNA Gene | GC21P016590 | 2.93 |
| BFSP1 | Beaded Filament Structural Protein 1 | Protein Coding | GC20M017493 | 2.93 |
| ADAL | Adenosine Deaminase Like | Protein Coding | GC15P043330 | 2.93 |
| DENND1A | DENN Domain Containing 1A | Protein Coding | GC09M123379 | 2.93 |
| GCN1 | GCN1 Activator Of EIF2AK4 | Protein Coding | GC12M120128 | 2.93 |
| SLC22A11 | Solute Carrier Family 22 Member 11 | Protein Coding | GC11P064573 | 2.92 |
| NUMB | NUMB Endocytic Adaptor Protein | Protein Coding | GC14M073275 | 2.92 |
| MIR128-1 | MicroRNA 128-1 | RNA Gene | GC02P135665 | 2.92 |
| NRP2 | Neuropilin 2 | Protein Coding | GC02P205681 | 2.92 |
| VPS25 | Vacuolar Protein Sorting 25 Homolog | Protein Coding | GC17P042773 | 2.92 |
| CDK5RAP3 | CDK5 Regulatory Subunit Associated Protein 3 | Protein Coding | GC17P047967 | 2.92 |
| LOC105372273 | Uncharacterized LOC105372273 | RNA Gene | GC19P011205 | 2.92 |
| POLK | DNA Polymerase Kappa | Protein Coding | GC05P075511 | 2.92 |
| RHBDF2 | Rhomboid 5 Homolog 2 | Protein Coding | GC17M076470 | 2.92 |
| FRK | Fyn Related Src Family Tyrosine Kinase | Protein Coding | GC06M115931 | 2.92 |
| UCN2 | Urocortin 2 | Protein Coding | GC03M048561 | 2.91 |
| PXN | Paxillin | Protein Coding | GC12M120210 | 2.91 |
| ARHGEF18 | Rho/Rac Guanine Nucleotide Exchange Factor 18 | Protein Coding | GC19P007351 | 2.91 |
| ZFAT | Zinc Finger And AT-Hook Domain Containing | Protein Coding | GC08M134477 | 2.91 |
| PPT2 | Palmitoyl-Protein Thioesterase 2 | Protein Coding | GC06P032153 | 2.91 |
| ANKLE2 | Ankyrin Repeat And LEM Domain Containing 2 | Protein Coding | GC12M132725 | 2.9 |
| RNF43 | Ring Finger Protein 43 | Protein Coding | GC17M058352 | 2.9 |
| RBM6 | RNA Binding Motif Protein 6 | Protein Coding | GC03P049940 | 2.9 |
| B4GALT5 | Beta-1,4-Galactosyltransferase 5 | Protein Coding | GC20M049632 | 2.9 |
| ADAMTS7P3 | ADAMTS7 Pseudogene 3 | Pseudogene | GC15P077976 | 2.89 |
| GLS2 | Glutaminase 2 | Protein Coding | GC12M056470 | 2.89 |
| DUSP2 | Dual Specificity Phosphatase 2 | Protein Coding | GC02M096168 | 2.89 |
| FRMD5 | FERM Domain Containing 5 | Protein Coding | GC15M043870 | 2.89 |
| HSP90AB1 | Heat Shock Protein 90 Alpha Family Class B Member 1 | Protein Coding | GC06P044246 | 2.89 |
| FNDC1 | Fibronectin Type III Domain Containing 1 | Protein Coding | GC06P159379 | 2.89 |
| ANAPC5 | Anaphase Promoting Complex Subunit 5 | Protein Coding | GC12M121308 | 2.89 |
| PDIA2 | Protein Disulfide Isomerase Family A Member 2 | Protein Coding | GC16P001384 | 2.89 |
| HMMR | Hyaluronan Mediated Motility Receptor | Protein Coding | GC05P163480 | 2.88 |
| PARG | Poly(ADP-Ribose) Glycohydrolase | Protein Coding | GC10M049818 | 2.88 |
| PKN2 | Protein Kinase N2 | Protein Coding | GC01P088684 | 2.88 |
| HSPA2 | Heat Shock Protein Family A (Hsp70) Member 2 | Protein Coding | GC14P064535 | 2.88 |
| LGALS7 | Galectin 7 | Protein Coding | GC19M038770 | 2.87 |
| INTS5 | Integrator Complex Subunit 5 | Protein Coding | GC11M063432 | 2.87 |
| TDRKH | Tudor And KH Domain Containing | Protein Coding | GC01M151815 | 2.87 |
| MIR487B | MicroRNA 487b | RNA Gene | GC14P104812 | 2.87 |
| RTN3 | Reticulon 3 | Protein Coding | GC11P063700 | 2.87 |
| PMAIP1 | Phorbol-12-Myristate-13-Acetate-Induced Protein 1 | Protein Coding | GC18P059899 | 2.87 |
| ZNF283 | Zinc Finger Protein 283 | Protein Coding | GC19P043827 | 2.87 |
| IL1F10 | Interleukin 1 Family Member 10 | Protein Coding | GC02P113067 | 2.86 |
| HES5 | Hes Family BHLH Transcription Factor 5 | Protein Coding | GC01M002528 | 2.86 |
| EXOSC4 | Exosome Component 4 | Protein Coding | GC08P144079 | 2.86 |
| IFIT2 | Interferon Induced Protein With Tetratricopeptide Repeats 2 | Protein Coding | GC10P089284 | 2.86 |
| PIP5K1A | Phosphatidylinositol-4-Phosphate 5-Kinase Type 1 Alpha | Protein Coding | GC01P151198 | 2.86 |
| MYO1F | Myosin IF | Protein Coding | GC19M008520 | 2.86 |
| KCTD10 | Potassium Channel Tetramerization Domain Containing 10 | Protein Coding | GC12M109448 | 2.86 |
| MIR136 | MicroRNA 136 | RNA Gene | GC14P104582 | 2.86 |
| PLCH2 | Phospholipase C Eta 2 | Protein Coding | GC01P002425 | 2.85 |
| NLRC5 | NLR Family CARD Domain Containing 5 | Protein Coding | GC16P056990 | 2.85 |
| CPN1 | Carboxypeptidase N Subunit 1 | Protein Coding | GC10M100042 | 2.85 |
| MNS1 | Meiosis Specific Nuclear Structural 1 | Protein Coding | GC15M056421 | 2.85 |
| THSD7B | Thrombospondin Type 1 Domain Containing 7B | Protein Coding | GC02P136765 | 2.85 |
| KCNA3 | Potassium Voltage-Gated Channel Subfamily A Member 3 | Protein Coding | GC01M110654 | 2.85 |
| URB2 | URB2 Ribosome Biogenesis Homolog | Protein Coding | GC01P229626 | 2.85 |
| ALDH1B1 | Aldehyde Dehydrogenase 1 Family Member B1 | Protein Coding | GC09P038392 | 2.85 |
| ZNF222 | Zinc Finger Protein 222 | Protein Coding | GC19P044026 | 2.85 |
| RUFY4 | RUN And FYVE Domain Containing 4 | Protein Coding | GC02P218034 | 2.85 |
| CDC25C | Cell Division Cycle 25C | Protein Coding | GC05M138285 | 2.85 |
| TBL3 | Transducin Beta Like 3 | Protein Coding | GC16P002426 | 2.85 |
| MIR24-2 | MicroRNA 24-2 | RNA Gene | GC19M014008 | 2.84 |
| NFKBIB | NFKB Inhibitor Beta | Protein Coding | GC19P038899 | 2.84 |
| RECQL5 | RecQ Like Helicase 5 | Protein Coding | GC17M075626 | 2.84 |
| SLC22A7 | Solute Carrier Family 22 Member 7 | Protein Coding | GC06P047528 | 2.84 |
| FABP7 | Fatty Acid Binding Protein 7 | Protein Coding | GC06P122779 | 2.83 |
| KCNH7 | Potassium Voltage-Gated Channel Subfamily H Member 7 | Protein Coding | GC02M162371 | 2.83 |
| MIR3936HG | MIR3936 Host Gene | RNA Gene | GC05M132312 | 2.83 |
| KCNK5 | Potassium Two Pore Domain Channel Subfamily K Member 5 | Protein Coding | GC06M042248 | 2.83 |
| CLSTN2 | Calsyntenin 2 | Protein Coding | GC03P139935 | 2.83 |
| ID1 | Inhibitor Of DNA Binding 1, HLH Protein | Protein Coding | GC20P031605 | 2.83 |
| DHRS3 | Dehydrogenase/Reductase 3 | Protein Coding | GC01M012567 | 2.83 |
| RAMP1 | Receptor Activity Modifying Protein 1 | Protein Coding | GC02P237858 | 2.83 |
| GRM8 | Glutamate Metabotropic Receptor 8 | Protein Coding | GC07M126438 | 2.83 |
| NR4A3 | Nuclear Receptor Subfamily 4 Group A Member 3 | Protein Coding | GC09P099821 | 2.83 |
| VWDE | Von Willebrand Factor D And EGF Domains | Protein Coding | GC07M012338 | 2.83 |
| ZEB2-AS1 | ZEB2 Antisense RNA 1 | RNA Gene | GC02P144519 | 2.82 |
| GPRC6A | G Protein-Coupled Receptor Class C Group 6 Member A | Protein Coding | GC06M116793 | 2.82 |
| CLEC4A | C-Type Lectin Domain Family 4 Member A | Protein Coding | GC12P008267 | 2.82 |
| PABPC4 | Poly(A) Binding Protein Cytoplasmic 4 | Protein Coding | GC01M039560 | 2.82 |
| GPR55 | G Protein-Coupled Receptor 55 | Protein Coding | GC02M230907 | 2.82 |
| GLTP | Glycolipid Transfer Protein | Protein Coding | GC12M109850 | 2.82 |
| CCDC63 | Coiled-Coil Domain Containing 63 | Protein Coding | GC12P110846 | 2.81 |
| MTMR10 | Myotubularin Related Protein 10 | Protein Coding | GC15M030938 | 2.81 |
| MYZAP | Myocardial Zonula Adherens Protein | Protein Coding | GC15P057594 | 2.81 |
| SULT1A2 | Sulfotransferase Family 1A Member 2 | Protein Coding | GC16M028591 | 2.81 |
| PLEKHG2 | Pleckstrin Homology And RhoGEF Domain Containing G2 | Protein Coding | GC19P039412 | 2.81 |
| SFRP1 | Secreted Frizzled Related Protein 1 | Protein Coding | GC08M041238 | 2.81 |
| CFAP91 | Cilia And Flagella Associated Protein 91 | Protein Coding | GC03P119705 | 2.81 |
| METTL16 | Methyltransferase Like 16 | Protein Coding | GC17M002405 | 2.81 |
| SNAPC4 | Small Nuclear RNA Activating Complex Polypeptide 4 | Protein Coding | GC09M136375 | 2.8 |
| CSGALNACT2 | Chondroitin Sulfate N-Acetylgalactosaminyltransferase 2 | Protein Coding | GC10P043138 | 2.8 |
| MALRD1 | MAM And LDL Receptor Class A Domain Containing 1 | Protein Coding | GC10P019048 | 2.8 |
| TMEM242 | Transmembrane Protein 242 | Protein Coding | GC06M157289 | 2.8 |
| MIR654 | MicroRNA 654 | RNA Gene | GC14P104823 | 2.8 |
| GPLD1 | Glycosylphosphatidylinositol Specific Phospholipase D1 | Protein Coding | GC06M024425 | 2.8 |
| ANKS1B | Ankyrin Repeat And Sterile Alpha Motif Domain Containing 1B | Protein Coding | GC12M098726 | 2.79 |
| IL24 | Interleukin 24 | Protein Coding | GC01P206897 | 2.79 |
| LIPH | Lipase H | Protein Coding | GC03M185506 | 2.79 |
| MTCH1 | Mitochondrial Carrier 1 | Protein Coding | GC06M042233 | 2.79 |
| MPP7 | Membrane Palmitoylated Protein 7 | Protein Coding | GC10M028057 | 2.78 |
| GORASP1 | Golgi Reassembly Stacking Protein 1 | Protein Coding | GC03M039096 | 2.78 |
| NAAA | N-Acylethanolamine Acid Amidase | Protein Coding | GC04M075910 | 2.78 |
| SNX13 | Sorting Nexin 13 | Protein Coding | GC07M017798 | 2.78 |
| SELENOI | Selenoprotein I | Protein Coding | GC02P026310 | 2.77 |
| SUGP1 | SURP And G-Patch Domain Containing 1 | Protein Coding | GC19M019276 | 2.77 |
| CD84 | CD84 Molecule | Protein Coding | GC01M160541 | 2.77 |
| PDIA3 | Protein Disulfide Isomerase Family A Member 3 | Protein Coding | GC15P043746 | 2.77 |
| DACH1 | Dachshund Family Transcription Factor 1 | Protein Coding | GC13M071437 | 2.77 |
| MAP3K14 | Mitogen-Activated Protein Kinase Kinase Kinase 14 | Protein Coding | GC17M045263 | 2.77 |
| PCSK7 | Proprotein Convertase Subtilisin/Kexin Type 7 | Protein Coding | GC11M117199 | 2.77 |
| MIR99B | MicroRNA 99b | RNA Gene | GC19P051692 | 2.77 |
| TNFSF14 | TNF Superfamily Member 14 | Protein Coding | GC19M006663 | 2.77 |
| MN298114-202 |  | RNA Gene | GC06M042424 | 2.77 |
| KCNK1 | Potassium Two Pore Domain Channel Subfamily K Member 1 | Protein Coding | GC01P233614 | 2.76 |
| TBC1D1 | TBC1 Domain Family Member 1 | Protein Coding | GC04P037892 | 2.76 |
| C8orf49 | Chromosome 8 Open Reading Frame 49 | Protein Coding | GC08P011761 | 2.76 |
| CIC | Capicua Transcriptional Repressor | Protein Coding | GC19P042268 | 2.76 |
| RMC1 | Regulator Of MON1-CCZ1 | Protein Coding | GC18P023504 | 2.76 |
| PAMR1 | Peptidase Domain Containing Associated With Muscle Regeneration 1 | Protein Coding | GC11M035453 | 2.76 |
| MANSC1 | MANSC Domain Containing 1 | Protein Coding | GC12M012326 | 2.76 |
| PRUNE2 | Prune Homolog 2 With BCH Domain | Protein Coding | GC09M076611 | 2.76 |
| IQCH | IQ Motif Containing H | Protein Coding | GC15P067254 | 2.76 |
| RAB3IL1 | RAB3A Interacting Protein Like 1 | Protein Coding | GC11M061897 | 2.76 |
| SEC23IP | SEC23 Interacting Protein | Protein Coding | GC10P119892 | 2.76 |
| ZFP36L1 | ZFP36 Ring Finger Protein Like 1 | Protein Coding | GC14M068787 | 2.75 |
| CCL23 | C-C Motif Chemokine Ligand 23 | Protein Coding | GC17M036013 | 2.75 |
| ABCF2 | ATP Binding Cassette Subfamily F Member 2 | Protein Coding | GC07M151211 | 2.75 |
| FAM167A | Family With Sequence Similarity 167 Member A | Protein Coding | GC08M011421 | 2.75 |
| TEX41 | Testis Expressed 41 | RNA Gene | GC02P144667 | 2.75 |
| ID3 | Inhibitor Of DNA Binding 3, HLH Protein | Protein Coding | GC01M023557 | 2.74 |
| TMEM38A | Transmembrane Protein 38A | Protein Coding | GC19P016661 | 2.74 |
| RAMP3 | Receptor Activity Modifying Protein 3 | Protein Coding | GC07P045163 | 2.74 |
| TIPARP | TCDD Inducible Poly(ADP-Ribose) Polymerase | Protein Coding | GC03P156673 | 2.74 |
| ALCAM | Activated Leukocyte Cell Adhesion Molecule | Protein Coding | GC03P105366 | 2.74 |
| ELOVL2 | ELOVL Fatty Acid Elongase 2 | Protein Coding | GC06M010980 | 2.74 |
| ZNF844 | Zinc Finger Protein 844 | Protein Coding | GC19P012106 | 2.73 |
| TOM1 | Target Of Myb1 Membrane Trafficking Protein | Protein Coding | GC22P035299 | 2.73 |
| GREM2 | Gremlin 2, DAN Family BMP Antagonist | Protein Coding | GC01M240489 | 2.73 |
| MAP3K11 | Mitogen-Activated Protein Kinase Kinase Kinase 11 | Protein Coding | GC11M065598 | 2.73 |
| NME6 | NME/NM23 Nucleoside Diphosphate Kinase 6 | Protein Coding | GC03M048292 | 2.73 |
| WTIP | WT1 Interacting Protein | Protein Coding | GC19P034481 | 2.73 |
| PLEKHA7 | Pleckstrin Homology Domain Containing A7 | Protein Coding | GC11M016778 | 2.73 |
| MUSTN1 | Musculoskeletal, Embryonic Nuclear Protein 1 | Protein Coding | GC03M052834 | 2.73 |
| DUSP5 | Dual Specificity Phosphatase 5 | Protein Coding | GC10P110497 | 2.73 |
| NUMA1 | Nuclear Mitotic Apparatus Protein 1 | Protein Coding | GC11M072002 | 2.73 |
| INKA1 | Inka Box Actin Regulator 1 | Protein Coding | GC03P049803 | 2.73 |
| FREM3 | FRAS1 Related Extracellular Matrix 3 | Protein Coding | GC04M143577 | 2.73 |
| ENSG00000260773 |  | RNA Gene | GC15M066314 | 2.73 |
| SGSM2 | Small G Protein Signaling Modulator 2 | Protein Coding | GC17P002337 | 2.73 |
| LINC00888 | Long Intergenic Non-Protein Coding RNA 888 | RNA Gene | GC03P183447 | 2.73 |
| LRG1 | Leucine Rich Alpha-2-Glycoprotein 1 | Protein Coding | GC19M004538 | 2.72 |
| MAP4 | Microtubule Associated Protein 4 | Protein Coding | GC03M047850 | 2.72 |
| JUND | JunD Proto-Oncogene, AP-1 Transcription Factor Subunit | Protein Coding | GC19M018279 | 2.72 |
| NAA25 | N-Alpha-Acetyltransferase 25, NatB Auxiliary Subunit | Protein Coding | GC12M112026 | 2.72 |
| EPB41L3 | Erythrocyte Membrane Protein Band 4.1 Like 3 | Protein Coding | GC18M005382 | 2.72 |
| KCNK15 | Potassium Two Pore Domain Channel Subfamily K Member 15 | Protein Coding | GC20P044745 | 2.72 |
| NUB1 | Negative Regulator Of Ubiquitin Like Proteins 1 | Protein Coding | GC07P151341 | 2.72 |
| PNOC | Prepronociceptin | Protein Coding | GC08P028316 | 2.72 |
| C1orf56 | Chromosome 1 Open Reading Frame 56 | Protein Coding | GC01P151047 | 2.72 |
| GRIN3A | Glutamate Ionotropic Receptor NMDA Type Subunit 3A | Protein Coding | GC09M101569 | 2.72 |
| CRISPLD1 | Cysteine Rich Secretory Protein LCCL Domain Containing 1 | Protein Coding | GC08P074984 | 2.71 |
| MIR769 | MicroRNA 769 | RNA Gene | GC19P046018 | 2.71 |
| GNPDA2 | Glucosamine-6-Phosphate Deaminase 2 | Protein Coding | GC04M044684 | 2.71 |
| LZTS1 | Leucine Zipper Tumor Suppressor 1 | Protein Coding | GC08M020246 | 2.71 |
| RNF214 | Ring Finger Protein 214 | Protein Coding | GC11P117232 | 2.71 |
| SORBS3 | Sorbin And SH3 Domain Containing 3 | Protein Coding | GC08P022544 | 2.71 |
| LILRB2 | Leukocyte Immunoglobulin Like Receptor B2 | Protein Coding | GC19M054409 | 2.71 |
| PREB | Prolactin Regulatory Element Binding | Protein Coding | GC02M027130 | 2.71 |
| PRPF38B | Pre-MRNA Processing Factor 38B | Protein Coding | GC01P108692 | 2.71 |
| ZBTB8OS | Zinc Finger And BTB Domain Containing 8 Opposite Strand | Protein Coding | GC01M032600 | 2.7 |
| FOXQ1 | Forkhead Box Q1 | Protein Coding | GC06P001312 | 2.7 |
| MTERF1 | Mitochondrial Transcription Termination Factor 1 | Protein Coding | GC07M091692 | 2.7 |
| GMDS | GDP-Mannose 4,6-Dehydratase | Protein Coding | GC06M001624 | 2.7 |
| RIPK3 | Receptor Interacting Serine/Threonine Kinase 3 | Protein Coding | GC14M024336 | 2.7 |
| RETNLB | Resistin Like Beta | Protein Coding | GC03M108743 | 2.7 |
| CORT | Cortistatin | Protein Coding | GC01P010449 | 2.7 |
| CALCB | Calcitonin Related Polypeptide Beta | Protein Coding | GC11P014904 | 2.69 |
| MIR212 | MicroRNA 212 | RNA Gene | GC17M002050 | 2.69 |
| TAF3 | TATA-Box Binding Protein Associated Factor 3 | Protein Coding | GC10P007860 | 2.69 |
| SOX7 | SRY-Box Transcription Factor 7 | Protein Coding | GC08M010723 | 2.69 |
| ADAMTS8 | ADAM Metallopeptidase With Thrombospondin Type 1 Motif 8 | Protein Coding | GC11M130308 | 2.69 |
| TBC1D23 | TBC1 Domain Family Member 23 | Protein Coding | GC03P100260 | 2.69 |
| SUPT5H | SPT5 Homolog, DSIF Elongation Factor Subunit | Protein Coding | GC19P039436 | 2.69 |
| GSTM2 | Glutathione S-Transferase Mu 2 | Protein Coding | GC01P109668 | 2.69 |
| LEKR1 | Leucine, Glutamate And Lysine Rich 1 | Protein Coding | GC03P156825 | 2.69 |
| ANKRD6 | Ankyrin Repeat Domain 6 | Protein Coding | GC06P089433 | 2.69 |
| NUDT1 | Nudix Hydrolase 1 | Protein Coding | GC07P002242 | 2.68 |
| RSPO3 | R-Spondin 3 | Protein Coding | GC06P127118 | 2.68 |
| DENND5A | DENN Domain Containing 5A | Protein Coding | GC11M009162 | 2.68 |
| INTS10 | Integrator Complex Subunit 10 | Protein Coding | GC08P019817 | 2.68 |
| SMC5 | Structural Maintenance Of Chromosomes 5 | Protein Coding | GC09P070258 | 2.68 |
| ARID3A | AT-Rich Interaction Domain 3A | Protein Coding | GC19P000926 | 2.68 |
| DACT3 | Dishevelled Binding Antagonist Of Beta Catenin 3 | Protein Coding | GC19M046647 | 2.68 |
| TMEM59 | Transmembrane Protein 59 | Protein Coding | GC01M054031 | 2.68 |
| SLC16A9 | Solute Carrier Family 16 Member 9 | Protein Coding | GC10M059650 | 2.68 |
| TTLL7 | Tubulin Tyrosine Ligase Like 7 | Protein Coding | GC01M083865 | 2.68 |
| CREB3 | CAMP Responsive Element Binding Protein 3 | Protein Coding | GC09P035722 | 2.68 |
| MAP3K6 | Mitogen-Activated Protein Kinase Kinase Kinase 6 | Protein Coding | GC01M027365 | 2.67 |
| ZNF385D | Zinc Finger Protein 385D | Protein Coding | GC03M021412 | 2.67 |
| ZNF592 | Zinc Finger Protein 592 | Protein Coding | GC15P084824 | 2.67 |
| TSHZ2 | Teashirt Zinc Finger Homeobox 2 | Protein Coding | GC20P052972 | 2.67 |
| EFNA1 | Ephrin A1 | Protein Coding | GC01P155127 | 2.67 |
| EREG | Epiregulin | Protein Coding | GC04P074366 | 2.67 |
| TPSAB1 | Tryptase Alpha/Beta 1 | Protein Coding | GC16P001240 | 2.67 |
| MPP3 | Membrane Palmitoylated Protein 3 | Protein Coding | GC17M043800 | 2.66 |
| CTAGE1 | Cutaneous T Cell Lymphoma-Associated Antigen 1 | Protein Coding | GC18M022413 | 2.66 |
| BEND6 | BEN Domain Containing 6 | Protein Coding | GC06P056954 | 2.66 |
| XAB2 | XPA Binding Protein 2 | Protein Coding | GC19M007619 | 2.66 |
| CMIP | C-Maf Inducing Protein | Protein Coding | GC16P081445 | 2.66 |
| MIR503 | MicroRNA 503 | RNA Gene | GC0XM134626 | 2.65 |
| GOLGB1 | Golgin B1 | Protein Coding | GC03M121663 | 2.65 |
| SLC6A20 | Solute Carrier Family 6 Member 20 | Protein Coding | GC03M045755 | 2.65 |
| APOO | Apolipoprotein O | Protein Coding | GC0XM023834 | 2.65 |
| ENSG00000258634 |  | RNA Gene | GC01P110058 | 2.65 |
| AURKAIP1 | Aurora Kinase A Interacting Protein 1 | Protein Coding | GC01M001373 | 2.65 |
| GSC2 | Goosecoid Homeobox 2 | Protein Coding | GC22M019148 | 2.65 |
| KLHL29 | Kelch Like Family Member 29 | Protein Coding | GC02P023348 | 2.65 |
| FSTL4 | Follistatin Like 4 | Protein Coding | GC05M133196 | 2.65 |
| BDP1 | B Double Prime 1, Subunit Of RNA Polymerase III Transcription Initiation Factor IIIB | Protein Coding | GC05P071456 | 2.64 |
| MIR484 | MicroRNA 484 | RNA Gene | GC16P015657 | 2.64 |
| SMCR2 | Smith-Magenis Syndrome Chromosome Region, Candidate 2 | RNA Gene | GC17M017674 | 2.64 |
| MCUR1 | Mitochondrial Calcium Uniporter Regulator 1 | Protein Coding | GC06M013788 | 2.64 |
| LMO2 | LIM Domain Only 2 | Protein Coding | GC11M033858 | 2.63 |
| LRIG1 | Leucine Rich Repeats And Immunoglobulin Like Domains 1 | Protein Coding | GC03M066429 | 2.63 |
| MINPP1 | Multiple Inositol-Polyphosphate Phosphatase 1 | Protein Coding | GC10P087504 | 2.63 |
| SLC30A1 | Solute Carrier Family 30 Member 1 | Protein Coding | GC01M211571 | 2.63 |
| FBXO46 | F-Box Protein 46 | Protein Coding | GC19M045710 | 2.63 |
| GAS2 | Growth Arrest Specific 2 | Protein Coding | GC11P022626 | 2.62 |
| BFAR | Bifunctional Apoptosis Regulator | Protein Coding | GC16P014634 | 2.62 |
| AMFR | Autocrine Motility Factor Receptor | Protein Coding | GC16M056361 | 2.62 |
| APPBP2 | Amyloid Beta Precursor Protein Binding Protein 2 | Protein Coding | GC17M060443 | 2.62 |
| SLC30A3 | Solute Carrier Family 30 Member 3 | Protein Coding | GC02M027254 | 2.62 |
| TDRD10 | Tudor Domain Containing 10 | Protein Coding | GC01P154502 | 2.62 |
| EMILIN3 | Elastin Microfibril Interfacer 3 | Protein Coding | GC20M041359 | 2.61 |
| PRMT5 | Protein Arginine Methyltransferase 5 | Protein Coding | GC14M022920 | 2.61 |
| CNTROB | Centrobin, Centriole Duplication And Spindle Assembly Protein | Protein Coding | GC17P007932 | 2.61 |
| ERVFRD-1 | Endogenous Retrovirus Group FRD Member 1, Envelope | Protein Coding | GC06M011103 | 2.61 |
| MIR30C1 | MicroRNA 30c-1 | RNA Gene | GC01P040757 | 2.61 |
| P2RY11 | Purinergic Receptor P2Y11 | Protein Coding | GC19P010149 | 2.61 |
| EXOC3L2 | Exocyst Complex Component 3 Like 2 | Protein Coding | GC19M045212 | 2.61 |
| C1orf167 | Chromosome 1 Open Reading Frame 167 | Protein Coding | GC01P011761 | 2.6 |
| MIR128-2 | MicroRNA 128-2 | RNA Gene | GC03P035750 | 2.6 |
| IMPDH2 | Inosine Monophosphate Dehydrogenase 2 | Protein Coding | GC03M049253 | 2.6 |
| UBASH3B | Ubiquitin Associated And SH3 Domain Containing B | Protein Coding | GC11P122655 | 2.6 |
| SESN1 | Sestrin 1 | Protein Coding | GC06M108986 | 2.6 |
| TRIM24 | Tripartite Motif Containing 24 | Protein Coding | GC07P138460 | 2.6 |
| PPFIA2 | PTPRF Interacting Protein Alpha 2 | Protein Coding | GC12M081257 | 2.6 |
| ENSG00000250155 |  | RNA Gene | GC05M036668 | 2.6 |
| NME3 | NME/NM23 Nucleoside Diphosphate Kinase 3 | Protein Coding | GC16M001770 | 2.59 |
| EDIL3 | EGF Like Repeats And Discoidin Domains 3 | Protein Coding | GC05M083940 | 2.59 |
| CSRP2 | Cysteine And Glycine Rich Protein 2 | Protein Coding | GC12M076859 | 2.59 |
| FGD6 | FYVE, RhoGEF And PH Domain Containing 6 | Protein Coding | GC12M095076 | 2.59 |
| GOLM2 | Golgi Membrane Protein 2 | Protein Coding | GC15P044289 | 2.59 |
| NR2C2 | Nuclear Receptor Subfamily 2 Group C Member 2 | Protein Coding | GC03P014947 | 2.59 |
| ENSA | Endosulfine Alpha | Protein Coding | GC01M150688 | 2.59 |
| MIR181D | MicroRNA 181d | RNA Gene | GC19P013874 | 2.59 |
| PARL | Presenilin Associated Rhomboid Like | Protein Coding | GC03M183825 | 2.58 |
| MIR325 | MicroRNA 325 | RNA Gene | GC0XM077005 | 2.58 |
| MDN1 | Midasin AAA ATPase 1 | Protein Coding | GC06M089642 | 2.58 |
| PARPBP | PARP1 Binding Protein | Protein Coding | GC12P102120 | 2.58 |
| LPCAT3 | Lysophosphatidylcholine Acyltransferase 3 | Protein Coding | GC12M006976 | 2.58 |
| ATP10A | ATPase Phospholipid Transporting 10A (Putative) | Protein Coding | GC15M025666 | 2.58 |
| CDC42BPA | CDC42 Binding Protein Kinase Alpha | Protein Coding | GC01M226989 | 2.58 |
| LECT2 | Leukocyte Cell Derived Chemotaxin 2 | Protein Coding | GC05M135922 | 2.57 |
| THAP5 | THAP Domain Containing 5 | Protein Coding | GC07M108554 | 2.57 |
| SPPL3 | Signal Peptide Peptidase Like 3 | Protein Coding | GC12M121051 | 2.57 |
| NBL1 | NBL1, DAN Family BMP Antagonist | Protein Coding | GC01P019600 | 2.57 |
| CLEC3B | C-Type Lectin Domain Family 3 Member B | Protein Coding | GC03P045357 | 2.57 |
| SSNA1 | SS Nuclear Autoantigen 1 | Protein Coding | GC09P137188 | 2.57 |
| MARK4 | Microtubule Affinity Regulating Kinase 4 | Protein Coding | GC19P045079 | 2.57 |
| BSX | Brain Specific Homeobox | Protein Coding | GC11M122977 | 2.56 |
| ST3GAL1 | ST3 Beta-Galactoside Alpha-2,3-Sialyltransferase 1 | Protein Coding | GC08M133454 | 2.56 |
| ZNF441 | Zinc Finger Protein 441 | Protein Coding | GC19P011767 | 2.56 |
| CARD16 | Caspase Recruitment Domain Family Member 16 | Protein Coding | GC11M105041 | 2.56 |
| PLEKHG1 | Pleckstrin Homology And RhoGEF Domain Containing G1 | Protein Coding | GC06P150599 | 2.56 |
| UHRF1BP1 | UHRF1 Binding Protein 1 | Protein Coding | GC06P047427 | 2.55 |
| RSU1 | Ras Suppressor Protein 1 | Protein Coding | GC10M016672 | 2.55 |
| SHC4 | SHC Adaptor Protein 4 | Protein Coding | GC15M048823 | 2.55 |
| DPH2 | Diphthamide Biosynthesis 2 | Protein Coding | GC01P043969 | 2.55 |
| TRERF1 | Transcriptional Regulating Factor 1 | Protein Coding | GC06M042224 | 2.55 |
| FAM193A | Family With Sequence Similarity 193 Member A | Protein Coding | GC04P002536 | 2.55 |
| TRIM72 | Tripartite Motif Containing 72 | Protein Coding | GC16P031549 | 2.55 |
| GNA12 | G Protein Subunit Alpha 12 | Protein Coding | GC07M002728 | 2.55 |
| KIR2DL3 | Killer Cell Immunoglobulin Like Receptor, Two Ig Domains And Long Cytoplasmic Tail 3 | Protein Coding | GC19P055469 | 2.55 |
| BCAP29 | B Cell Receptor Associated Protein 29 | Protein Coding | GC07P107579 | 2.55 |
| CREG1 | Cellular Repressor Of E1A Stimulated Genes 1 | Protein Coding | GC01M167498 | 2.55 |
| MXD3 | MAX Dimerization Protein 3 | Protein Coding | GC05M177408 | 2.54 |
| CALU | Calumenin | Protein Coding | GC07P128739 | 2.54 |
| RRM1 | Ribonucleotide Reductase Catalytic Subunit M1 | Protein Coding | GC11P004115 | 2.53 |
| PRORP | Protein Only RNase P Catalytic Subunit | Protein Coding | GC14P035123 | 2.53 |
| ATG4C | Autophagy Related 4C Cysteine Peptidase | Protein Coding | GC01P062784 | 2.53 |
| ZC2HC1C | Zinc Finger C2HC-Type Containing 1C | Protein Coding | GC14P075064 | 2.53 |
| FGD5 | FYVE, RhoGEF And PH Domain Containing 5 | Protein Coding | GC03P014810 | 2.53 |
| CTHRC1 | Collagen Triple Helix Repeat Containing 1 | Protein Coding | GC08P103371 | 2.53 |
| SLCO2B1 | Solute Carrier Organic Anion Transporter Family Member 2B1 | Protein Coding | GC11P075835 | 2.53 |
| ELL | Elongation Factor For RNA Polymerase II | Protein Coding | GC19M018443 | 2.52 |
| GRID2IP | Grid2 Interacting Protein | Protein Coding | GC07M006496 | 2.52 |
| PIAS3 | Protein Inhibitor Of Activated STAT 3 | Protein Coding | GC01M145848 | 2.52 |
| ZNF491 | Zinc Finger Protein 491 | Protein Coding | GC19P011773 | 2.52 |
| NPY2R | Neuropeptide Y Receptor Y2 | Protein Coding | GC04P155173 | 2.52 |
| SPNS2 | Sphingolipid Transporter 2 | Protein Coding | GC17P004498 | 2.52 |
| CRTC3 | CREB Regulated Transcription Coactivator 3 | Protein Coding | GC15P090529 | 2.52 |
| BLID | BH3-Like Motif Containing, Cell Death Inducer | Protein Coding | GC11M122115 | 2.52 |
| RNF139 | Ring Finger Protein 139 | Protein Coding | GC08P124474 | 2.52 |
| ZFP36L2 | ZFP36 Ring Finger Protein Like 2 | Protein Coding | GC02M043184 | 2.52 |
| GIMAP1 | GTPase, IMAP Family Member 1 | Protein Coding | GC07P150717 | 2.52 |
| ABCC5 | ATP Binding Cassette Subfamily C Member 5 | Protein Coding | GC03M183919 | 2.52 |
| SLCO3A1 | Solute Carrier Organic Anion Transporter Family Member 3A1 | Protein Coding | GC15P091856 | 2.52 |
| ROPN1L | Rhophilin Associated Tail Protein 1 Like | Protein Coding | GC05P010441 | 2.51 |
| ENSG00000256879 |  | RNA Gene | GC12M020361 | 2.51 |
| PIF1 | PIF1 5'-To-3' DNA Helicase | Protein Coding | GC15M064815 | 2.51 |
| CCDC3 | Coiled-Coil Domain Containing 3 | Protein Coding | GC10M012896 | 2.51 |
| DOCK1 | Dedicator Of Cytokinesis 1 | Protein Coding | GC10P126905 | 2.51 |
| IL20 | Interleukin 20 | Protein Coding | GC01P206866 | 2.51 |
| JMJD6 | Jumonji Domain Containing 6, Arginine Demethylase And Lysine Hydroxylase | Protein Coding | GC17M076718 | 2.51 |
| LILRB3 | Leukocyte Immunoglobulin Like Receptor B3 | Protein Coding | GC19M054216 | 2.51 |
| C1QTNF5 | C1q And TNF Related 5 | Protein Coding | GC11M119338 | 2.51 |
| PARP2 | Poly(ADP-Ribose) Polymerase 2 | Protein Coding | GC14P020343 | 2.51 |
| ING1 | Inhibitor Of Growth Family Member 1 | Protein Coding | GC13P110712 | 2.5 |
| PDE1C | Phosphodiesterase 1C | Protein Coding | GC07M031616 | 2.5 |
| NMUR1 | Neuromedin U Receptor 1 | Protein Coding | GC02M231572 | 2.5 |
| FGD3 | FYVE, RhoGEF And PH Domain Containing 3 | Protein Coding | GC09P092947 | 2.5 |
| OVCA2 | OVCA2 Serine Hydrolase Domain Containing | Protein Coding | GC17P002041 | 2.5 |
| ZNF404 | Zinc Finger Protein 404 | Protein Coding | GC19M043872 | 2.5 |
| NAB1 | NGFI-A Binding Protein 1 | Protein Coding | GC02P190646 | 2.5 |
| GPR22 | G Protein-Coupled Receptor 22 | Protein Coding | GC07P107470 | 2.5 |
| CCDC89 | Coiled-Coil Domain Containing 89 | Protein Coding | GC11M085683 | 2.5 |
| ALPI | Alkaline Phosphatase, Intestinal | Protein Coding | GC02P232456 | 2.5 |
| RALYL | RALY RNA Binding Protein Like | Protein Coding | GC08P084182 | 2.49 |
| MSI2 | Musashi RNA Binding Protein 2 | Protein Coding | GC17P057255 | 2.49 |
| WDR76 | WD Repeat Domain 76 | Protein Coding | GC15P043826 | 2.49 |
| PPP1R1A | Protein Phosphatase 1 Regulatory Inhibitor Subunit 1A | Protein Coding | GC12M054576 | 2.49 |
| CAPN9 | Calpain 9 | Protein Coding | GC01P230747 | 2.49 |
| HCG27 | HLA Complex Group 27 | RNA Gene | GC06P031197 | 2.49 |
| MAP6 | Microtubule Associated Protein 6 | Protein Coding | GC11M075586 | 2.49 |
| UIMC1 | Ubiquitin Interaction Motif Containing 1 | Protein Coding | GC05M176905 | 2.49 |
| PSORS1C3 | Psoriasis Susceptibility 1 Candidate 3 | RNA Gene | GC06M031179 | 2.49 |
| GALR1 | Galanin Receptor 1 | Protein Coding | GC18P077250 | 2.49 |
| PROKR1 | Prokineticin Receptor 1 | Protein Coding | GC02P068643 | 2.49 |
| UBXN4 | UBX Domain Protein 4 | Protein Coding | GC02P135741 | 2.48 |
| OARD1 | O-Acyl-ADP-Ribose Deacylase 1 | Protein Coding | GC06M042275 | 2.48 |
| AP2A2 | Adaptor Related Protein Complex 2 Subunit Alpha 2 | Protein Coding | GC11P000915 | 2.48 |
| VTI1A | Vesicle Transport Through Interaction With T-SNAREs 1A | Protein Coding | GC10P112446 | 2.48 |
| QTRT1 | Queuine TRNA-Ribosyltransferase Catalytic Subunit 1 | Protein Coding | GC19P010701 | 2.47 |
| GRK6 | G Protein-Coupled Receptor Kinase 6 | Protein Coding | GC05P177403 | 2.47 |
| COL6A5 | Collagen Type VI Alpha 5 Chain | Protein Coding | GC03P130345 | 2.47 |
| FHL3 | Four And A Half LIM Domains 3 | Protein Coding | GC01M037997 | 2.46 |
| MIR217 | MicroRNA 217 | RNA Gene | GC02M055982 | 2.46 |
| SSR3 | Signal Sequence Receptor Subunit 3 | Protein Coding | GC03M156540 | 2.46 |
| AKR1C4 | Aldo-Keto Reductase Family 1 Member C4 | Protein Coding | GC10P005195 | 2.46 |
| CDC123 | Cell Division Cycle 123 | Protein Coding | GC10P012195 | 2.46 |
| PPHLN1 | Periphilin 1 | Protein Coding | GC12P042238 | 2.46 |
| DNLZ | DNL-Type Zinc Finger | Protein Coding | GC09M136359 | 2.46 |
| PLCB2 | Phospholipase C Beta 2 | Protein Coding | GC15M040278 | 2.46 |
| DDRGK1 | DDRGK Domain Containing 1 | Protein Coding | GC20M003191 | 2.46 |
| MAP9 | Microtubule Associated Protein 9 | Protein Coding | GC04M155342 | 2.46 |
| RF00017-5492 |  | RNA Gene | GC06M043409 | 2.46 |
| EVL | Enah/Vasp-Like | Protein Coding | GC14P099971 | 2.45 |
| LOC111365141 | NOS2 5' Regulatory Region | Biological Region | GC17P027800 | 2.45 |
| LOC101928725 | Uncharacterized LOC101928725 | RNA Gene | GC15M058768 | 2.45 |
| FPR2 | Formyl Peptide Receptor 2 | Protein Coding | GC19P051752 | 2.45 |
| CCR5AS | CCR5 Antisense RNA | RNA Gene | GC03M046365 | 2.45 |
| MIR625 | MicroRNA 625 | RNA Gene | GC14P065471 | 2.45 |
| ACOT1 | Acyl-CoA Thioesterase 1 | Protein Coding | GC14P073493 | 2.45 |
| HSD17B1 | Hydroxysteroid 17-Beta Dehydrogenase 1 | Protein Coding | GC17P042548 | 2.44 |
| SMYD4 | SET And MYND Domain Containing 4 | Protein Coding | GC17M001779 | 2.44 |
| MCF2L | MCF.2 Cell Line Derived Transforming Sequence Like | Protein Coding | GC13P112894 | 2.44 |
| ARHGEF6 | Rac/Cdc42 Guanine Nucleotide Exchange Factor 6 | Protein Coding | GC0XM136665 | 2.44 |
| TFCP2L1 | Transcription Factor CP2 Like 1 | Protein Coding | GC02M121216 | 2.44 |
| MIR98 | MicroRNA 98 | RNA Gene | GC0XM053582 | 2.44 |
| RLN2 | Relaxin 2 | Protein Coding | GC09M005306 | 2.43 |
| PDCD6IP | Programmed Cell Death 6 Interacting Protein | Protein Coding | GC03P033798 | 2.43 |
| DFFB | DNA Fragmentation Factor Subunit Beta | Protein Coding | GC01P003797 | 2.43 |
| SERPINA2 | Serpin Family A Member 2 (Gene/Pseudogene) | Protein Coding | GC14M094404 | 2.43 |
| RPS27P19 | Ribosomal Protein S27 Pseudogene 19 | Pseudogene | GC11M117035 | 2.43 |
| PI16 | Peptidase Inhibitor 16 | Protein Coding | GC06P047467 | 2.43 |
| FBXO3 | F-Box Protein 3 | Protein Coding | GC11M033740 | 2.43 |
| ORM1 | Orosomucoid 1 | Protein Coding | GC09P114323 | 2.43 |
| KIAA1586 | KIAA1586 | Protein Coding | GC06P057046 | 2.43 |
| ZNF788P | Zinc Finger Family Member 788, Pseudogene | Protein Coding | GC19P012270 | 2.43 |
| CBLL1 | Cbl Proto-Oncogene Like 1 | Protein Coding | GC07P107743 | 2.42 |
| ASIC1 | Acid Sensing Ion Channel Subunit 1 | Protein Coding | GC12P050057 | 2.42 |
| CILP2 | Cartilage Intermediate Layer Protein 2 | Protein Coding | GC19P019538 | 2.42 |
| EGR3 | Early Growth Response 3 | Protein Coding | GC08M022687 | 2.42 |
| FUT3 | Fucosyltransferase 3 (Lewis Blood Group) | Protein Coding | GC19M005843 | 2.42 |
| MARCHF8 | Membrane Associated Ring-CH-Type Finger 8 | Protein Coding | GC10M045585 | 2.42 |
| STMND1 | Stathmin Domain Containing 1 | Protein Coding | GC06P017104 | 2.41 |
| EDEM2 | ER Degradation Enhancing Alpha-Mannosidase Like Protein 2 | Protein Coding | GC20M035115 | 2.41 |
| CA10 | Carbonic Anhydrase 10 | Protein Coding | GC17M051630 | 2.41 |
| TUSC1 | Tumor Suppressor Candidate 1 | Protein Coding | GC09M025668 | 2.41 |
| MAD2L1 | Mitotic Arrest Deficient 2 Like 1 | Protein Coding | GC04M120055 | 2.41 |
| ENSG00000244716 |  | Pseudogene | GC01P108993 | 2.41 |
| PIGB | Phosphatidylinositol Glycan Anchor Biosynthesis Class B | Protein Coding | GC15P055318 | 2.41 |
| RF00017-1272 |  | RNA Gene | GC12M111248 | 2.41 |
| SCD5 | Stearoyl-CoA Desaturase 5 | Protein Coding | GC04M082629 | 2.41 |
| PLCB3 | Phospholipase C Beta 3 | Protein Coding | GC11P064251 | 2.41 |
| BLMH | Bleomycin Hydrolase | Protein Coding | GC17M030248 | 2.41 |
| GABPB2 | GA Binding Protein Transcription Factor Subunit Beta 2 | Protein Coding | GC01P151070 | 2.4 |
| FGD5-AS1 | FGD5 Antisense RNA 1 | RNA Gene | GC03M015794 | 2.4 |
| TNFRSF14 | TNF Receptor Superfamily Member 14 | Protein Coding | GC01P002555 | 2.4 |
| IGLL1 | Immunoglobulin Lambda Like Polypeptide 1 | Protein Coding | GC22M023573 | 2.4 |
| EFNA5 | Ephrin A5 | Protein Coding | GC05M107376 | 2.4 |
| DGKQ | Diacylglycerol Kinase Theta | Protein Coding | GC04M000942 | 2.4 |
| NEURL4 | Neuralized E3 Ubiquitin Protein Ligase 4 | Protein Coding | GC17M007315 | 2.4 |
| DHX58 | DExH-Box Helicase 58 | Protein Coding | GC17M042101 | 2.4 |
| CCDC159 | Coiled-Coil Domain Containing 159 | Protein Coding | GC19P011344 | 2.4 |
| SLC7A6 | Solute Carrier Family 7 Member 6 | Protein Coding | GC16P068263 | 2.4 |
| THUMPD2 | THUMP Domain Containing 2 | Protein Coding | GC02M039702 | 2.4 |
| MIR374A | MicroRNA 374a | RNA Gene | GC0XM074296 | 2.4 |
| SRPX | Sushi Repeat Containing Protein X-Linked | Protein Coding | GC0XM038149 | 2.4 |
| CARF | Calcium Responsive Transcription Factor | Protein Coding | GC02P202912 | 2.4 |
| FAM117B | Family With Sequence Similarity 117 Member B | Protein Coding | GC02P202767 | 2.4 |
| ZNF224 | Zinc Finger Protein 224 | Protein Coding | GC19P044094 | 2.4 |
| MACO1 | Macoilin 1 | Protein Coding | GC01P025430 | 2.39 |
| ENSG00000259202 |  | RNA Gene | GC15M067142 | 2.39 |
| TEAD4 | TEA Domain Transcription Factor 4 | Protein Coding | GC12P002959 | 2.39 |
| CD200 | CD200 Molecule | Protein Coding | GC03P112332 | 2.39 |
| HLA-S | Major Histocompatibility Complex, Class I, S (Pseudogene) | Pseudogene | GC06M031381 | 2.39 |
| CORO6 | Coronin 6 | Protein Coding | GC17M029614 | 2.39 |
| GSTA4 | Glutathione S-Transferase Alpha 4 | Protein Coding | GC06M052977 | 2.39 |
| C5orf15 | Chromosome 5 Open Reading Frame 15 | Protein Coding | GC05M133955 | 2.38 |
| NUCB2 | Nucleobindin 2 | Protein Coding | GC11P017221 | 2.38 |
| PLEKHA1 | Pleckstrin Homology Domain Containing A1 | Protein Coding | GC10P122374 | 2.38 |
| RBM5 | RNA Binding Motif Protein 5 | Protein Coding | GC03P050110 | 2.38 |
| CLK3 | CDC Like Kinase 3 | Protein Coding | GC15P074598 | 2.38 |
| SHISA9 | Shisa Family Member 9 | Protein Coding | GC16P012901 | 2.38 |
| SERPINB8 | Serpin Family B Member 8 | Protein Coding | GC18P063969 | 2.38 |
| F2RL2 | Coagulation Factor II Thrombin Receptor Like 2 | Protein Coding | GC05M076615 | 2.38 |
| PRM2 | Protamine 2 | Protein Coding | GC16M011324 | 2.38 |
| CHN2 | Chimerin 2 | Protein Coding | GC07P029186 | 2.38 |
| SNX29 | Sorting Nexin 29 | Protein Coding | GC16P011976 | 2.38 |
| COBLL1 | Cordon-Bleu WH2 Repeat Protein Like 1 | Protein Coding | GC02M164654 | 2.37 |
| ATAD5 | ATPase Family AAA Domain Containing 5 | Protein Coding | GC17P030832 | 2.37 |
| BLOC1S2 | Biogenesis Of Lysosomal Organelles Complex 1 Subunit 2 | Protein Coding | GC10M100273 | 2.37 |
| PLEKHH1 | Pleckstrin Homology, MyTH4 And FERM Domain Containing H1 | Protein Coding | GC14P067533 | 2.37 |
| SUSD2 | Sushi Domain Containing 2 | Protein Coding | GC22P024181 | 2.37 |
| KCTD11 | Potassium Channel Tetramerization Domain Containing 11 | Protein Coding | GC17P007351 | 2.37 |
| PLXDC2 | Plexin Domain Containing 2 | Protein Coding | GC10P019769 | 2.37 |
| F11R | F11 Receptor | Protein Coding | GC01M160995 | 2.37 |
| SPTSSB | Serine Palmitoyltransferase Small Subunit B | Protein Coding | GC03M161344 | 2.37 |
| TEX2 | Testis Expressed 2 | Protein Coding | GC17M064147 | 2.37 |
| SLCO1A2 | Solute Carrier Organic Anion Transporter Family Member 1A2 | Protein Coding | GC12M021264 | 2.37 |
| GNL2 | G Protein Nucleolar 2 | Protein Coding | GC01M037566 | 2.36 |
| ZNF823 | Zinc Finger Protein 823 | Protein Coding | GC19M011721 | 2.36 |
| HTR1F | 5-Hydroxytryptamine Receptor 1F | Protein Coding | GC03P087792 | 2.36 |
| EHBP1L1 | EH Domain Binding Protein 1 Like 1 | Protein Coding | GC11P065814 | 2.36 |
| ALOX15B | Arachidonate 15-Lipoxygenase Type B | Protein Coding | GC17P008039 | 2.36 |
| SNX5 | Sorting Nexin 5 | Protein Coding | GC20M017972 | 2.36 |
| ENSG00000267114 |  | RNA Gene | GC19M044950 | 2.36 |
| AP1G2 | Adaptor Related Protein Complex 1 Subunit Gamma 2 | Protein Coding | GC14M023559 | 2.35 |
| OBP2B | Odorant Binding Protein 2B | Protein Coding | GC09M133205 | 2.35 |
| SERPINB9 | Serpin Family B Member 9 | Protein Coding | GC06M002887 | 2.35 |
| MS4A6A | Membrane Spanning 4-Domains A6A | Protein Coding | GC11M061292 | 2.35 |
| SURF6 | Surfeit 6 | Protein Coding | GC09M133331 | 2.35 |
| DSCR10 | Down Syndrome Critical Region 10 | RNA Gene | GC21P038206 | 2.35 |
| ARHGEF3 | Rho Guanine Nucleotide Exchange Factor 3 | Protein Coding | GC03M056736 | 2.35 |
| REEP5 | Receptor Accessory Protein 5 | Protein Coding | GC05M112876 | 2.35 |
| LATS1 | Large Tumor Suppressor Kinase 1 | Protein Coding | GC06M149658 | 2.35 |
| CCL8 | C-C Motif Chemokine Ligand 8 | Protein Coding | GC17P034319 | 2.35 |
| BCAS3 | BCAS3 Microtubule Associated Cell Migration Factor | Protein Coding | GC17P060678 | 2.34 |
| RNF130 | Ring Finger Protein 130 | Protein Coding | GC05M179911 | 2.34 |
| ACTRT2 | Actin Related Protein T2 | Protein Coding | GC01P003021 | 2.34 |
| STARD7 | StAR Related Lipid Transfer Domain Containing 7 | Protein Coding | GC02M096184 | 2.34 |
| TMEM214 | Transmembrane Protein 214 | Protein Coding | GC02P026997 | 2.34 |
| IFIT5 | Interferon Induced Protein With Tetratricopeptide Repeats 5 | Protein Coding | GC10P089414 | 2.34 |
| ARID3B | AT-Rich Interaction Domain 3B | Protein Coding | GC15P074541 | 2.34 |
| CYP2C18 | Cytochrome P450 Family 2 Subfamily C Member 18 | Protein Coding | GC10P094684 | 2.34 |
| ZNF223 | Zinc Finger Protein 223 | Protein Coding | GC19P044051 | 2.34 |
| ZFYVE9 | Zinc Finger FYVE-Type Containing 9 | Protein Coding | GC01P052142 | 2.33 |
| MATN2 | Matrilin 2 | Protein Coding | GC08P097868 | 2.33 |
| GSTM4 | Glutathione S-Transferase Mu 4 | Protein Coding | GC01P109657 | 2.33 |
| PPID | Peptidylprolyl Isomerase D | Protein Coding | GC04M158709 | 2.33 |
| NOC4L | Nucleolar Complex Associated 4 Homolog | Protein Coding | GC12P132144 | 2.33 |
| JAKMIP1 | Janus Kinase And Microtubule Interacting Protein 1 | Protein Coding | GC04M006026 | 2.33 |
| OLFM4 | Olfactomedin 4 | Protein Coding | GC13P053028 | 2.33 |
| ANXA4 | Annexin A4 | Protein Coding | GC02P069644 | 2.33 |
| PIWIL2 | Piwi Like RNA-Mediated Gene Silencing 2 | Protein Coding | GC08P022275 | 2.33 |
| IFI30 | IFI30 Lysosomal Thiol Reductase | Protein Coding | GC19P018173 | 2.33 |
| IPMK | Inositol Polyphosphate Multikinase | Protein Coding | GC10M058191 | 2.33 |
| UBXN2B | UBX Domain Protein 2B | Protein Coding | GC08P058411 | 2.32 |
| KLF14 | Kruppel Like Factor 14 | Protein Coding | GC07M130731 | 2.32 |
| ENSG00000242798 |  | RNA Gene | GC07M100287 | 2.32 |
| CYP20A1 | Cytochrome P450 Family 20 Subfamily A Member 1 | Protein Coding | GC02P203238 | 2.32 |
| MMP16 | Matrix Metallopeptidase 16 | Protein Coding | GC08M088032 | 2.32 |
| NUCKS1 | Nuclear Casein Kinase And Cyclin Dependent Kinase Substrate 1 | Protein Coding | GC01M205681 | 2.32 |
| EAF1 | ELL Associated Factor 1 | Protein Coding | GC03P015427 | 2.32 |
| CEP128 | Centrosomal Protein 128 | Protein Coding | GC14M080476 | 2.32 |
| MIR16-2 | MicroRNA 16-2 | RNA Gene | GC03P160413 | 2.32 |
| FBXO33 | F-Box Protein 33 | Protein Coding | GC14M039396 | 2.31 |
| HHIPL2 | HHIP Like 2 | Protein Coding | GC01M222794 | 2.31 |
| KCNK13 | Potassium Two Pore Domain Channel Subfamily K Member 13 | Protein Coding | GC14P090061 | 2.31 |
| MACIR | Macrophage Immunometabolism Regulator | Protein Coding | GC05P103259 | 2.31 |
| NMU | Neuromedin U | Protein Coding | GC04M055595 | 2.31 |
| C5orf38 | Chromosome 5 Open Reading Frame 38 | Protein Coding | GC05P002752 | 2.31 |
| FOXB1 | Forkhead Box B1 | Protein Coding | GC15P060004 | 2.31 |
| PSMD6 | Proteasome 26S Subunit, Non-ATPase 6 | Protein Coding | GC03M063973 | 2.31 |
| HOXC13 | Homeobox C13 | Protein Coding | GC12P053938 | 2.31 |
| TANK | TRAF Family Member Associated NFKB Activator | Protein Coding | GC02P161136 | 2.31 |
| UHMK1 | U2AF Homology Motif Kinase 1 | Protein Coding | GC01P162467 | 2.31 |
| RRM2 | Ribonucleotide Reductase Regulatory Subunit M2 | Protein Coding | GC02P010123 | 2.3 |
| ACYP1 | Acylphosphatase 1 | Protein Coding | GC14M075053 | 2.3 |
| UGT1A7 | UDP Glucuronosyltransferase Family 1 Member A7 | Protein Coding | GC02P233681 | 2.3 |
| C3orf62 | Chromosome 3 Open Reading Frame 62 | Protein Coding | GC03M049268 | 2.3 |
| ULK3 | Unc-51 Like Kinase 3 | Protein Coding | GC15M074836 | 2.3 |
| MIR218-1 | MicroRNA 218-1 | RNA Gene | GC04P020677 | 2.3 |
| ATF1 | Activating Transcription Factor 1 | Protein Coding | GC12P050763 | 2.3 |
| E2F3 | E2F Transcription Factor 3 | Protein Coding | GC06P020402 | 2.3 |
| LINC00841 | Long Intergenic Non-Protein Coding RNA 841 | RNA Gene | GC10P043909 | 2.3 |
| PLPP1 | Phospholipid Phosphatase 1 | Protein Coding | GC05M055425 | 2.3 |
| ZBTB33 | Zinc Finger And BTB Domain Containing 33 | Protein Coding | GC0XP120250 | 2.29 |
| GSTA1 | Glutathione S-Transferase Alpha 1 | Protein Coding | GC06M052791 | 2.29 |
| ZNF507 | Zinc Finger Protein 507 | Protein Coding | GC19P032345 | 2.29 |
| MCM7 | Minichromosome Maintenance Complex Component 7 | Protein Coding | GC07M100092 | 2.29 |
| NYAP2 | Neuronal Tyrosine-Phosphorylated Phosphoinositide-3-Kinase Adaptor 2 | Protein Coding | GC02P225400 | 2.29 |
| CPA3 | Carboxypeptidase A3 | Protein Coding | GC03P148865 | 2.29 |
| RGPD8 | RANBP2 Like And GRIP Domain Containing 8 | Protein Coding | GC02M112368 | 2.29 |
| GRB14 | Growth Factor Receptor Bound Protein 14 | Protein Coding | GC02M164492 | 2.29 |
| C19orf38 | Chromosome 19 Open Reading Frame 38 | Protein Coding | GC19P010837 | 2.29 |
| ZNF284 | Zinc Finger Protein 284 | Protein Coding | GC19P044072 | 2.29 |
| DHX8 | DEAH-Box Helicase 8 | Protein Coding | GC17P043483 | 2.29 |
| CISD1 | CDGSH Iron Sulfur Domain 1 | Protein Coding | GC10P058269 | 2.29 |
| WFDC1 | WAP Four-Disulfide Core Domain 1 | Protein Coding | GC16P084328 | 2.29 |
| EZH1 | Enhancer Of Zeste 1 Polycomb Repressive Complex 2 Subunit | Protein Coding | GC17M042700 | 2.29 |
| COL16A1 | Collagen Type XVI Alpha 1 Chain | Protein Coding | GC01M031653 | 2.28 |
| CDK18 | Cyclin Dependent Kinase 18 | Protein Coding | GC01P205504 | 2.28 |
| TRIM69 | Tripartite Motif Containing 69 | Protein Coding | GC15P044729 | 2.28 |
| ENSG00000284686 |  | Protein Coding | GC01M056173 | 2.28 |
| LRTM1 | Leucine Rich Repeats And Transmembrane Domains 1 | Protein Coding | GC03M054928 | 2.28 |
| SH3BP5 | SH3 Domain Binding Protein 5 | Protein Coding | GC03M015805 | 2.28 |
| LTBR | Lymphotoxin Beta Receptor | Protein Coding | GC12P006375 | 2.28 |
| MMP26 | Matrix Metallopeptidase 26 | Protein Coding | GC11P004706 | 2.28 |
| TSPAN16 | Tetraspanin 16 | Protein Coding | GC19P011296 | 2.28 |
| PSORS1C1 | Psoriasis Susceptibility 1 Candidate 1 | Protein Coding | GC06P031114 | 2.27 |
| MALL | Mal, T Cell Differentiation Protein Like | Protein Coding | GC02M110083 | 2.27 |
| ICA1L | Islet Cell Autoantigen 1 Like | Protein Coding | GC02M202797 | 2.27 |
| NOL7 | Nucleolar Protein 7 | Protein Coding | GC06P013615 | 2.27 |
| SLC14A2 | Solute Carrier Family 14 Member 2 | Protein Coding | GC18P045212 | 2.27 |
| HEMGN | Hemogen | Protein Coding | GC09M097927 | 2.27 |
| SMURF1 | SMAD Specific E3 Ubiquitin Protein Ligase 1 | Protein Coding | GC07M099027 | 2.27 |
| RYK | Receptor Like Tyrosine Kinase | Protein Coding | GC03M134065 | 2.27 |
| LILRB1 | Leukocyte Immunoglobulin Like Receptor B1 | Protein Coding | GC19P055408 | 2.27 |
| ELK1 | ETS Transcription Factor ELK1 | Protein Coding | GC0XM047635 | 2.26 |
| RSRC2 | Arginine And Serine Rich Coiled-Coil 2 | Protein Coding | GC12M122503 | 2.26 |
| IL17D | Interleukin 17D | Protein Coding | GC13P020702 | 2.26 |
| IQCA1 | IQ Motif Containing With AAA Domain 1 | Protein Coding | GC02M236324 | 2.26 |
| ANKRD22 | Ankyrin Repeat Domain 22 | Protein Coding | GC10M088819 | 2.26 |
| CLEC5A | C-Type Lectin Domain Containing 5A | Protein Coding | GC07M141927 | 2.26 |
| CMTR1 | Cap Methyltransferase 1 | Protein Coding | GC06P047474 | 2.26 |
| TFPI2 | Tissue Factor Pathway Inhibitor 2 | Protein Coding | GC07M093885 | 2.26 |
| SUPT3H | SPT3 Homolog, SAGA And STAGA Complex Component | Protein Coding | GC06M044807 | 2.25 |
| ADGRE5 | Adhesion G Protein-Coupled Receptor E5 | Protein Coding | GC19P014381 | 2.25 |
| MIR202 | MicroRNA 202 | RNA Gene | GC10M133247 | 2.25 |
| ADSS2 | Adenylosuccinate Synthase 2 | Protein Coding | GC01M244410 | 2.25 |
| POLR2D | RNA Polymerase II Subunit D | Protein Coding | GC02M127981 | 2.25 |
| ARHGAP1 | Rho GTPase Activating Protein 1 | Protein Coding | GC11M061106 | 2.25 |
| ARHGAP42 | Rho GTPase Activating Protein 42 | Protein Coding | GC11P100687 | 2.25 |
| SLC23A2 | Solute Carrier Family 23 Member 2 | Protein Coding | GC20M004852 | 2.25 |
| LCMT2 | Leucine Carboxyl Methyltransferase 2 | Protein Coding | GC15M043323 | 2.25 |
| TOR2A | Torsin Family 2 Member A | Protein Coding | GC09M127732 | 2.25 |
| DEGS2 | Delta 4-Desaturase, Sphingolipid 2 | Protein Coding | GC14M100143 | 2.25 |
| NCF1C | Neutrophil Cytosolic Factor 1C Pseudogene | Pseudogene | GC07M075156 | 2.24 |
| PAX8-AS1 | PAX8 Antisense RNA 1 | RNA Gene | GC02P113211 | 2.24 |
| LOC107984189 | Uncharacterized Protein C10orf142-Like | Protein Coding | GC10M043825 | 2.24 |
| LOC107984814 | Uncharacterized LOC107984814 | Protein Coding | GC16P072664 | 2.24 |
| ANAPC1 | Anaphase Promoting Complex Subunit 1 | Protein Coding | GC02M111611 | 2.24 |
| DDX25 | DEAD-Box Helicase 25 | Protein Coding | GC11P125903 | 2.24 |
| ARHGEF26 | Rho Guanine Nucleotide Exchange Factor 26 | Protein Coding | GC03P154124 | 2.24 |
| LOC100996842 | Uncharacterized LOC100996842 | Protein Coding | GC17M007583 | 2.24 |
| STK38 | Serine/Threonine Kinase 38 | Protein Coding | GC06M036493 | 2.24 |
| SNX1 | Sorting Nexin 1 | Protein Coding | GC15P064094 | 2.24 |
| VWA7 | Von Willebrand Factor A Domain Containing 7 | Protein Coding | GC06M032622 | 2.24 |
| UGT1A9 | UDP Glucuronosyltransferase Family 1 Member A9 | Protein Coding | GC02P233671 | 2.24 |
| RANGAP1 | Ran GTPase Activating Protein 1 | Protein Coding | GC22M041244 | 2.24 |
| NSUN4 | NOP2/Sun RNA Methyltransferase 4 | Protein Coding | GC01P046341 | 2.23 |
| NID2 | Nidogen 2 | Protein Coding | GC14M052004 | 2.23 |
| RNF20 | Ring Finger Protein 20 | Protein Coding | GC09P101533 | 2.23 |
| TSPAN8 | Tetraspanin 8 | Protein Coding | GC12M071125 | 2.23 |
| FGFBP2 | Fibroblast Growth Factor Binding Protein 2 | Protein Coding | GC04M015961 | 2.23 |
| RANBP3L | RAN Binding Protein 3 Like | Protein Coding | GC05M036286 | 2.23 |
| INTS11 | Integrator Complex Subunit 11 | Protein Coding | GC01M001375 | 2.23 |
| SSH1 | Slingshot Protein Phosphatase 1 | Protein Coding | GC12M108784 | 2.23 |
| PCOLCE | Procollagen C-Endopeptidase Enhancer | Protein Coding | GC07P100602 | 2.23 |
| NRIP3 | Nuclear Receptor Interacting Protein 3 | Protein Coding | GC11M009001 | 2.22 |
| GTF3C2 | General Transcription Factor IIIC Subunit 2 | Protein Coding | GC02M027325 | 2.22 |
| TTC39B | Tetratricopeptide Repeat Domain 39B | Protein Coding | GC09M015170 | 2.22 |
| HDGFL1 | HDGF Like 1 | Protein Coding | GC06P022569 | 2.21 |
| PLEKHO2 | Pleckstrin Homology Domain Containing O2 | Protein Coding | GC15P073903 | 2.21 |
| VAMP3 | Vesicle Associated Membrane Protein 3 | Protein Coding | GC01P007765 | 2.21 |
| MS | Multiple Sclerosis | Genetic Locus | GC00U990205 | 2.21 |
| TTC29 | Tetratricopeptide Repeat Domain 29 | Protein Coding | GC04M146706 | 2.21 |
| SCAMP5 | Secretory Carrier Membrane Protein 5 | Protein Coding | GC15P074957 | 2.21 |
| MIR497 | MicroRNA 497 | RNA Gene | GC17M007022 | 2.21 |
| ABCA8 | ATP Binding Cassette Subfamily A Member 8 | Protein Coding | GC17M068867 | 2.2 |
| MYO3B | Myosin IIIB | Protein Coding | GC02P170178 | 2.2 |
| ENSG00000253775 |  | RNA Gene | GC08P020079 | 2.2 |
| PCNPP1 | PEST Containing Nuclear Protein Pseudogene 1 | Pseudogene | GC12M111666 | 2.2 |
| ITM2A | Integral Membrane Protein 2A | Protein Coding | GC0XM079360 | 2.2 |
| CASTOR3 | CASTOR Family Member 3 | Pseudogene | GC07M100290 | 2.2 |
| MSL2 | MSL Complex Subunit 2 | Protein Coding | GC03M136149 | 2.2 |
| LYSMD4 | LysM Domain Containing 4 | Protein Coding | GC15M103921 | 2.2 |
| SAMSN1 | SAM Domain, SH3 Domain And Nuclear Localization Signals 1 | Protein Coding | GC21M014485 | 2.2 |
| DDX60L | DExD/H-Box 60 Like | Protein Coding | GC04M168356 | 2.2 |
| PIAS4 | Protein Inhibitor Of Activated STAT 4 | Protein Coding | GC19P004007 | 2.2 |
| SRGN | Serglycin | Protein Coding | GC10P069088 | 2.2 |
| ZNF112 | Zinc Finger Protein 112 | Protein Coding | GC19M044327 | 2.19 |
| GPR17 | G Protein-Coupled Receptor 17 | Protein Coding | GC02P127645 | 2.19 |
| GIMAP6 | GTPase, IMAP Family Member 6 | Protein Coding | GC07M150625 | 2.19 |
| SEC31B | SEC31 Homolog B, COPII Coat Complex Component | Protein Coding | GC10M100486 | 2.19 |
| KNTC1 | Kinetochore Associated 1 | Protein Coding | GC12P122527 | 2.19 |
| ANP32B | Acidic Nuclear Phosphoprotein 32 Family Member B | Protein Coding | GC09P097983 | 2.19 |
| TDRD15 | Tudor Domain Containing 15 | Protein Coding | GC02P021123 | 2.19 |
| C4BPB | Complement Component 4 Binding Protein Beta | Protein Coding | GC01P207088 | 2.19 |
| AKTIP | AKT Interacting Protein | Protein Coding | GC16M053492 | 2.19 |
| CWF19L2 | CWF19 Like Cell Cycle Control Factor 2 | Protein Coding | GC11M107326 | 2.19 |
| CPM | Carboxypeptidase M | Protein Coding | GC12M068842 | 2.18 |
| HGFAC | HGF Activator | Protein Coding | GC04P003443 | 2.18 |
| SLC25A35 | Solute Carrier Family 25 Member 35 | Protein Coding | GC17M008287 | 2.18 |
| ADGRL3 | Adhesion G Protein-Coupled Receptor L3 | Protein Coding | GC04P061201 | 2.18 |
| POLN | DNA Polymerase Nu | Protein Coding | GC04M002073 | 2.18 |
| STEAP1 | STEAP Family Member 1 | Protein Coding | GC07P090154 | 2.17 |
| IGFALS | Insulin Like Growth Factor Binding Protein Acid Labile Subunit | Protein Coding | GC16M001790 | 2.17 |
| SPECC1L-ADORA2A | SPECC1L-ADORA2A Readthrough (NMD Candidate) | RNA Gene | GC22P024836 | 2.17 |
| GOLPH3L | Golgi Phosphoprotein 3 Like | Protein Coding | GC01M150711 | 2.17 |
| THBS3 | Thrombospondin 3 | Protein Coding | GC01M155195 | 2.17 |
| GABRG3 | Gamma-Aminobutyric Acid Type A Receptor Subunit Gamma3 | Protein Coding | GC15P026971 | 2.17 |
| ANAPC4 | Anaphase Promoting Complex Subunit 4 | Protein Coding | GC04P025379 | 2.17 |
| EHD3 | EH Domain Containing 3 | Protein Coding | GC02P031234 | 2.17 |
| VPS41 | VPS41 Subunit Of HOPS Complex | Protein Coding | GC07M038730 | 2.17 |
| LINC01589 | Long Intergenic Non-Protein Coding RNA 1589 | RNA Gene | GC22M045687 | 2.16 |
| TRIM4 | Tripartite Motif Containing 4 | Protein Coding | GC07M099876 | 2.16 |
| MIR501 | MicroRNA 501 | RNA Gene | GC0XP050074 | 2.16 |
| ZNF385B | Zinc Finger Protein 385B | Protein Coding | GC02M179441 | 2.16 |
| MEGF6 | Multiple EGF Like Domains 6 | Protein Coding | GC01M003487 | 2.16 |
| ENSG00000251405 |  | RNA Gene | GC05M157363 | 2.16 |
| SNORD10 | Small Nucleolar RNA, C/D Box 10 | RNA Gene | GC17P008044 | 2.16 |
| SHE | Src Homology 2 Domain Containing E | Protein Coding | GC01M154442 | 2.16 |
| YIPF5 | Yip1 Domain Family Member 5 | Protein Coding | GC05M144158 | 2.16 |
| GRID1 | Glutamate Ionotropic Receptor Delta Type Subunit 1 | Protein Coding | GC10M085599 | 2.15 |
| PEBP4 | Phosphatidylethanolamine Binding Protein 4 | Protein Coding | GC08M022713 | 2.15 |
| PCDH9 | Protocadherin 9 | Protein Coding | GC13M066302 | 2.15 |
| SEMA4G | Semaphorin 4G | Protein Coding | GC10P100969 | 2.15 |
| ENSG00000272501 |  | RNA Gene | GC06M031195 | 2.15 |
| E2F6 | E2F Transcription Factor 6 | Protein Coding | GC02M011434 | 2.15 |
| NPY5R | Neuropeptide Y Receptor Y5 | Protein Coding | GC04P163343 | 2.15 |
| MIR411 | MicroRNA 411 | RNA Gene | GC14P104808 | 2.15 |
| SEC16A | SEC16 Homolog A, Endoplasmic Reticulum Export Factor | Protein Coding | GC09M136440 | 2.15 |
| ARL5B | ADP Ribosylation Factor Like GTPase 5B | Protein Coding | GC10P018659 | 2.15 |
| FLG-AS1 | FLG Antisense RNA 1 | RNA Gene | GC01P152168 | 2.15 |
| SLK | STE20 Like Kinase | Protein Coding | GC10P103967 | 2.15 |
| PINX1 | PIN2 (TERF1) Interacting Telomerase Inhibitor 1 | Protein Coding | GC08M010726 | 2.14 |
| NIPSNAP3B | Nipsnap Homolog 3B | Protein Coding | GC09P104763 | 2.14 |
| ESYT3 | Extended Synaptotagmin 3 | Protein Coding | GC03P138434 | 2.14 |
| MYH4 | Myosin Heavy Chain 4 | Protein Coding | GC17M010443 | 2.14 |
| TTC32 | Tetratricopeptide Repeat Domain 32 | Protein Coding | GC02M019896 | 2.14 |
| CCDC158 | Coiled-Coil Domain Containing 158 | Protein Coding | GC04M076312 | 2.14 |
| RPRD2 | Regulation Of Nuclear Pre-MRNA Domain Containing 2 | Protein Coding | GC01P150363 | 2.14 |
| APOL4 | Apolipoprotein L4 | Protein Coding | GC22M036190 | 2.14 |
| MIR29B2 | MicroRNA 29b-2 | RNA Gene | GC01M207806 | 2.13 |
| SNORA48 | Small Nucleolar RNA, H/ACA Box 48 | RNA Gene | GC17P008045 | 2.13 |
| ENSG00000265749 |  | RNA Gene | GC17P008365 | 2.13 |
| GRIK4 | Glutamate Ionotropic Receptor Kainate Type Subunit 4 | Protein Coding | GC11P120480 | 2.13 |
| GSTM5 | Glutathione S-Transferase Mu 5 | Protein Coding | GC01P109711 | 2.13 |
| ABHD2 | Abhydrolase Domain Containing 2, Acylglycerol Lipase | Protein Coding | GC15P089087 | 2.13 |
| FOXS1 | Forkhead Box S1 | Protein Coding | GC20M031844 | 2.13 |
| NCKAP5 | NCK Associated Protein 5 | Protein Coding | GC02M132671 | 2.13 |
| SMAD5 | SMAD Family Member 5 | Protein Coding | GC05P136132 | 2.13 |
| piR-51327 |  | RNA Gene | GC12M111437 | 2.13 |
| lnc-FAM109A-1 |  | RNA Gene | GC12M111463 | 2.13 |
| piR-56480-015 |  | RNA Gene | GC12M111439 | 2.13 |
| piR-51449 |  | RNA Gene | GC12P111448 | 2.13 |
| piR-50346 |  | RNA Gene | GC12M111466 | 2.13 |
| piR-36455 |  | RNA Gene | GC12M111444 | 2.13 |
| piR-38259 |  | RNA Gene | GC12M111462 | 2.13 |
| piR-48007 |  | RNA Gene | GC12M111465 | 2.13 |
| FBXO15 | F-Box Protein 15 | Protein Coding | GC18M074073 | 2.13 |
| MAPK13 | Mitogen-Activated Protein Kinase 13 | Protein Coding | GC06P047452 | 2.13 |
| LINC02881 | Long Intergenic Non-Protein Coding RNA 2881 | RNA Gene | GC10P044293 | 2.13 |
| LINC-ROR | Long Intergenic Non-Protein Coding RNA, Regulator Of Reprogramming | RNA Gene | GC18M057054 | 2.12 |
| LRRC2 | Leucine Rich Repeat Containing 2 | Protein Coding | GC03M046515 | 2.12 |
| ACVR1B | Activin A Receptor Type 1B | Protein Coding | GC12P051951 | 2.12 |
| ADAMTS14 | ADAM Metallopeptidase With Thrombospondin Type 1 Motif 14 | Protein Coding | GC10P070672 | 2.12 |
| ZNF887P | Zinc Finger Protein 887, Pseudogene | Pseudogene | GC19P011652 | 2.12 |
| ANKRD28 | Ankyrin Repeat Domain 28 | Protein Coding | GC03M015667 | 2.11 |
| LIPC-AS1 | LIPC Antisense RNA 1 | RNA Gene | GC15M060616 | 2.11 |
| KLK11 | Kallikrein Related Peptidase 11 | Protein Coding | GC19M051023 | 2.11 |
| SLC8A2 | Solute Carrier Family 8 Member A2 | Protein Coding | GC19M047428 | 2.11 |
| STXBP5 | Syntaxin Binding Protein 5 | Protein Coding | GC06P147204 | 2.11 |
| SFRP5 | Secreted Frizzled Related Protein 5 | Protein Coding | GC10M097766 | 2.11 |
| LINC01089 | Long Intergenic Non-Protein Coding RNA 1089 | RNA Gene | GC12M121796 | 2.11 |
| DUXA | Double Homeobox A | Protein Coding | GC19M057152 | 2.11 |
| DIO1 | Iodothyronine Deiodinase 1 | Protein Coding | GC01P053891 | 2.1 |
| MAGEC2 | MAGE Family Member C2 | Protein Coding | GC0XM142202 | 2.1 |
| GTF3C2-AS1 | GTF3C2 Antisense RNA 1 | RNA Gene | GC02P027336 | 2.1 |
| LINC00472 | Long Intergenic Non-Protein Coding RNA 472 | RNA Gene | GC06M071344 | 2.1 |
| METRNL | Meteorin Like, Glial Cell Differentiation Regulator | Protein Coding | GC17P083079 | 2.1 |
| SELENOM | Selenoprotein M | Protein Coding | GC22M031105 | 2.1 |
| MAGI1 | Membrane Associated Guanylate Kinase, WW And PDZ Domain Containing 1 | Protein Coding | GC03M065330 | 2.1 |
| TREH | Trehalase | Protein Coding | GC11M118657 | 2.1 |
| GCOM1 | GRINL1A Complex Locus 1 | Protein Coding | GC15P057591 | 2.1 |
| STAB2 | Stabilin 2 | Protein Coding | GC12P103587 | 2.1 |
| UBE2D3P3 | Ubiquitin Conjugating Enzyme E2 D3 Pseudogene 3 | Pseudogene | GC01P150800 | 2.1 |
| CXCL14 | C-X-C Motif Chemokine Ligand 14 | Protein Coding | GC05M135617 | 2.1 |
| SLC44A5 | Solute Carrier Family 44 Member 5 | Protein Coding | GC01M075202 | 2.1 |
| UGT1A4 | UDP Glucuronosyltransferase Family 1 Member A4 | Protein Coding | GC02P233718 | 2.1 |
| IRAG1 | Inositol 1,4,5-Triphosphate Receptor Associated 1 | Protein Coding | GC11M010656 | 2.1 |
| MIR26A2 | MicroRNA 26a-2 | RNA Gene | GC12M057824 | 2.1 |
| INTS8 | Integrator Complex Subunit 8 | Protein Coding | GC08P094813 | 2.09 |
| IGF2BP1 | Insulin Like Growth Factor 2 MRNA Binding Protein 1 | Protein Coding | GC17P048997 | 2.09 |
| ENSG00000234268 |  | Pseudogene | GC11M117036 | 2.09 |
| MIR454 | MicroRNA 454 | RNA Gene | GC17M059137 | 2.09 |
| GIT1 | GIT ArfGAP 1 | Protein Coding | GC17M029573 | 2.09 |
| STOML1 | Stomatin Like 1 | Protein Coding | GC15M073978 | 2.09 |
| CNPY4 | Canopy FGF Signaling Regulator 4 | Protein Coding | GC07P100119 | 2.09 |
| RNF139-AS1 | RNF139 Antisense RNA 1 (Head To Head) | RNA Gene | GC08M124462 | 2.09 |
| CLEC11A | C-Type Lectin Domain Containing 11A | Protein Coding | GC19P050723 | 2.09 |
| RPL18AP13 | Ribosomal Protein L18a Pseudogene 13 | Pseudogene | GC19P011521 | 2.08 |
| WASHC5-AS1 | WASHC5 Antisense RNA 1 | RNA Gene | GC08P125062 | 2.08 |
| P2RX6 | Purinergic Receptor P2X 6 | Protein Coding | GC22P021015 | 2.08 |
| CPED1 | Cadherin Like And PC-Esterase Domain Containing 1 | Protein Coding | GC07P120988 | 2.08 |
| SBSPON | Somatomedin B And Thrombospondin Type 1 Domain Containing | Protein Coding | GC08M073064 | 2.08 |
| CXCL3 | C-X-C Motif Chemokine Ligand 3 | Protein Coding | GC04M074036 | 2.08 |
| MFAP1 | Microfibril Associated Protein 1 | Protein Coding | GC15M043804 | 2.08 |
| PRELID1 | PRELI Domain Containing 1 | Protein Coding | GC05P177305 | 2.08 |
| ABCA2 | ATP Binding Cassette Subfamily A Member 2 | Protein Coding | GC09M137007 | 2.08 |
| HMGB2 | High Mobility Group Box 2 | Protein Coding | GC04M173331 | 2.07 |
| ZBTB14 | Zinc Finger And BTB Domain Containing 14 | Protein Coding | GC18M005289 | 2.07 |
| VTA1 | Vesicle Trafficking 1 | Protein Coding | GC06P142147 | 2.07 |
| GOLGA6L3 | Golgin A6 Family Like 3 | Protein Coding | GC15P085240 | 2.07 |
| ENSG00000226849 |  | RNA Gene | GC01P011068 | 2.07 |
| CDH9 | Cadherin 9 | Protein Coding | GC05M026916 | 2.07 |
| TRHDE | Thyrotropin Releasing Hormone Degrading Enzyme | Protein Coding | GC12P072087 | 2.07 |
| ENSG00000272657 |  | RNA Gene | GC21P034073 | 2.07 |
| ADH5 | Alcohol Dehydrogenase 5 (Class III), Chi Polypeptide | Protein Coding | GC04M099070 | 2.07 |
| MLXIP | MLX Interacting Protein | Protein Coding | GC12P122078 | 2.07 |
| IFIT1B | Interferon Induced Protein With Tetratricopeptide Repeats 1B | Protein Coding | GC10P089378 | 2.06 |
| KRTCAP3 | Keratinocyte Associated Protein 3 | Protein Coding | GC02P027442 | 2.06 |
| ZNF225 | Zinc Finger Protein 225 | Protein Coding | GC19P044112 | 2.06 |
| ENSG00000253106 |  | RNA Gene | GC08P124488 | 2.06 |
| RPN1 | Ribophorin I | Protein Coding | GC03M128619 | 2.06 |
| P2RY4 | Pyrimidinergic Receptor P2Y4 | Protein Coding | GC0XM070258 | 2.06 |
| PDLIM7 | PDZ And LIM Domain 7 | Protein Coding | GC05M177483 | 2.06 |
| FAM13C | Family With Sequence Similarity 13 Member C | Protein Coding | GC10M059246 | 2.06 |
| ZNF227 | Zinc Finger Protein 227 | Protein Coding | GC19P044207 | 2.06 |
| CHST9 | Carbohydrate Sulfotransferase 9 | Protein Coding | GC18M026906 | 2.06 |
| BCO1 | Beta-Carotene Oxygenase 1 | Protein Coding | GC16P081238 | 2.05 |
| KRTCAP2 | Keratinocyte Associated Protein 2 | Protein Coding | GC01M155141 | 2.05 |
| C10orf71 | Chromosome 10 Open Reading Frame 71 | Protein Coding | GC10P049297 | 2.05 |
| ENSG00000264545 |  | Protein Coding | GC09P021802 | 2.05 |
| ZNF235 | Zinc Finger Protein 235 | Protein Coding | GC19M044228 | 2.05 |
| USP42 | Ubiquitin Specific Peptidase 42 | Protein Coding | GC07P006078 | 2.05 |
| RASSF10 | Ras Association Domain Family Member 10 | Protein Coding | GC11P012990 | 2.05 |
| UBE2G1 | Ubiquitin Conjugating Enzyme E2 G1 | Protein Coding | GC17M004270 | 2.05 |
| CAP1 | Cyclase Associated Actin Cytoskeleton Regulatory Protein 1 | Protein Coding | GC01P040041 | 2.05 |
| NME2 | NME/NM23 Nucleoside Diphosphate Kinase 2 | Protein Coding | GC17P051165 | 2.05 |
| GTF2B | General Transcription Factor IIB | Protein Coding | GC01M088853 | 2.04 |
| KCTD12 | Potassium Channel Tetramerization Domain Containing 12 | Protein Coding | GC13M076880 | 2.04 |
| GRK3 | G Protein-Coupled Receptor Kinase 3 | Protein Coding | GC22P025565 | 2.04 |
| ENSG00000214955 |  | RNA Gene | GC21P034205 | 2.04 |
| lnc-JCAD-2 |  | RNA Gene | GC10M030507 | 2.04 |
| KCNK10 | Potassium Two Pore Domain Channel Subfamily K Member 10 | Protein Coding | GC14M088180 | 2.04 |
| DENND2B | DENN Domain Containing 2B | Protein Coding | GC11M008695 | 2.04 |
| SLC29A4 | Solute Carrier Family 29 Member 4 | Protein Coding | GC07P005289 | 2.03 |
| TSC22D1 | TSC22 Domain Family Member 1 | Protein Coding | GC13M044432 | 2.03 |
| COL6A4P1 | Collagen Type VI Alpha 4 Pseudogene 1 | Pseudogene | GC03M015151 | 2.03 |
| P2RY6 | Pyrimidinergic Receptor P2Y6 | Protein Coding | GC11P073264 | 2.03 |
| ENSG00000253111 |  | RNA Gene | GC08P125466 | 2.03 |
| RNF182 | Ring Finger Protein 182 | Protein Coding | GC06P013924 | 2.02 |
| RPL7P8 | Ribosomal Protein L7 Pseudogene 8 | Pseudogene | GC01P109651 | 2.02 |
| PAFAH1B2 | Platelet Activating Factor Acetylhydrolase 1b Catalytic Subunit 2 | Protein Coding | GC11P117144 | 2.02 |
| MN298114-200 |  | RNA Gene | GC06M031041 | 2.02 |
| ZNF300 | Zinc Finger Protein 300 | Protein Coding | GC05M150894 | 2.02 |
| MAT2B | Methionine Adenosyltransferase 2B | Protein Coding | GC05P163523 | 2.02 |
| PLA2G2D | Phospholipase A2 Group IID | Protein Coding | GC01M020111 | 2.02 |
| TM7SF3 | Transmembrane 7 Superfamily Member 3 | Protein Coding | GC12M026971 | 2.02 |
| OSBPL3 | Oxysterol Binding Protein Like 3 | Protein Coding | GC07M024836 | 2.02 |
| AASDH | Aminoadipate-Semialdehyde Dehydrogenase | Protein Coding | GC04M056340 | 2.02 |
| ZNF230 | Zinc Finger Protein 230 | Protein Coding | GC19P044002 | 2.02 |
| MOGAT2 | Monoacylglycerol O-Acyltransferase 2 | Protein Coding | GC11P075717 | 2.02 |
| CTR9 | CTR9 Homolog, Paf1/RNA Polymerase II Complex Component | Protein Coding | GC11P010772 | 2.01 |
| NFKBIE | NFKB Inhibitor Epsilon | Protein Coding | GC06M044258 | 2.01 |
| CD160 | CD160 Molecule | Protein Coding | GC01P145719 | 2.01 |
| CCDC71L | Coiled-Coil Domain Containing 71 Like | Protein Coding | GC07M106656 | 2.01 |
| HSALNG0099364 |  | RNA Gene | GC13M110312 | 2.01 |
| SYT9 | Synaptotagmin 9 | Protein Coding | GC11P007238 | 2.01 |
| CACFD1 | Calcium Channel Flower Domain Containing 1 | Protein Coding | GC09P133459 | 2.01 |
| ENSG00000248734 |  | RNA Gene | GC05P096785 | 2.01 |
| ACAA2 | Acetyl-CoA Acyltransferase 2 | Protein Coding | GC18M049782 | 2.01 |
| ZNF558 | Zinc Finger Protein 558 | Protein Coding | GC19M008806 | 2 |
| SNHG3 | Small Nucleolar RNA Host Gene 3 | RNA Gene | GC01P028506 | 2 |
| DNAJC5B | DnaJ Heat Shock Protein Family (Hsp40) Member C5 Beta | Protein Coding | GC08P066021 | 2 |
| MAS1 | MAS1 Proto-Oncogene, G Protein-Coupled Receptor | Protein Coding | GC06P159906 | 2 |
| GUSBP4 | GUSB Pseudogene 4 | Pseudogene | GC06M057910 | 2 |
| TINAGL1 | Tubulointerstitial Nephritis Antigen Like 1 | Protein Coding | GC01P031576 | 2 |
| RRBP1 | Ribosome Binding Protein 1 | Protein Coding | GC20M017613 | 2 |
| PPFIA1 | PTPRF Interacting Protein Alpha 1 | Protein Coding | GC11P070270 | 2 |
| ZSCAN29 | Zinc Finger And SCAN Domain Containing 29 | Protein Coding | GC15M043358 | 2 |
| HAPLN3 | Hyaluronan And Proteoglycan Link Protein 3 | Protein Coding | GC15M088877 | 2 |
| UBE2Q2P1 | Ubiquitin Conjugating Enzyme E2 Q2 Pseudogene 1 | Pseudogene | GC15M084526 | 2 |
| GTPBP10 | GTP Binding Protein 10 | Protein Coding | GC07P090336 | 2 |
| ZNF226 | Zinc Finger Protein 226 | Protein Coding | GC19P044165 | 2 |
| ISLR | Immunoglobulin Superfamily Containing Leucine Rich Repeat | Protein Coding | GC15P074173 | 1.99 |
| XKR6 | XK Related 6 | Protein Coding | GC08M010896 | 1.99 |
| FCRL6 | Fc Receptor Like 6 | Protein Coding | GC01P159800 | 1.99 |
| CBLC | Cbl Proto-Oncogene C | Protein Coding | GC19P044777 | 1.99 |
| KBTBD11 | Kelch Repeat And BTB Domain Containing 11 | Protein Coding | GC08P001973 | 1.99 |
| ENSG00000262089 |  | RNA Gene | GC17M006994 | 1.99 |
| NPFF | Neuropeptide FF-Amide Peptide Precursor | Protein Coding | GC12M053516 | 1.99 |
| BMERB1 | BMERB Domain Containing 1 | Protein Coding | GC16P015434 | 1.99 |
| ABCC10 | ATP Binding Cassette Subfamily C Member 10 | Protein Coding | GC06P043427 | 1.99 |
| FRY | FRY Microtubule Binding Protein | Protein Coding | GC13P031852 | 1.99 |
| ANKRD65 | Ankyrin Repeat Domain 65 | Protein Coding | GC01M001418 | 1.99 |
| CDC25B | Cell Division Cycle 25B | Protein Coding | GC20P003787 | 1.99 |
| CBWD2 | COBW Domain Containing 2 | Protein Coding | GC02P115309 | 1.98 |
| CBR4 | Carbonyl Reductase 4 | Protein Coding | GC04M168864 | 1.98 |
| LOC107987125 | Uncharacterized LOC107987125 | Protein Coding | GC09P121810 | 1.98 |
| MIR493 | MicroRNA 493 | RNA Gene | GC14P104813 | 1.98 |
| PHACTR4 | Phosphatase And Actin Regulator 4 | Protein Coding | GC01P028369 | 1.98 |
| NONHSAG017238.2 |  | RNA Gene | GC15P073144 | 1.98 |
| lnc-APOC1-1 |  | RNA Gene | GC19P044910 | 1.98 |
| MN298678-088 |  | RNA Gene | GC06M133858 | 1.98 |
| ST6GAL1 | ST6 Beta-Galactoside Alpha-2,6-Sialyltransferase 1 | Protein Coding | GC03P186930 | 1.97 |
| SCML4 | Scm Polycomb Group Protein Like 4 | Protein Coding | GC06M107701 | 1.97 |
| ZNF155 | Zinc Finger Protein 155 | Protein Coding | GC19P043967 | 1.97 |
| UGT1A | UDP Glucuronosyltransferase Family 1 Member A Complex Locus | Uncategorized | GC02P233586 | 1.97 |
| LARP6 | La Ribonucleoprotein 6, Translational Regulator | Protein Coding | GC15M070829 | 1.97 |
| FHOD1 | Formin Homology 2 Domain Containing 1 | Protein Coding | GC16M067230 | 1.97 |
| ZNF665 | Zinc Finger Protein 665 | Protein Coding | GC19M053159 | 1.97 |
| CLEC14A | C-Type Lectin Domain Containing 14A | Protein Coding | GC14M038254 | 1.97 |
| CGREF1 | Cell Growth Regulator With EF-Hand Domain 1 | Protein Coding | GC02M027098 | 1.97 |
| CCNL1 | Cyclin L1 | Protein Coding | GC03M157146 | 1.97 |
| PF4V1 | Platelet Factor 4 Variant 1 | Protein Coding | GC04P073853 | 1.97 |
| KCNH5 | Potassium Voltage-Gated Channel Subfamily H Member 5 | Protein Coding | GC14M062699 | 1.97 |
| TRIM22 | Tripartite Motif Containing 22 | Protein Coding | GC11P005689 | 1.97 |
| IKZF2 | IKAROS Family Zinc Finger 2 | Protein Coding | GC02M213001 | 1.96 |
| VAV2 | Vav Guanine Nucleotide Exchange Factor 2 | Protein Coding | GC09M133761 | 1.96 |
| VAMP5 | Vesicle Associated Membrane Protein 5 | Protein Coding | GC02P085589 | 1.96 |
| EVPL | Envoplakin | Protein Coding | GC17M076004 | 1.96 |
| USP9Y | Ubiquitin Specific Peptidase 9 Y-Linked | Protein Coding | GC0YP012543 | 1.96 |
| MPST | Mercaptopyruvate Sulfurtransferase | Protein Coding | GC22P037019 | 1.96 |
| CD74 | CD74 Molecule | Protein Coding | GC05M150378 | 1.96 |
| EXOC6 | Exocyst Complex Component 6 | Protein Coding | GC10P092826 | 1.96 |
| HSALNG0063781 |  | RNA Gene | GC08P019960 | 1.95 |
| UGT1A10 | UDP Glucuronosyltransferase Family 1 Member A10 | Protein Coding | GC02P233636 | 1.95 |
| H3-4 | H3.4 Histone | Protein Coding | GC01M228427 | 1.95 |
| MIR7-1 | MicroRNA 7-1 | RNA Gene | GC09M084057 | 1.95 |
| RF00017-8038 |  | RNA Gene | GC09P133163 | 1.95 |
| IGSF5 | Immunoglobulin Superfamily Member 5 | Protein Coding | GC21P039632 | 1.95 |
| RXFP2 | Relaxin Family Peptide Receptor 2 | Protein Coding | GC13P031739 | 1.95 |
| ENSG00000236838 |  | RNA Gene | GC17P002175 | 1.95 |
| ENSG00000232110 |  | RNA Gene | GC10P089283 | 1.95 |
| LAMP3 | Lysosomal Associated Membrane Protein 3 | Protein Coding | GC03M183122 | 1.95 |
| FGL1 | Fibrinogen Like 1 | Protein Coding | GC08M017864 | 1.95 |
| XKR8 | XK Related 8 | Protein Coding | GC01P027971 | 1.95 |
| S100A13 | S100 Calcium Binding Protein A13 | Protein Coding | GC01M153618 | 1.95 |
| ADAD1 | Adenosine Deaminase Domain Containing 1 | Protein Coding | GC04P122378 | 1.95 |
[truncated: 161,614 more chars]
