# Supplementary material for: Proangiogenesis effects of compound danshen dripping pills in zebrafish
Source: BMC Complement Med Ther. 2022 Apr 22;22:112. doi: 10.1186/s12906-022-03589-y (PMC9034551; doi:10.1186/s12906-022-03589-y)
Supplement: Supplementary file 9 — Additional file 9. Table 8. Screenedtarget genes of CDDP for CHD treatment. In total, 65 potential target genes of CDDP forCHD treatment were identified. [file 12906_2022_3589_MOESM9_ESM.docx]

**Supplementary Table 8.** Screened target genes of CDDP for CHD treatment.

| **#** | **Gene symbol** |
| --- | --- |
| 1 | ACHE |
| 2 | ADRA1A |
| 3 | ADRA1B |
| 4 | ADRA2A |
| 5 | ADRB2 |
| 6 | AKR1B1 |
| 7 | AKT1 |
| 8 | AR |
| 9 | BCL2 |
| 10 | BCL2L1 |
| 11 | BIRC5 |
| 12 | CASP3 |
| 13 | CASP9 |
| 14 | CCNB1 |
| 15 | CCND1 |
| 16 | CD40LG |
| 17 | CDKN1A |
| 18 | CHRM1 |
| 19 | CHRM2 |
| 20 | CHRM3 |
| 21 | CYP1A1 |
| 22 | CYP1A2 |
| 23 | CYP3A4 |
| 24 | EGFR |
| 25 | ERBB2 |
| 26 | ESR1 |
| 27 | F7 |
| 28 | FOS |
| 29 | GABRA1 |
| 30 | GSTP1 |
| 31 | HMOX1 |
| 32 | ICAM1 |
| 33 | IFNG |
| 34 | IL10 |
| 35 | IL2 |
| 36 | IL6 |
| 37 | INSR |
| 38 | JUN |
| 39 | KCNH2 |
| 40 | MAPK1 |
| 41 | MMP1 |
| 42 | MMP2 |
| 43 | MMP9 |
| 44 | MYC |
| 45 | NCOA1 |
| 46 | NCOA2 |
| 47 | NFKBIA |
| 48 | NR1I2 |
| 49 | NR3C2 |
| 50 | OPRM1 |
| 51 | PGR |
| 52 | PPARG |
| 53 | PRSS1 |
| 54 | PTGS1 |
| 55 | PTGS2 |
| 56 | RB1 |
| 57 | RELA |
| 58 | RXRA |
| 59 | SCN5A |
| 60 | SLC2A4 |
| 61 | SLC6A2 |
| 62 | SLC6A4 |
| 63 | TNFSF15 |
| 64 | TOP1 |
| 65 | VEGFA |
